# Predicting high risk births with contraceptive prevalence and contraceptive method-mix in an ecologic analysis

**DOI:** 10.1186/s12889-017-4741-6

**Published:** 2017-11-07

**Authors:** Jamie Perin, Agbessi Amouzou, Neff Walker

**Affiliations:** Department of International Health, Johns Hopkins Bloomberg School of Public Health, 615 North Wolfe Street, Baltimore, MD USA

**Keywords:** Contraception, Lives Saved Tool, LiST, Hierarchical modeling, Contraception prevalence rate, Birth risk, Method-mix

## Abstract

**Background:**

Increased contraceptive use has been associated with a decrease in high parity births, births that occur close together in time, and births to very young or to older women. These types of births are also associated with high risk of under-five mortality. Previous studies have looked at the change in the level of contraception use and the average change in these types of high-risk births. We aim to predict the distribution of births in a specific country when there is a change in the level and method of modern contraception.

**Methods:**

We used data from full birth histories and modern contraceptive use from 207 nationally representative Demographic and Health Surveys covering 71 countries to describe the distribution of births in each survey based on birth order, preceding birth space, and mother’s age at birth. We estimated the ecologic associations between the prevalence and method-mix of modern contraceptives and the proportion of births in each category. Hierarchical modelling was applied to these aggregated cross sectional proportions, so that random effects were estimated for countries with multiple surveys. We use these results to predict the change in type of births associated with scaling up modern contraception in three different scenarios.

**Results:**

We observed marked differences between regions, in the absolute rates of contraception, the types of contraceptives in use, and in the distribution of type of birth. Contraceptive method-mix was a significant determinant of proportion of high-risk births, especially for birth spacing, but also for mother’s age and parity. Increased use of modern contraceptives is especially predictive of reduced parity and more births with longer preceding space. However, increased contraception alone is not associated with fewer births to women younger than 18 years or a decrease in short-spaced births.

**Conclusions:**

Both the level and the type of contraception are important factors in determining the effects of family planning on changes in distribution of high-risk births. The best predictions for how birth risk changes with increased modern contraception and for different contraception methods allow for more nuanced predictions specific to each country and can aid better planning for the scaling up of modern contraception.

**Electronic supplementary material:**

The online version of this article (10.1186/s12889-017-4741-6) contains supplementary material, which is available to authorized users.

## Background

The Lives Saved Tool (LiST) was designed to estimate the impact of a small set of interventions on child mortality. Over time the scope has increased to include a broader range of interventions and outputs (e.g., maternal mortality, stillbirths, stunting). For the last 5 years, LiST has also sought to accurately capture the effect of family planning on child survival [1]. The Lives Saved Tool is built inside the Spectrum software package, which includes both a demographic module and a family planning module (FamPlan) [2]. The FamPlan module is a family planning software package that allows users to estimate the impact of scaling up family planning programs on fertility. The software allows users to scale up contraception usage and/or method-mix and then estimates the reduction in fertility. This change in fertility is passed to LiST, resulting in fewer births and consequently fewer maternal deaths and also fewer child deaths. This does not have an impact on the maternal mortality ratio or neonatal or under-five mortality rates.

Family planning may also influence mortality through redistribution of births to types with lower risk of child mortality. There is an extensive literature that links births under different conditions to increased mortality risk [3]. These “risky” births have been identified primarily by three factors: mother’s age, birth order or parity of births, and interval of time between births or pregnancies. The risk of mortality for the child is generally greater when the mother is young (defined as under 18 years of age) or older (over 34 years of age) [4–6]. First births as well as higher parity births also are associated with higher mortality risk than those with medium parity, although first births also tend to have higher risk [7–9]. In addition, births that occur within 18 or 24 months of the birth of their next oldest sibling are at higher risk for mortality than births with longer inter-birth intervals [10–13], although very long intervals may also be associated with excess under five mortality [14].

Family planning could potentially reduce mortality among children under-five by reducing the proportion of births in these high-risk categories. For example, women might use contraception to delay pregnancy, resulting in more time between pregnancies. Stover and Ross [15] recently analyzed data from 194 Demographic and Household Surveys to quantify the link between total fertility rate, contraceptive prevalence and the distribution of births in high-risk categories. This was an ecological analysis, where each survey was aggregated for an estimate of total fertility, contraceptive prevalence rate and births into risk (or no risk) categories. They found that the proportion of births in some high-risk categories declined as contraceptive prevalence increased. For example, the proportion of high parity births (defined as births of order five or more) decreased as contraceptive prevalence increased. In addition, the proportion of births to women aged 35 years or older also decreased as fertility dropped. However, not all of the high-risk births decreased with fertility. As one would expect, the proportion of births that were first births increased as fertility dropped.

An interesting finding from this analysis [15] was the non-association between contraceptive use and the proportions of births that are short spaced, suggesting that contraception may be used mostly for fertility limiting rather than spacing purposes. Overall, the proportion of births that were short-spaced did not change significantly as fertility decreased. In a sub-analysis, Stover and Ross found a slight difference in patterns between modern contraception prevalence (mCPR) and birth spacing for countries in sub-Saharan Africa compared to Asia. They hypothesized that this could be due to different types of contraceptive methods common in the two regions. Specifically, because permanent methods are strictly fertility limiting, they may control fertility and lead to a reduction in high parity births and births to older mothers, while not having an impact on birth spacing. One purpose of this work was to investigate the differential impact of contraceptive method-mix on the relationship between contraceptive use and the proportion of births that are high risk.

The user of FamPlan can develop models using various options, setting goals for contraception, reducing unmet need, and by changing contraceptive method-mix. Based on these inputs FamPlan re-estimates births and the distribution of births into risk categories defined by mothers’ age, parity and birth spacing. These values are then passed to the Lived Saved Tool, where categories of risk are linked to sub-optimal birth outcomes (preterm and appropriate size for gestational age, term and small for gestational age, and preterm and small for gestational age) and from there to increased risk of neonatal mortality and later stunting and then to mortality risk. This work builds on the work of Stover and Ross [15] and further investigates the relationship between modern contraceptive prevalence rate and the distribution of births into risk categories. Our analyses examine two primary aspects. First, we investigate how contraceptive method-mix alters the relationship between contraceptive use and distribution of birth risk. Second, we investigate possible country specific differences and how these affect the relationship between mCPR and birth risk. We recommend these findings to refine the linkages between family planning, birth outcomes, and mortality risk currently in the Lives Saved Tool.

## Methods

### Data sets

We used data from Demographic and Health Surveys (DHS) carried out in low- and middle-income countries to inform the type of birth we expect on average with changing mCPR and total fertility rate (TFR). We examined all publicly available surveys from DHS phase II and later, including 207 surveys covering 71 countries. These surveys represent an extensive library available to the public for research purposes starting in the early 1980s, including information about the timing and circumstances of recent births as well as current contraceptive practices among women of child-bearing age [16].

### Analyses

Method-mix in each survey was specified by *% permanent,* the percent of women using permanent methods of contraception (including tubal ligation and vasectomy) among those that use modern contraception and *% long acting and reversible,* the percent of contracepting women who used IUDs or implants. We described the risk category for each birth in the 3 years prior to each survey. Assessment of risk was based on three characteristics: birth spacing, mother’s age at birth, and parity. We divided birth spacing into four categories based on mortality risk: preceding birth space less than 18 months, preceding space 18 to 24 months, preceding space 24 to 36 months, and preceding space 36 months or longer. We described mother’s age in three categories: less than 18 years, 18 to 34 years, and 35 or more years. For parity, we categorized births as first births, second to fourth births, or fifth and later births. We predicted average changes in birth risk across surveys with three different models with increasing complexity. In the first model we predicted changes in birth risk with changing mCPR using a log linear regression for each risk category. For the second model we estimated these relationships between mCPR and percent of births in each category using log linear regression while adjusting for method-mix. For the third model, we modelled associations between the national percent of births in each risk category and rates of mCPR using multilevel log linear regression with random effects for country of survey. We transformed the percent in each risk category with the natural logarithm as the independent response. For each birth risk category, and the expected percent of births in that category ***p***_*i*_ in country *i*, the relationships between percent at risk (***p***_*i*_), mCPR, and method-mix are modelled in this framework by

$$ \log \left({\boldsymbol{p}}_i\right)={\boldsymbol{\upbeta}}_0+{\boldsymbol{\upbeta}}_1\ \mathrm{mCPR}+{\boldsymbol{\upbeta}}_2\ \left(\% permanent\right)+{\boldsymbol{\upbeta}}_3\ \left(\% long acting\  and\  reversible\right)+{\mathbf{b}}_{\mathrm{i}} $$

where **b**_i_ is specific to each country, and regression coefficients **β**_0_, **β**_1_, **β**_2_, and **β**_3_ are associations common to all countries. In each dimension of risk (birth order, birth space, and mother’s age), several ***p***_*i*_ are modelled for each category. The resulting predicted percentages are scaled to cover 100% of births.

This model for each ***p***_*i*_ used empirical Bayesian methods for multilevel data to further refine predictions of birth risk distribution with the random effect **b**_i_ [17]. Although surveys are cross sectional, there are often multiple surveys in the same country over time, covering the same geography among different individuals. Hierarchical models are commonly used to predict outcomes in individuals over time [18], although similar hierarchical models have also been used to predict population level characteristics, including TFR [19, 20] and the causes of maternal mortality [21]. A hierarchical model estimates effects within units or clustered observations, in our case, for countries. A complete treatment of hierarchical methods is available from Gelman and Hill [22] and McCullogh and Neuhaus [23]. These predictive models allow for country-specific interpretation of the effects of mCPR [24], while incorporating information from all countries in the general relationship between mCPR and type of birth.

We also examined the relationship between birth risk distribution and mCPR in order to demonstrate the relative advantage of country-specific methods. We estimated the average linear association across survey data, and compared with results from hierarchical modeling with country-specific effects. We examined the predicted birth risk distribution for a selection of low- and middle-income countries. We also predicted the percent of births in each of the categories described above in these countries for three scenarios: (A) increasing mCPR with permanent methods, (B) increasing mCPR with long acting and reversible methods, and (C) increasing mCPR with short-term methods, that is, modern methods that are not permanent or long acting such as condoms, injections, and pills. We examine these scenarios for an increase in mCPR of 10% from the most recent estimated mCPR.

## Results

**Fig. 1:**
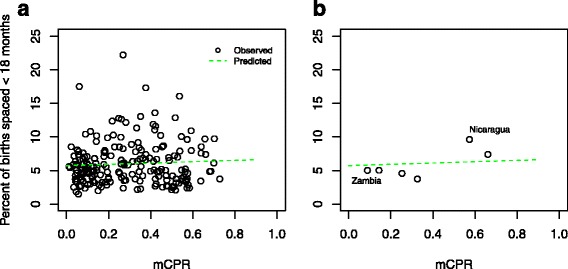
Modern Contraceptive prevalence among women age 15 to 49 years in union, for 71 countries (207 standard DHS surveys), and percent of births with preceding space less than 18 months, from 1990 to 2015. All 207 surveys are shown in (**a**), along with the least squares predicted percent of births, in green. Seven surveys from Zambia (1992 to 2007) and Nicaragua (1998 to 2001) are shown in (**b**)

A summary of the average percent of births in each of these categories from the 3 years prior to survey is shown in Table 1 for the eight regions defined by the millennium development goals. There is substantial variation across regions, with average measured mCPR ranging from 17% of women age 15 to 49 years in sub-Saharan Africa, to 48% in Latin America and the Caribbean. In addition, areas with similar mCPR can have different distributions of birth risk. For example, surveys from western Asia and developed countries have similar rates of mCPR, however, developed areas have the smallest percent of births with a short preceding space, at 3.8%, while Western Asia has the largest, at 15%. Figure 1 shows the percent of births with very short spaces against mCPR for all surveys, and two purposively selected countries for illustration whose trend over time runs counter to the average (Fig. 1 (b)). The dashed line represents the percent of births with a short space predicted by all surveys on average.

**Table 1:** Summary of household surveys by MDG region, with average birth risk distribution as a percent of births in the three most recent years of birth history, TFR in the three prior years and cross-sectional mCPR for 207 surveys conducted since 1990

						Mother’s Age (%)			Birth Spacing (%)				Birth Order (%)		
	N Surveys	Year (Average)	Births (thousands)	TFR Mean (range)	mCPR (%)	< 18 years	18–34 years	≥ 35 years	< 18 mo.	18–23 mo.	24–35 mo.	≥ 36 mo.	First	2nd to 4th	5th or later
Caucasus and Central Asia	10	2003	14	2.6 (1.7–3.8)	34.2	1.7	91.0	7.3	8.2	10.5	15.1	66.1	38.1	47.2	14.7
Developed^*^	3	2007	3	1.5 (1.2–1.7)	34.0	2.7	91.0	6.3	3.8	4.2	8.1	83.9	46.5	46.6	6.9
Latin America and the Caribbean	37	2001	179	3.3 (2.1–5.1)	48.1	8.2	78.9	12.9	6.9	9.6	18.8	64.7	31.1	40.1	28.8
Northern Africa	10	1999	48	3.1 (2.2–4.0)	46.3	3.2	85.1	11.7	7.4	8.8	20.0	63.9	28.7	40.7	30.5
South-eastern Asia	17	2002	110	3.2 (1.9–5.7)	41.1	2.9	81.8	15.2	6.0	8.5	17.7	67.8	30.2	41.0	28.8
Southern Asia	16	2002	156	3.4 (2.3–4.9)	37.6	10.3	82.7	7.1	6.4	9.1	21.9	62.6	28.3	40.6	31.1
Sub-Saharan Africa	107	2003	503	5.4 (2.5–7.6)	17.0	7.0	78.4	14.6	4.8	8.7	28.2	58.3	21.9	33.8	44.3
Western Asia	7	2001	36	4.6 (3.5–7.7)	34.0	2.1	81.1	16.8	15.0	14.7	22.3	48.0	19.5	34.6	45.9
Overall	207	2002	1049	4.4 (1.2–7.7)	29.2	6.5	80.3	13.2	6.0	9.1	23.6	61.3	26.1	37.2	36.6

^*^Moldova 2005, Ukraine 2007, and Albania 2008

The estimated associations between mCPR and percent at risk for three different models are shown in Table 2: on average for all surveys with log linear regression (Model 1), on average across surveys adjusting for method-mix (Model 2), and for those adjusted for method-mix in addition to random effects for each country estimated with multilevel modelling (Model 3).

At the crudest level, model 1 indicates no statistically significant association between mCPR and short birth intervals (<18 months) and births to younger women aged less than 18 years. Association with other categories was statistically significant and in the expected direction. For example, fewer births to older women and births with high parity are associated with increased mCPR. However, for birth interval, increased mCPR is associated with reduced percent of births with the optimal preceding interval of 24–35 months. Only the proportion of births with preceding interval of 36 months or more is positively associated with prevalence of mCPR. These relationships change when method-mix is introduced (Model 2). Specifically, the association between short birth interval and prevalence of mCPR changed direction and became statistically significant. Model 3, which accounts for country specific random effects, showed similar results as models 1 and 2. All parameter estimates for the hierarchical model are included in Additional files 1, 2, and 3.

**Table 2:** Estimated associations between birth risk factors and mCPR for (Model 1) ordinary least squares average across surveys with only mCPR; (Model 2) for mCPR change adjusted by method-mix; (Model 3) hierarchical model for change in mCPR within country adjusted for method-mix

		Mother’s Age (%)			Birth Spacing (%)				Birth Order (%)		
		< 18 years	18–34 years	≥ 35 years	< 18 mo.	18–23 mo.	24–35 mo.	≥ 36 mo.	First	2nd to 4th	5th or later
(Model 1) β mCPR (for 10% change)	Est	0.010	0.050	−0.080	0.020	−0.050	−0.170	0.130	0.140	0.090	−0.220
	SE	0.020	0.010	0.010	0.020	0.010	0.010	0.010	0.010	0.010	0.010
	*p*	0.676	< 0.001	< 0.001	0.804	< 0.001	< 0.001	< 0.001	< 0.001	< 0.001	< 0.001
(Model 2) β mCPR (for 10% change)	Est	0.020	0.000	−0.040	−0.090	−0.110	−0.140	0.070	0.100	0.060	−0.140
	SE	0.020	0.000	0.010	0.020	0.010	0.010	0.010	0.010	0.000	0.020
	*p*	0.837	0.965	0.001	< 0.001	< 0.001	< 0.001	< 0.001	< 0.001	< 0.001	< 0.001
(Model 3) β mCPR (for 10% change)	Est	−0.010	0.010	−0.060	−0.110	−0.100	−0.120	0.070	0.090	0.060	−0.140
	SE	0.020	0.000	0.010	0.020	0.010	0.010	0.010	0.010	0.000	0.020
	*p*	0.300	< 0.001	< 0.001	< 0.001	< 0.001	< 0.001	< 0.001	< 0.001	< 0.001	< 0.001

**Fig. 2:**
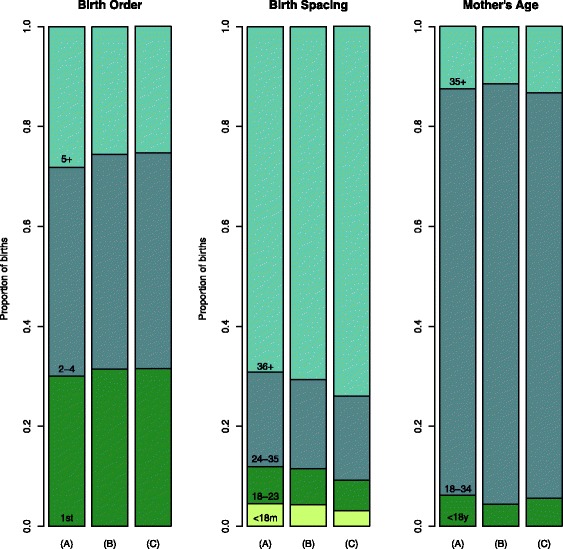
Predicted distribution of preceding birth space, mother’s age, and birth order in Zambia with changes in mCPR depending on three different scenarios for 10% increases in mCPR given most recent survey estimates (from 33% to 43%): (A) increasing mCPR with permanent methods, (B) increasing mCPR with long acting and reversible methods, and (C) increasing mCPR with short term methods

**Fig. 3:**
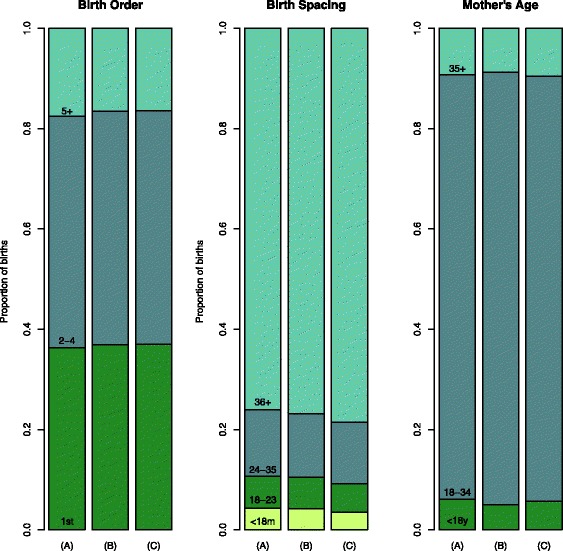
Predicted distribution of preceding birth space, mother’s age, and birth order in Nicaragua with changes in mCPR depending on three different scenarios for 10% increases in mCPR given most recent survey estimates (from 67% to 77%): (A) increasing mCPR with permanent methods, (B) increasing mCPR with long acting and reversible methods, and (C) increasing mCPR with short term methods

Finally, as a way to visualize the impact of different contraceptive methods on the distribution of births into risk categories, we predicted the percent of births in each category described above for three scenarios: (A) increasing mCPR with permanent methods, (B) increasing mCPR with long acting and reversible methods, and (C) increasing mCPR with short-term methods. We made these predictions for Zambia and Nicaragua, starting from the most recently estimated mCPR (33% and 67%, respectively), with predominantly methods that are not permanent or long term, scaling up by 10% among women age 15 to 49 years. Estimated distributions of birth space, birth order, and mother’s age are shown for Zambia in Fig. 2 and for Nicaragua in Fig. 3. Graphical depictions of these predicted changes for all 71 countries in our sample are included in Additional file 1. In both Zambia and Nicaragua, scaling up mCPR with permanent methods in scenario (A) predicts the smallest reduction in births of order five or later, while scaling up by short term methods in scenario (C) has the longest predicted birth intervals. Long acting and reversible methods show effects that are intermediary between permanent methods and short term methods.

## Discussion

We have used recent mCPR and contraception method-mix from nationally representative household surveys to predict the distribution of high-risk births for unobserved ranges of mCPR. This analysis confirmed at a crude level, as in the earlier analyses reported by Stover and Ross [15], that the proportion of short-spaced birth did not decrease with increasing contraceptive prevalence. However, further analyses showed that this seemingly counter-intuitive effect was explained in large part by contraceptive methods used as well as by unmeasured circumstances specific to each country. In addition, projections in Zambia and Nicaragua showed that when contraceptive prevalence is increased using permanent methods, the proportion of births with short intervals is largest compared to when methods that are not permanent or long term are predominant. Prevalence of short birth intervals might be high in countries with predominantly permanent methods if couples use short birth intervals as a strategy for reaching their desired number of children before their fertility is curtailed.

This work builds on previous research on contraceptive method-mix [25], allowing that method-mix be incorporated into LiST for planning and country level analysis. These analyses also provide a basis for improving the model for the impact of family planning on under-five mortality in LiST. We showed that to fully reflect the impact of family planning on under-five mortality we must consider more than the simple relationship between contraceptive prevalence rate and birth risk. Method-mix and country-specific information can be used to more accurately reflect this relationship.

The analytical methods in this analysis examined a relationship between mCPR and proportion of high-risk births that is common across countries, while using hierarchical methods for a specific country to predict how birth risk will change with increased mCPR or with a change in the contraceptive method-mix. This method takes systematic advantage of national data to make predictions within each country. Stark differences were observed between the mCPR rates as well as the distribution of high-risk births for surveys from different regions in our analysis. Specifying the type of contraception being used allows more specific predictions for the percent of high-risk births, and random effects for surveys from the same country gives that country’s data more influence in their prediction than surveys from other countries.

These country specific predictions including contraception method-mix can be incorporated into the framework of the Lives Saved Tool. Every country with a recent national survey including a full birth history has an estimated random effect for each birth risk category (see Additional file 3 for graphical depiction of country estimates). These values can be substituted into equations that predict the percent of births in each category. These equations are determined by user given values for mCPR and method-mix, and estimates for four parameters from the hierarchical model: coefficients for mCPR, permanent methods, and long acting and reversible methods, and an additional country specific intercept or random effect. For each type of risk, the predicted proportions are then scaled to sum to 100% of births, to predict separately the distribution of birth order, birth spacing, and mother’s age. The estimated relative mortality risk for each category is then applied, so that with a change in the distribution of birth risk there is a corresponding change in mortality. There is potential for predicting birth risk in countries without recent survey data, in which case an estimate for country effect would not be available. Although there are limited options in this case, it is possible to substitute an estimate based on a similar country or countries, for example an average of effects by region.

There are limitations to this analysis. We have not specified the overlap of risk factors, for example, short preceding birth space for adolescent mothers, which may be common in some countries and may confer additional risk. There is also evidence that very long spaces between births have an adverse effect on child mortality, and for birth spacing to influence the mortality risk among older siblings, which our current analysis does not specify [10].

In addition to these limitations, contraception use was measured at the time of the survey while proportion of births by risk category was measured in the 3 years preceding survey. Furthermore, the cross sectional nature of household surveys and the delayed effect of contraceptive use on individuals and communities means that the expected change in birth risk with increasing mCPR can be estimated only with ecological associations. Wide variability between observed birth risk in countries and over time suggests that there are unspecified mechanisms influencing the determinants of birth order, spacing, and mother’s age. We are not able to identify those mechanisms, however, we can use information from surveyed households to maximum advantage when predicting birth risk for planning purposes. The best possible predictions are critical as contraception use gains attention in new initiatives such as Family Planning 2020.

## Conclusions

We observed great variety in fertility behavior among surveyed households in more than 70 countries beginning in 1990, in the parity of births, the time between births, and the age of mothers at birth. We used hierarchical modelling to predict how these factors might change with changes in the levels or types of contraception in a given country. This more nuanced predictive capacity can contribute to the planning and understanding of patterns of contraception use, and how the future of family planning can best be tailored for global and country priorities.

## Additional files

Additional file 1: Supplementary material is provided for the hierarchical modelling results. (DOCX 18 kb)

Additional file 2: Graphics for the predictions in the scenarios as shown for Zambia and Nicaragua for 71 countries are included. (PDF 111 kb)

Additional file 3: Additional predictions for how birth spacing will change with mCPR ranging between 20 and 80% for similar scenarios are shown for 71 countries. (PDF 1962 kb)

## Abbreviations

DHS: Demographic and Health Surveys
FamPlan: Family planning module of Spectrum
LiST: Lives Saved Tool
mCPR: Modern contraception prevalence
TFR: Total fertility rate
