# Supplementary material for: Predicting high risk births with contraceptive prevalence and contraceptive method-mix in an ecologic analysis
Source: BMC Public Health. 2017 Nov 7;17(Suppl 4):786. doi: 10.1186/s12889-017-4741-6 (PMC5688497; doi:10.1186/s12889-017-4741-6)
Supplement: Supplementary file 1 — Supplementary material is provided for the hierarchical modelling results. (DOCX 18 kb) [file 12889_2017_4741_MOESM1_ESM.docx]

**Additional file 1**

**Table S1. Estimated ecologic associations between birth risk factors and mCPR across surveys with mCPR adjusted by conceptive method mix in a log linear multilevel model within country (Model 3).**

|  |  |  |  | **Regression Coefficient** | | |
| --- | --- | --- | --- | --- | --- | --- |
| **Domain** | **Birth Risk Category** | **Parameter** | **Description** | **Estimate** | **Std. Error** | ***p*** |
| **Birth Spacing (%)** | Percent of births with preceding space less than 18 months | **β_0_** | (Intercept) | -3.07 | 0.08 | < 0.001 |
|  |  | **β_1_** | mCPR, for 10% change | -0.11 | 0.02 | < 0.001 |
|  |  | **β_2_** | % sterilization, for %10 change | 0.14 | 0.02 | < 0.001 |
|  |  | **β_3_** | % long term, for %10 change | 0.13 | 0.02 | < 0.001 |
|  | Percent of births with preceding space 18 - 23 months | **β_0_** | (Intercept) | -2.44 | 0.06 | < 0.001 |
|  |  | **β_1_** | mCPR, for 10% change | -0.10 | 0.01 | < 0.001 |
|  |  | **β_2_** | % sterilization, for %10 change | 0.08 | 0.02 | < 0.001 |
|  |  | **β_3_** | % long term, for %10 change | 0.07 | 0.01 | < 0.001 |
|  | Percent of births with preceding space 24 - 35 months | **β_0_** | (Intercept) | -1.30 | 0.06 | < 0.001 |
|  |  | **β_1_** | mCPR, for 10% change | -0.12 | 0.01 | < 0.001 |
|  |  | **β_2_** | % sterilization, for %10 change | 0.04 | 0.02 | 0.019 |
|  |  | **β_3_** | % long term, for %10 change | 0.02 | 0.01 | 0.115 |
|  | Percent of births with preceding space 36+ | **β_0_** | (Intercept) | -0.58 | 0.03 | < 0.001 |
|  |  | **β_1_** | mCPR, for 10% change | 0.07 | 0.01 | < 0.001 |
|  |  | **β_2_** | % sterilization, for %10 change | -0.04 | 0.01 | < 0.001 |
|  |  | **β_3_** | % long term, for %10 change | -0.02 | 0.01 | < 0.001 |
| **Mothers Age (%)** | Percent of births with mother's age < 18 years | **β_0_** | (Intercept) | -2.88 | 0.12 | < 0.001 |
|  |  | **β_1_** | mCPR, for 10% change | -0.01 | 0.02 | 0.598 |
|  |  | **β_2_** | % sterilization, for %10 change | 0.05 | 0.03 | 0.107 |
|  |  | **β_3_** | % long term, for %10 change | -0.09 | 0.02 | < 0.001 |
|  | Percent of births with mother's age 18 - 34 years | **β_0_** | (Intercept) | -0.27 | 0.01 | < 0.001 |
|  |  | **β_1_** | mCPR, for 10% change | 0.01 | 0.00 | < 0.001 |
|  |  | **β_2_** | % sterilization, for %10 change | 0.00 | 0.00 | 0.325 |
|  |  | **β_3_** | % long term, for %10 change | 0.02 | 0.00 | < 0.001 |
|  | Percent of births with mother's age 35+ years | **β_0_** | (Intercept) | -1.77 | 0.06 | < 0.001 |
|  |  | **β_1_** | mCPR, for 10% change | -0.06 | 0.01 | < 0.001 |
|  |  | **β_2_** | % sterilization, for %10 change | -0.03 | 0.01 | 0.066 |
|  |  | **β_3_** | % long term, for %10 change | -0.06 | 0.01 | < 0.001 |
| **Birth Order (%)** | Percent of births with first parity | **β_0_** | (Intercept) | -1.61 | 0.04 | < 0.001 |
|  |  | **β_1_** | mCPR, for 10% change | 0.09 | 0.01 | < 0.001 |
|  |  | **β_2_** | % sterilization, for %10 change | -0.02 | 0.01 | 0.085 |
|  |  | **β_3_** | % long term, for %10 change | 0.01 | 0.01 | 0.586 |
|  | Percent of births with parity second to fourth | **β_0_** | (Intercept) | -1.17 | 0.02 | < 0.001 |
|  |  | **β_1_** | mCPR, for 10% change | 0.06 | 0.00 | < 0.001 |
|  |  | **β_2_** | % sterilization, for %10 change | -0.01 | 0.01 | 0.048 |
|  |  | **β_3_** | % long term, for %10 change | 0.01 | 0.01 | 0.333 |
|  | Percent of births with parity fifth or later | **β_0_** | (Intercept) | -0.85 | 0.09 | < 0.001 |
|  |  | **β_1_** | mCPR, for 10% change | -0.14 | 0.02 | < 0.001 |
|  |  | **β_2_** | % sterilization, for %10 change | 0.05 | 0.02 | 0.022 |
|  |  | **β_3_** | % long term, for %10 change | 0.01 | 0.02 | 0.415 |
