# Supplementary material for: Predicting high risk births with contraceptive prevalence and contraceptive method-mix in an ecologic analysis
Source: BMC Public Health. 2017 Nov 7;17(Suppl 4):786. doi: 10.1186/s12889-017-4741-6 (PMC5688497; doi:10.1186/s12889-017-4741-6)
Supplement: Supplementary file 2 — Graphics for the predictions in the scenarios as shown for Zambia and Nicaragua for 71 countries are included. (PDF 111 kb) [file 12889_2017_4741_MOESM2_ESM.pdf]

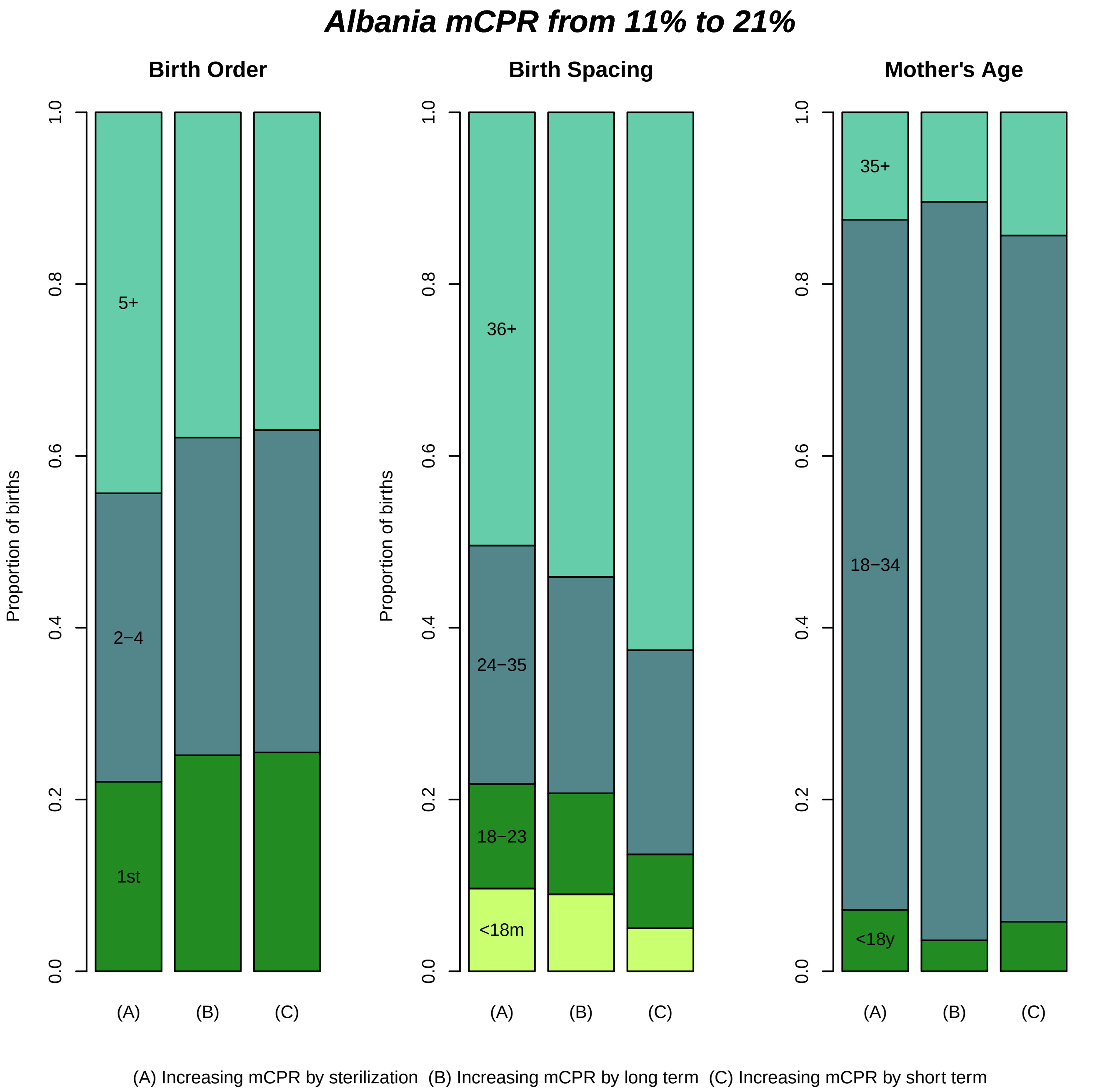

Albania mCPR from 11% to 21%
Birth Order
Birth Spacing
Mother's Age
Proportion of births
0.0
0.2
0.4
0.6
0.8
1.0
5+
2−4
1st
36+
24−35
18−23
<18m
35+
18−34
<18y
(A)
(B)
(C)
(A) Increasing mCPR by sterilization (B) Increasing mCPR by long term (C) Increasing mCPR by short term

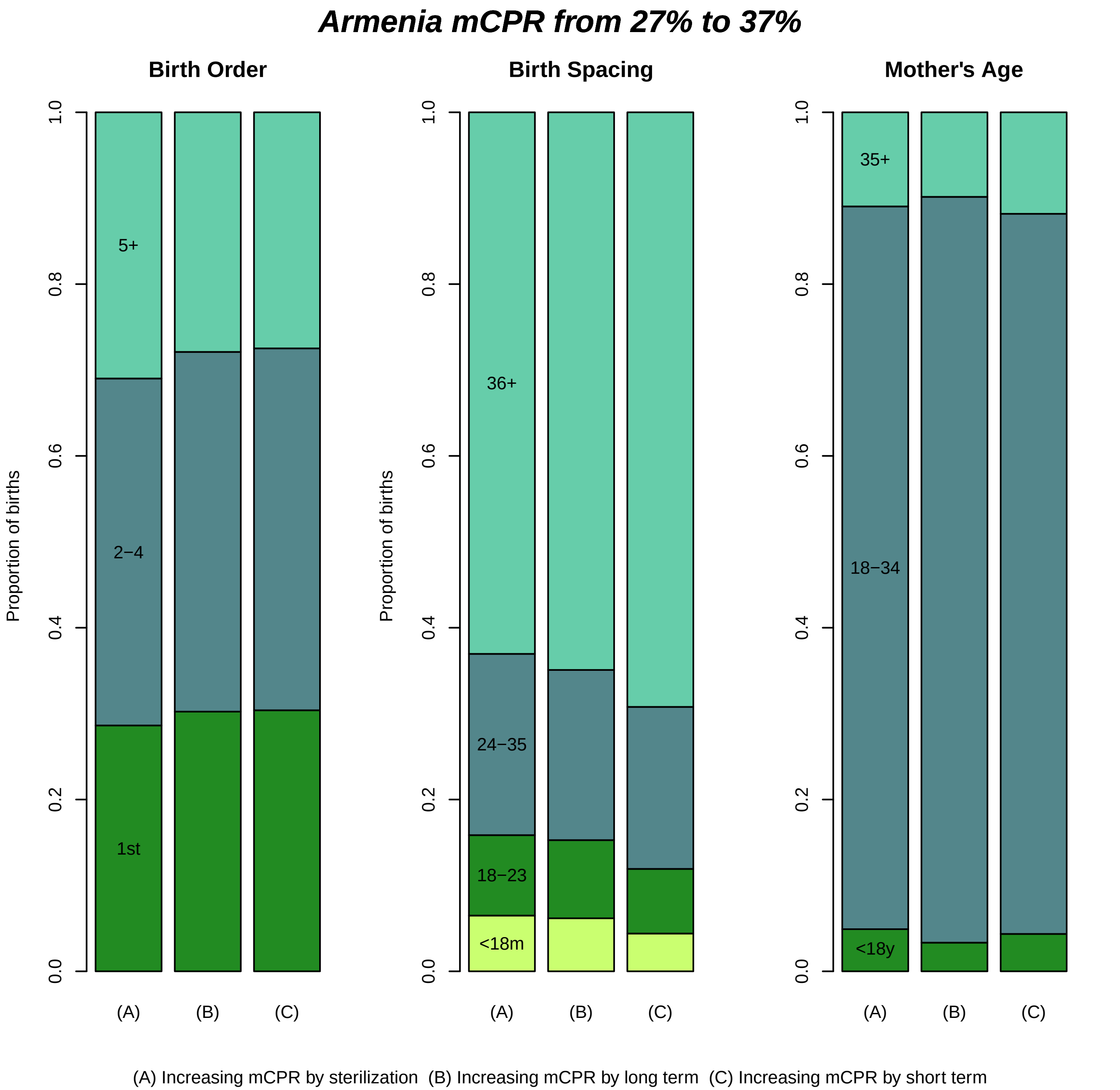

Armenia mCPR from 27% to 37%
Birth Order
Birth Spacing
Mother's Age
Proportion of births
0.0
0.2
0.4
0.6
0.8
1.0
5+
2−4
1st
36+
24−35
18−23
<18m
35+
18−34
<18y
(A)
(B)
(C)
(A) Increasing mCPR by sterilization (B) Increasing mCPR by long term (C) Increasing mCPR by short term

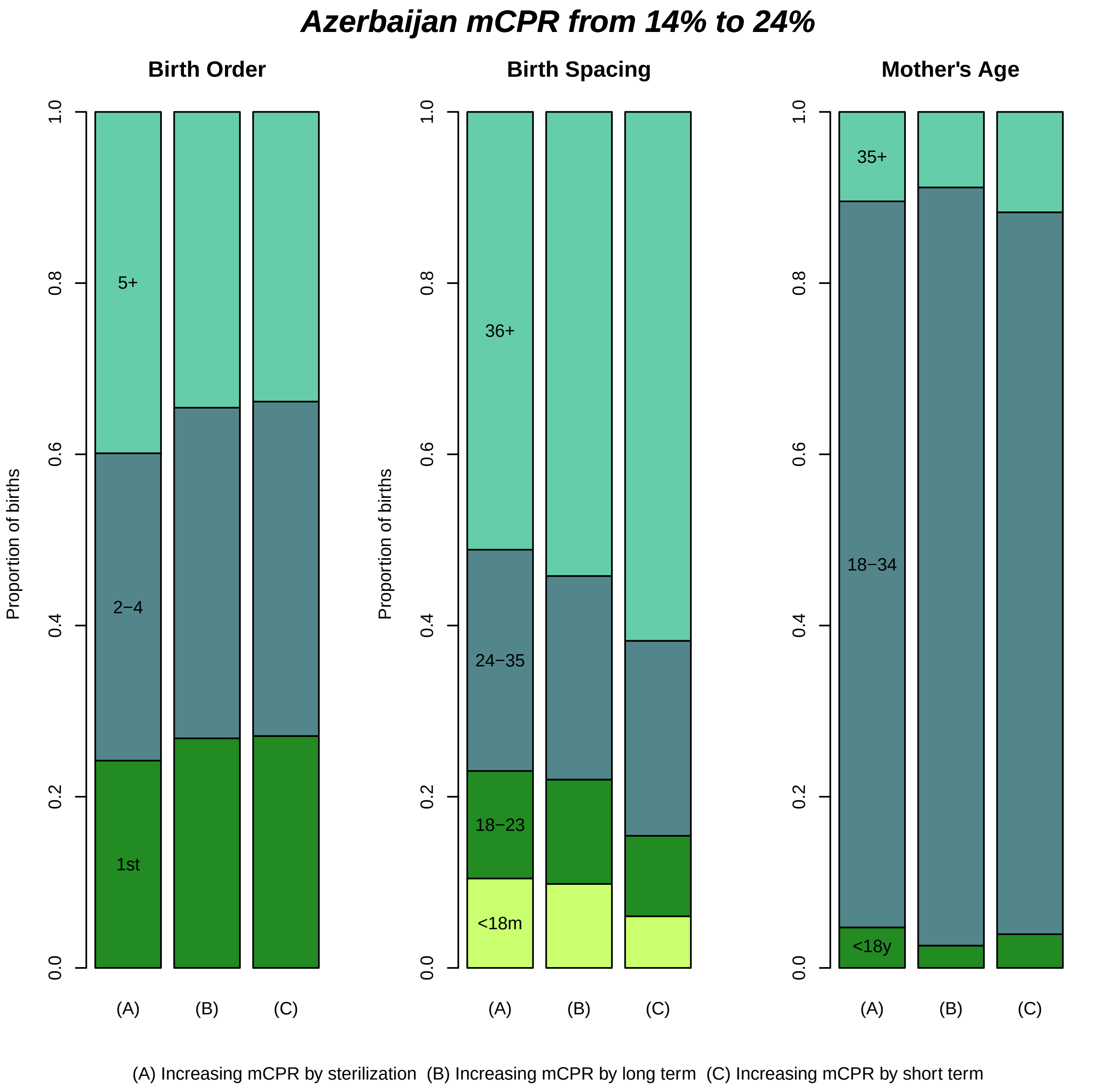
Azerbaijan mCPR from 14% to 24%
Birth Order
Birth Spacing
Mother's Age
Proportion of births
Proportion of births
1.0
0.8
0.6
0.4
0.2
0.0
5+
2−4
1st
36+
24−35
18−23
<18m
35+
18−34
<18y
(A)
(B)
(C)
(A) Increasing mCPR by sterilization (B) Increasing mCPR by long term (C) Increasing mCPR by short term

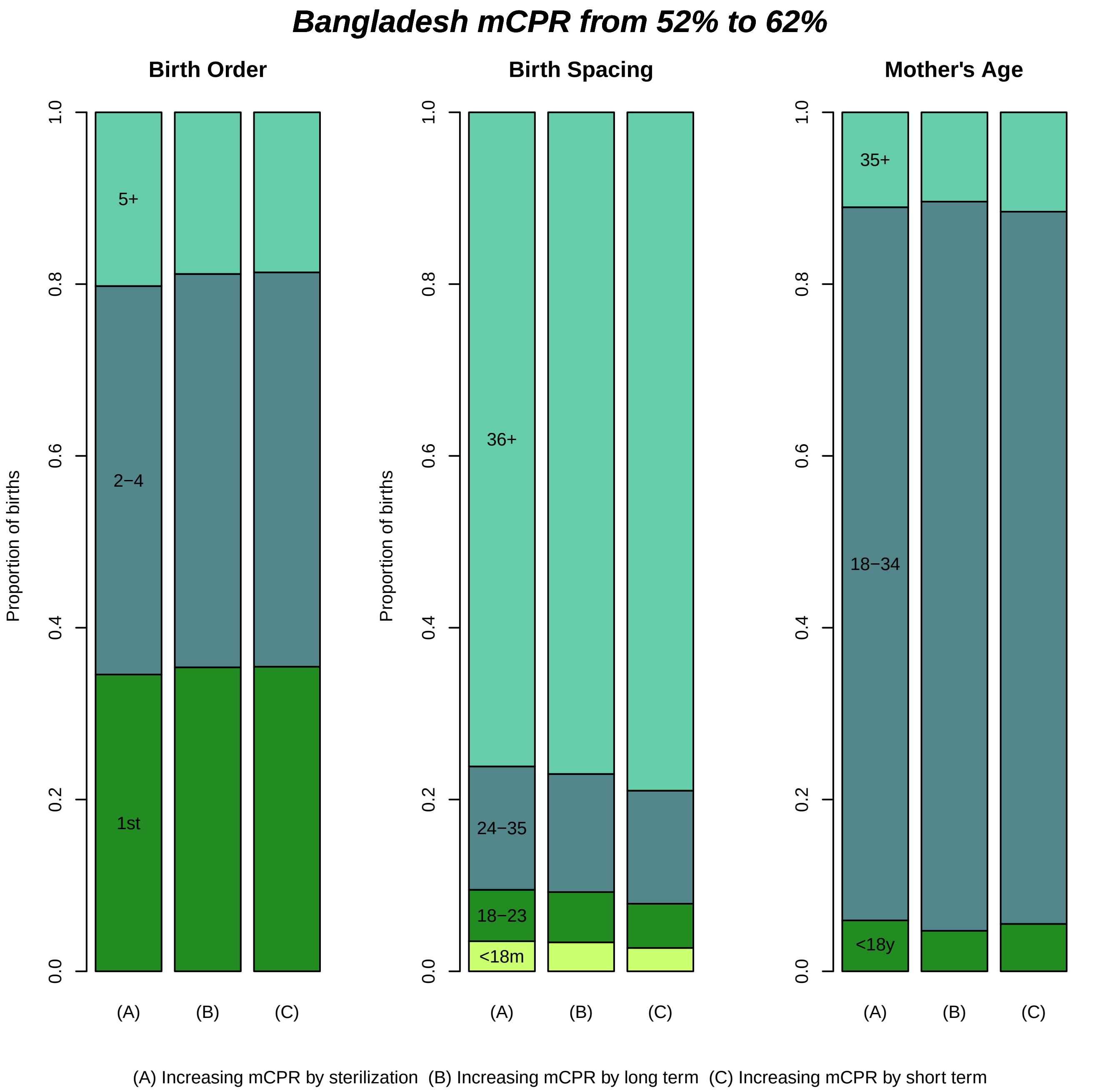
Bangladesh mCPR from 52% to 62%
Birth Order
Birth Spacing
Mother's Age
Proportion of births
1.0
0.8
0.6
0.4
0.2
0.0
5+
2−4
1st
36+
24−35
18−23
<18m
35+
18−34
<18y
(A)
(B)
(C)
(A) Increasing mCPR by sterilization (B) Increasing mCPR by long term (C) Increasing mCPR by short term

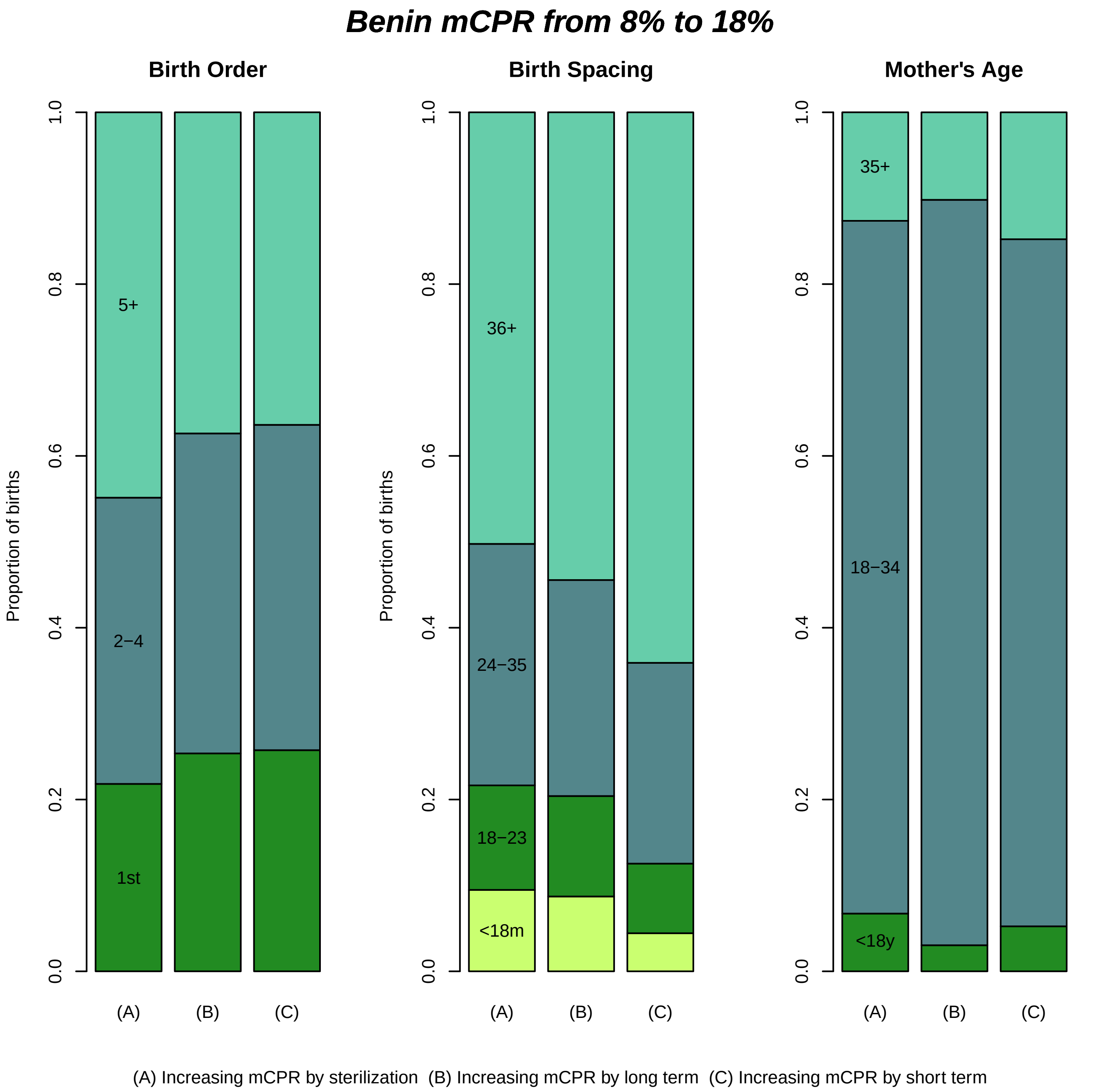
Benin mCPR from 8% to 18%
Birth Order
Birth Spacing
Mother's Age
Proportion of births
0.0
0.2
0.4
0.6
0.8
1.0
5+
2−4
1st
36+
24−35
18−23
<18m
35+
18−34
<18y
(A)
(B)
(C)
(A) Increasing mCPR by sterilization (B) Increasing mCPR by long term (C) Increasing mCPR by short term

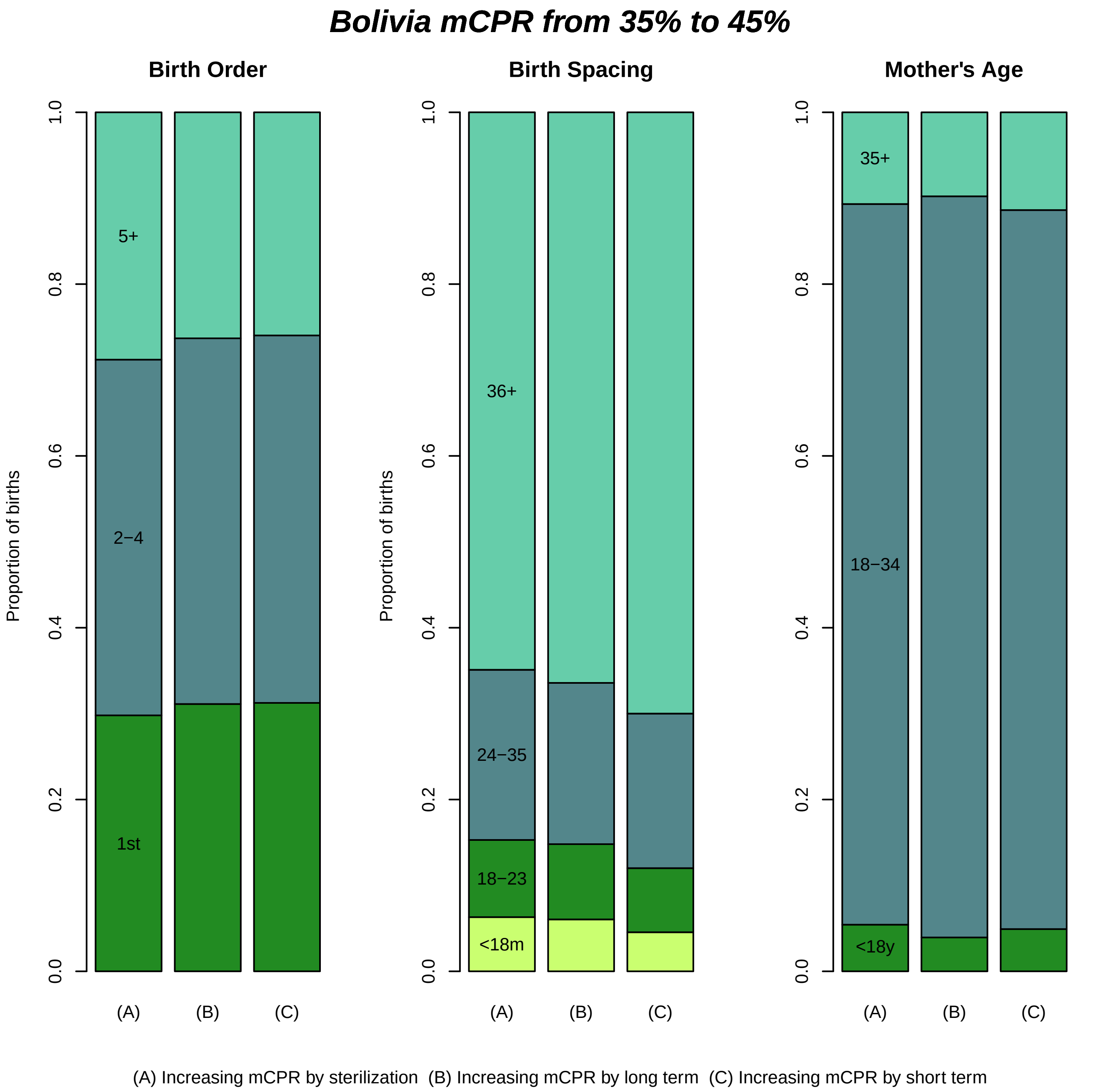
Bolivia mCPR from 35% to 45%
Birth Order
Birth Spacing
Mother's Age
Proportion of births
0.0
0.2
0.4
0.6
0.8
1.0
5+
2−4
1st
36+
24−35
18−23
<18m
35+
18−34
<18y
(A)
(B)
(C)
(A) Increasing mCPR by sterilization (B) Increasing mCPR by long term (C) Increasing mCPR by short term

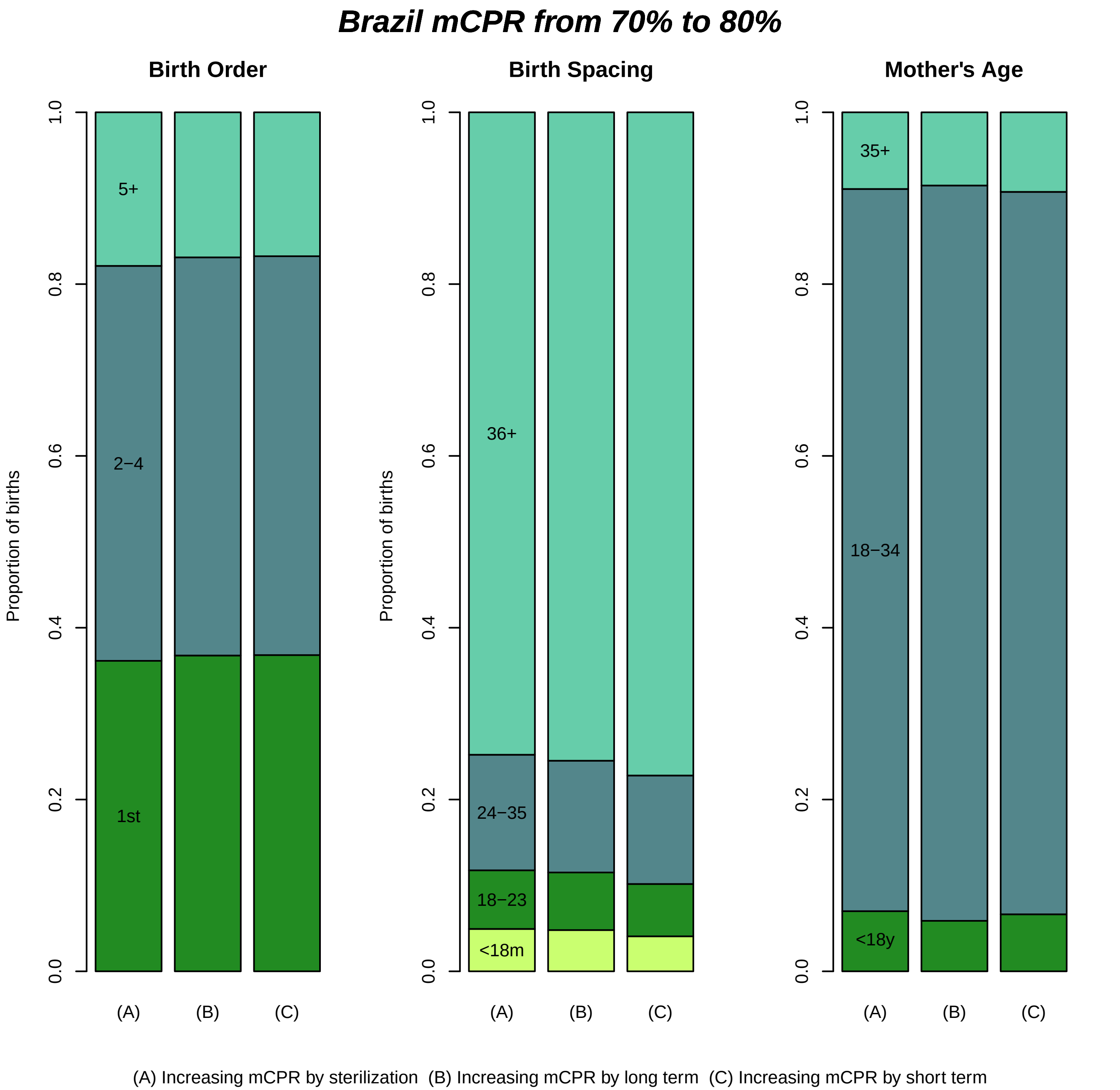

Brazil mCPR from 70% to 80%
Birth Order
Birth Spacing
Mother's Age
Proportion of births
0.0
0.2
0.4
0.6
0.8
1.0
5+
2−4
1st
36+
24−35
18−23
<18m
35+
18−34
<18y
(A)
(B)
(C)
(A) Increasing mCPR by sterilization (B) Increasing mCPR by long term (C) Increasing mCPR by short term

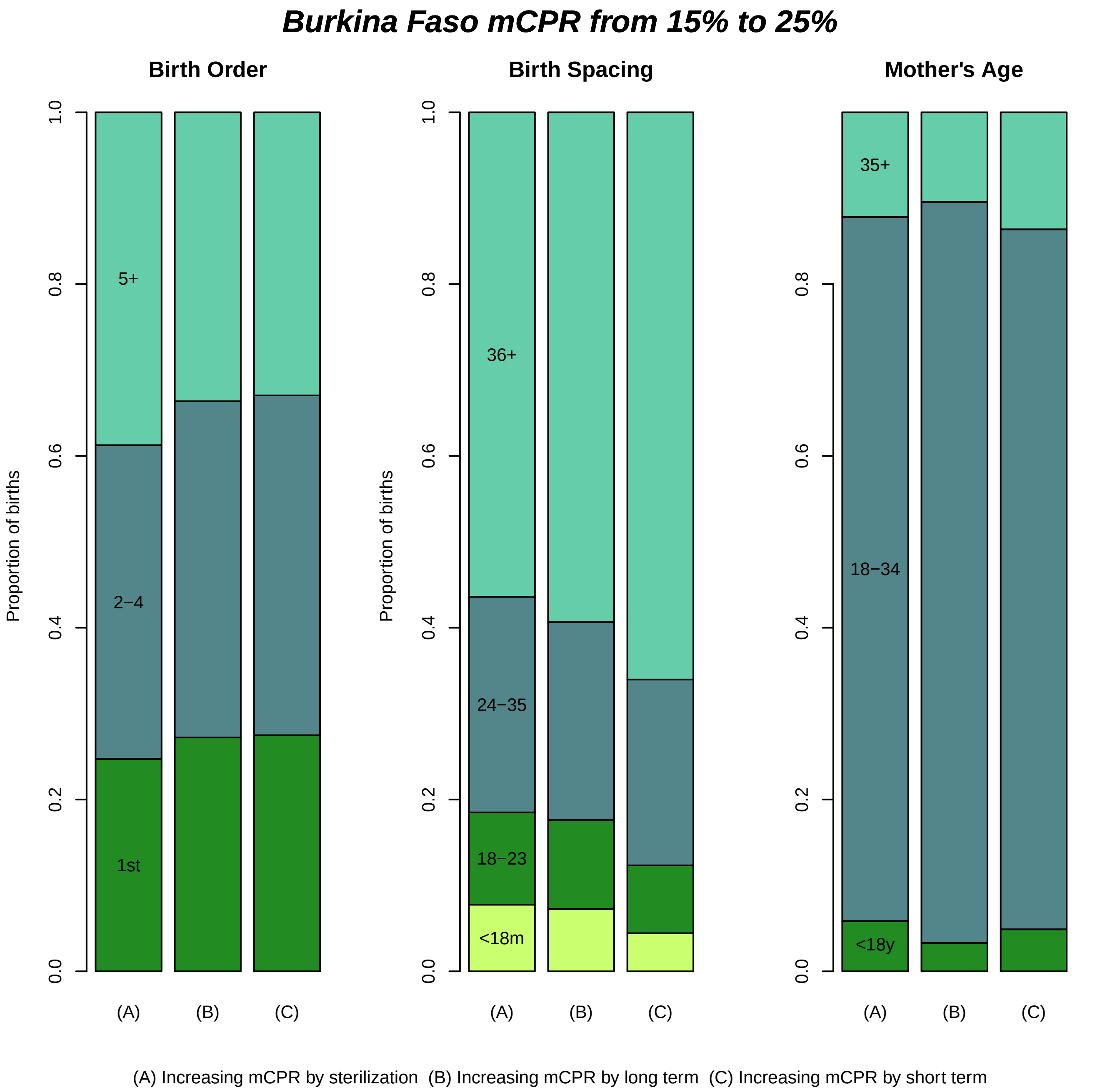

Burkina Faso mCPR from 15% to 25%
Birth Order
Birth Spacing
Mother's Age
Proportion of births
0.0
0.2
0.4
0.6
0.8
1.0
5+
2−4
1st
36+
24−35
18−23
<18m
35+
18−34
<18y
(A)
(B)
(C)
(A) Increasing mCPR by sterilization (B) Increasing mCPR by long term (C) Increasing mCPR by short term

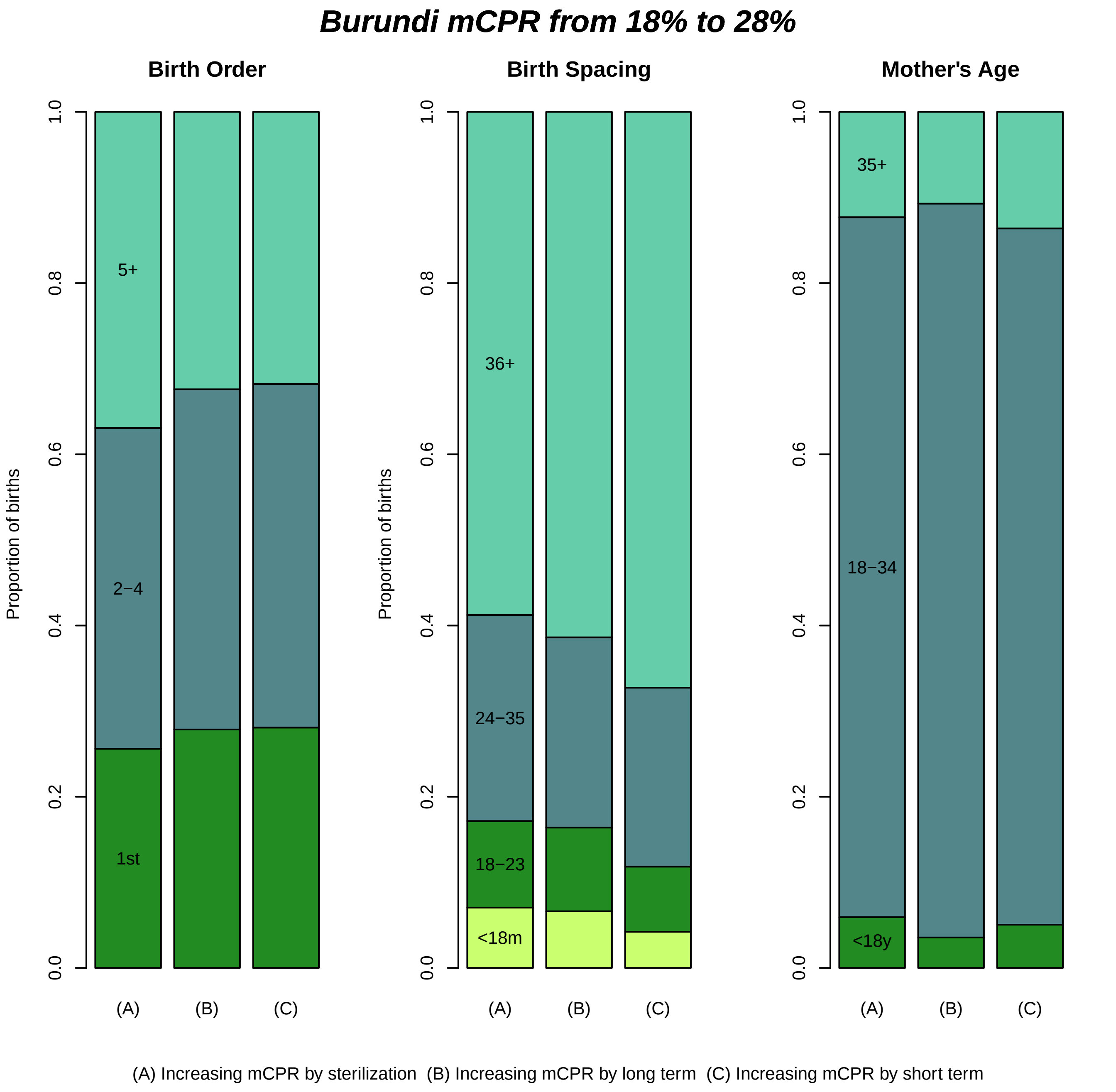
Burundi mCPR from 18% to 28%
Birth Order
Birth Spacing
Mother's Age
Proportion of births
Proportion of births
1.0
0.8
0.6
0.4
0.2
0.0
5+
2−4
1st
36+
24−35
18−23
<18m
35+
18−34
<18y
(A)
(B)
(C)
(A) Increasing mCPR by sterilization (B) Increasing mCPR by long term (C) Increasing mCPR by short term

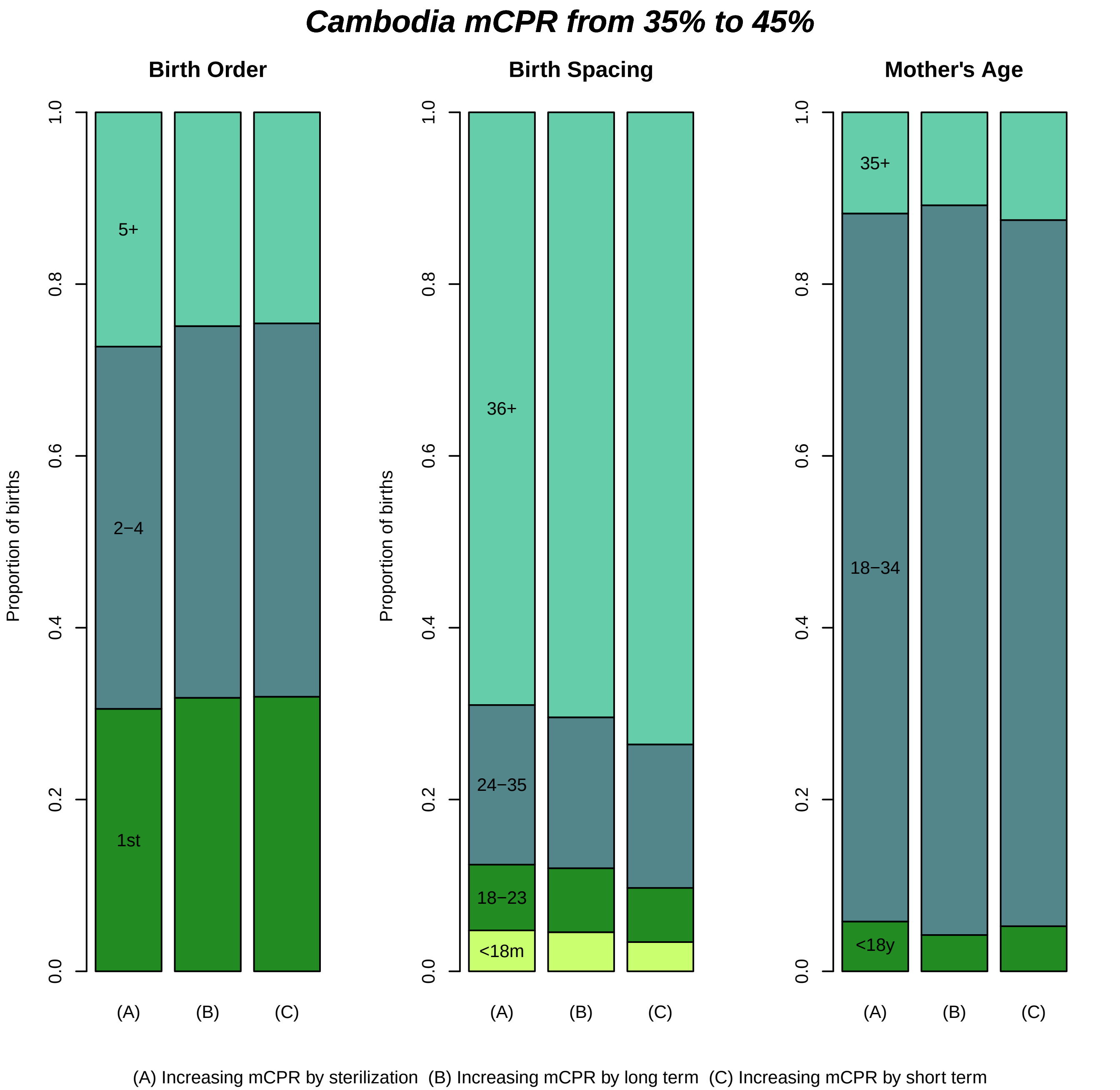
Cambodia mCPR from 35% to 45%
Birth Order
Birth Spacing
Mother's Age
Proportion of births
0.0
0.2
0.4
0.6
0.8
1.0
5+
2−4
1st
36+
24−35
18−23
<18m
35+
18−34
<18y
(A)
(B)
(C)
(A) Increasing mCPR by sterilization (B) Increasing mCPR by long term (C) Increasing mCPR by short term

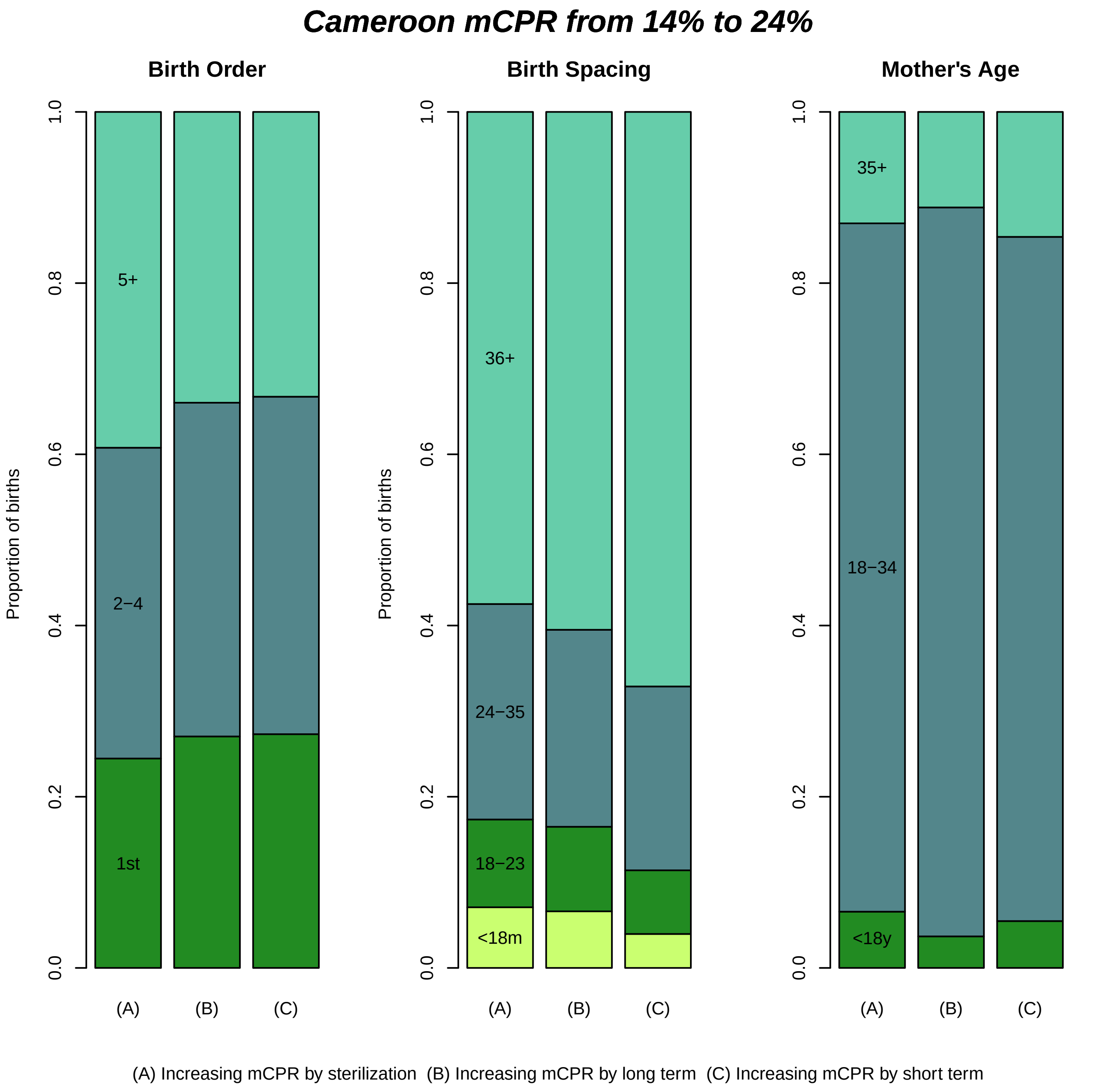
Cameroon mCPR from 14% to 24%
Birth Order
Birth Spacing
Mother's Age
Proportion of births
0.0
0.2
0.4
0.6
0.8
1.0
5+
2−4
1st
36+
24−35
18−23
<18m
35+
18−34
<18y
(A)
(B)
(C)
(A) Increasing mCPR by sterilization (B) Increasing mCPR by long term (C) Increasing mCPR by short term

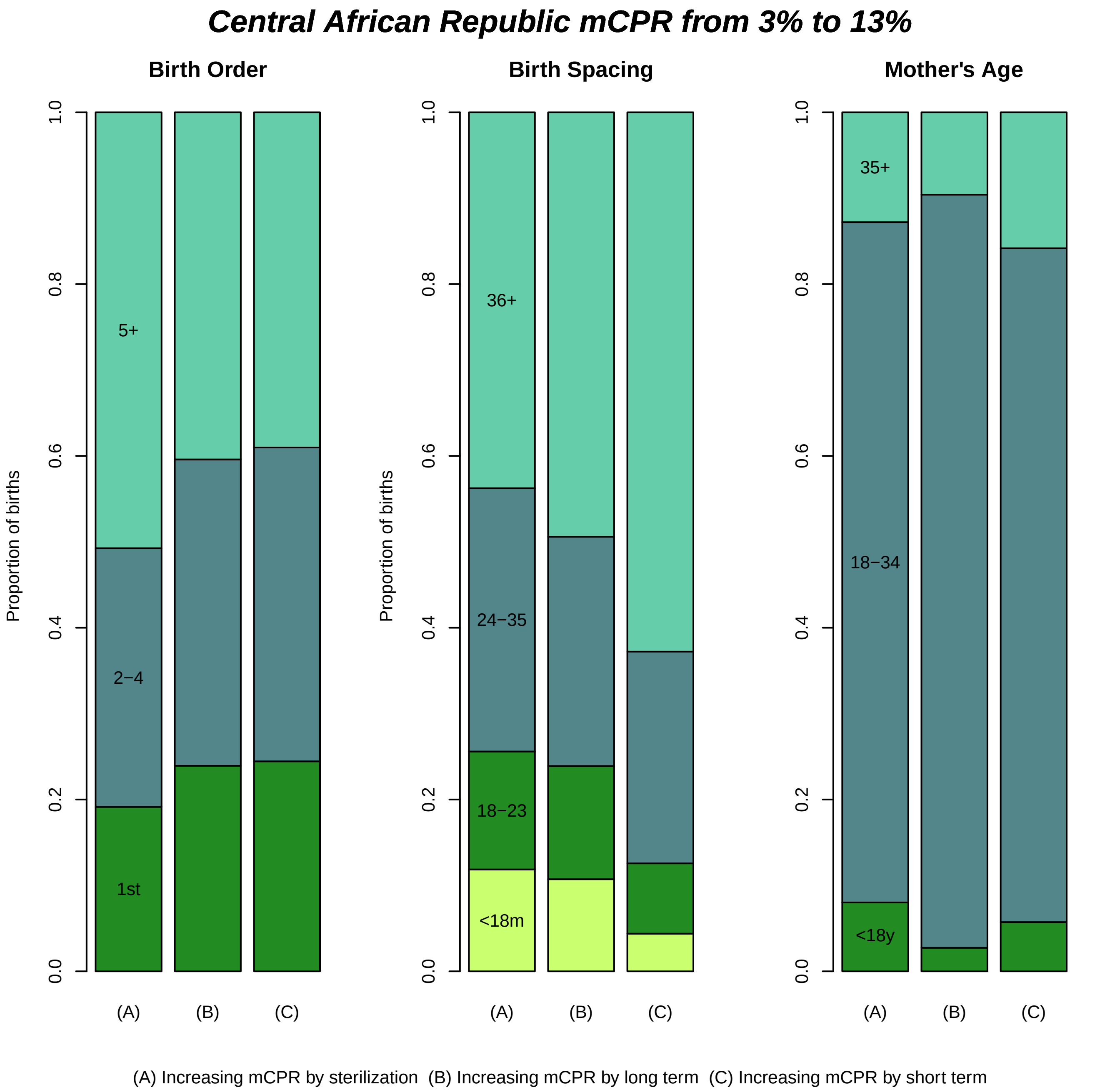
Central African Republic mCPR from 3% to 13%
Birth Order
Birth Spacing
Mother's Age
Proportion of births
0.0
0.2
0.4
0.6
0.8
1.0
5+
2−4
1st
36+
24−35
18−23
<18m
35+
18−34
<18y
(A)
(B)
(C)
(A) Increasing mCPR by sterilization (B) Increasing mCPR by long term (C) Increasing mCPR by short term

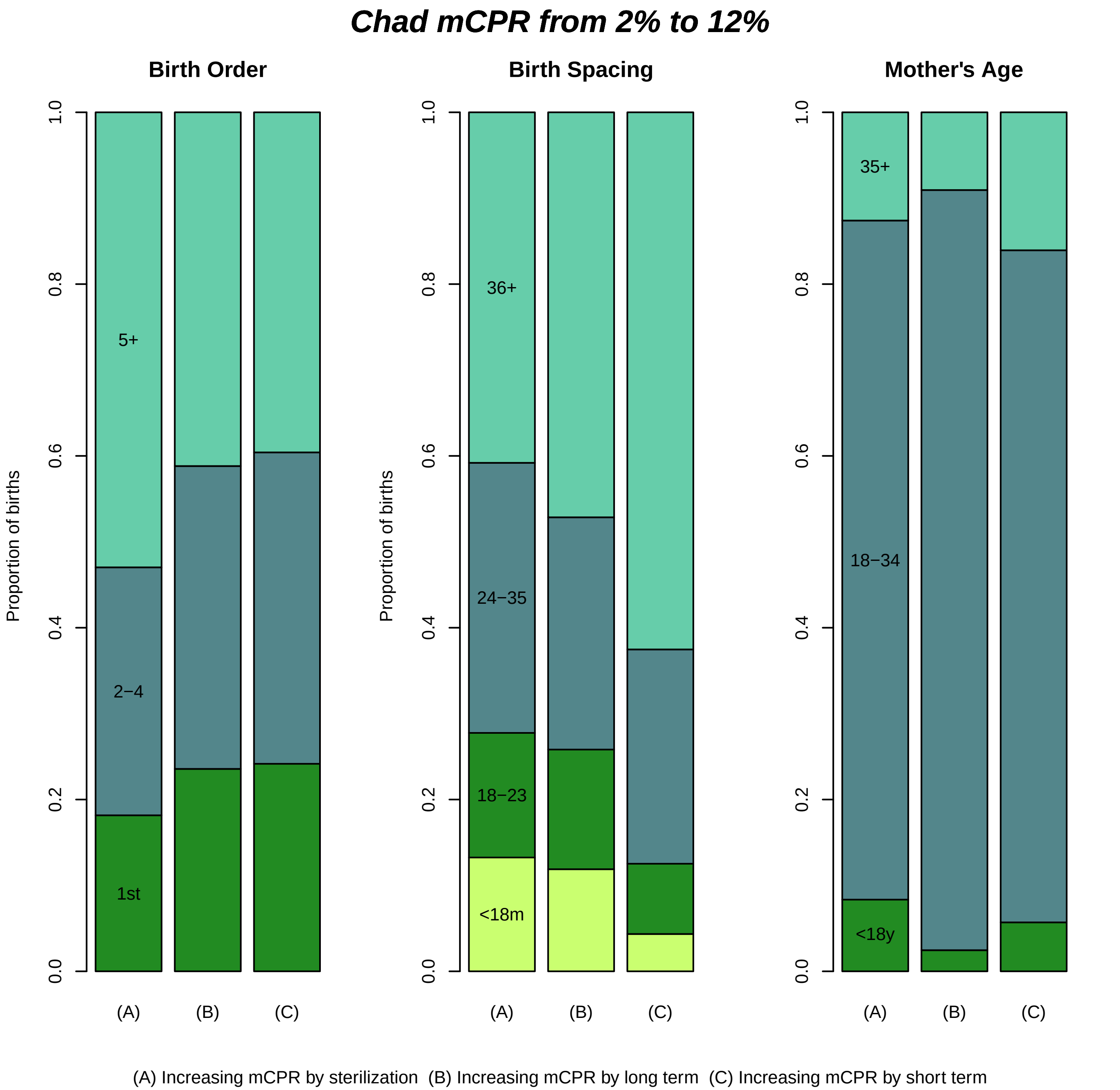

Chad mCPR from 2% to 12%
Birth Order
Birth Spacing
Mother's Age
Proportion of births
0.0
0.2
0.4
0.6
0.8
1.0
5+
2−4
1st
36+
24−35
18−23
<18m
35+
18−34
<18y
(A)
(B)
(C)
(A) Increasing mCPR by sterilization (B) Increasing mCPR by long term (C) Increasing mCPR by short term

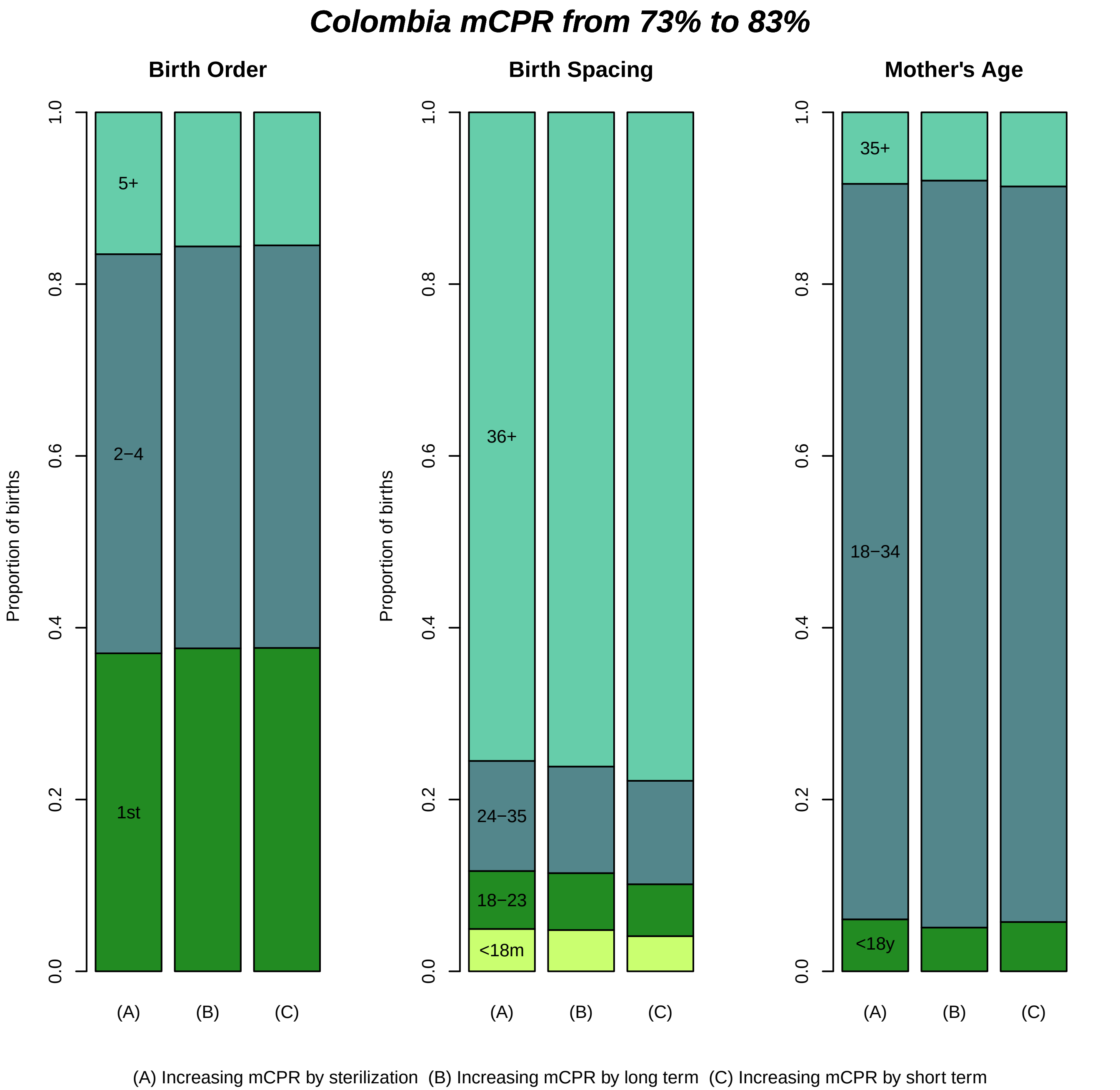

Colombia mCPR from 73% to 83%
Birth Order
Birth Spacing
Mother's Age
Proportion of births
Proportion of births
1.0
0.8
0.6
0.4
0.2
0.0
5+
2−4
1st
36+
24−35
18−23
<18m
35+
18−34
<18y
(A)
(B)
(C)
(A) Increasing mCPR by sterilization (B) Increasing mCPR by long term (C) Increasing mCPR by short term

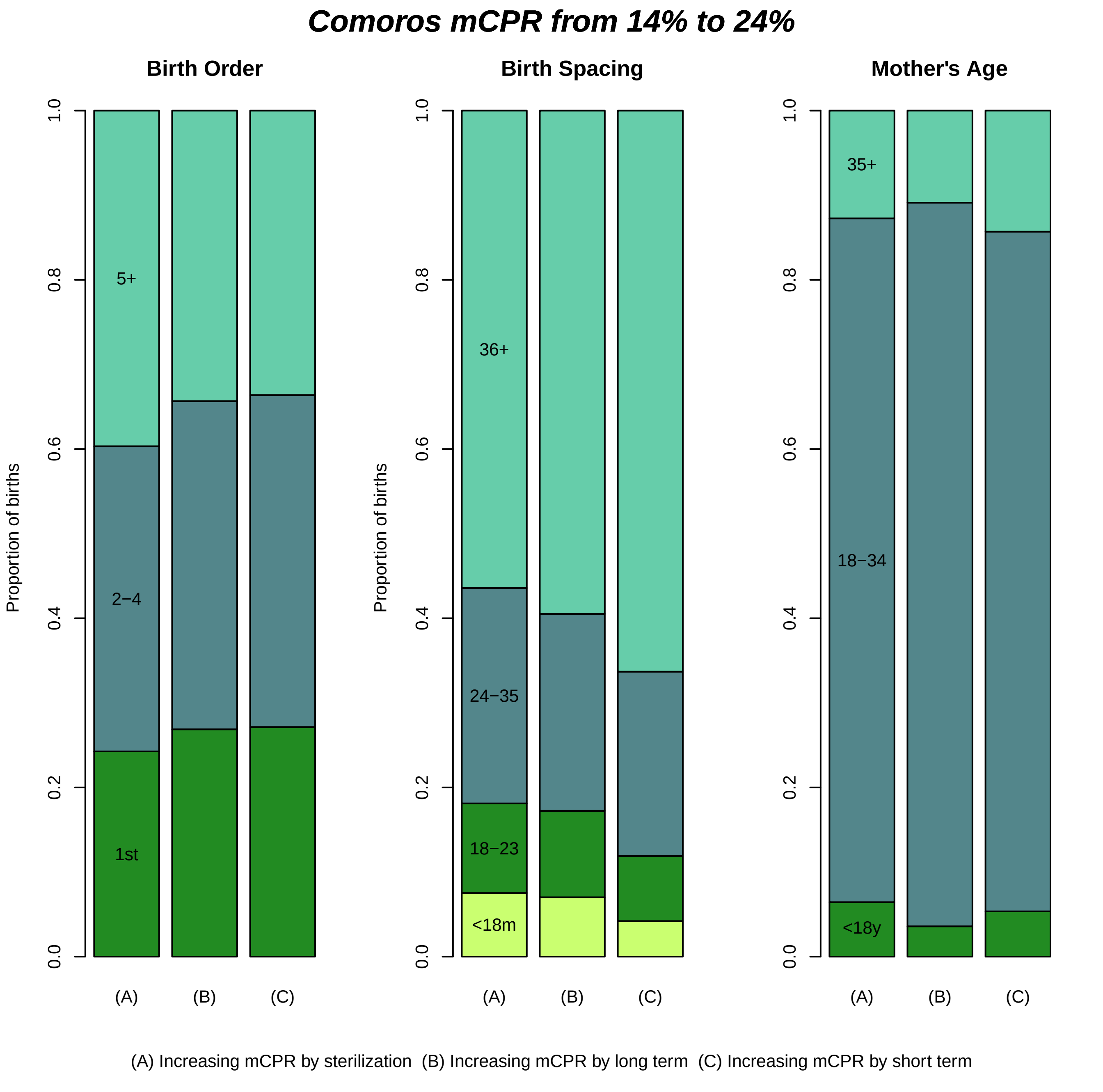

Comoros mCPR from 14% to 24%
Birth Order
Birth Spacing
Mother's Age
Proportion of births
0.0
0.2
0.4
0.6
0.8
1.0
5+
2−4
1st
36+
24−35
18−23
<18m
35+
18−34
<18y
(A)
(B)
(C)
(A) Increasing mCPR by sterilization (B) Increasing mCPR by long term (C) Increasing mCPR by short term

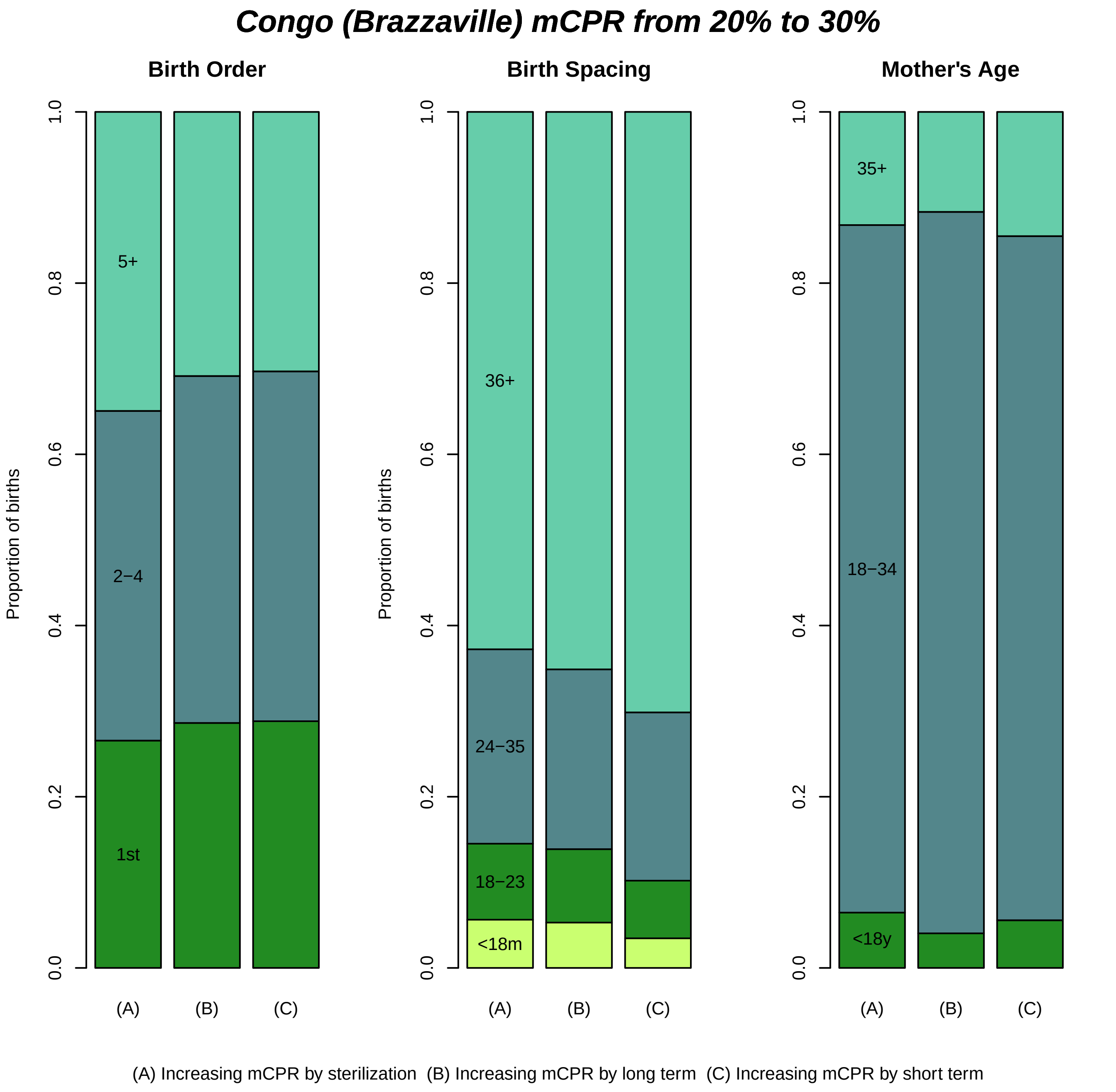

Congo (Brazzaville) mCPR from 20% to 30%
Birth Order
Birth Spacing
Mother's Age
Proportion of births
0.0
0.2
0.4
0.6
0.8
1.0
5+
2−4
1st
36+
24−35
18−23
<18m
35+
18−34
<18y
(A)
(B)
(C)
(A) Increasing mCPR by sterilization (B) Increasing mCPR by long term (C) Increasing mCPR by short term

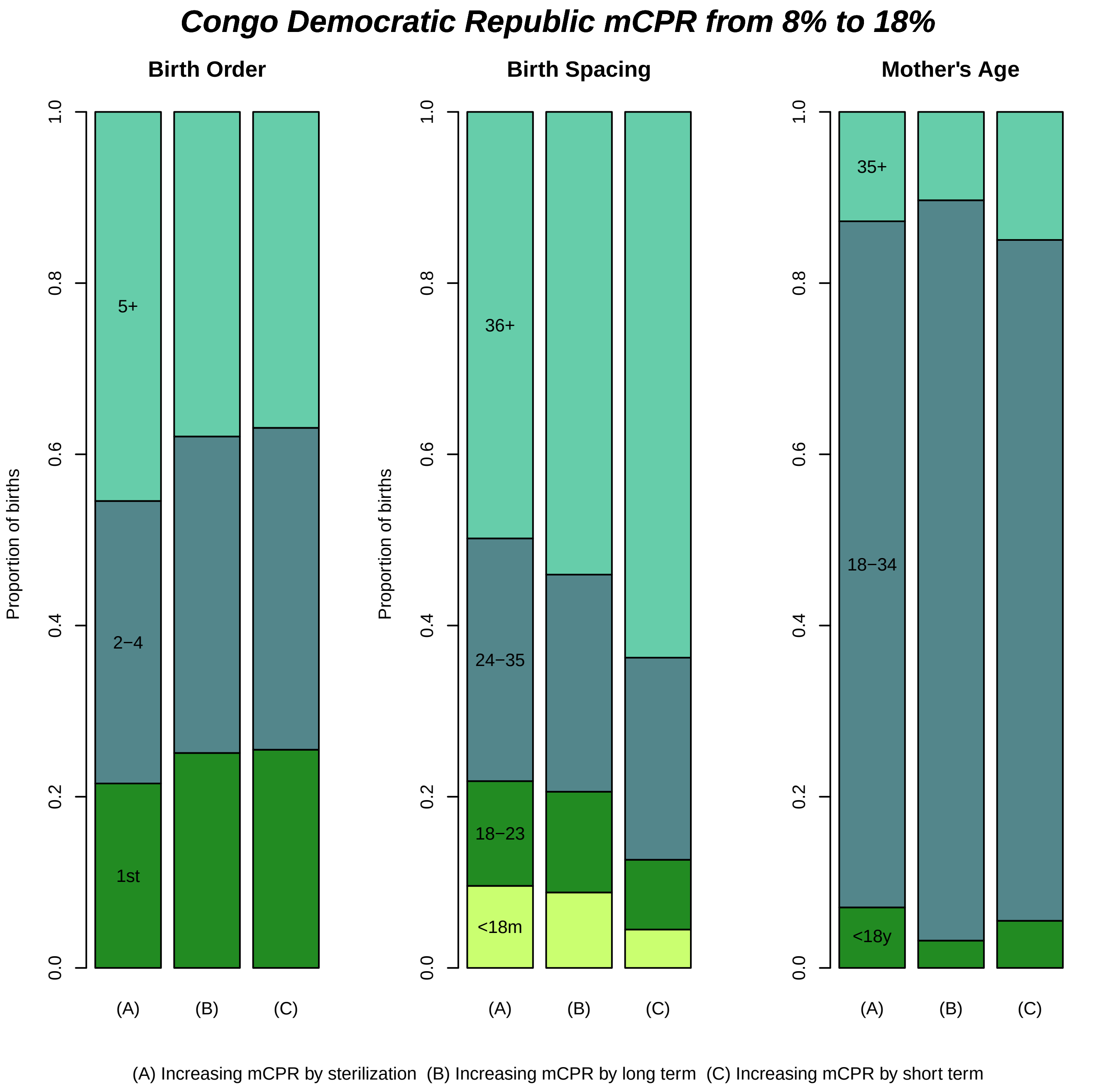
Congo Democratic Republic mCPR from 8% to 18%
Birth Order
Birth Spacing
Mother's Age
Proportion of births
0.0
0.2
0.4
0.6
0.8
1.0
5+
2−4
1st
36+
24−35
18−23
<18m
35+
18−34
<18y
(A)
(B)
(C)
(A) Increasing mCPR by sterilization (B) Increasing mCPR by long term (C) Increasing mCPR by short term

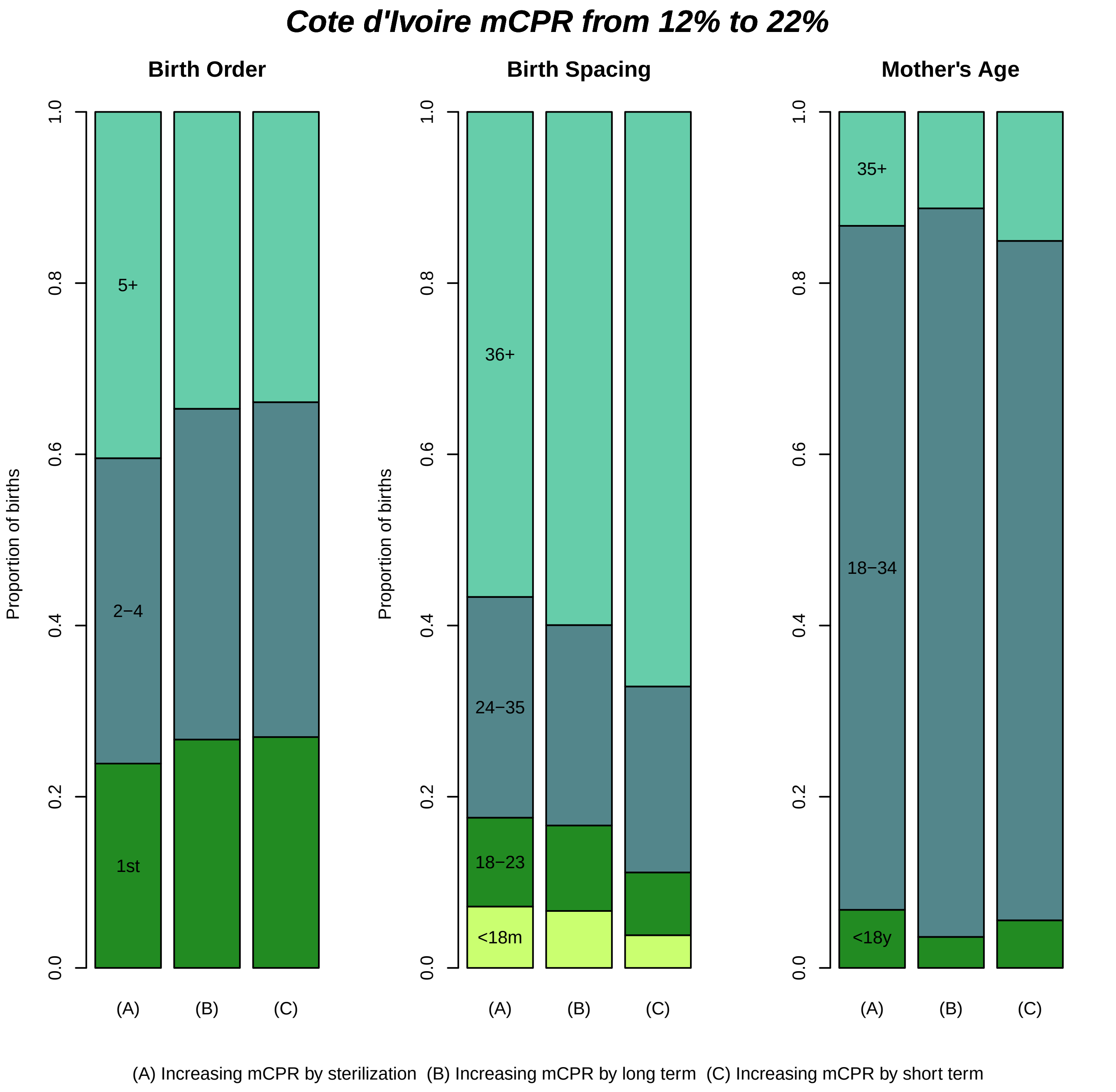

Cote d'Ivoire mCPR from 12% to 22%
Birth Order
Birth Spacing
Mother's Age
Proportion of births
0.0
0.2
0.4
0.6
0.8
1.0
5+
2−4
1st
36+
24−35
18−23
<18m
35+
18−34
<18y
(A)
(B)
(C)
(A) Increasing mCPR by sterilization (B) Increasing mCPR by long term (C) Increasing mCPR by short term

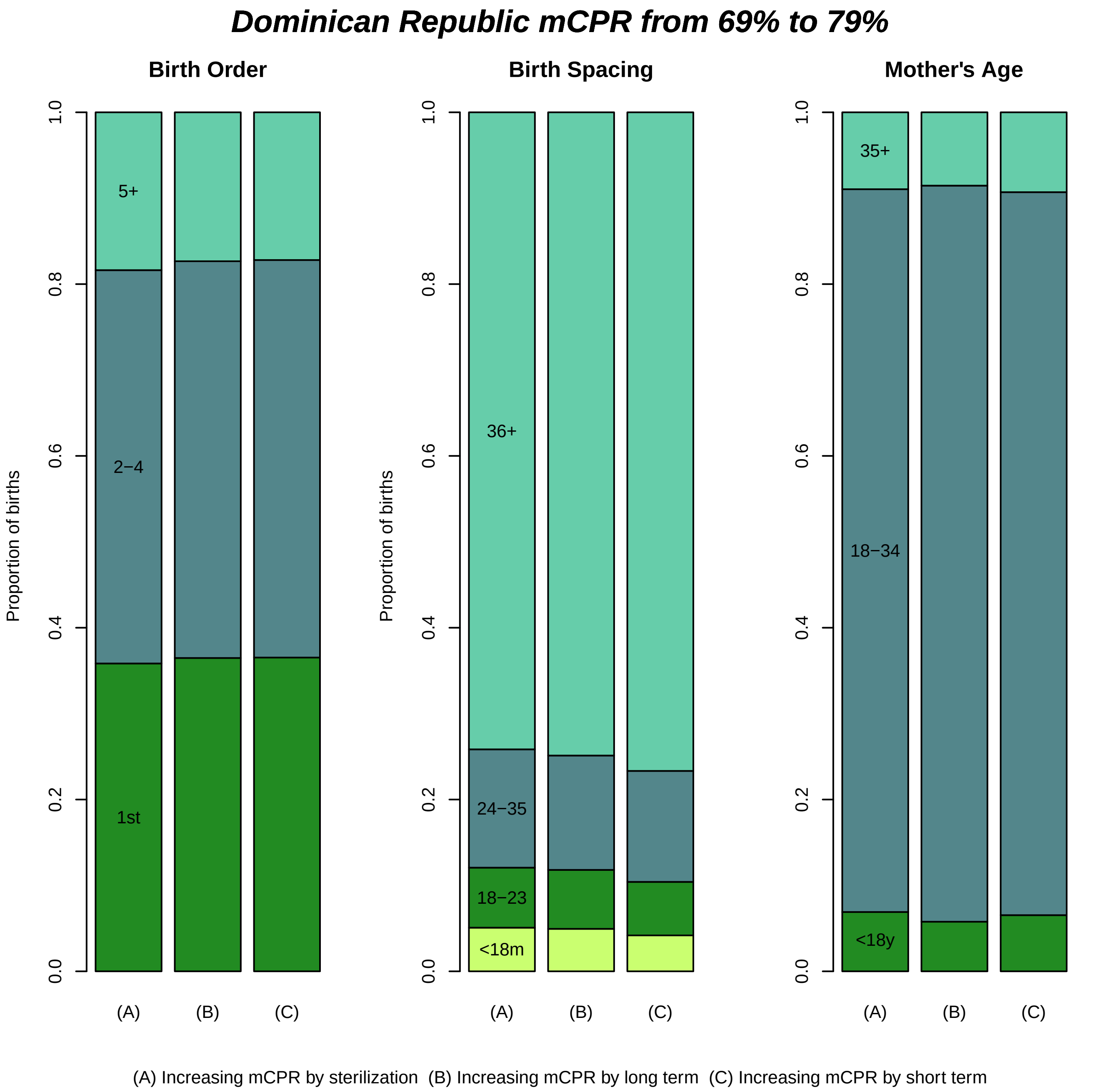
Dominican Republic mCPR from 69% to 79%
Birth Order
Birth Spacing
Mother's Age
Proportion of births
0.0
0.2
0.4
0.6
0.8
1.0
5+
2−4
1st
36+
24−35
18−23
<18m
35+
18−34
<18y
(A)
(B)
(C)
(A) Increasing mCPR by sterilization (B) Increasing mCPR by long term (C) Increasing mCPR by short term

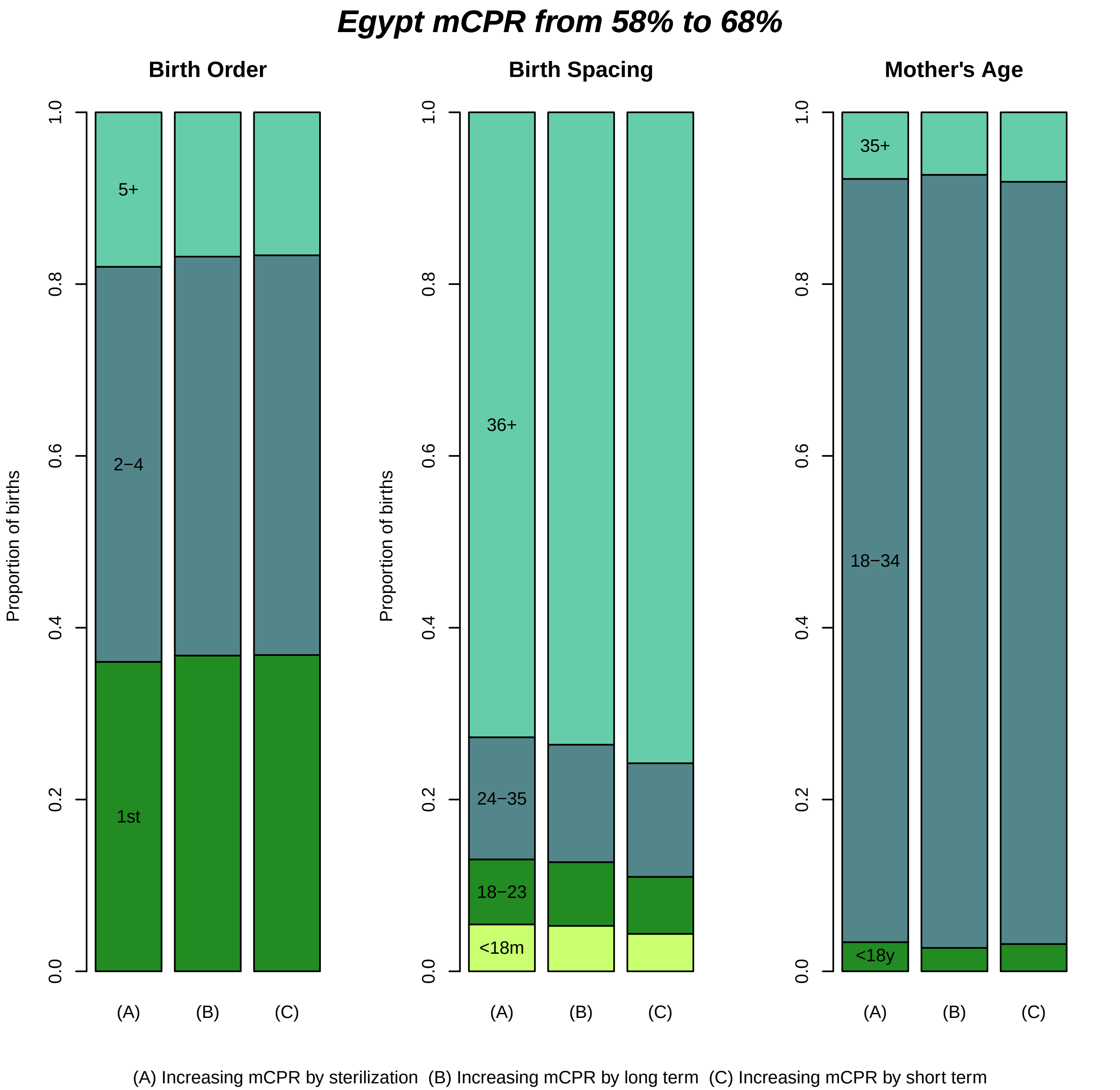
Egypt mCPR from 58% to 68%
Birth Order
Birth Spacing
Mother's Age
Proportion of births
0.0
0.2
0.4
0.6
0.8
1.0
5+
2−4
1st
36+
24−35
18−23
<18m
35+
18−34
<18y
(A)
(B)
(C)
(A) Increasing mCPR by sterilization (B) Increasing mCPR by long term (C) Increasing mCPR by short term

Ethiopia mCPR from 27% to 37%

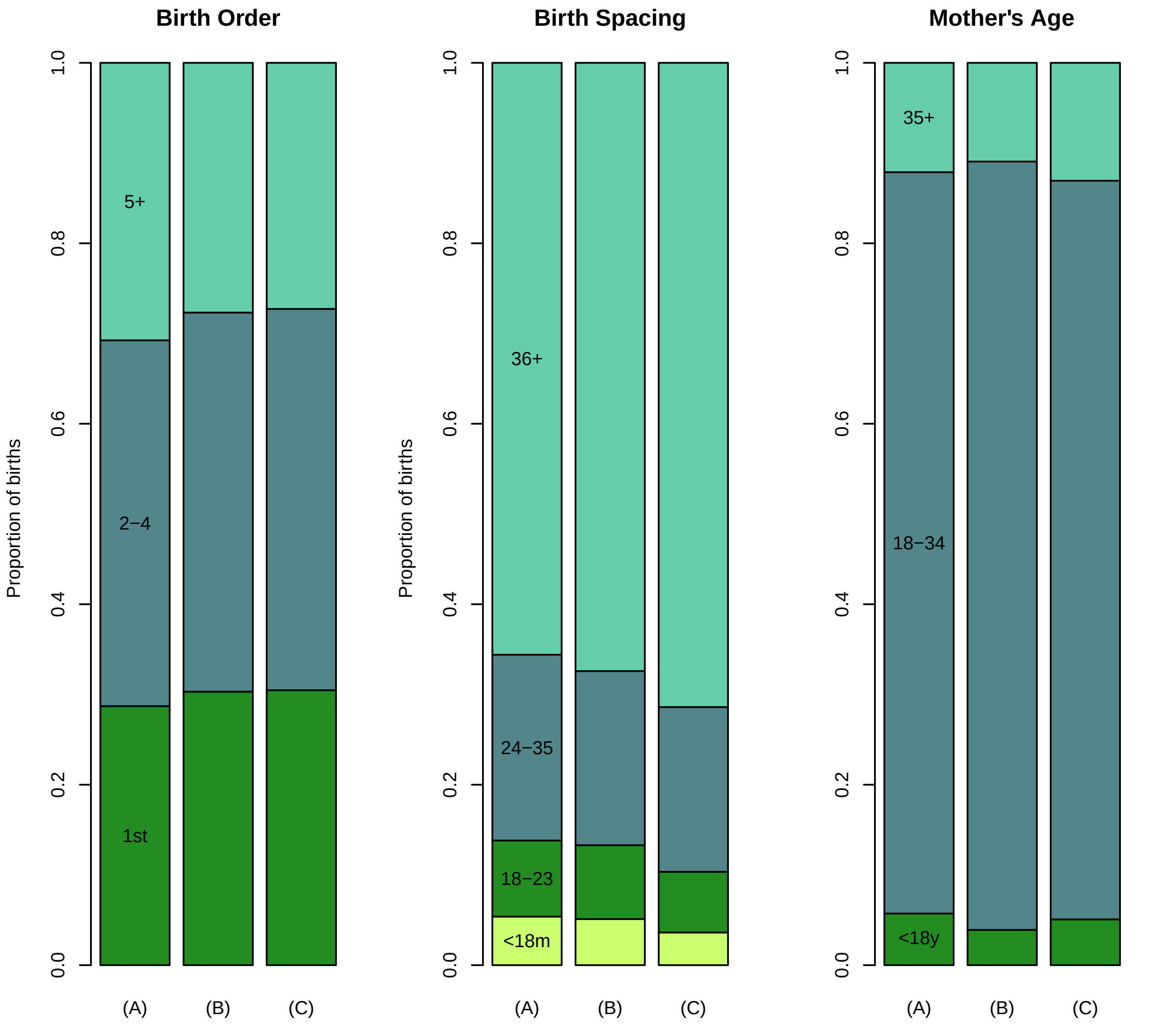

(A) Increasing mCPR by sterilization (B) Increasing mCPR by long term (C) Increasing mCPR by short term

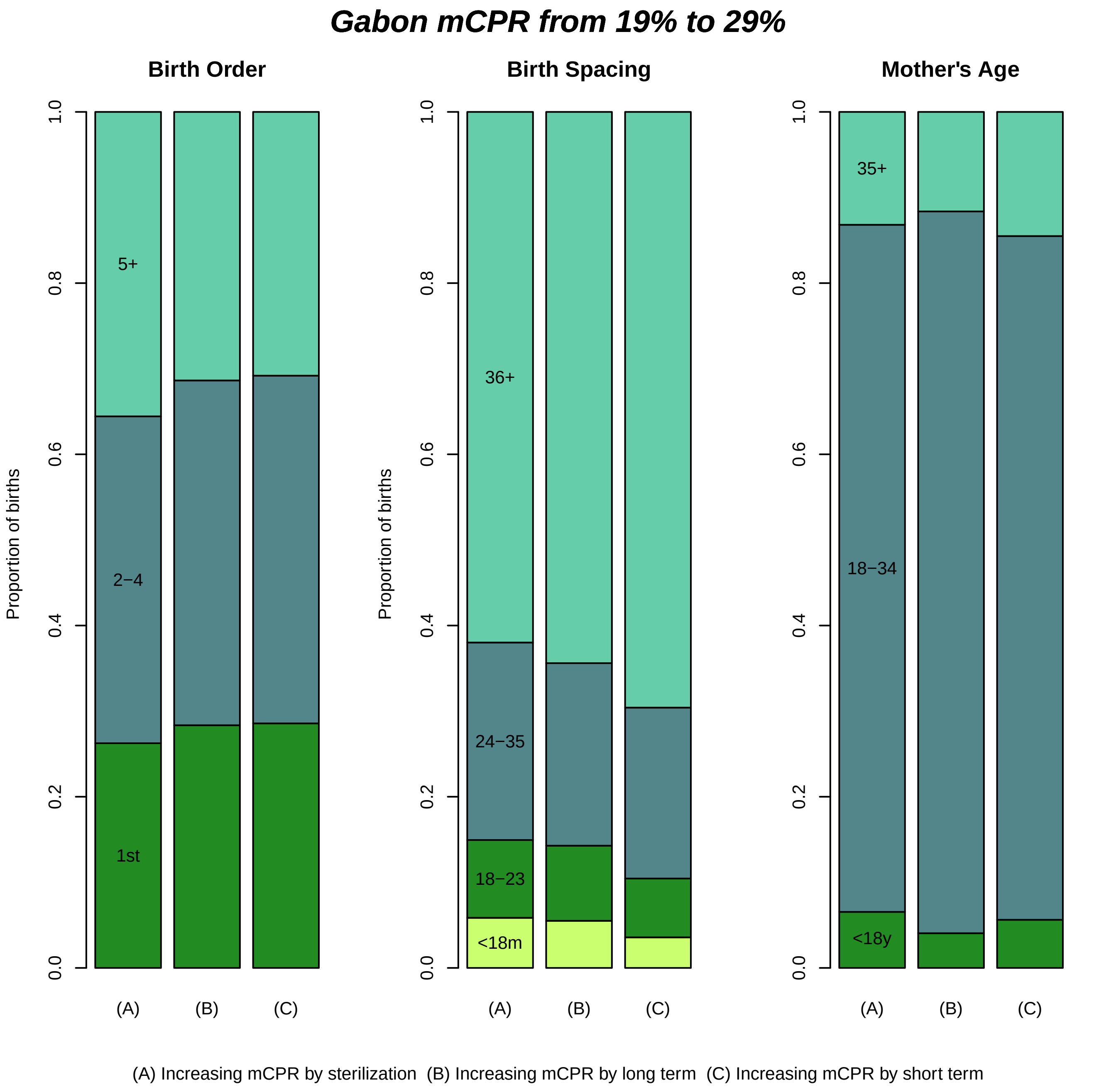

Gabon mCPR from 19% to 29%
Birth Order
Birth Spacing
Mother's Age
Proportion of births
1.0
0.8
0.6
0.4
0.2
0.0
5+
2−4
1st
36+
24−35
18−23
<18m
35+
18−34
<18y
(A)
(B)
(C)
(A) Increasing mCPR by sterilization (B) Increasing mCPR by long term (C) Increasing mCPR by short term

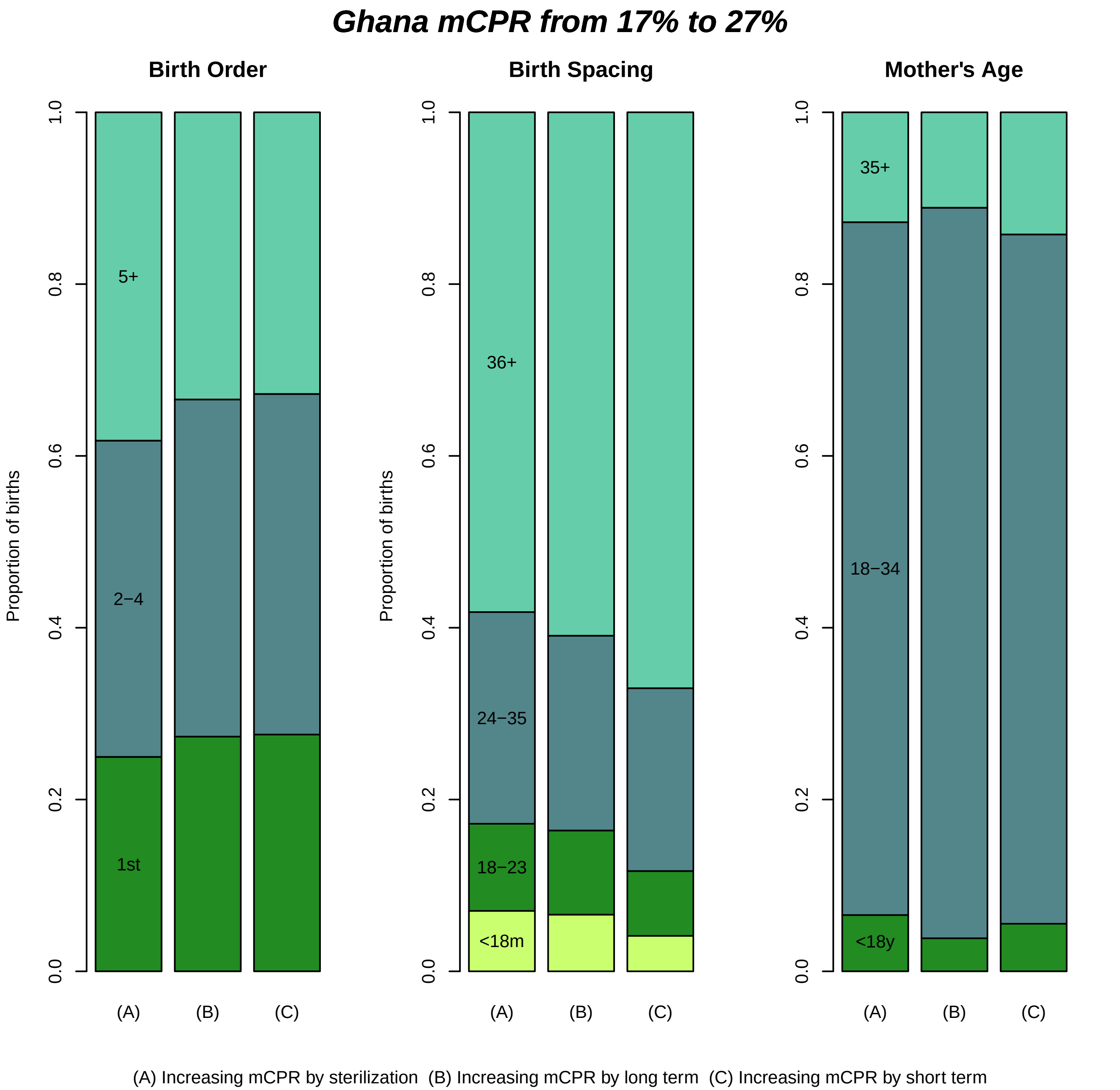
Ghana mCPR from 17% to 27%
Birth Order
Birth Spacing
Mother's Age
Proportion of births
Proportion of births
0.0
0.2
0.4
0.6
0.8
1.0
5+
2−4
1st
36+
24−35
18−23
<18m
35+
18−34
<18y
(A)
(B)
(C)
(A) Increasing mCPR by sterilization (B) Increasing mCPR by long term (C) Increasing mCPR by short term

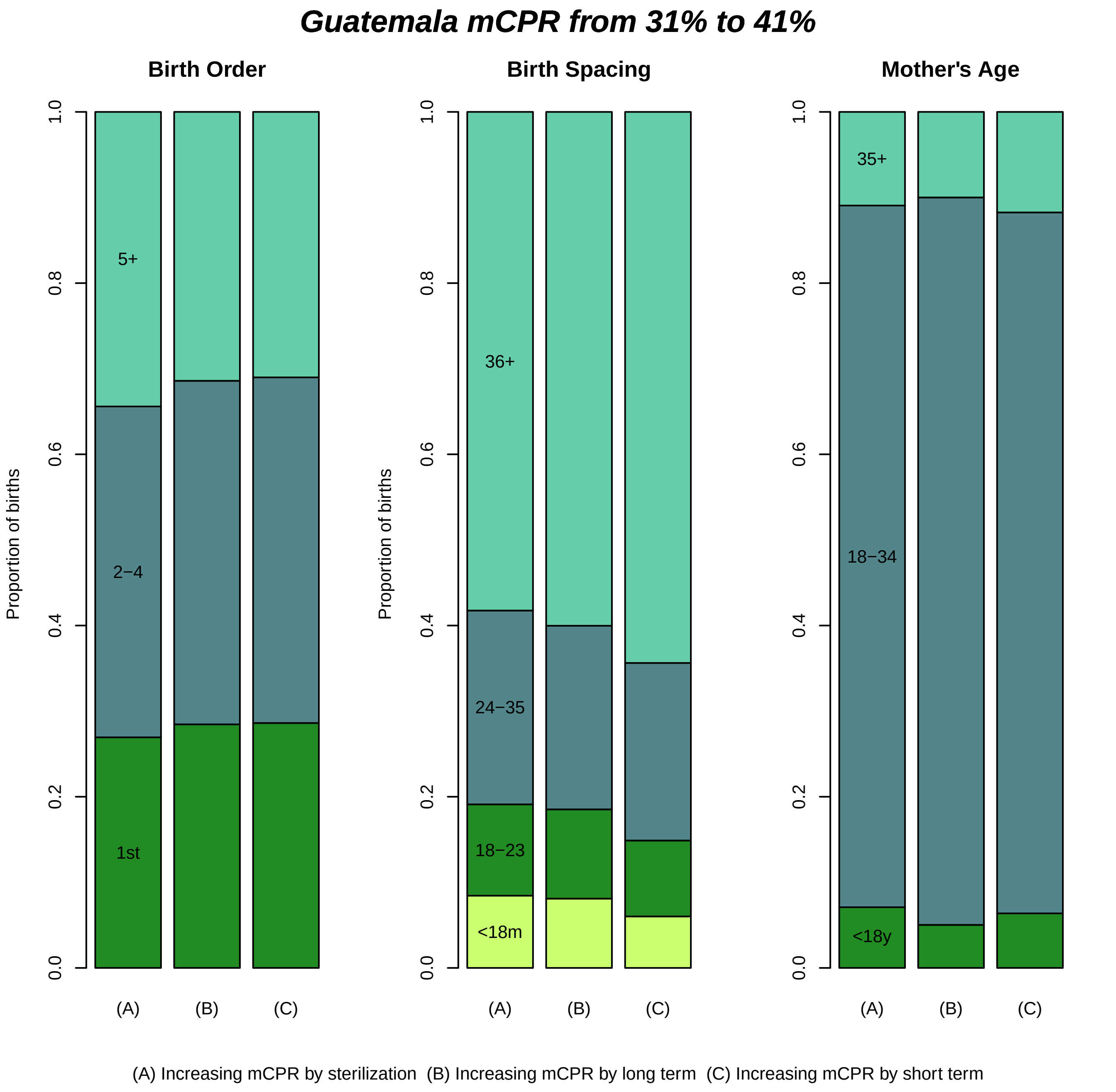
Guatemala mCPR from 31% to 41%
Birth Order
Birth Spacing
Mother's Age
Proportion of births
0.0
0.2
0.4
0.6
0.8
1.0
5+
2−4
1st
36+
24−35
18−23
<18m
35+
18−34
<18y
(A)
(B)
(C)
(A) Increasing mCPR by sterilization (B) Increasing mCPR by long term (C) Increasing mCPR by short term

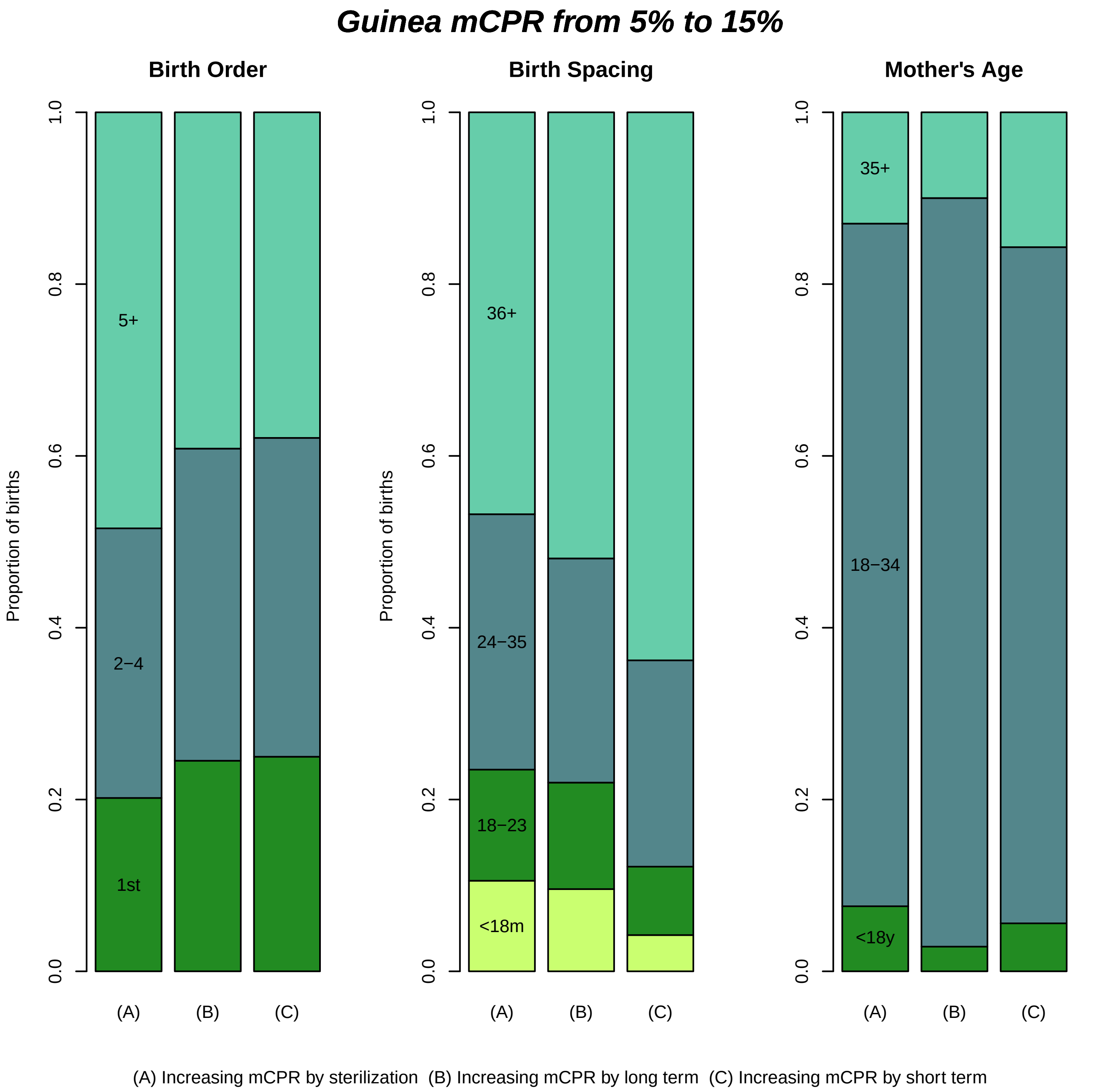

Guinea mCPR from 5% to 15%
Birth Order
Birth Spacing
Mother's Age
Proportion of births
0.0
0.2
0.4
0.6
0.8
1.0
5+
2−4
1st
36+
24−35
18−23
<18m
35+
18−34
<18y
(A)
(B)
(C)
(A) Increasing mCPR by sterilization (B) Increasing mCPR by long term (C) Increasing mCPR by short term

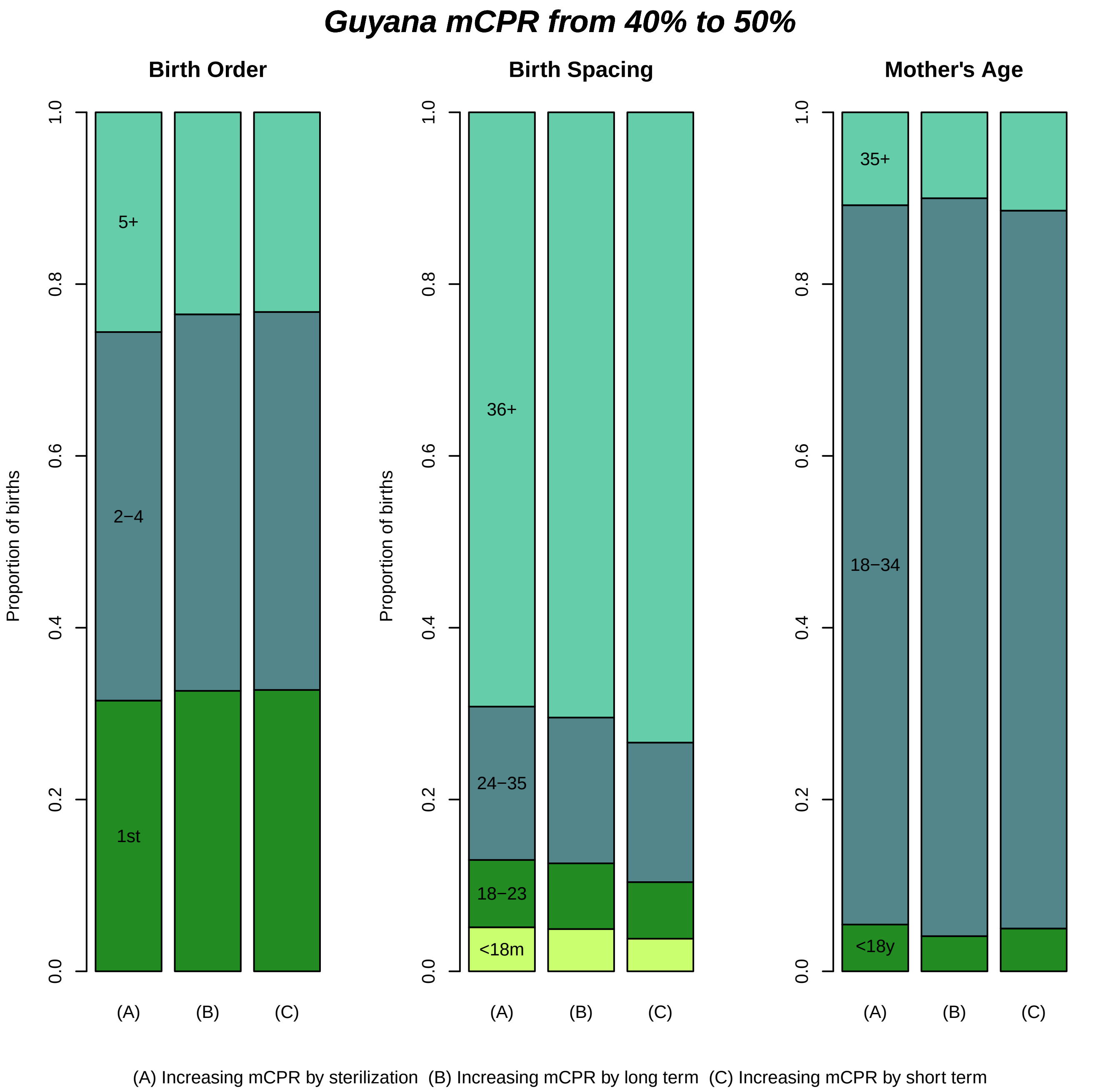

Guyana mCPR from 40% to 50%
Birth Order
Birth Spacing
Mother's Age
Proportion of births
0.0
0.2
0.4
0.6
0.8
1.0
5+
2−4
1st
36+
24−35
18−23
<18m
35+
18−34
<18y
(A)
(B)
(C)
(A) Increasing mCPR by sterilization (B) Increasing mCPR by long term (C) Increasing mCPR by short term

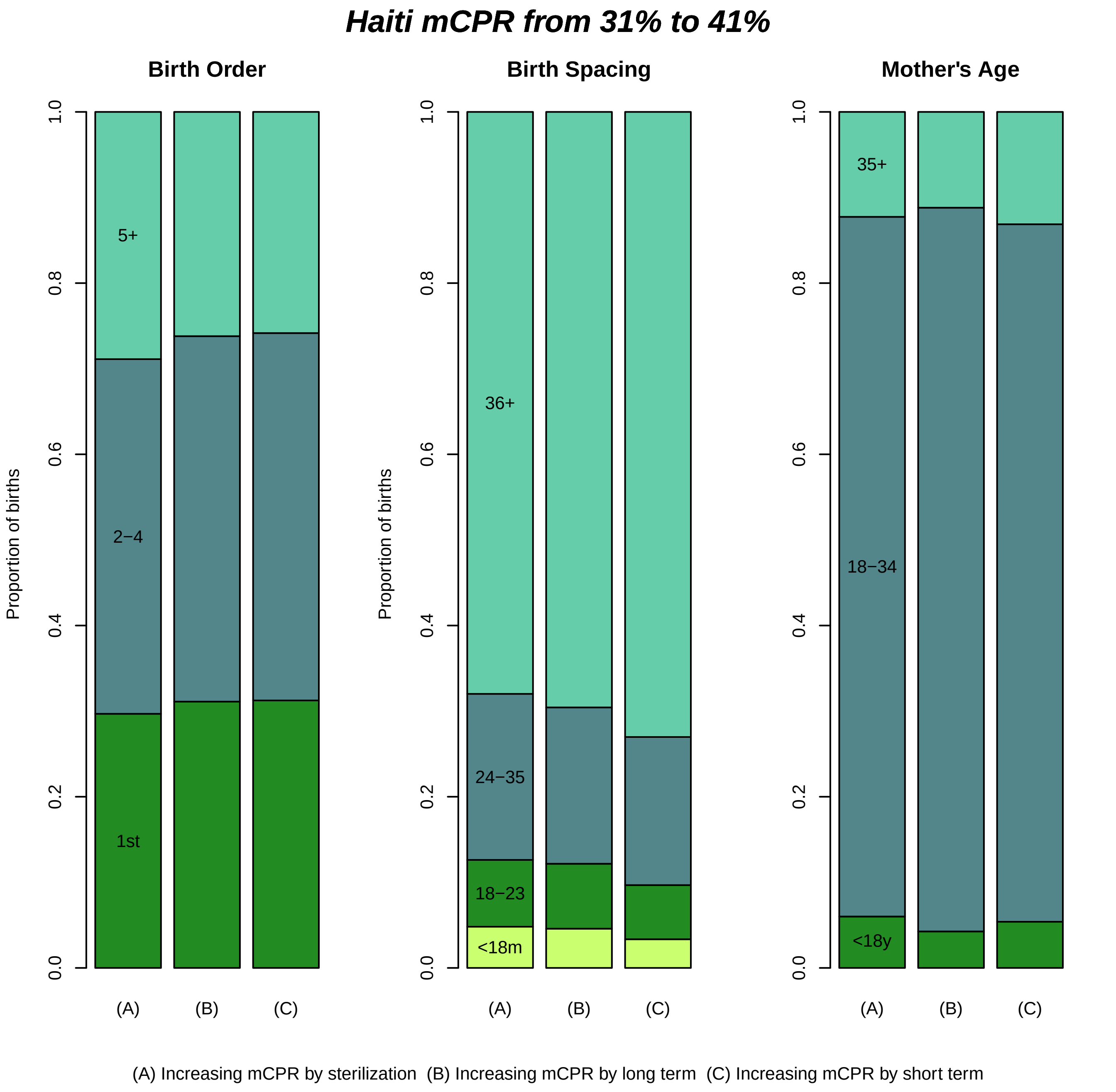

Haiti mCPR from 31% to 41%
Birth Order
Birth Spacing
Mother's Age
Proportion of births
0.0
0.2
0.4
0.6
0.8
1.0
5+
2−4
1st
36+
24−35
18−23
<18m
35+
18−34
<18y
(A)
(B)
(C)
(A) Increasing mCPR by sterilization (B) Increasing mCPR by long term (C) Increasing mCPR by short term

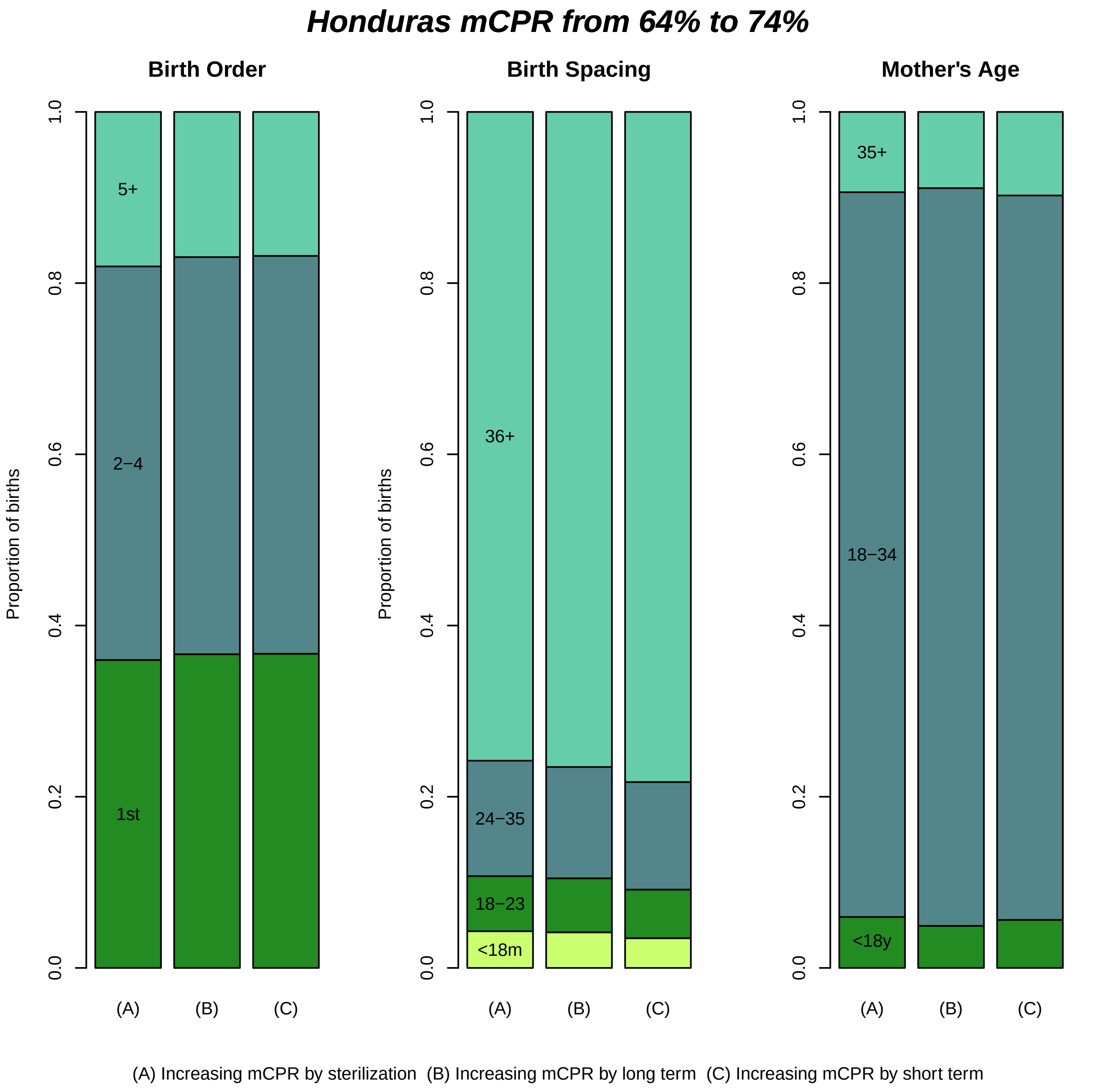

Honduras mCPR from 64% to 74%
Birth Order
Birth Spacing
Mother's Age
Proportion of births
Proportion of births
0.0
0.2
0.4
0.6
0.8
1.0
5+
2−4
1st
36+
24−35
18−23
<18m
35+
18−34
<18y
(A)
(B)
(C)
(A) Increasing mCPR by sterilization (B) Increasing mCPR by long term (C) Increasing mCPR by short term

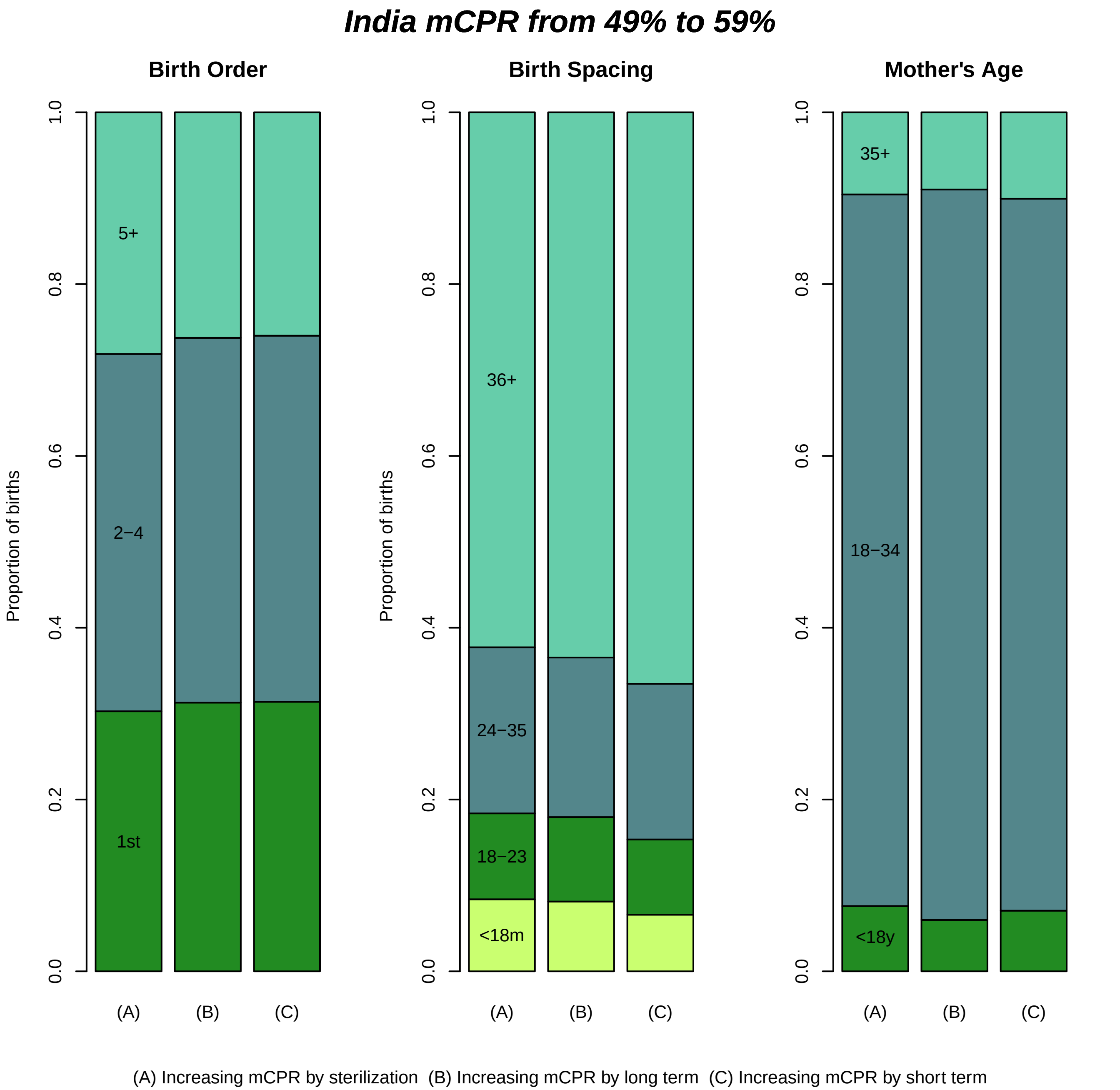
India mCPR from 49% to 59%
Birth Order
Birth Spacing
Mother's Age
Proportion of births
Proportion of births
1.0
0.8
0.6
0.4
0.2
0.0
5+
2−4
1st
36+
24−35
18−23
<18m
35+
18−34
<18y
(A)
(B)
(C)
(A) Increasing mCPR by sterilization (B) Increasing mCPR by long term (C) Increasing mCPR by short term

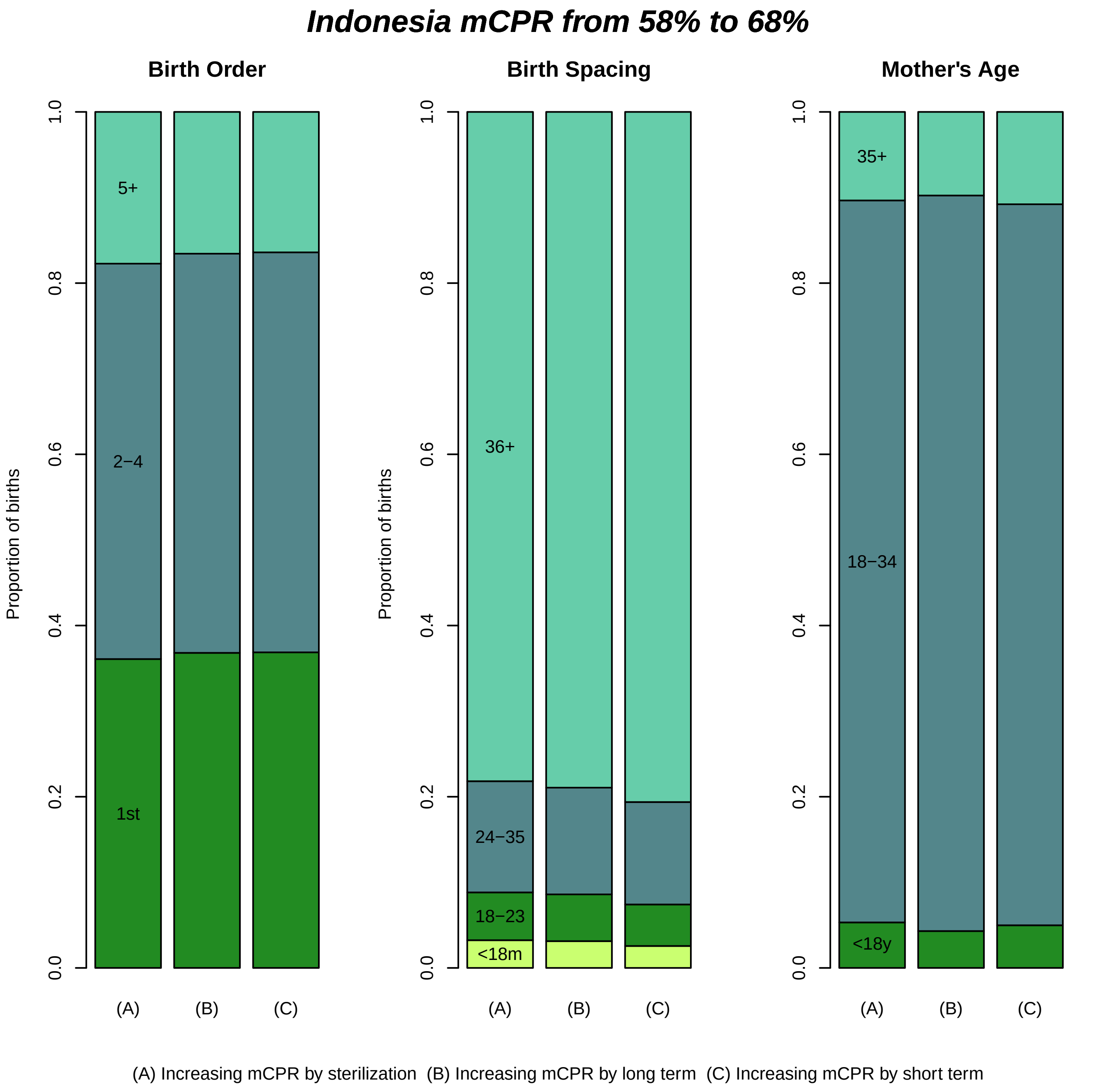
Indonesia mCPR from 58% to 68%
Birth Order
Birth Spacing
Mother's Age
Proportion of births
0.0
0.2
0.4
0.6
0.8
1.0
5+
2−4
1st
36+
24−35
18−23
<18m
35+
18−34
<18y
(A)
(B)
(C)
(A) Increasing mCPR by sterilization (B) Increasing mCPR by long term (C) Increasing mCPR by short term

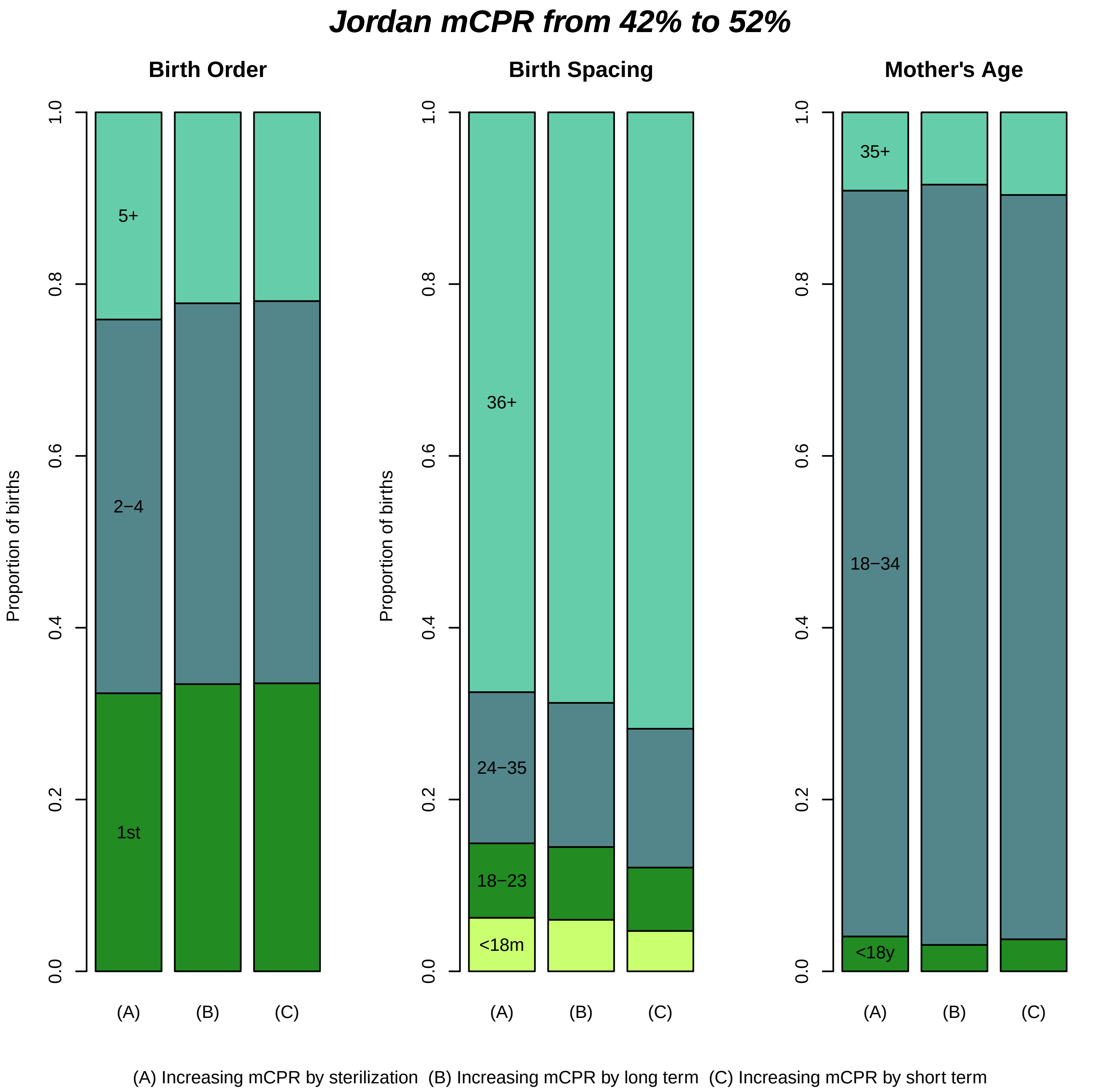

Jordan mCPR from 42% to 52%
Birth Order
Birth Spacing
Mother's Age
Proportion of births
0.0
0.2
0.4
0.6
0.8
1.0
5+
2−4
1st
36+
24−35
18−23
<18m
35+
18−34
<18y
(A)
(B)
(C)
(A) Increasing mCPR by sterilization (B) Increasing mCPR by long term (C) Increasing mCPR by short term

Kazakhstan mCPR from 53% to 63%

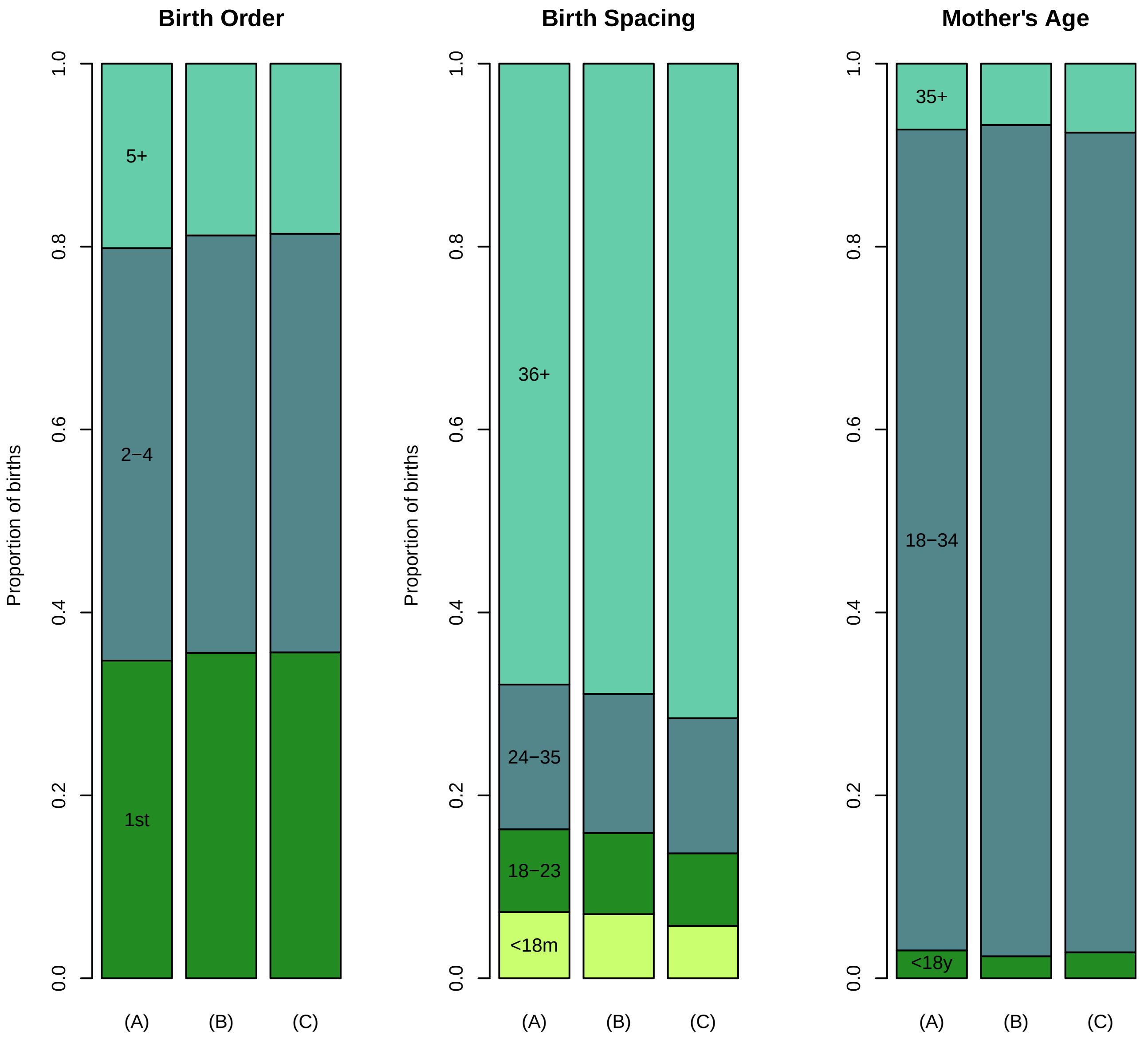

(A) Increasing mCPR by sterilization (B) Increasing mCPR by long term (C) Increasing mCPR by short term

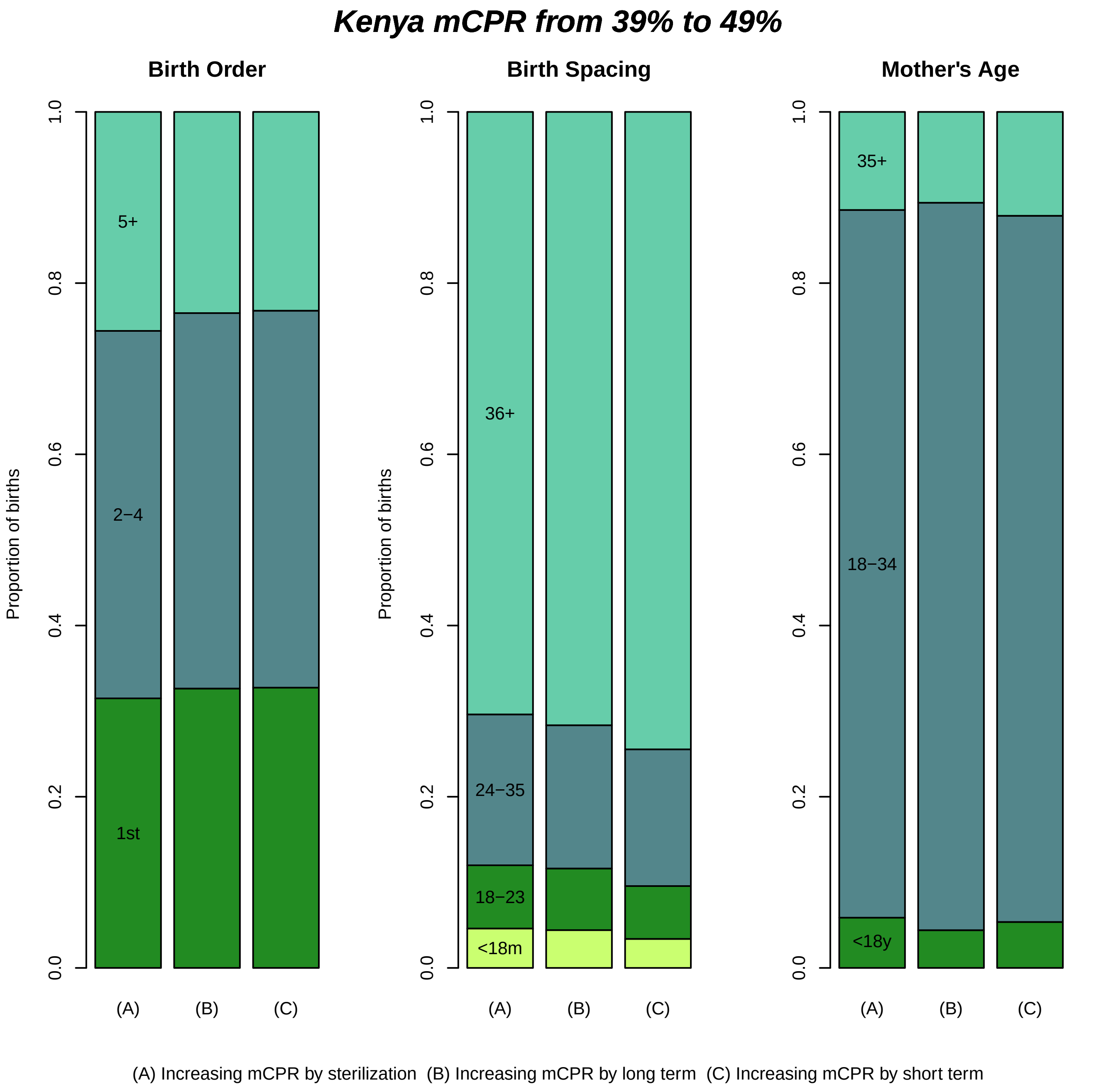

Kenya mCPR from 39% to 49%
Birth Order
Birth Spacing
Mother's Age
Proportion of births
0.0
0.2
0.4
0.6
0.8
1.0
5+
2−4
1st
36+
24−35
18−23
<18m
35+
18−34
<18y
(A)
(B)
(C)
(A) Increasing mCPR by sterilization (B) Increasing mCPR by long term (C) Increasing mCPR by short term

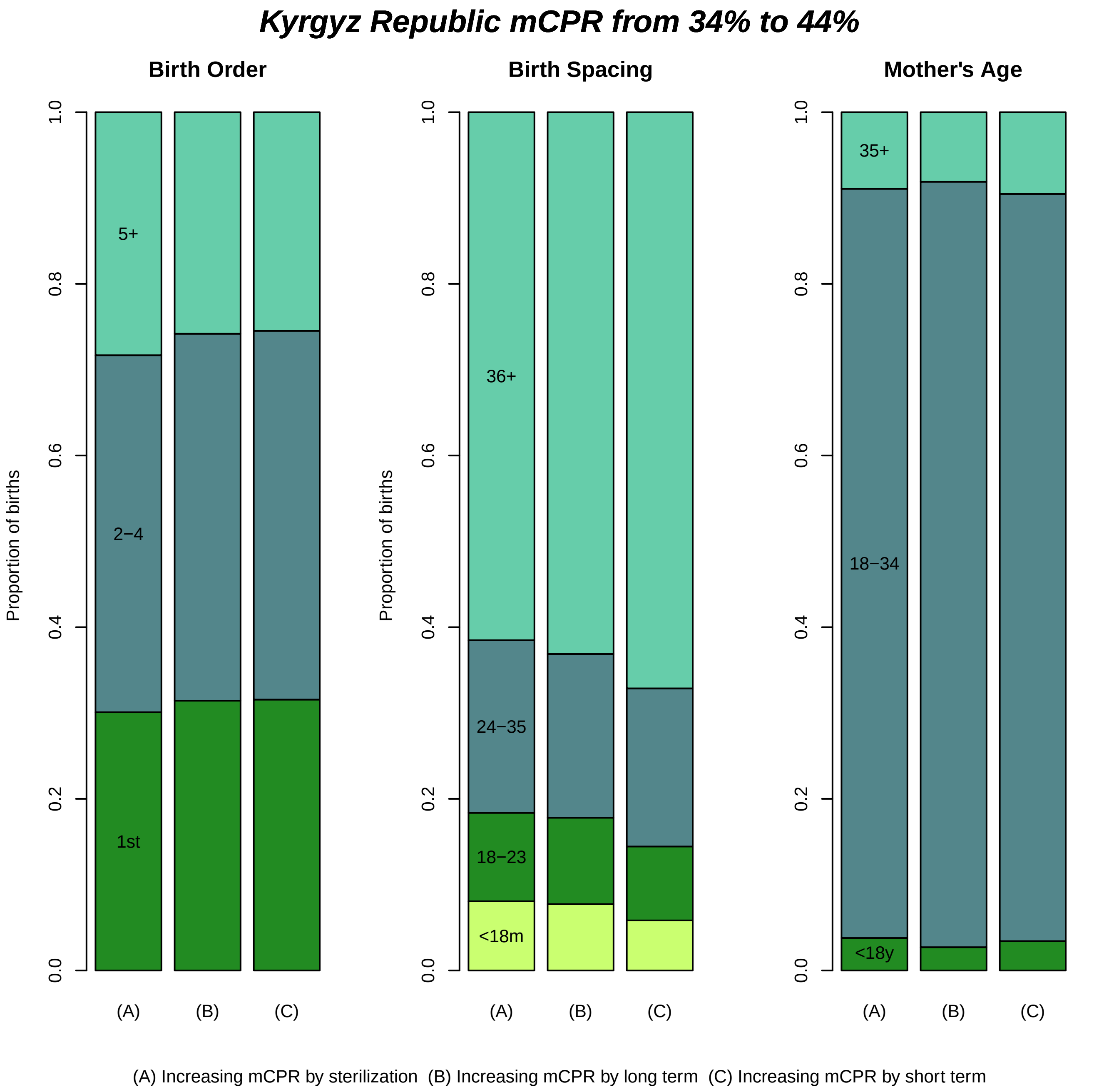
Kyrgyz Republic mCPR from 34% to 44%
Birth Order
Birth Spacing
Mother's Age
Proportion of births
0.0
0.2
0.4
0.6
0.8
1.0
5+
2−4
1st
36+
24−35
18−23
<18m
35+
18−34
<18y
(A)
(B)
(C)
(A) Increasing mCPR by sterilization (B) Increasing mCPR by long term (C) Increasing mCPR by short term

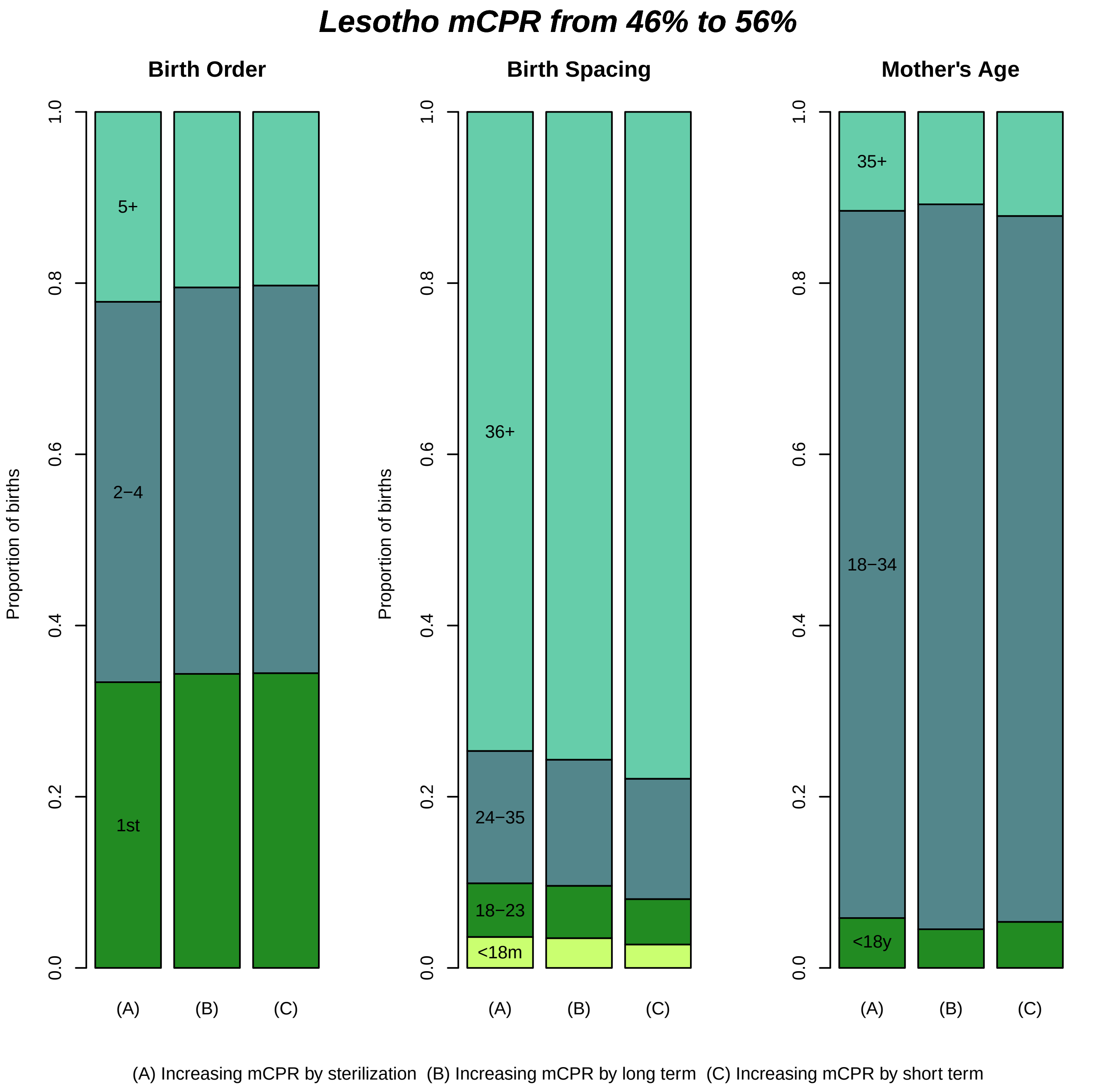

Lesotho mCPR from 46% to 56%
Birth Order
Birth Spacing
Mother's Age
Proportion of births
0.0
0.2
0.4
0.6
0.8
1.0
5+
2−4
1st
36+
24−35
18−23
<18m
35+
18−34
<18y
(A)
(B)
(C)
(A) Increasing mCPR by sterilization (B) Increasing mCPR by long term (C) Increasing mCPR by short term

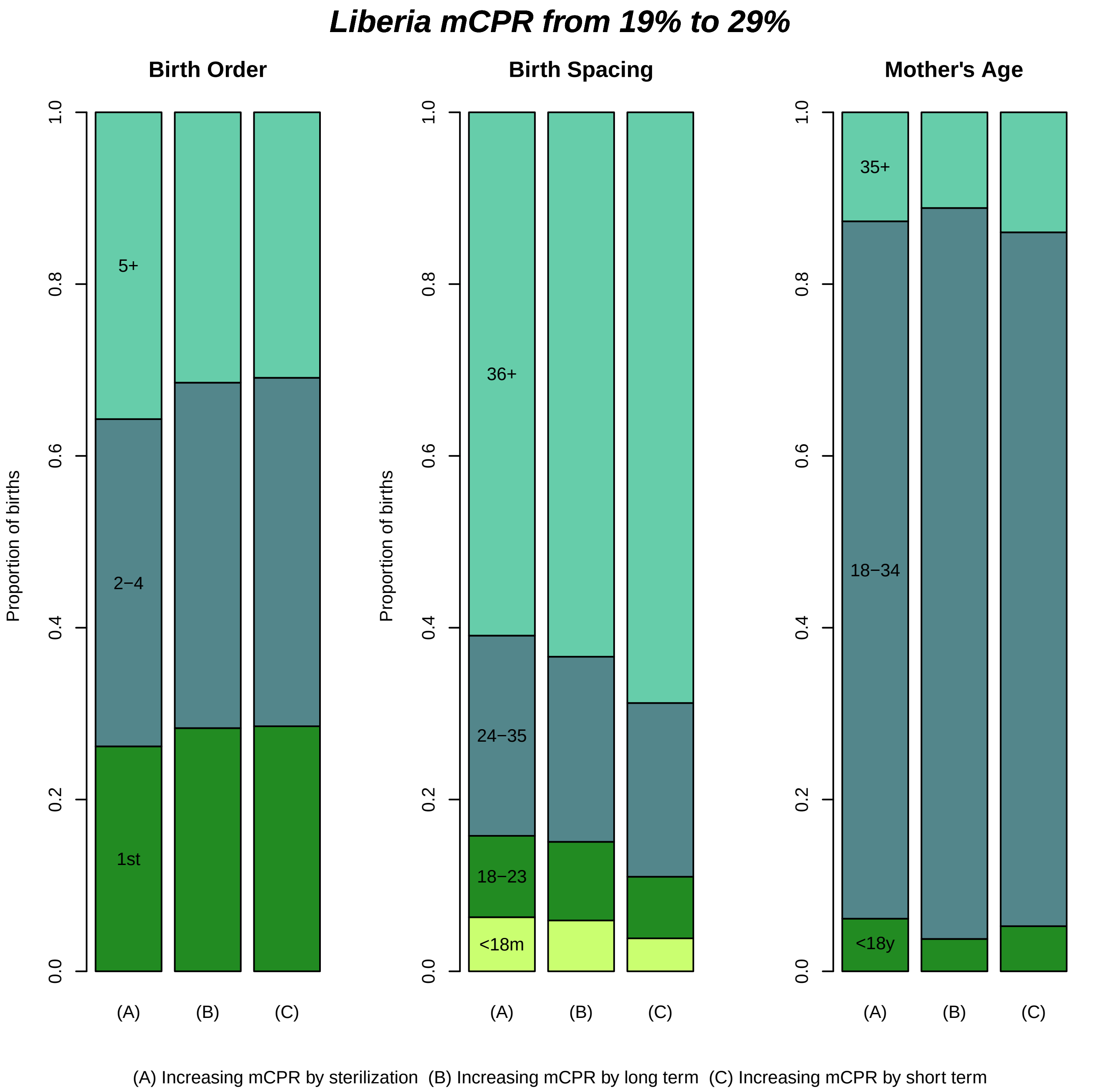
Liberia mCPR from 19% to 29%
Birth Order
Birth Spacing
Mother's Age
Proportion of births
0.0
0.2
0.4
0.6
0.8
1.0
5+
2−4
1st
36+
24−35
18−23
<18m
35+
18−34
<18y
(A)
(B)
(C)
(A) Increasing mCPR by sterilization (B) Increasing mCPR by long term (C) Increasing mCPR by short term

Madagascar mCPR from 29% to 39%

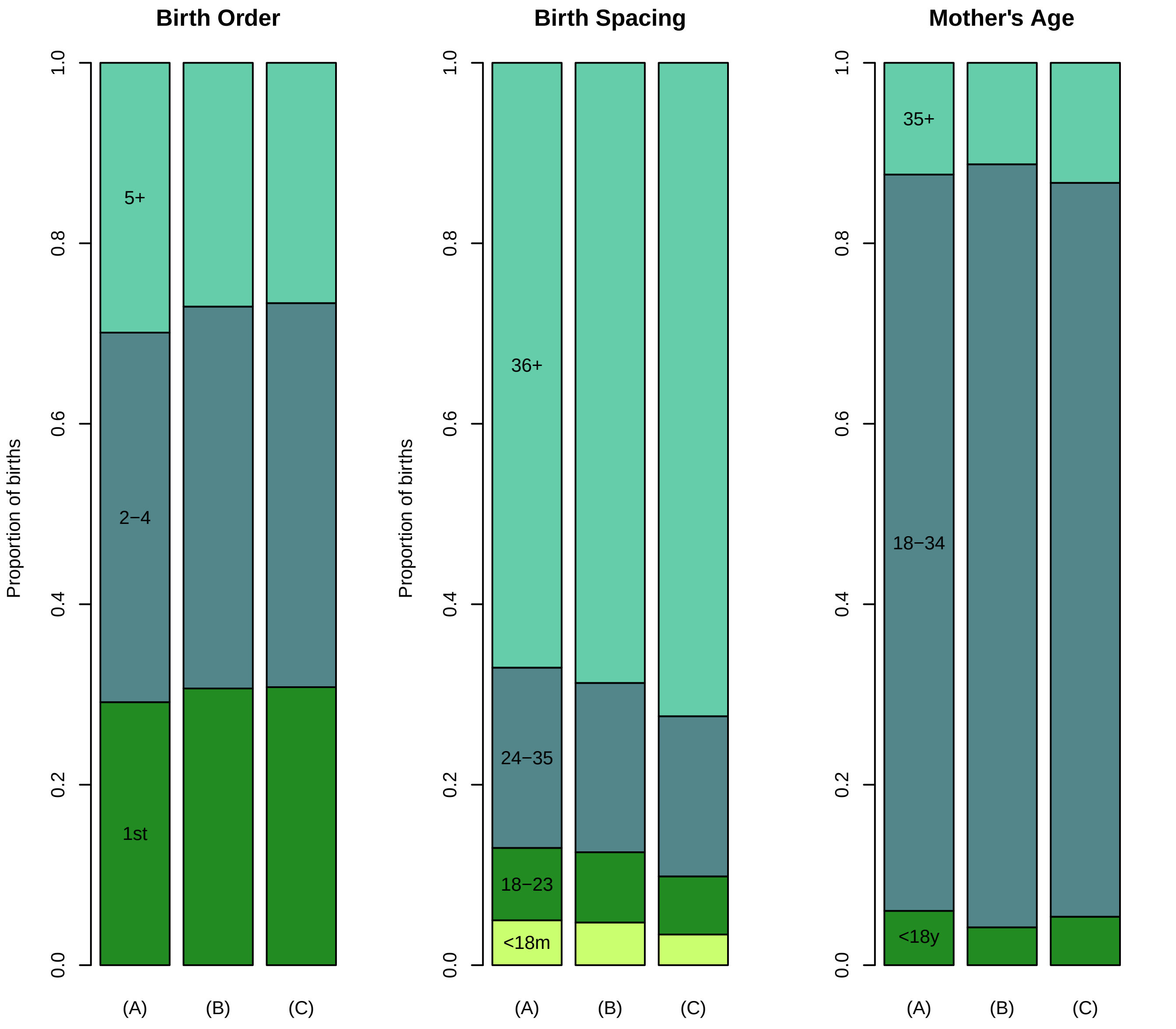

(A) Increasing mCPR by sterilization (B) Increasing mCPR by long term (C) Increasing mCPR by short term

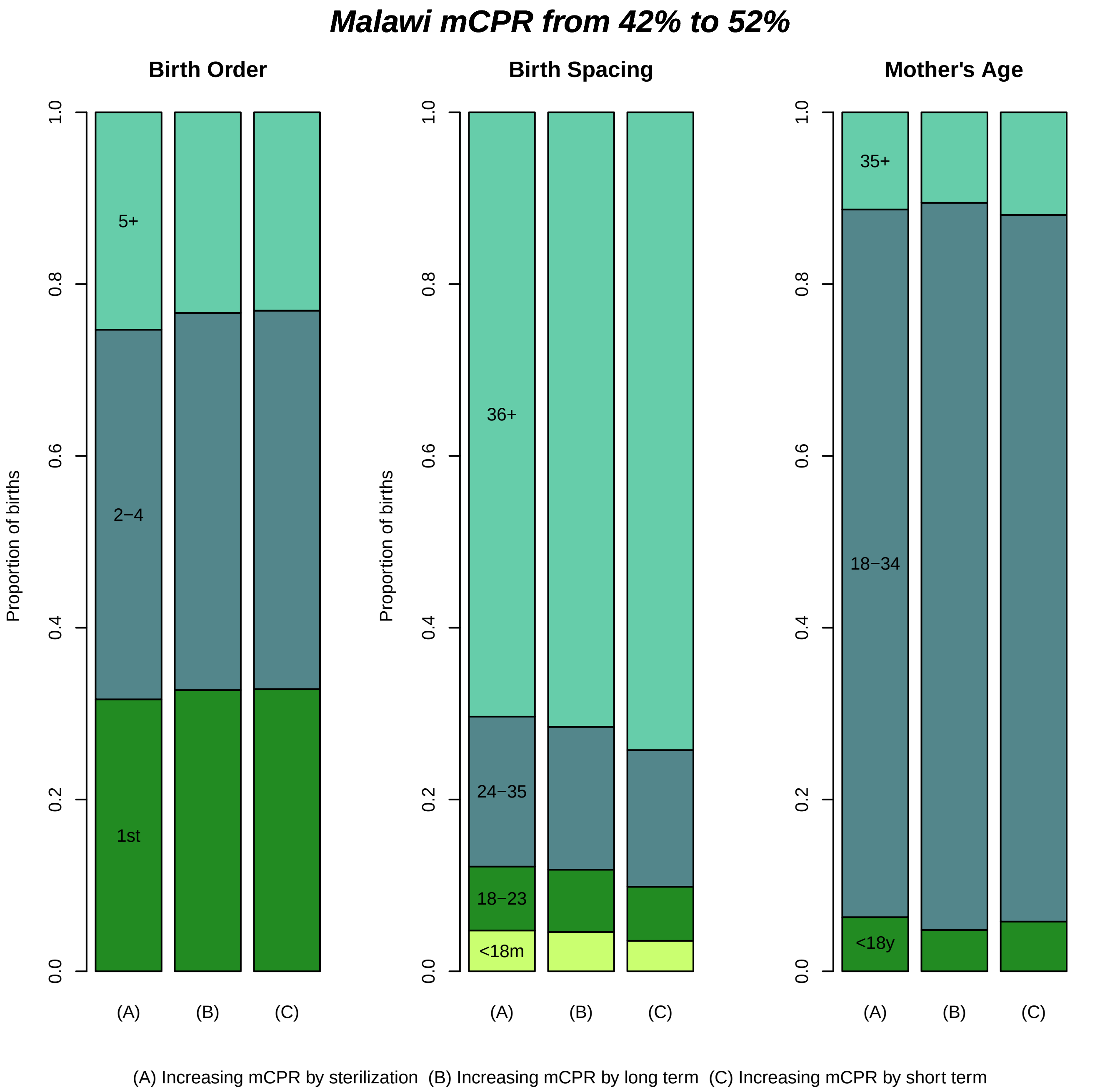

Malawi mCPR from 42% to 52%
Birth Order
Birth Spacing
Mother's Age
Proportion of births
0.0
0.2
0.4
0.6
0.8
1.0
5+
2−4
1st
36+
24−35
18−23
<18m
35+
18−34
<18y
(A)
(B)
(C)
(A) Increasing mCPR by sterilization (B) Increasing mCPR by long term (C) Increasing mCPR by short term

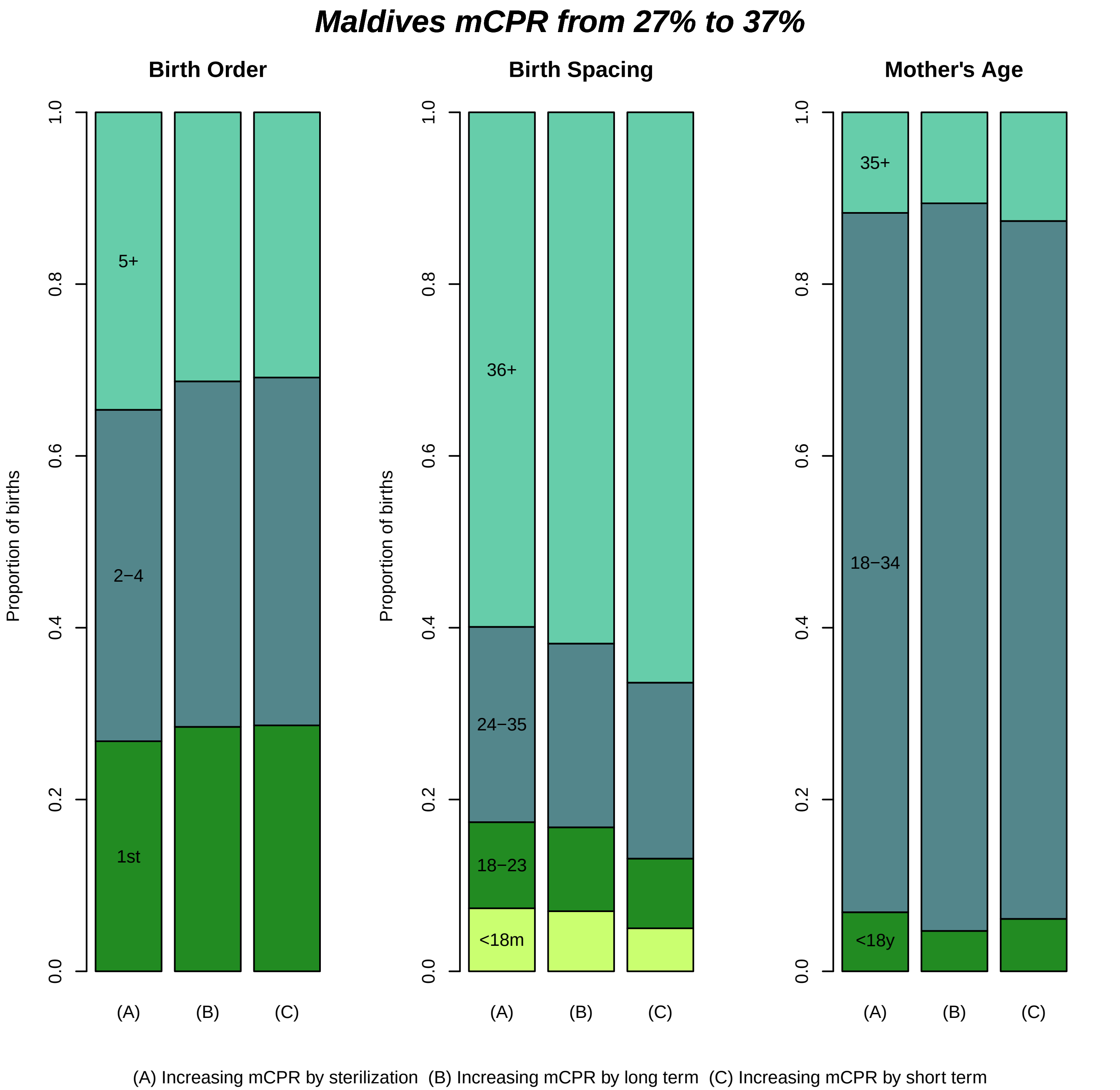

Maldives mCPR from 27% to 37%
Birth Order
Birth Spacing
Mother's Age
Proportion of births
0.0
0.2
0.4
0.6
0.8
1.0
5+
2−4
1st
36+
24−35
18−23
<18m
35+
18−34
<18y
(A)
(B)
(C)
(A) Increasing mCPR by sterilization (B) Increasing mCPR by long term (C) Increasing mCPR by short term

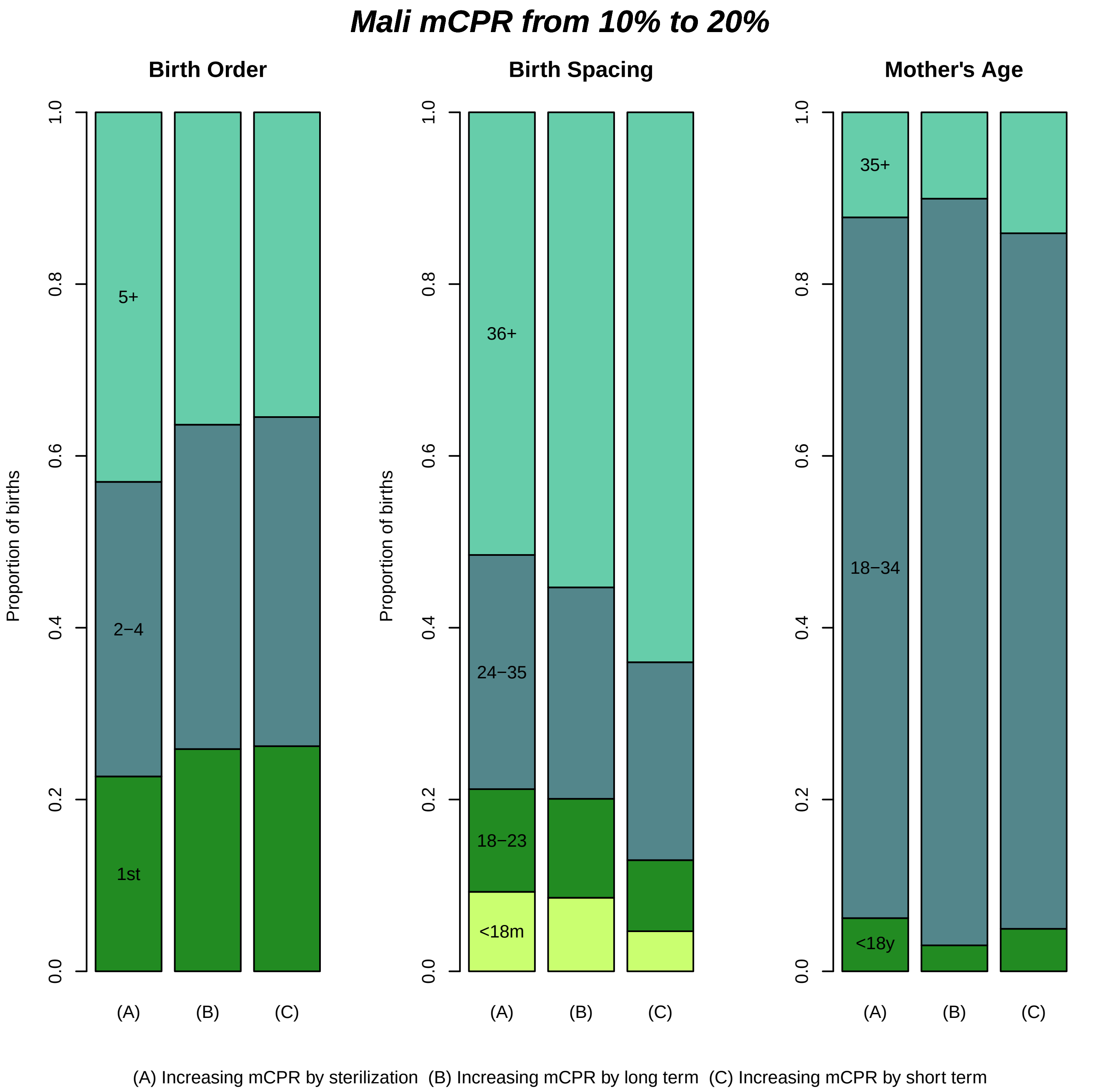

Mali mCPR from 10% to 20%
Birth Order
Birth Spacing
Mother's Age
Proportion of births
0.0
0.2
0.4
0.6
0.8
1.0
5+
2−4
1st
36+
24−35
18−23
<18m
35+
18−34
<18y
(A)
(B)
(C)
(A) Increasing mCPR by sterilization (B) Increasing mCPR by long term (C) Increasing mCPR by short term

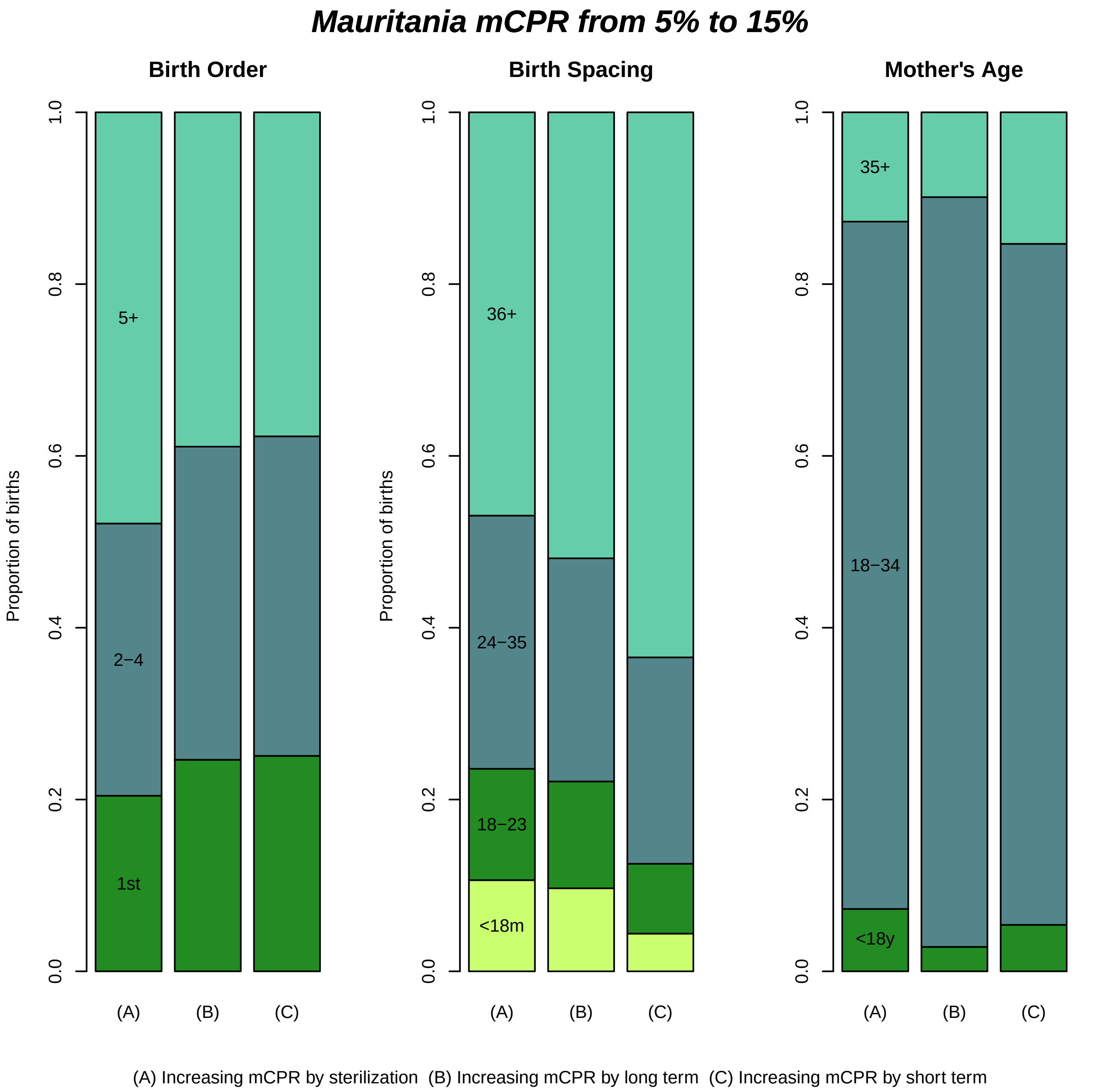

Mauritania mCPR from 5% to 15%
Birth Order
Birth Spacing
Mother's Age
Proportion of births
Proportion of births
0.0
0.2
0.4
0.6
0.8
1.0
5+
2−4
1st
36+
24−35
18−23
<18m
35+
18−34
<18y
(A)
(B)
(C)
(A) Increasing mCPR by sterilization (B) Increasing mCPR by long term (C) Increasing mCPR by short term

Moldova mCPR from 44% to 54%

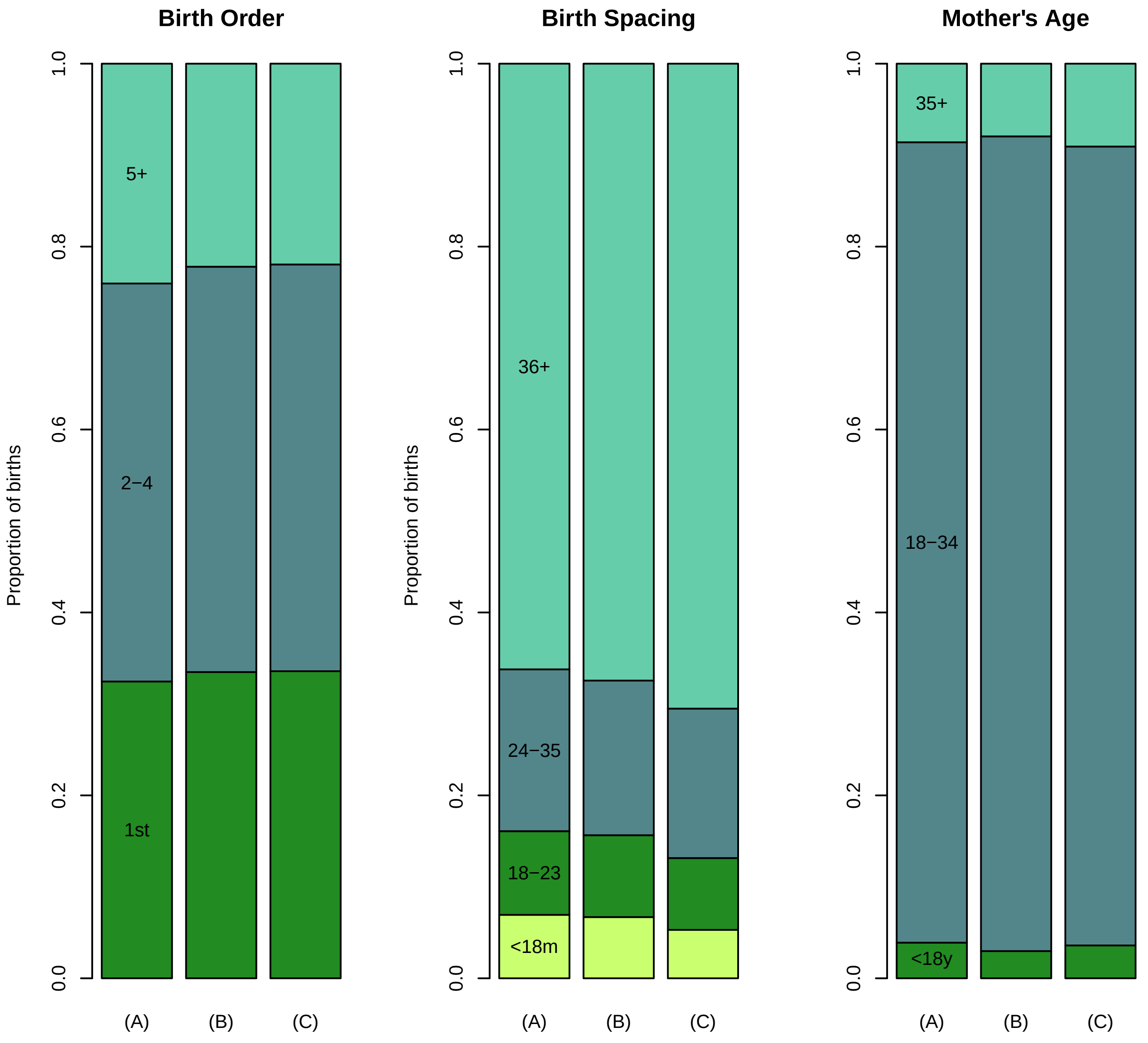

(A) Increasing mCPR by sterilization (B) Increasing mCPR by long term (C) Increasing mCPR by short term

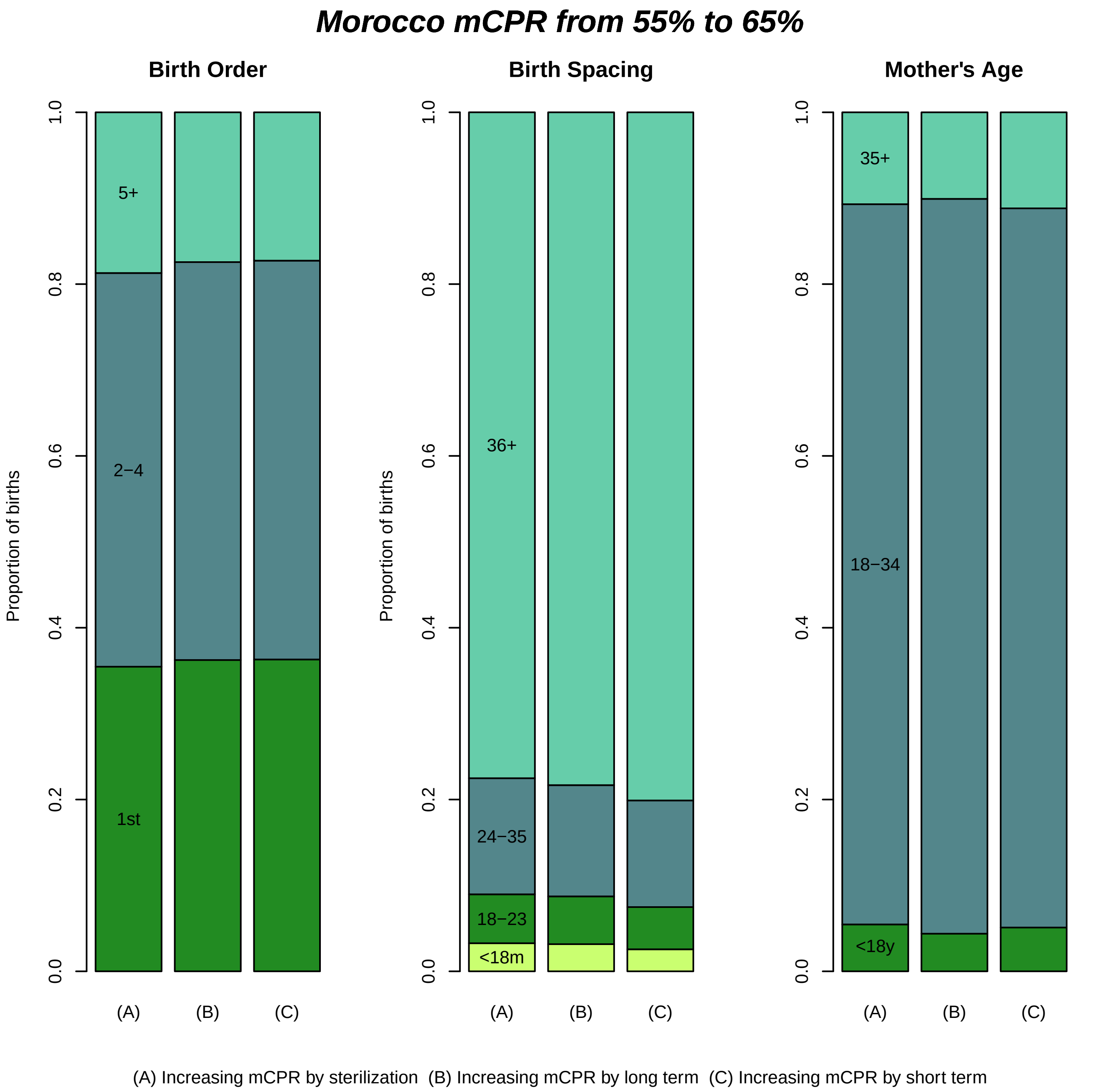

Morocco mCPR from 55% to 65%
Birth Order
Birth Spacing
Mother's Age
Proportion of births
0.0
0.2
0.4
0.6
0.8
1.0
5+
2−4
1st
36+
24−35
18−23
<18m
35+
18−34
<18y
(A)
(B)
(C)
(A) Increasing mCPR by sterilization (B) Increasing mCPR by long term (C) Increasing mCPR by short term

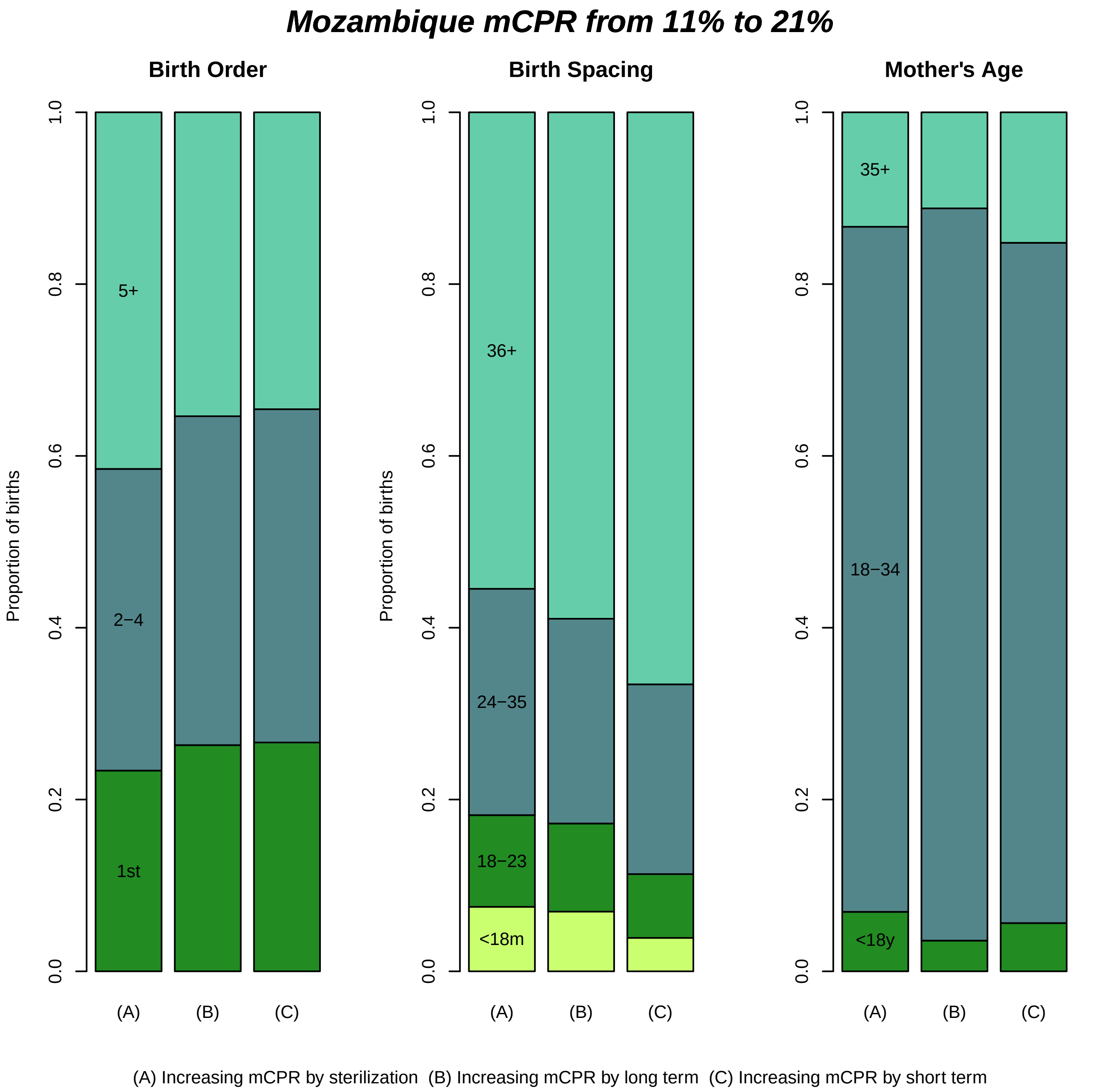

Mozambique mCPR from 11% to 21%
Birth Order
Birth Spacing
Mother's Age
Proportion of births
1.0
0.8
0.6
0.4
0.2
0.0
5+
2−4
1st
36+
24−35
18−23
<18m
35+
18−34
<18y
(A)
(B)
(C)
(A) Increasing mCPR by sterilization (B) Increasing mCPR by long term (C) Increasing mCPR by short term

Namibia mCPR from 53% to 63%

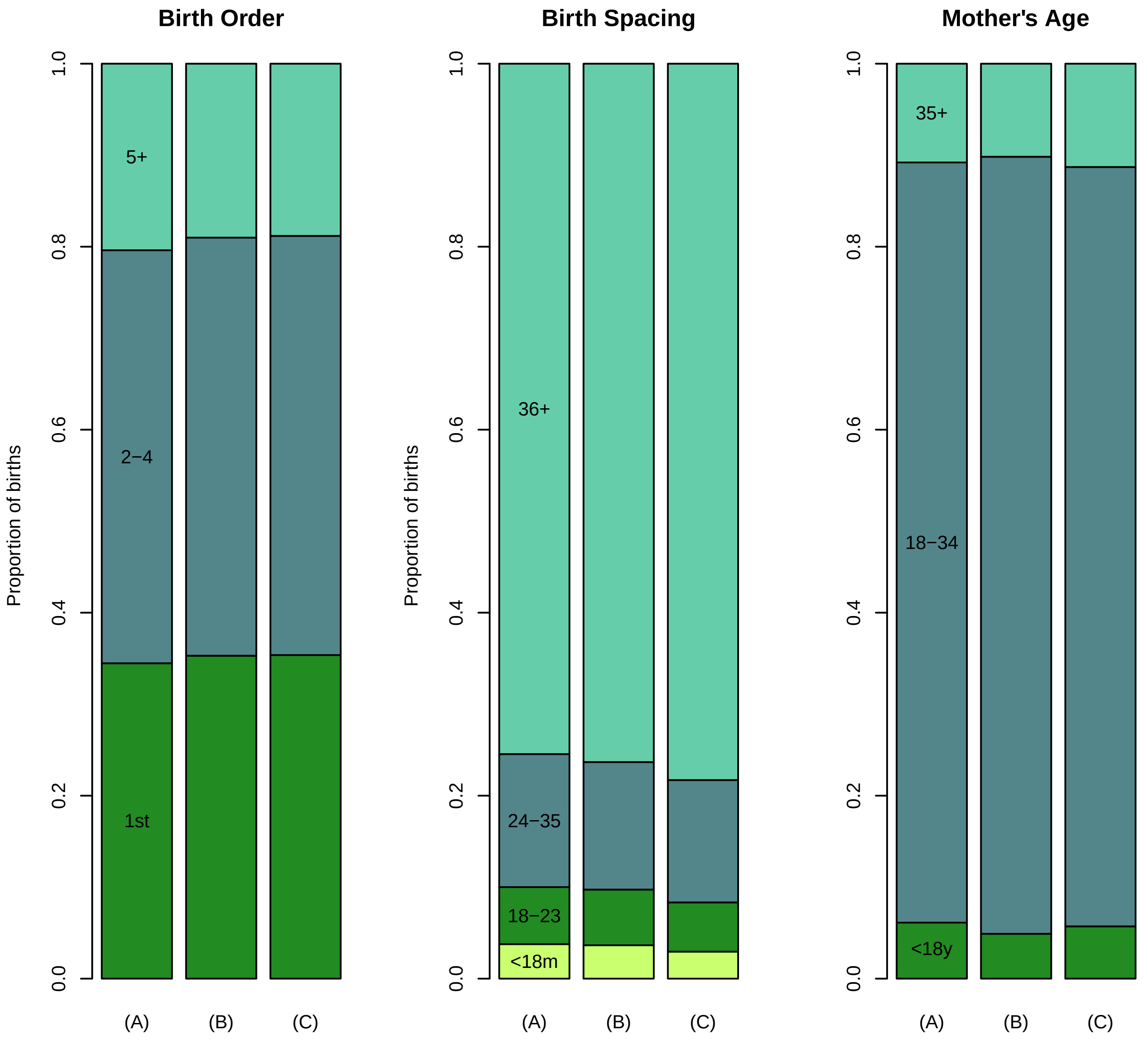

(A) Increasing mCPR by sterilization (B) Increasing mCPR by long term (C) Increasing mCPR by short term

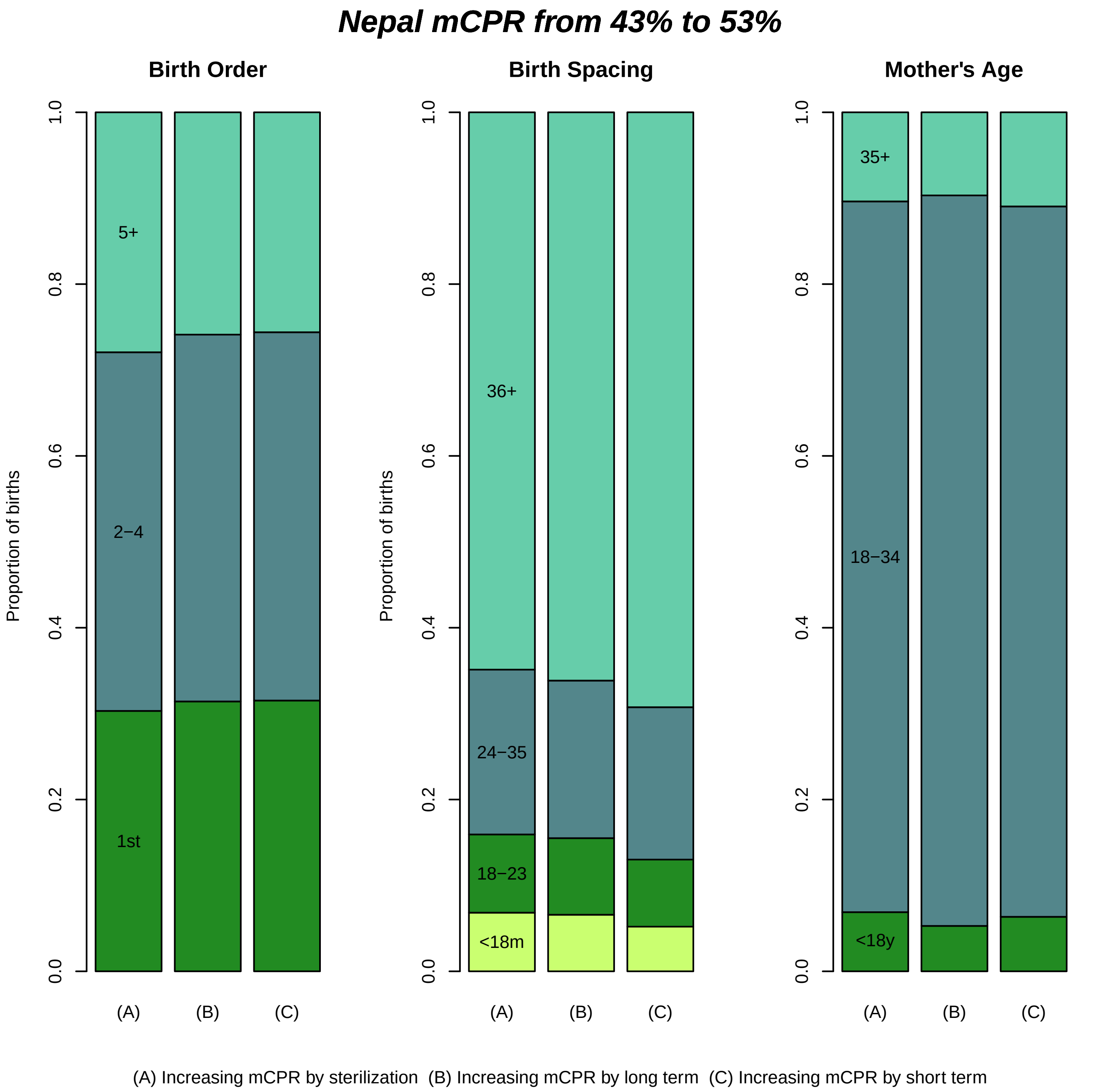

Nepal mCPR from 43% to 53%
Birth Order
Birth Spacing
Mother's Age
Proportion of births
0.0
0.2
0.4
0.6
0.8
1.0
5+
2−4
1st
36+
24−35
18−23
<18m
35+
18−34
<18y
(A)
(B)
(C)
(A) Increasing mCPR by sterilization (B) Increasing mCPR by long term (C) Increasing mCPR by short term

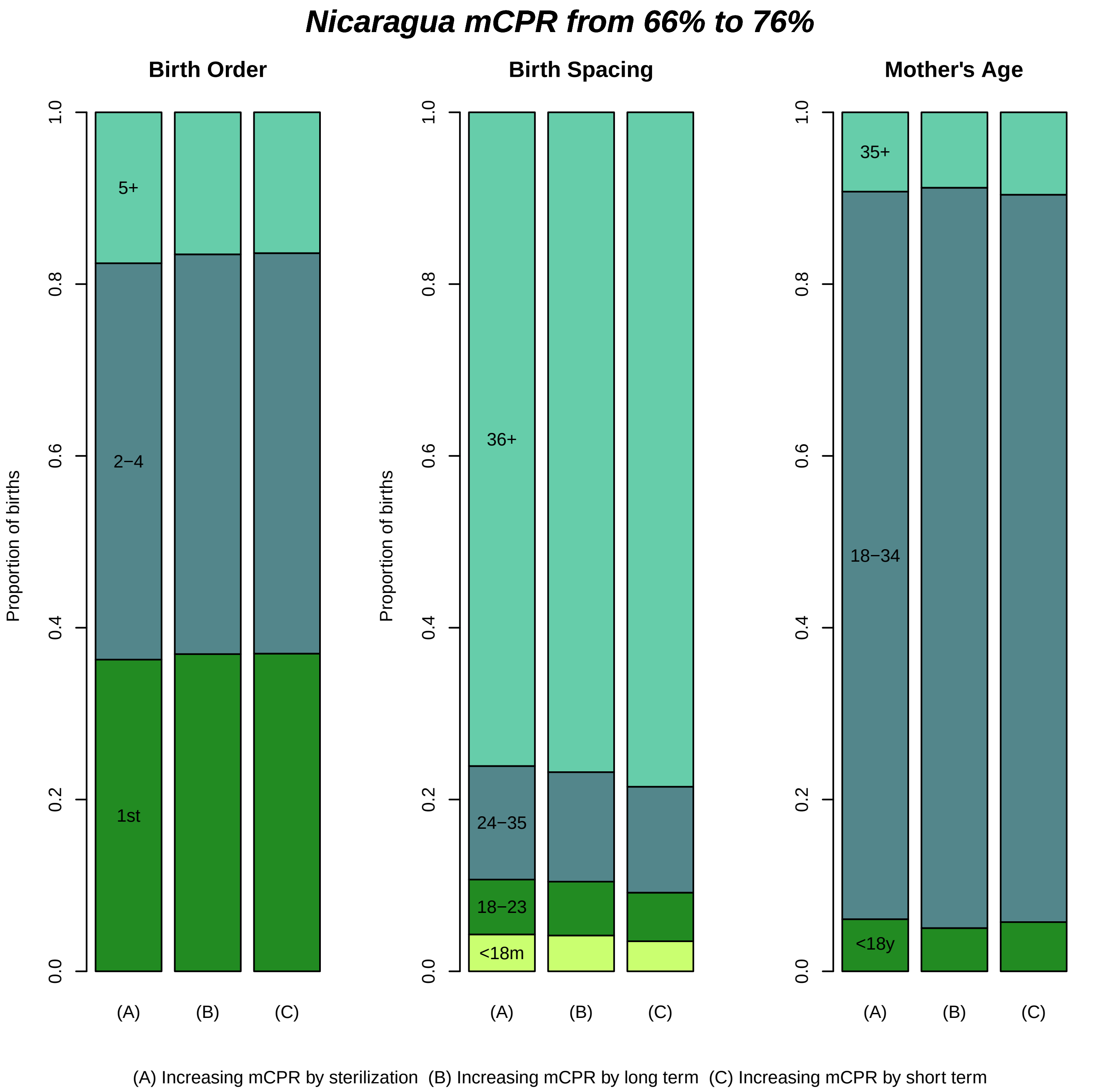

Nicaragua mCPR from 66% to 76%
Birth Order
Birth Spacing
Mother's Age
Proportion of births
1.0
0.8
0.6
0.4
0.2
0.0
5+
2−4
1st
36+
24−35
18−23
<18m
35+
18−34
<18y
(A)
(B)
(C)
(A) Increasing mCPR by sterilization (B) Increasing mCPR by long term (C) Increasing mCPR by short term

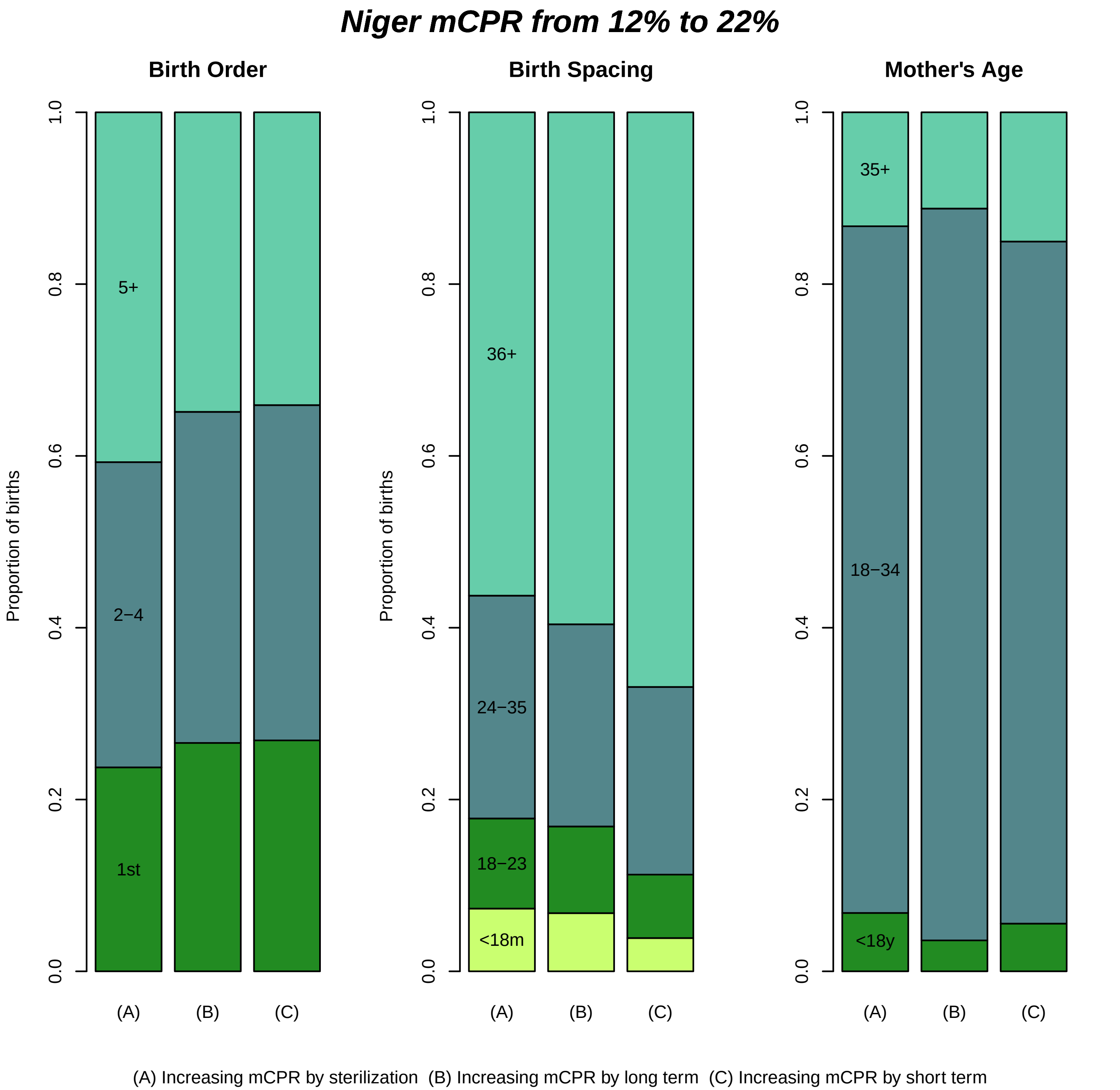

Niger mCPR from 12% to 22%
Birth Order
Birth Spacing
Mother's Age
Proportion of births
Proportion of births
1.0
0.8
0.6
0.4
0.2
0.0
5+
2−4
1st
36+
24−35
18−23
<18m
35+
18−34
<18y
(A)
(B)
(C)
(A) Increasing mCPR by sterilization (B) Increasing mCPR by long term (C) Increasing mCPR by short term

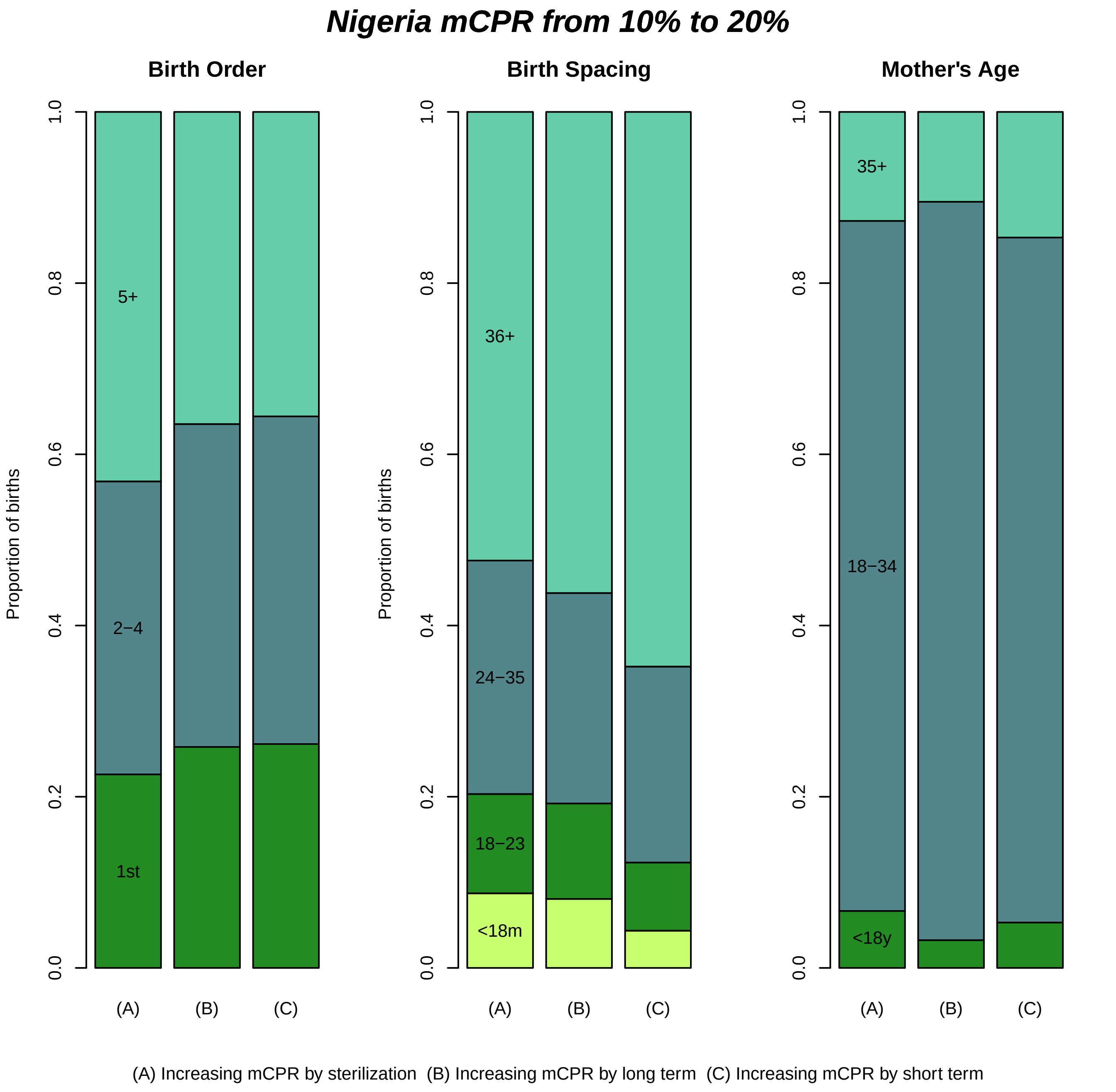

Nigeria mCPR from 10% to 20%
Birth Order
Birth Spacing
Mother's Age
Proportion of births
0.0
0.2
0.4
0.6
0.8
1.0
5+
2−4
1st
36+
24−35
18−23
<18m
35+
18−34
<18y
(A)
(B)
(C)
(A) Increasing mCPR by sterilization (B) Increasing mCPR by long term (C) Increasing mCPR by short term

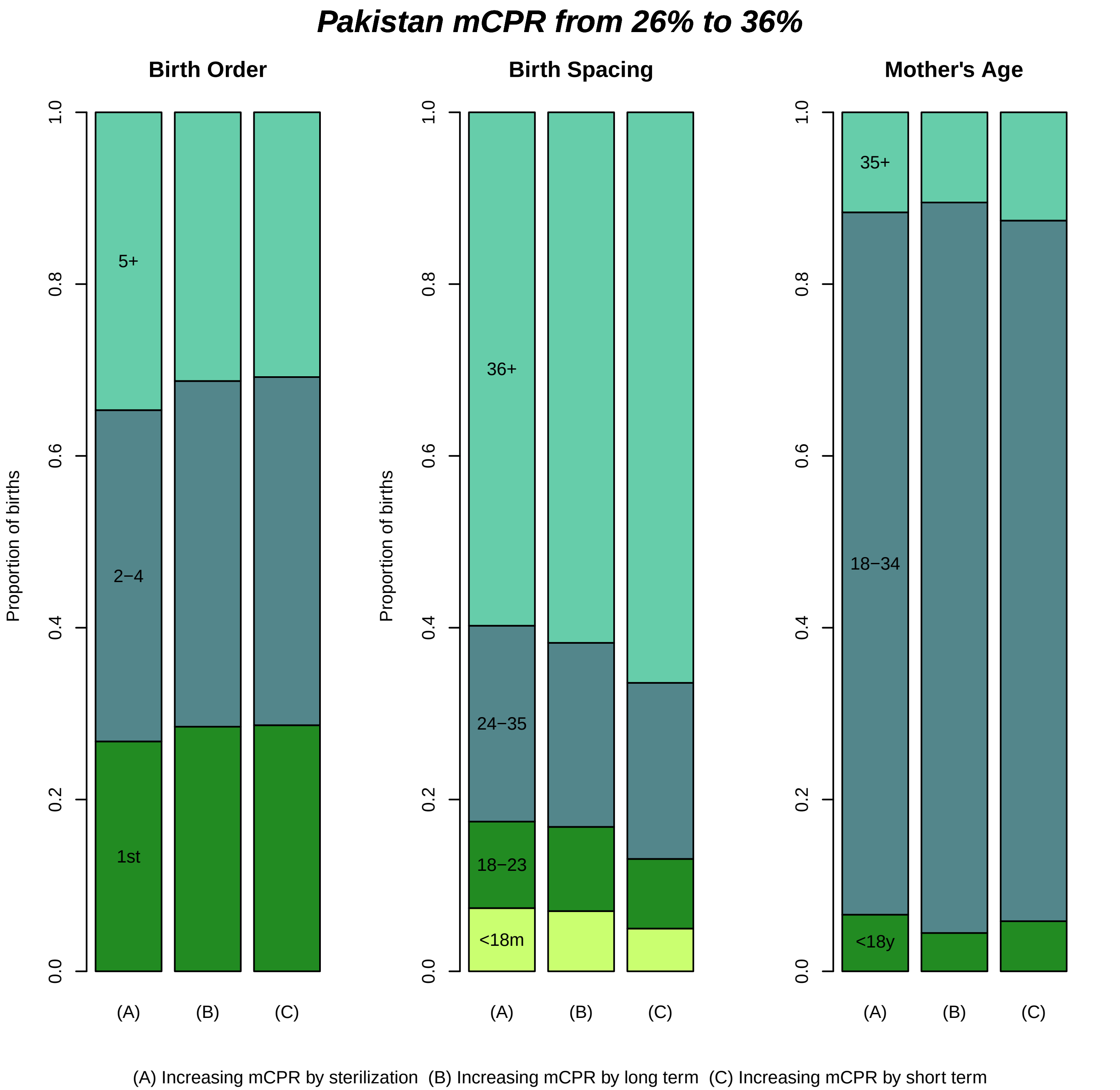

Pakistan mCPR from 26% to 36%
Birth Order
Birth Spacing
Mother's Age
Proportion of births
0.0
0.2
0.4
0.6
0.8
1.0
5+
2−4
1st
36+
24−35
18−23
<18m
35+
18−34
<18y
(A)
(B)
(C)
(A) Increasing mCPR by sterilization (B) Increasing mCPR by long term (C) Increasing mCPR by short term

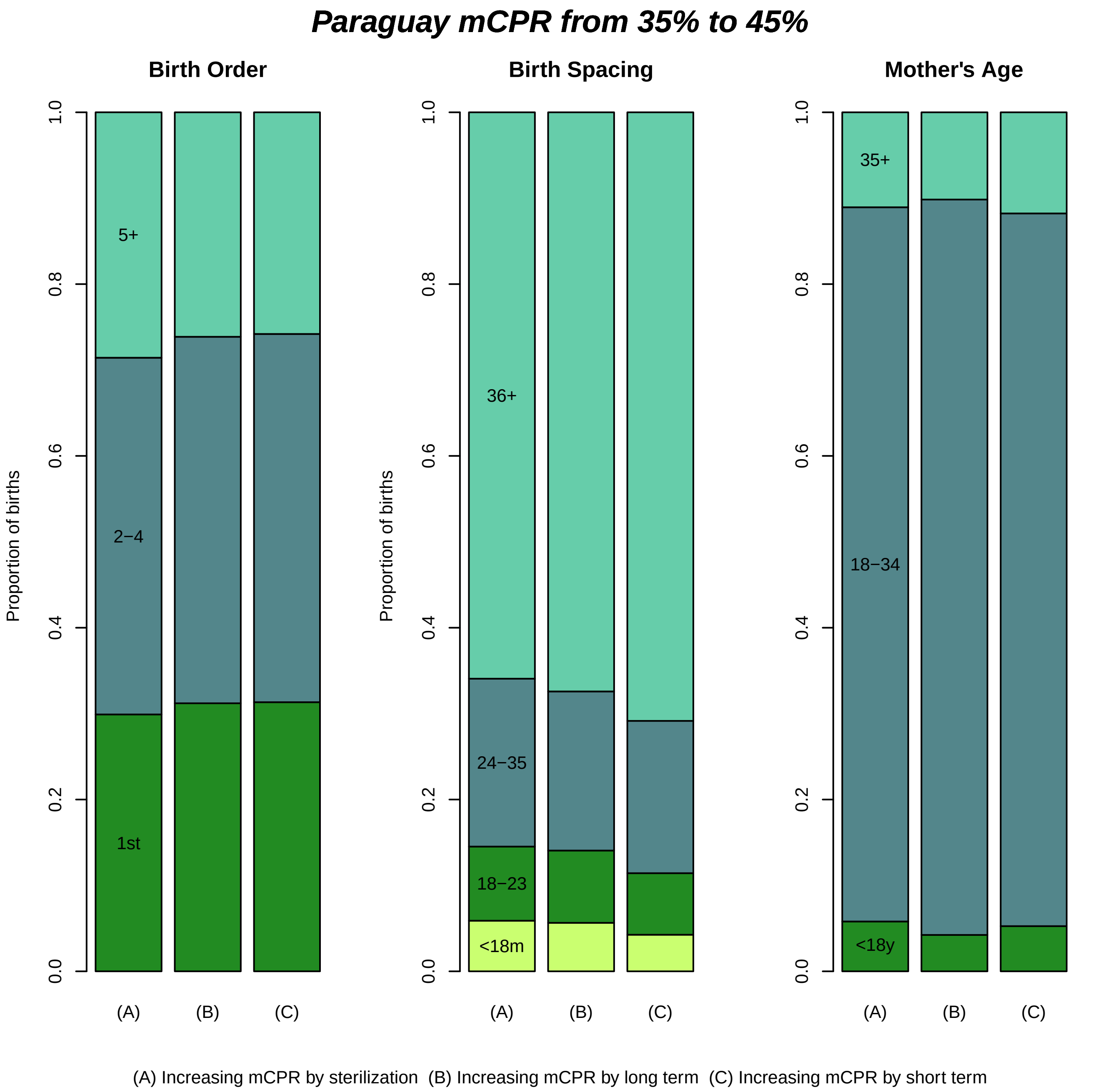
Paraguay mCPR from 35% to 45%
Birth Order
Birth Spacing
Mother's Age
Proportion of births
0.0
0.2
0.4
0.6
0.8
1.0
5+
2−4
1st
36+
24−35
18−23
<18m
35+
18−34
<18y
(A)
(B)
(C)
(A) Increasing mCPR by sterilization (B) Increasing mCPR by long term (C) Increasing mCPR by short term

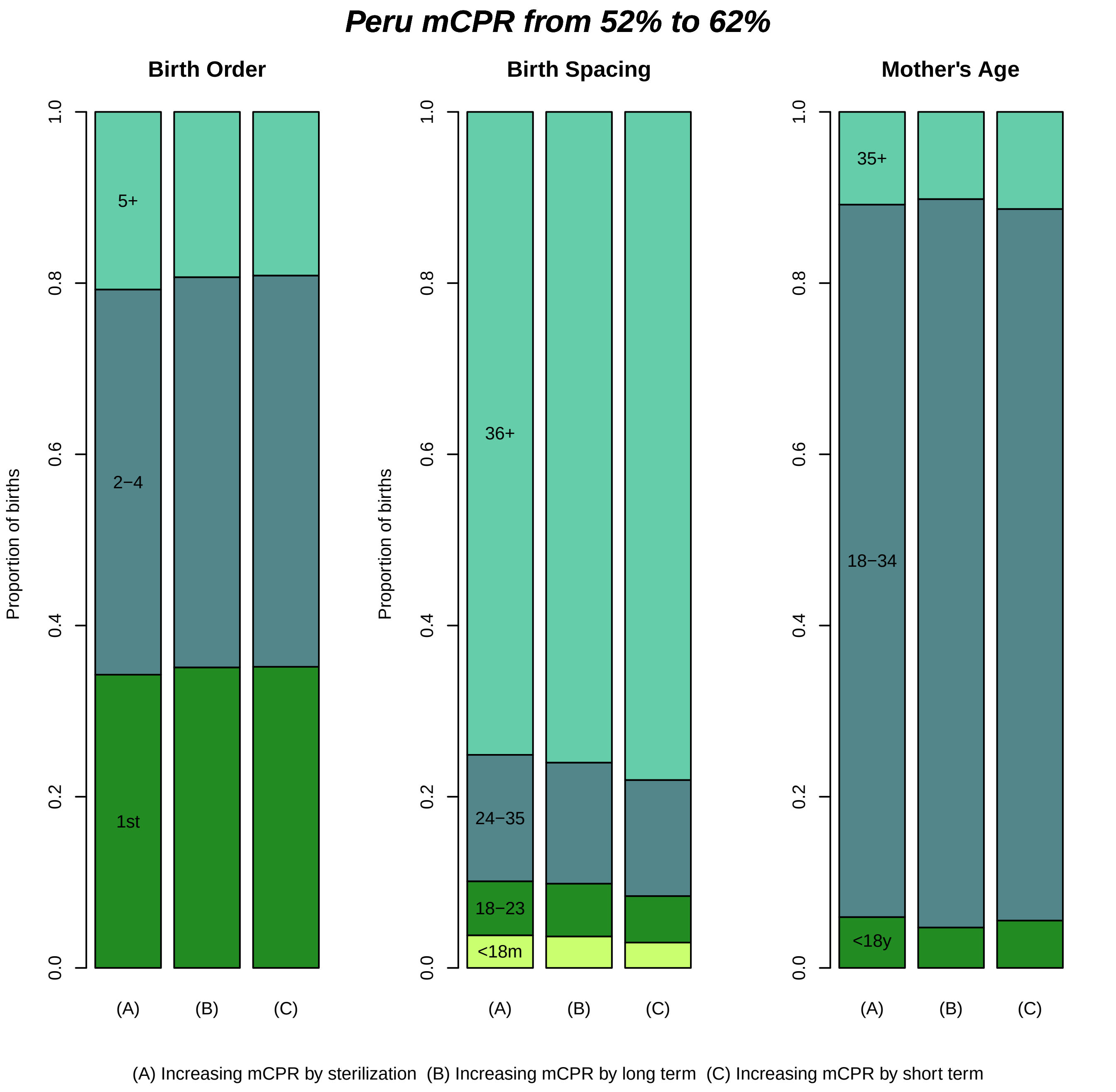
Peru mCPR from 52% to 62%
Birth Order
Birth Spacing
Mother's Age
Proportion of births
0.0
0.2
0.4
0.6
0.8
1.0
5+
2−4
1st
36+
24−35
18−23
<18m
35+
18−34
<18y
(A)
(B)
(C)
(A) Increasing mCPR by sterilization (B) Increasing mCPR by long term (C) Increasing mCPR by short term

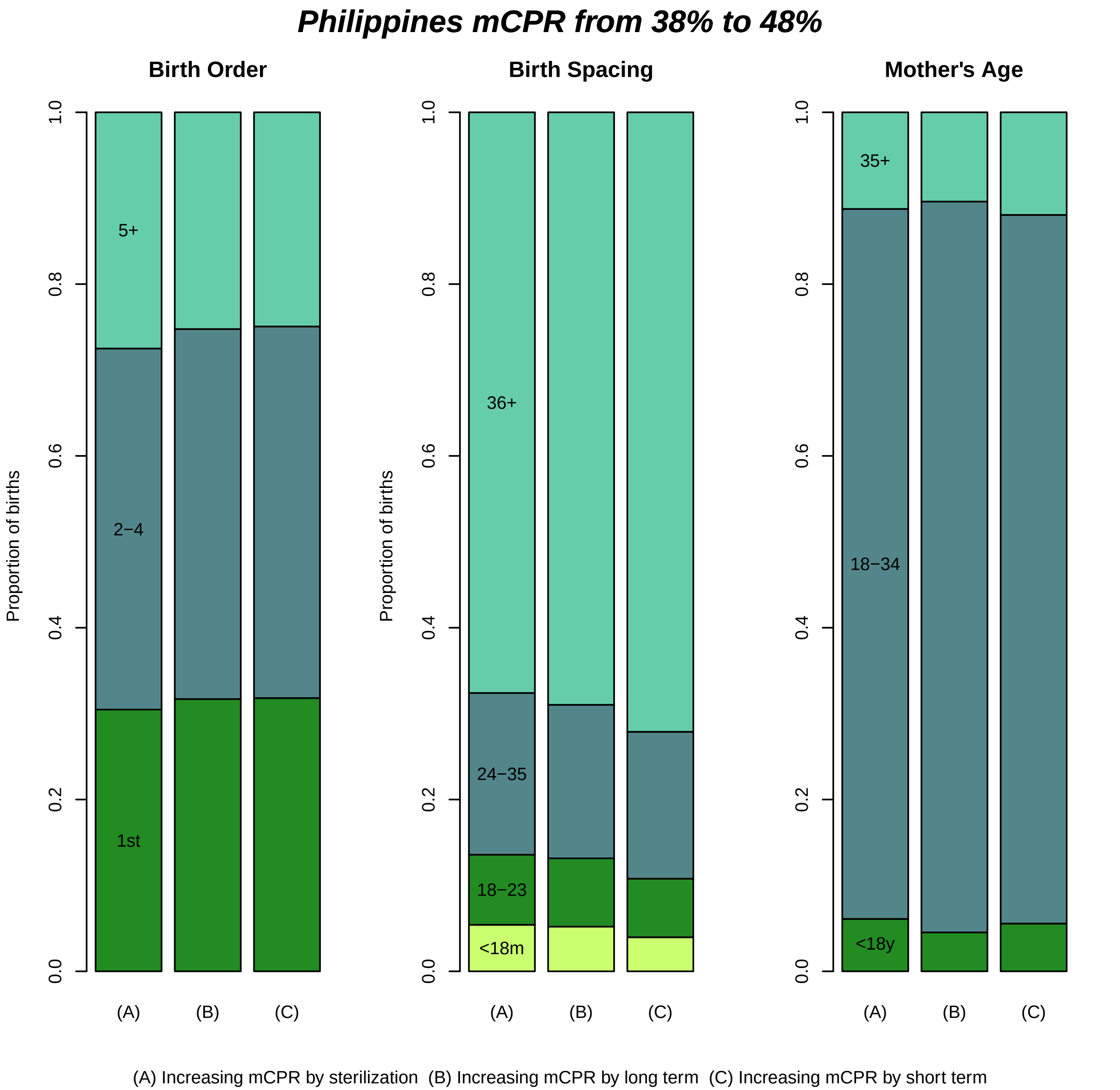
Philippines mCPR from 38% to 48%
Birth Order
Birth Spacing
Mother's Age
Proportion of births
1.0
0.8
0.6
0.4
0.2
0.0
5+
2−4
1st
36+
24−35
18−23
<18m
35+
18−34
<18y
(A)
(B)
(C)
(A) Increasing mCPR by sterilization (B) Increasing mCPR by long term (C) Increasing mCPR by short term

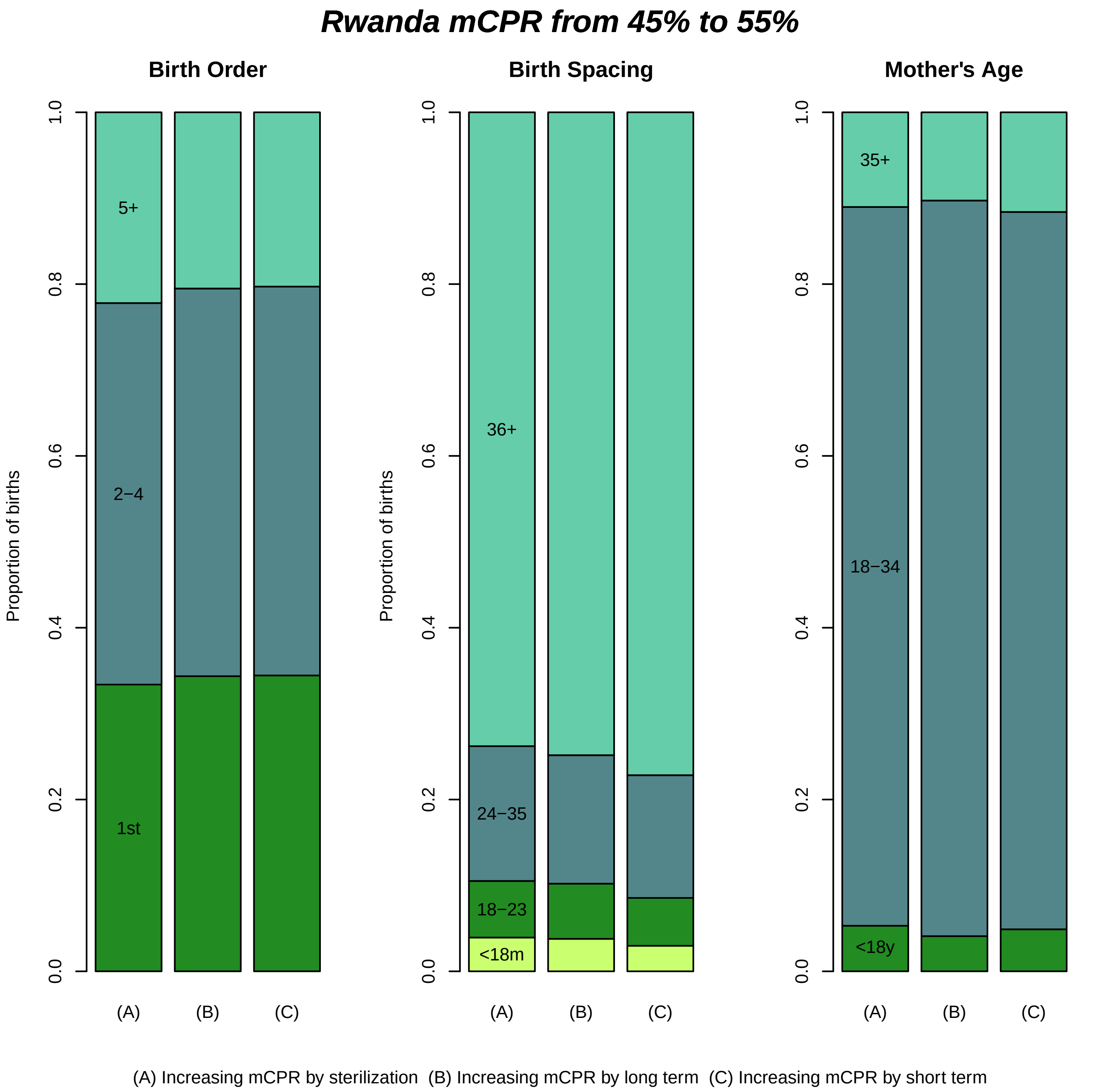
Rwanda mCPR from 45% to 55%
Birth Order
Birth Spacing
Mother's Age
Proportion of births
0.0
0.2
0.4
0.6
0.8
1.0
5+
2−4
1st
36+
24−35
18−23
<18m
35+
18−34
<18y
(A)
(B)
(C)
(A) Increasing mCPR by sterilization (B) Increasing mCPR by long term (C) Increasing mCPR by short term

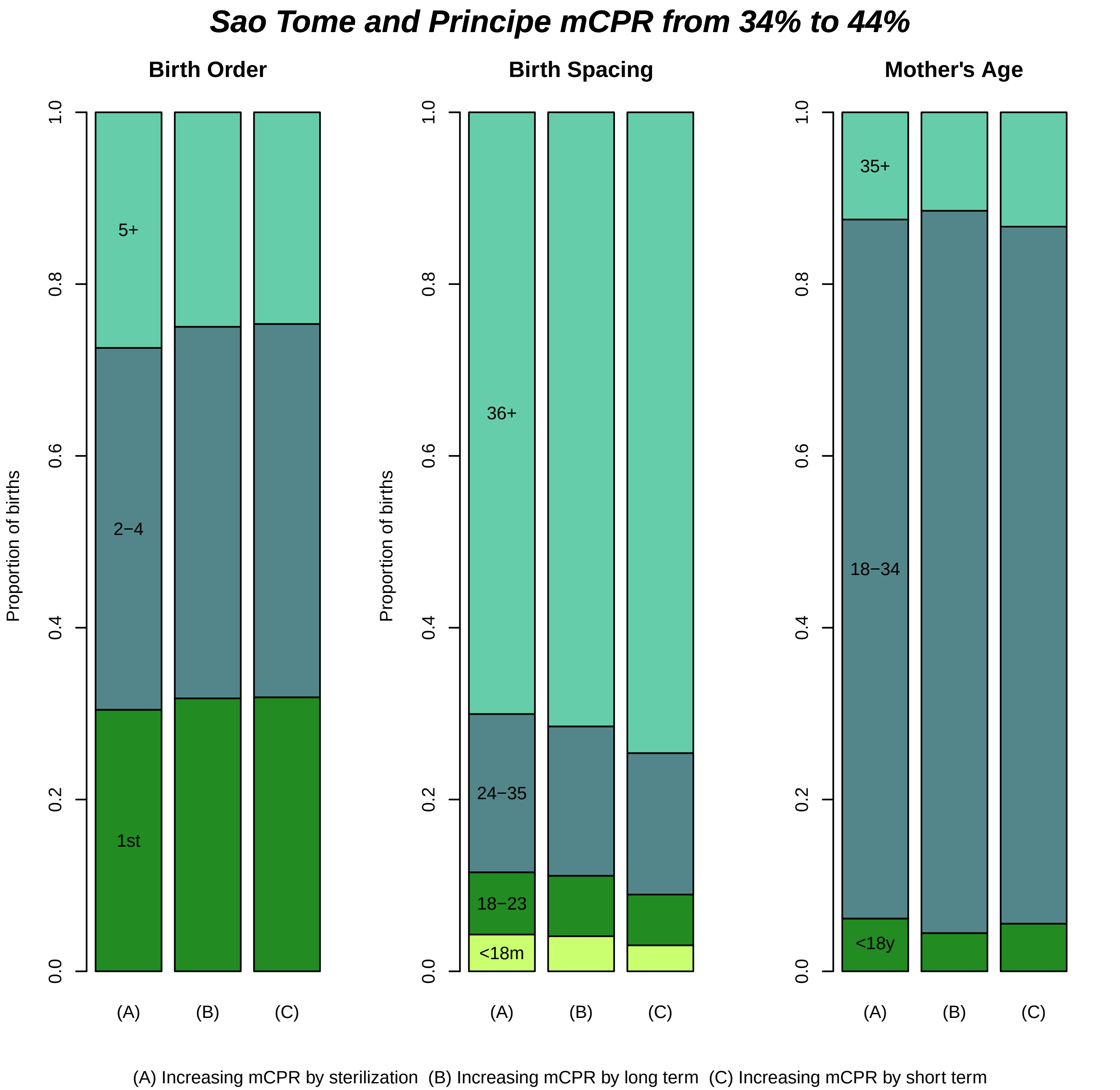

Sao Tome and Principe mCPR from 34% to 44%
Birth Order
Birth Spacing
Mother's Age
Proportion of births
0.0
0.2
0.4
0.6
0.8
1.0
5+
2−4
1st
36+
24−35
18−23
<18m
35+
18−34
<18y
(A)
(B)
(C)
(A) Increasing mCPR by sterilization (B) Increasing mCPR by long term (C) Increasing mCPR by short term

Senegal mCPR from 16% to 26%

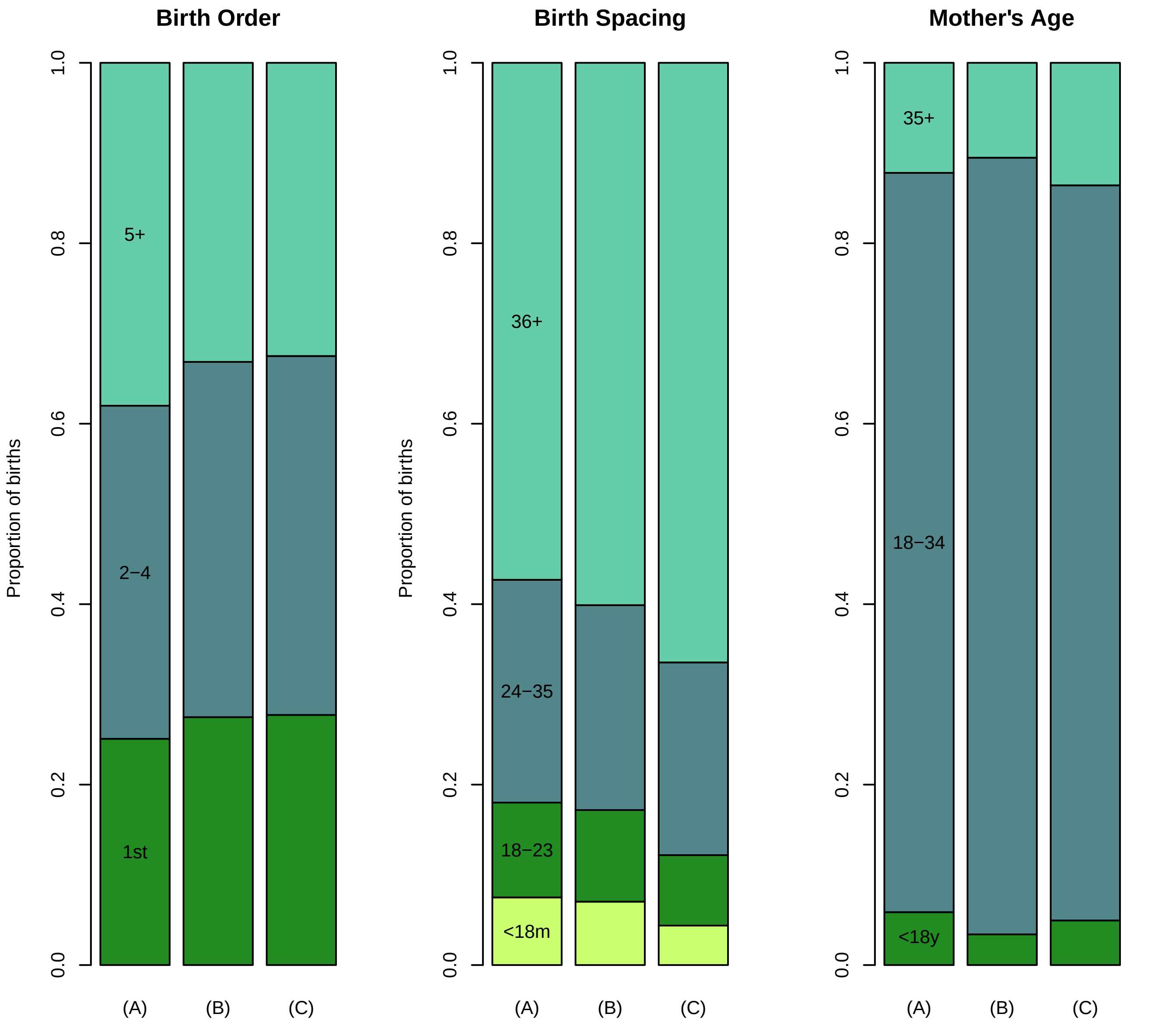

(A) Increasing mCPR by sterilization (B) Increasing mCPR by long term (C) Increasing mCPR by short term

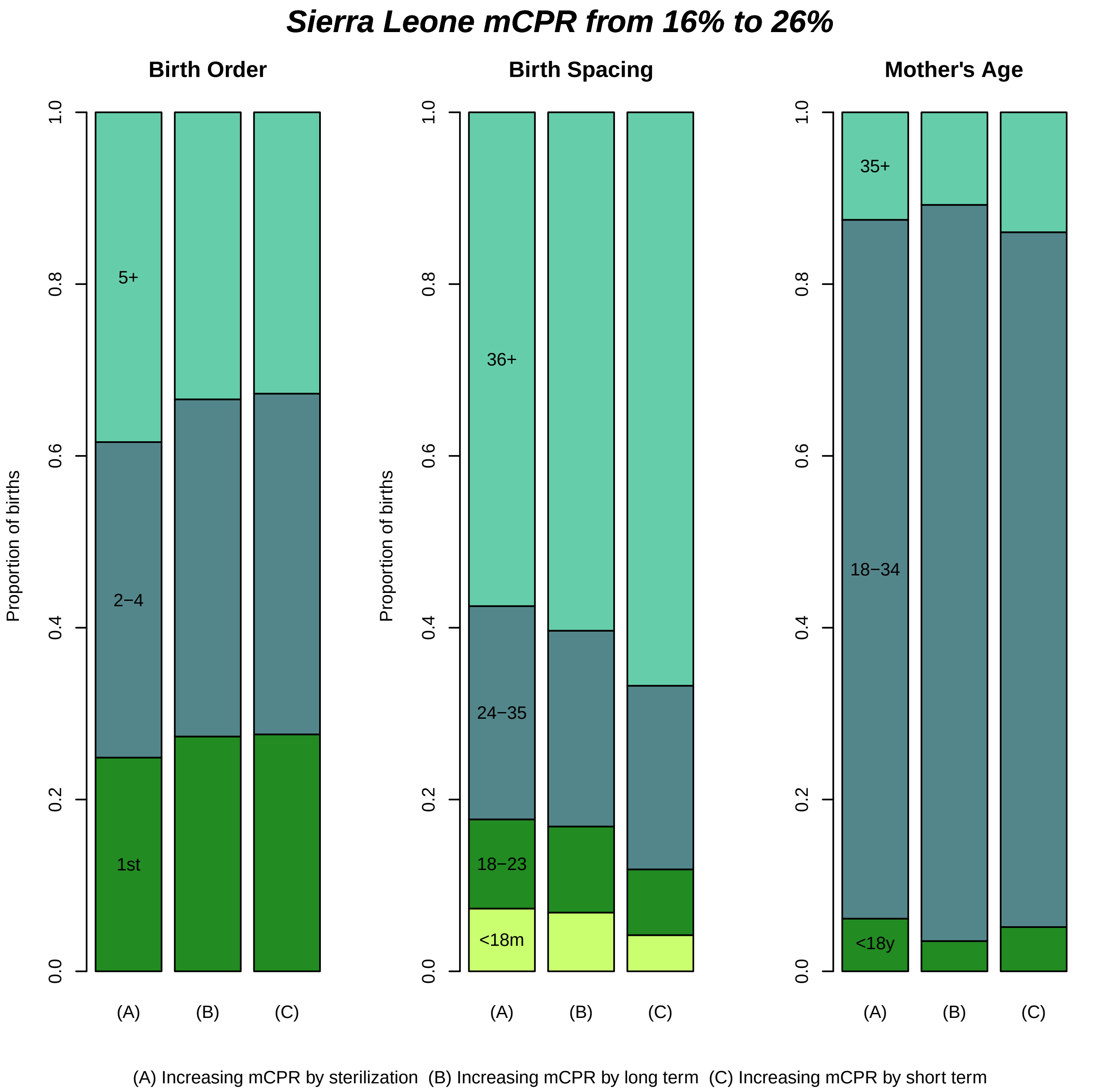

Sierra Leone mCPR from 16% to 26%
Birth Order
Birth Spacing
Mother's Age
Proportion of births
0.0
0.2
0.4
0.6
0.8
1.0
5+
2−4
1st
36+
24−35
18−23
<18m
35+
18−34
<18y
(A)
(B)
(C)
(A) Increasing mCPR by sterilization (B) Increasing mCPR by long term (C) Increasing mCPR by short term

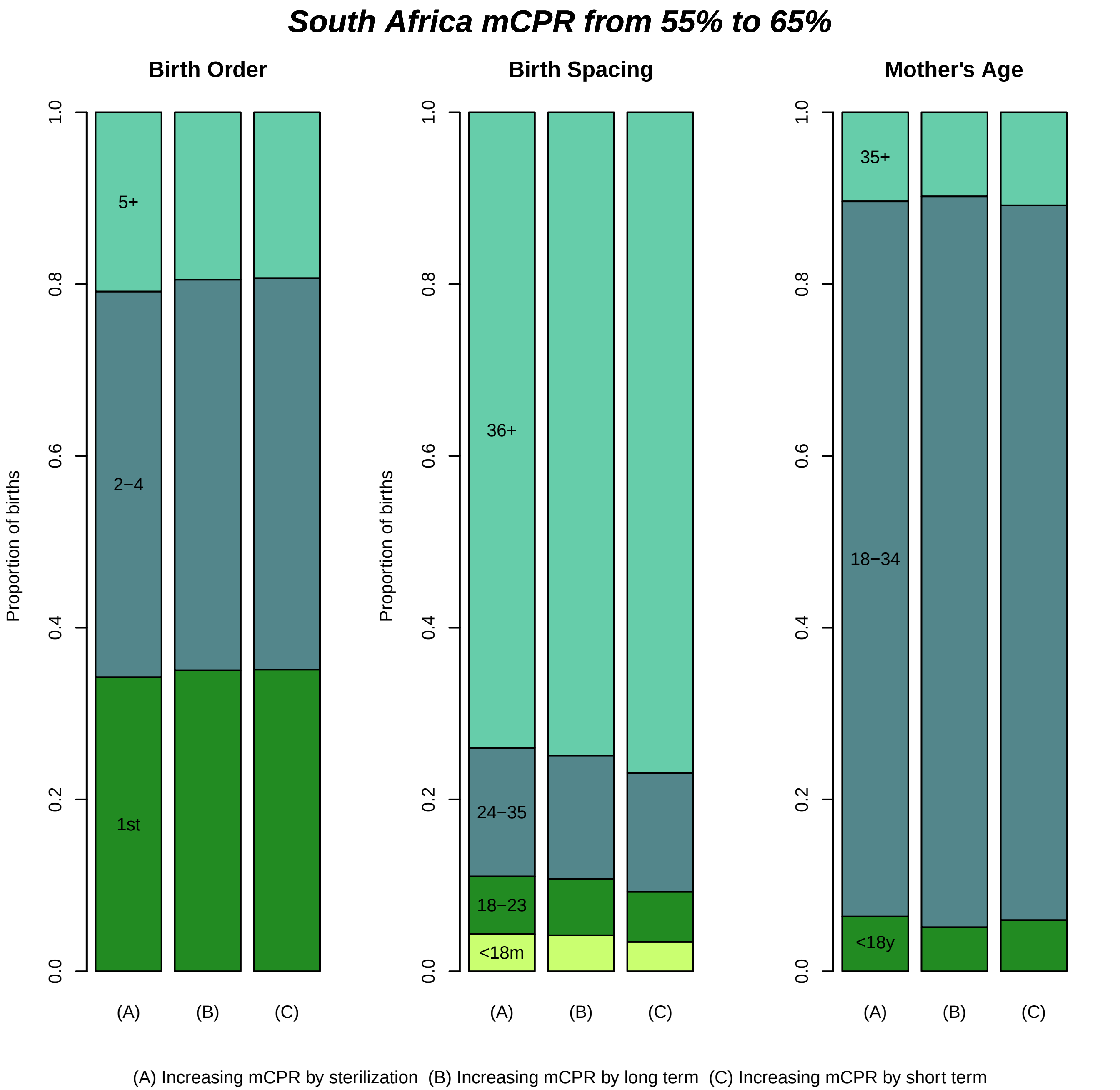

South Africa mCPR from 55% to 65%
Birth Order
Birth Spacing
Mother's Age
Proportion of births
Proportion of births
0.0
0.2
0.4
0.6
0.8
1.0
5+
2−4
1st
36+
24−35
18−23
<18m
35+
18−34
<18y
(A)
(B)
(C)
(A) Increasing mCPR by sterilization (B) Increasing mCPR by long term (C) Increasing mCPR by short term

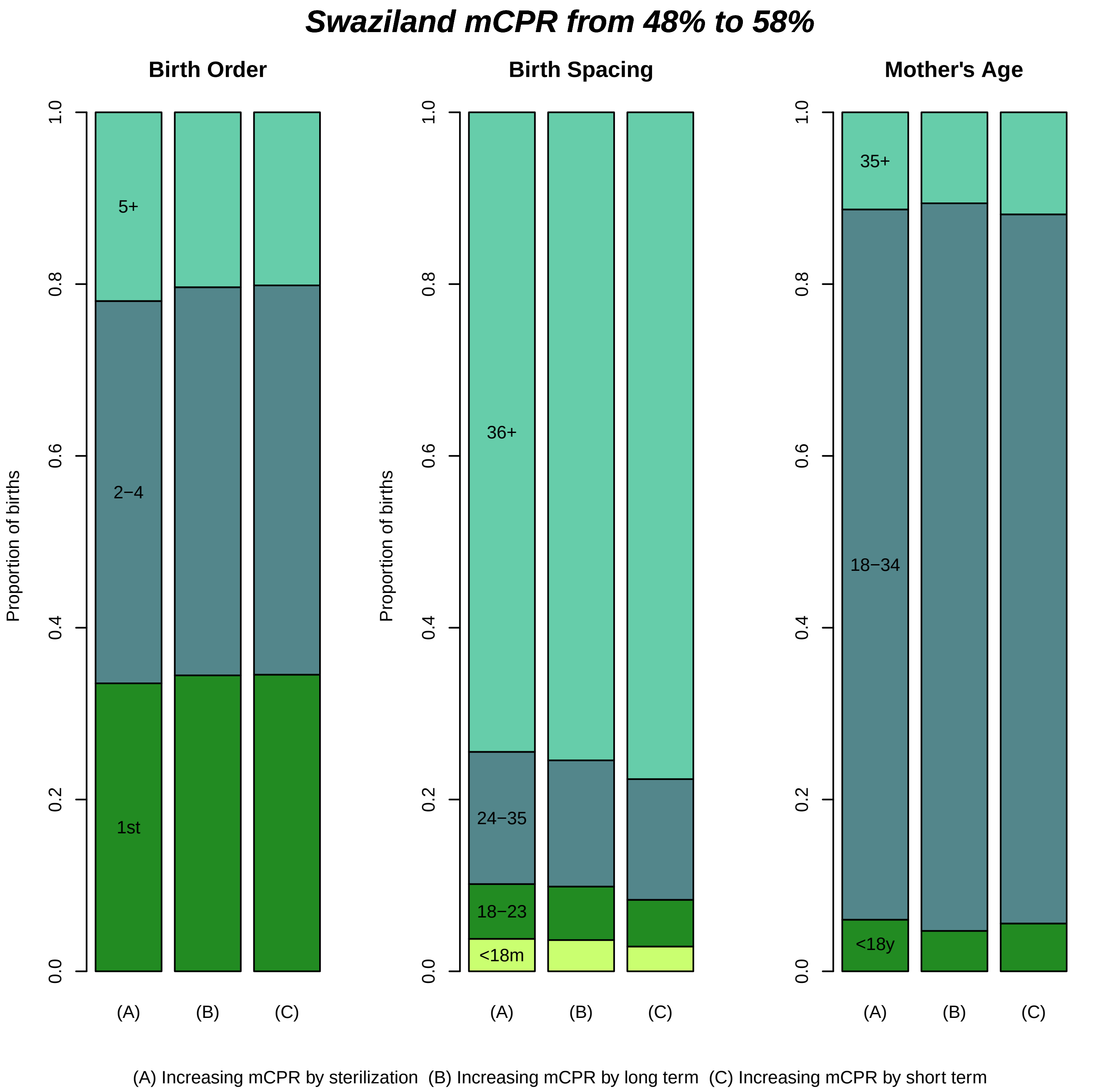

Swaziland mCPR from 48% to 58%
Birth Order
Birth Spacing
Mother's Age
Proportion of births
0.0
0.2
0.4
0.6
0.8
1.0
5+
2−4
1st
36+
24−35
18−23
<18m
35+
18−34
<18y
(A)
(B)
(C)
(A) Increasing mCPR by sterilization (B) Increasing mCPR by long term (C) Increasing mCPR by short term

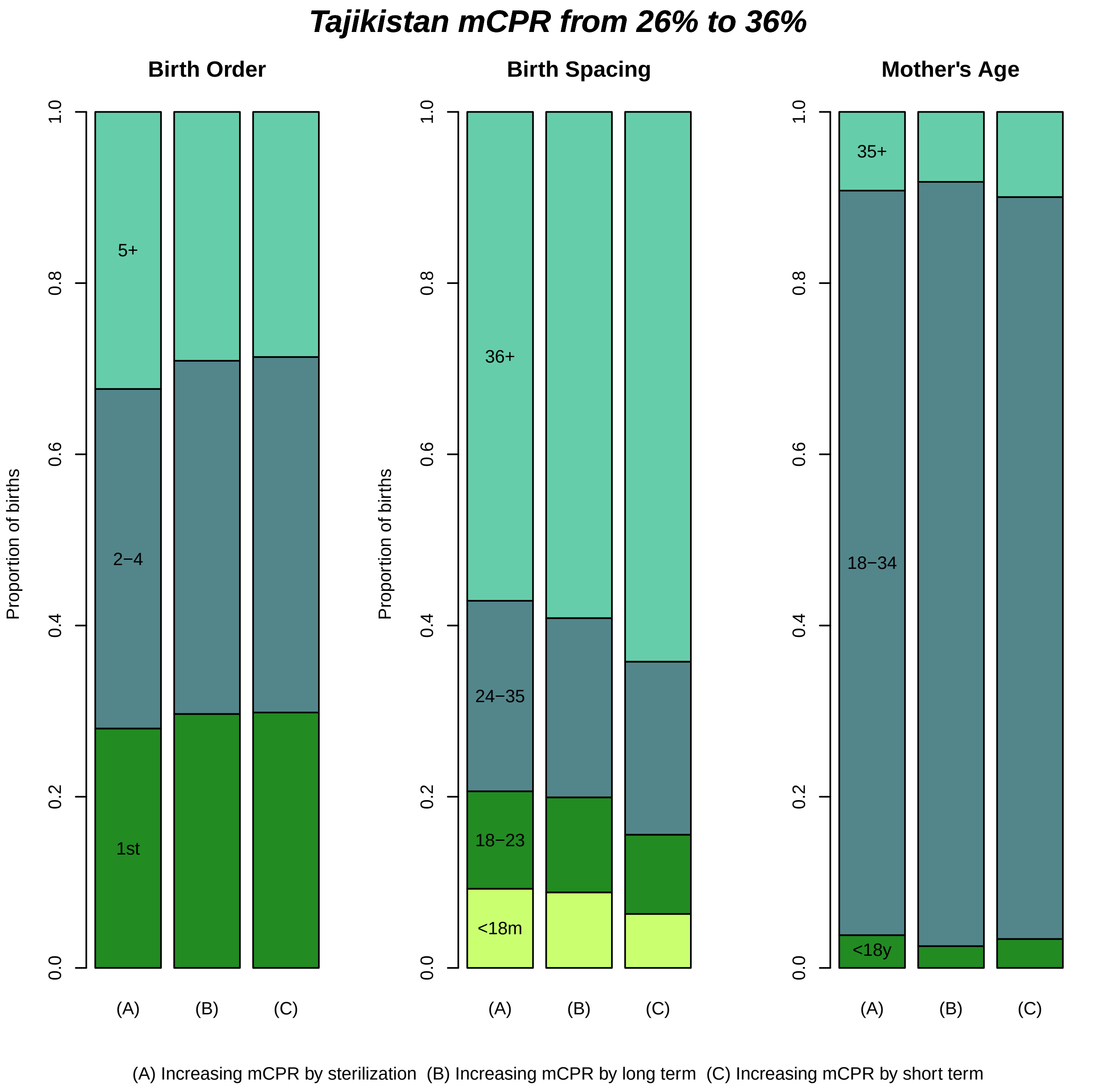

Tajikistan mCPR from 26% to 36%
Birth Order
Birth Spacing
Mother's Age
Proportion of births
Proportion of births
1.0
0.8
0.6
0.4
0.2
0.0
5+
2−4
1st
36+
24−35
18−23
<18m
35+
18−34
<18y
(A)
(B)
(C)
(A) Increasing mCPR by sterilization (B) Increasing mCPR by long term (C) Increasing mCPR by short term

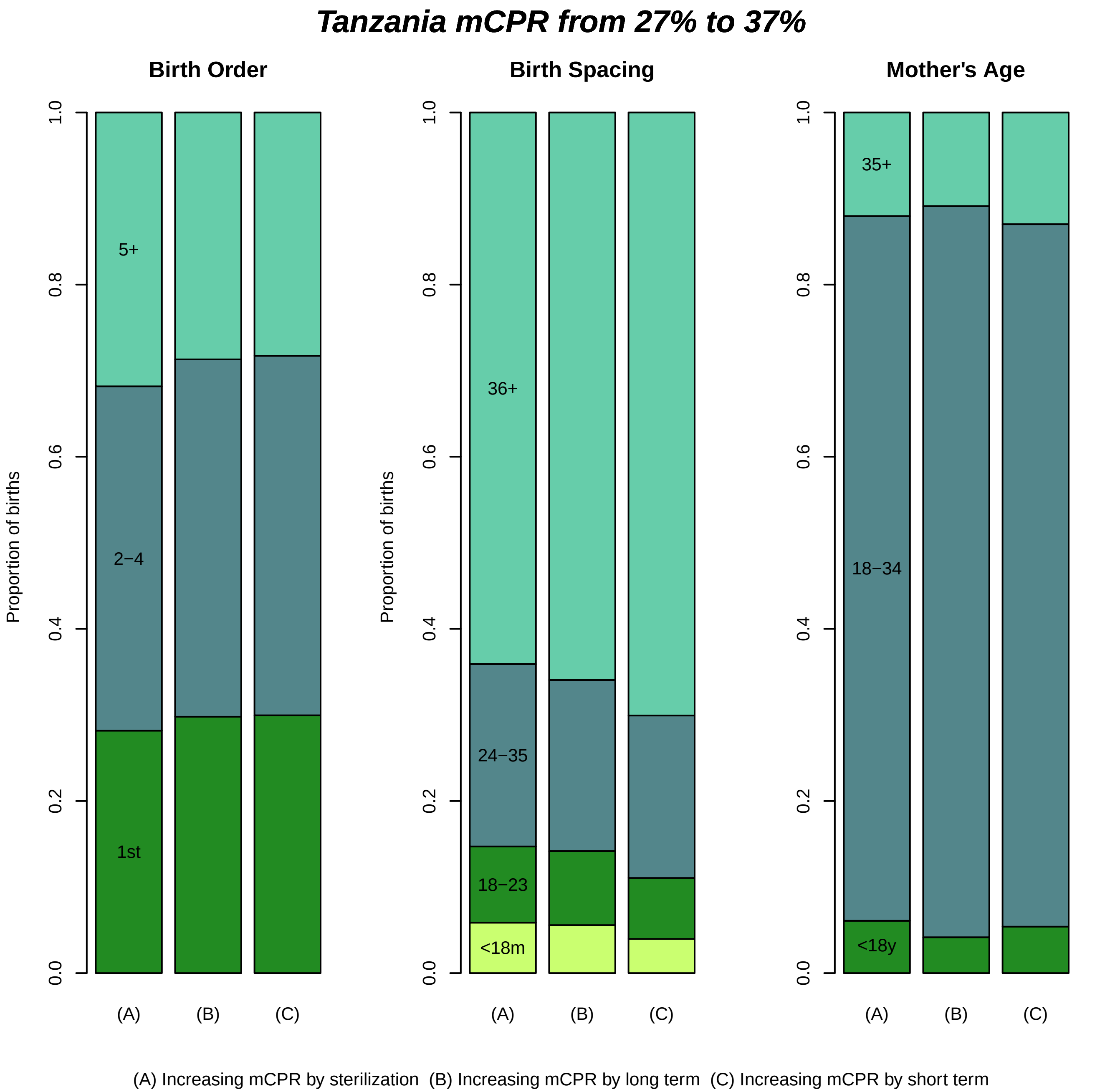

Tanzania mCPR from 27% to 37%
Birth Order
Birth Spacing
Mother's Age
Proportion of births
0.0
0.2
0.4
0.6
0.8
1.0
5+
2−4
1st
36+
24−35
18−23
<18m
35+
18−34
<18y
(A)
(B)
(C)
(A) Increasing mCPR by sterilization (B) Increasing mCPR by long term (C) Increasing mCPR by short term

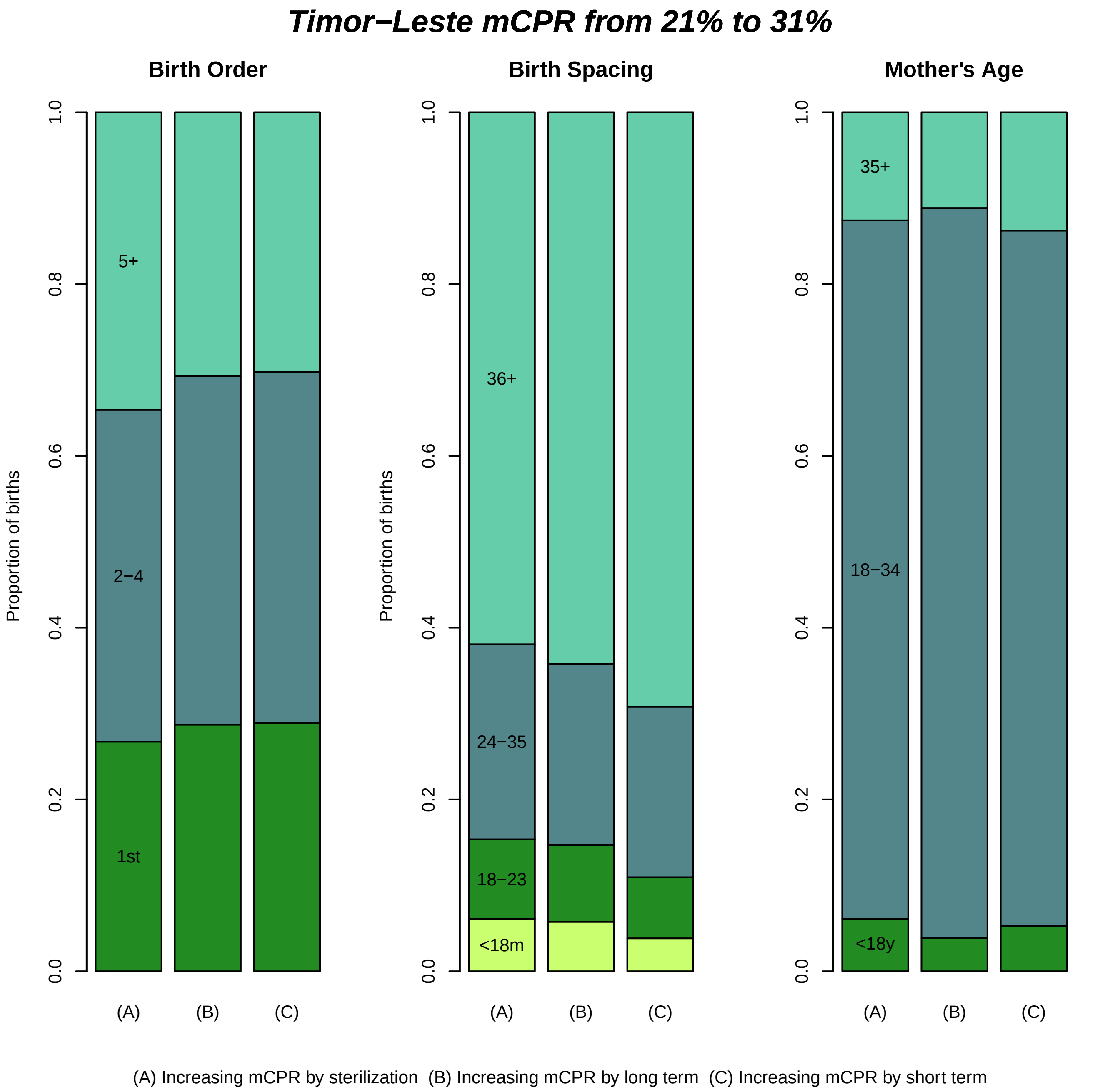

Timor−Leste mCPR from 21% to 31%
Birth Order
Birth Spacing
Mother's Age
Proportion of births
1.0
0.8
0.6
0.4
0.2
0.0
5+
2−4
1st
36+
24−35
18−23
<18m
35+
18−34
<18y
(A)
(B)
(C)
(A) Increasing mCPR by sterilization (B) Increasing mCPR by long term (C) Increasing mCPR by short term

Togo mCPR from 7% to 17%

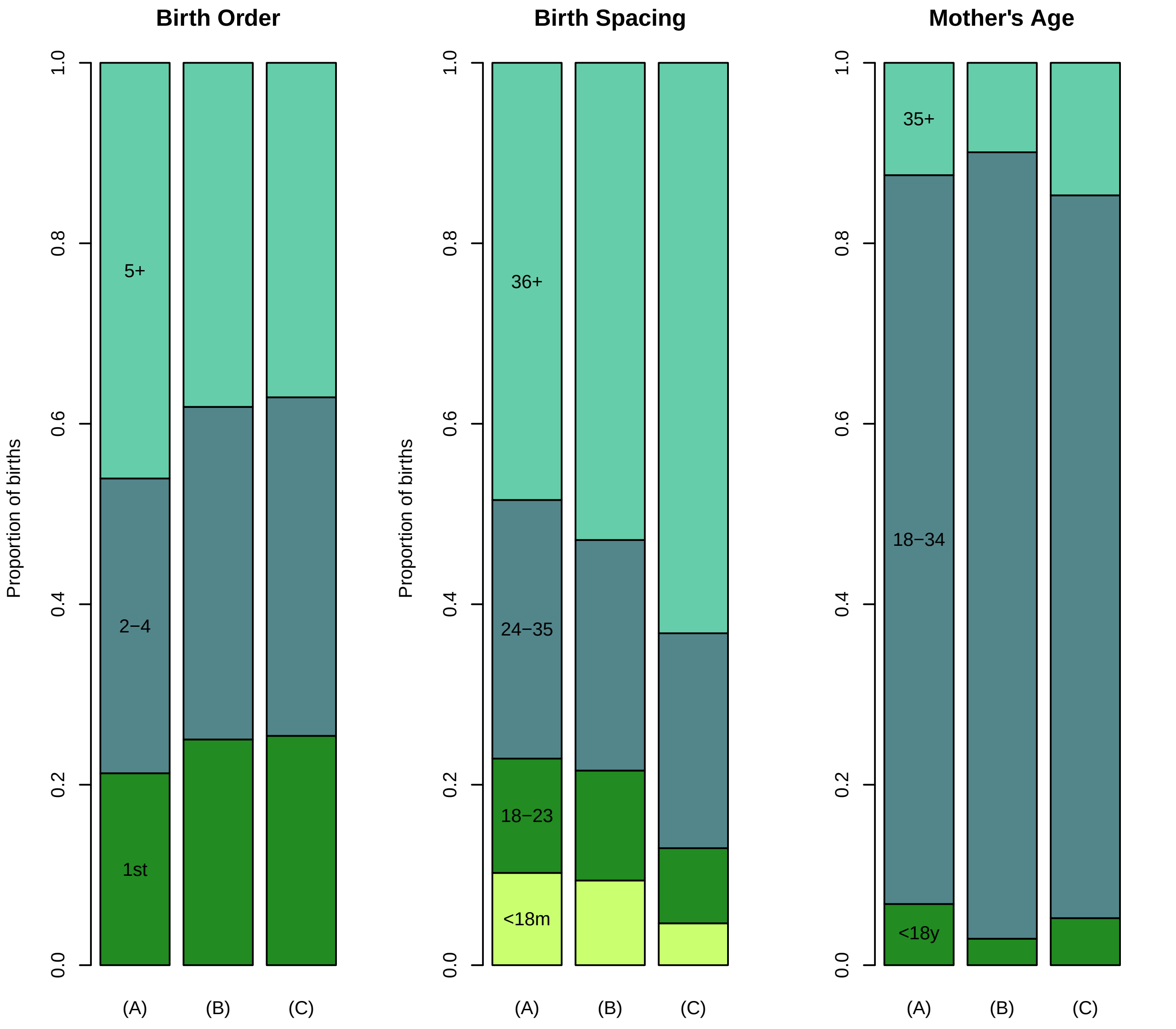

(A) Increasing mCPR by sterilization (B) Increasing mCPR by long term (C) Increasing mCPR by short term

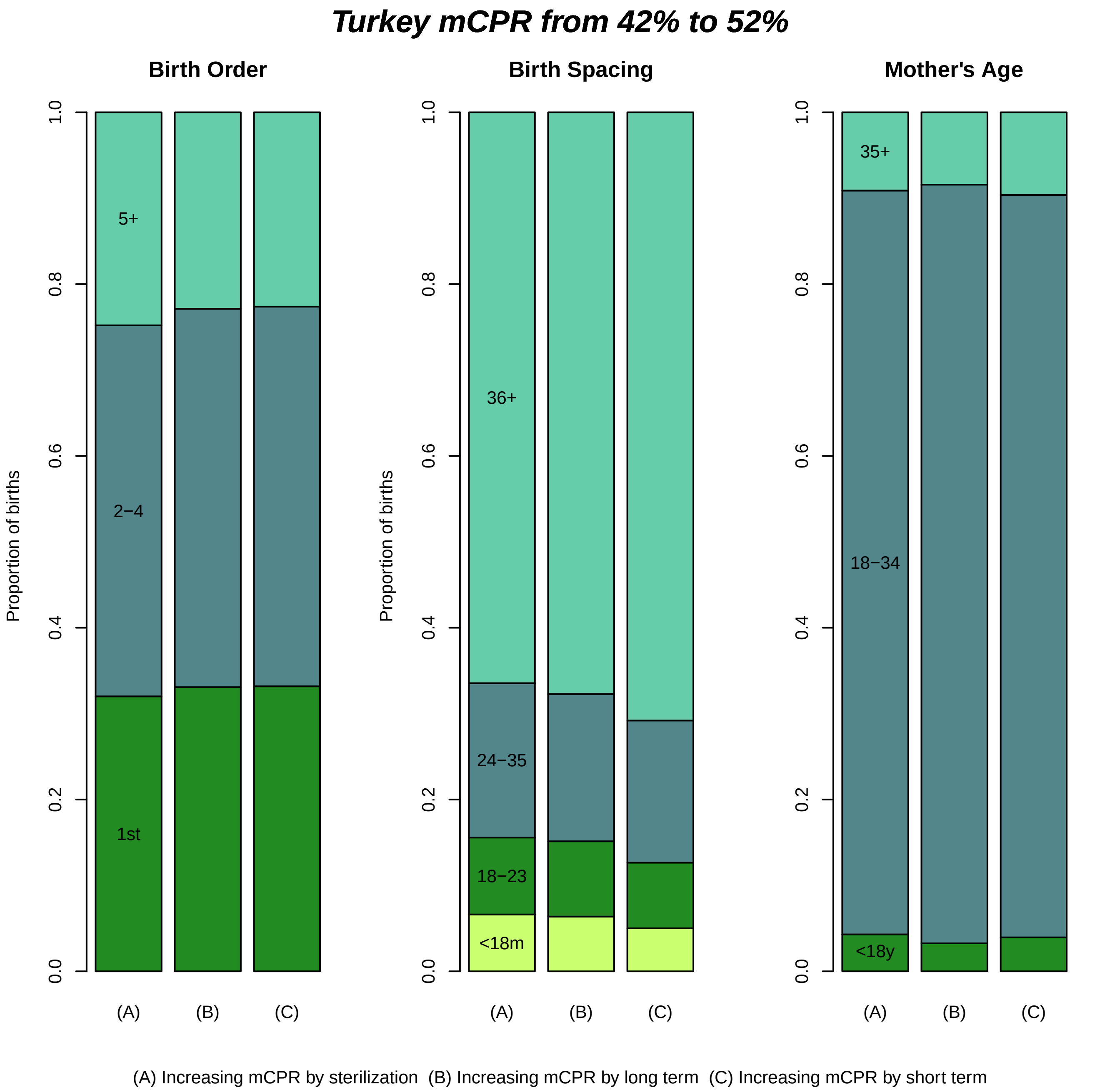

Turkey mCPR from 42% to 52%
Birth Order
Birth Spacing
Mother's Age
Proportion of births
0.0
0.2
0.4
0.6
0.8
1.0
5+
2−4
1st
36+
24−35
18−23
<18m
35+
18−34
<18y
(A)
(B)
(C)
(A) Increasing mCPR by sterilization (B) Increasing mCPR by long term (C) Increasing mCPR by short term

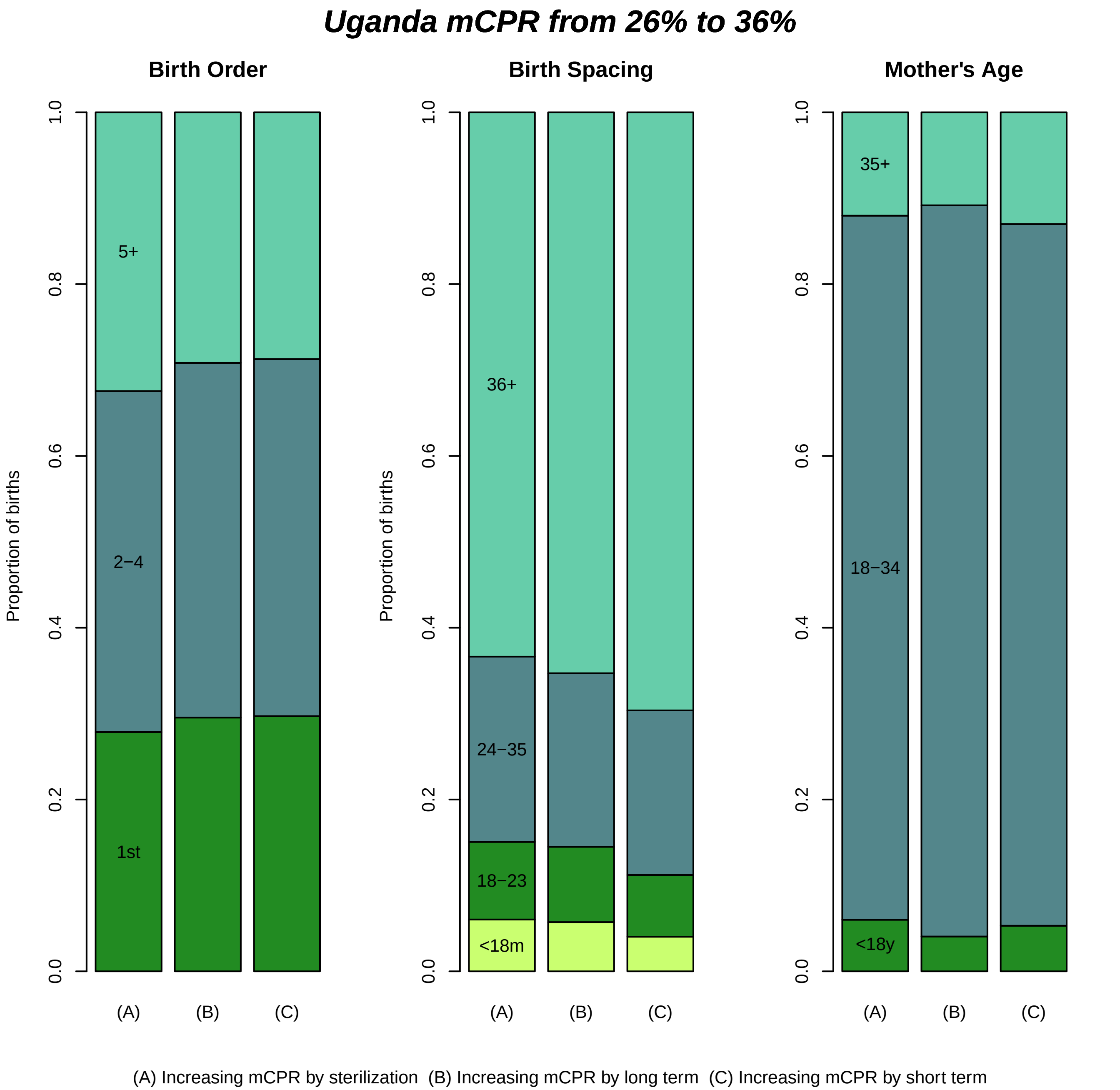
Uganda mCPR from 26% to 36%
Birth Order
Birth Spacing
Mother's Age
Proportion of births
0.0
0.2
0.4
0.6
0.8
1.0
5+
2−4
1st
36+
24−35
18−23
<18m
35+
18−34
<18y
(A)
(B)
(C)
(A) Increasing mCPR by sterilization (B) Increasing mCPR by long term (C) Increasing mCPR by short term

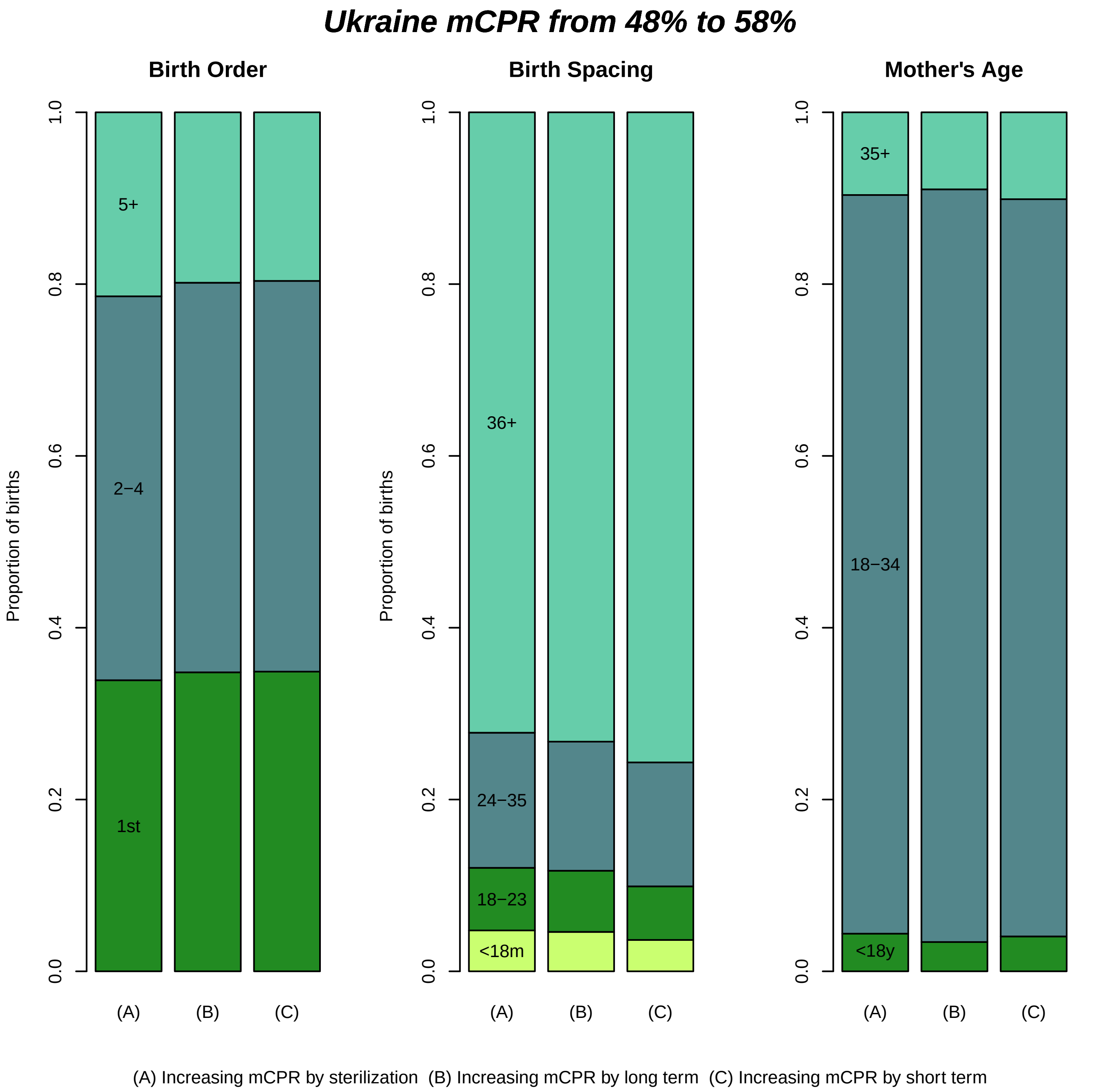
Ukraine mCPR from 48% to 58%
Birth Order
Birth Spacing
Mother's Age
Proportion of births
0.0
0.2
0.4
0.6
0.8
1.0
5+
2−4
1st
36+
24−35
18−23
<18m
35+
18−34
<18y
(A)
(B)
(C)
(A) Increasing mCPR by sterilization (B) Increasing mCPR by long term (C) Increasing mCPR by short term

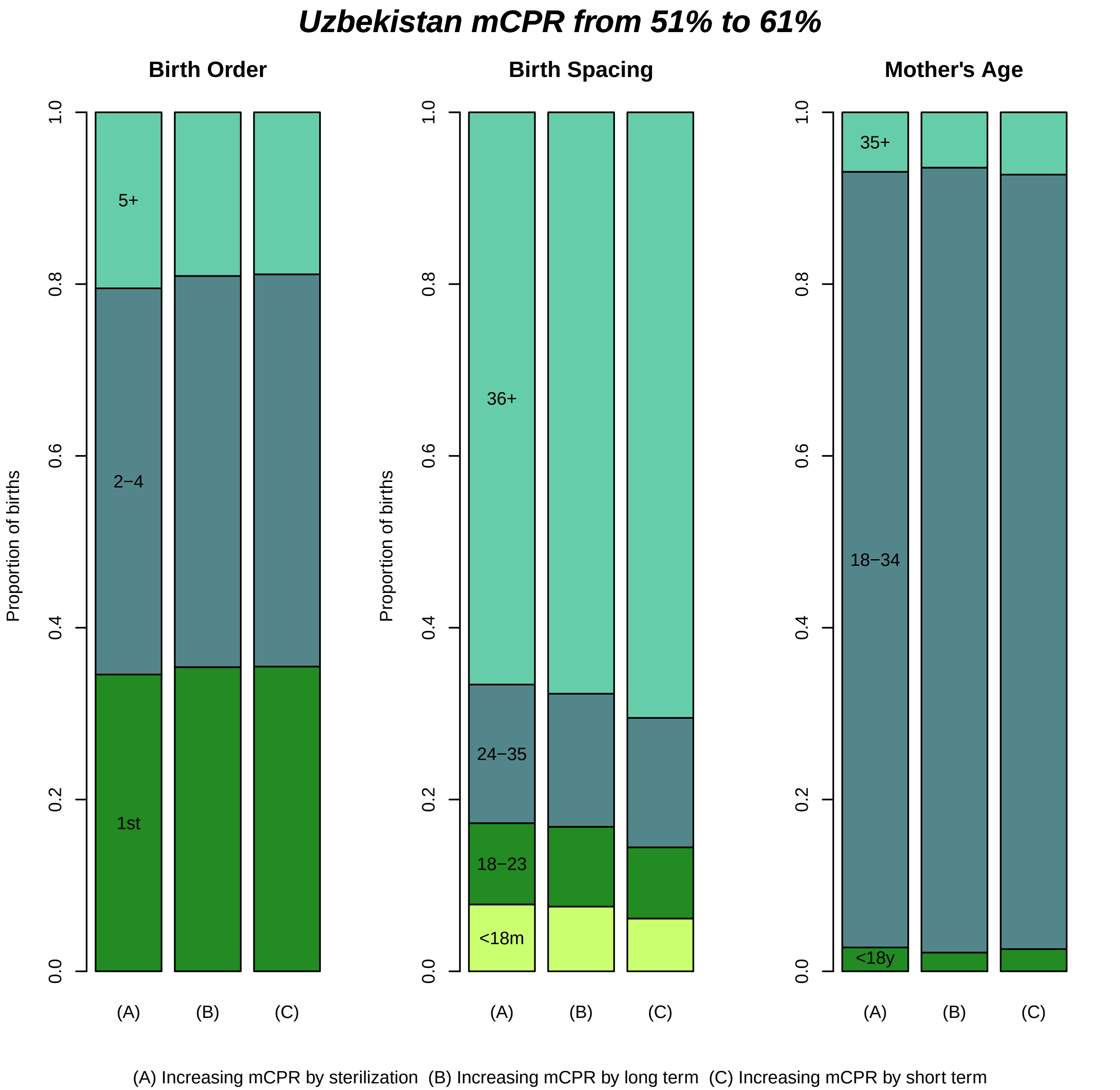

Uzbekistan mCPR from 51% to 61%
Birth Order
Birth Spacing
Mother's Age
Proportion of births
0.0
0.2
0.4
0.6
0.8
1.0
5+
2−4
1st
36+
24−35
18−23
<18m
35+
18−34
<18y
(A)
(B)
(C)
(A) Increasing mCPR by sterilization (B) Increasing mCPR by long term (C) Increasing mCPR by short term

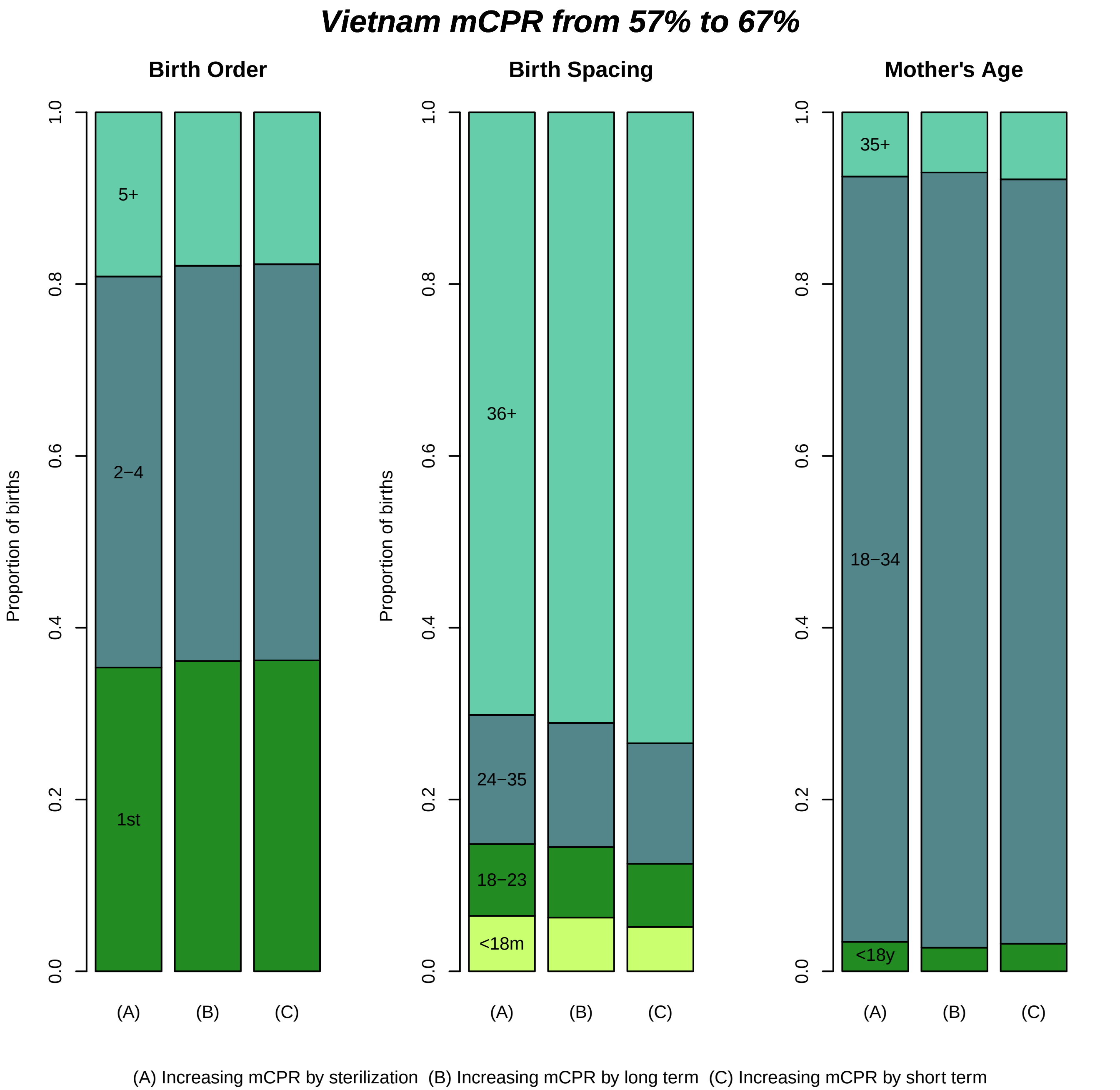

Vietnam mCPR from 57% to 67%
Birth Order
Birth Spacing
Mother's Age
Proportion of births
0.0
0.2
0.4
0.6
0.8
1.0
5+
2−4
1st
36+
24−35
18−23
<18m
35+
18−34
<18y
(A)
(B)
(C)
(A) Increasing mCPR by sterilization (B) Increasing mCPR by long term (C) Increasing mCPR by short term

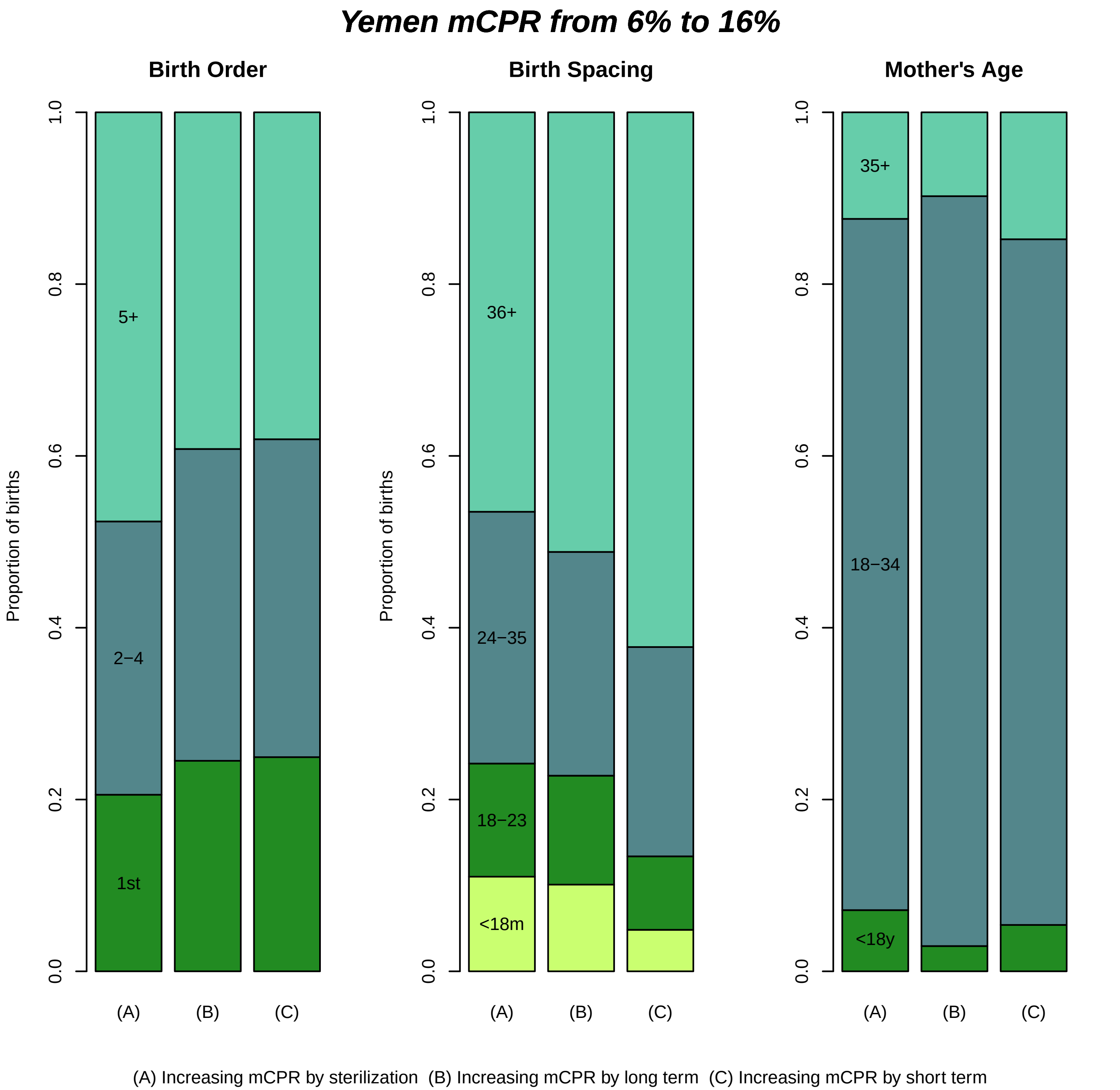
Yemen mCPR from 6% to 16%
Birth Order
Birth Spacing
Mother's Age
Proportion of births
Proportion of births
0.0
0.2
0.4
0.6
0.8
1.0
5+
2−4
1st
36+
24−35
18−23
<18m
35+
18−34
<18y
(A)
(B)
(C)
(A) Increasing mCPR by sterilization (B) Increasing mCPR by long term (C) Increasing mCPR by short term

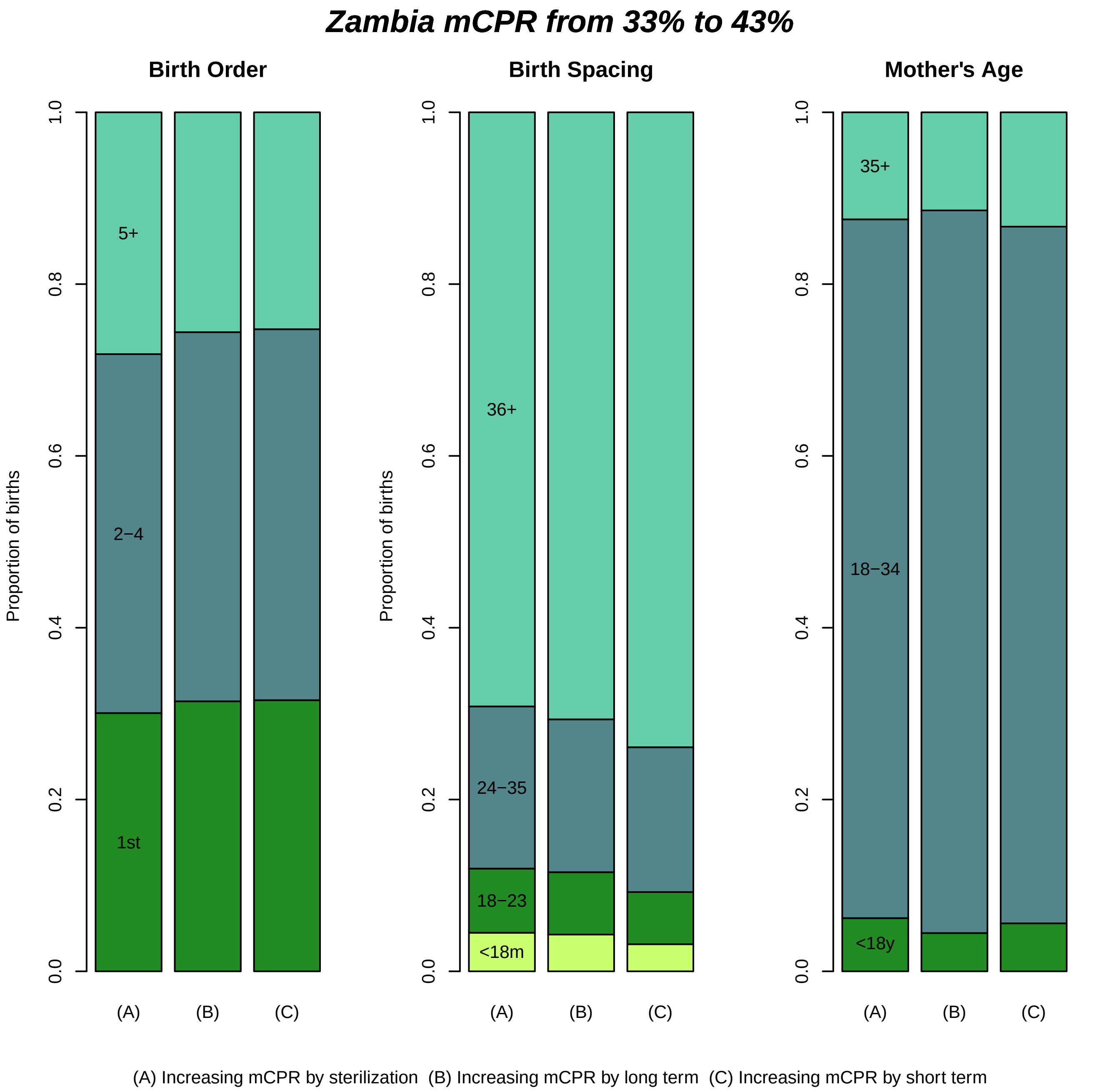
Zambia mCPR from 33% to 43%
Birth Order
Birth Spacing
Mother's Age
Proportion of births
Proportion of births
0.0
0.2
0.4
0.6
0.8
1.0
5+
2−4
1st
36+
24−35
18−23
<18m
35+
18−34
<18y
(A)
(B)
(C)
(A) Increasing mCPR by sterilization (B) Increasing mCPR by long term (C) Increasing mCPR by short term

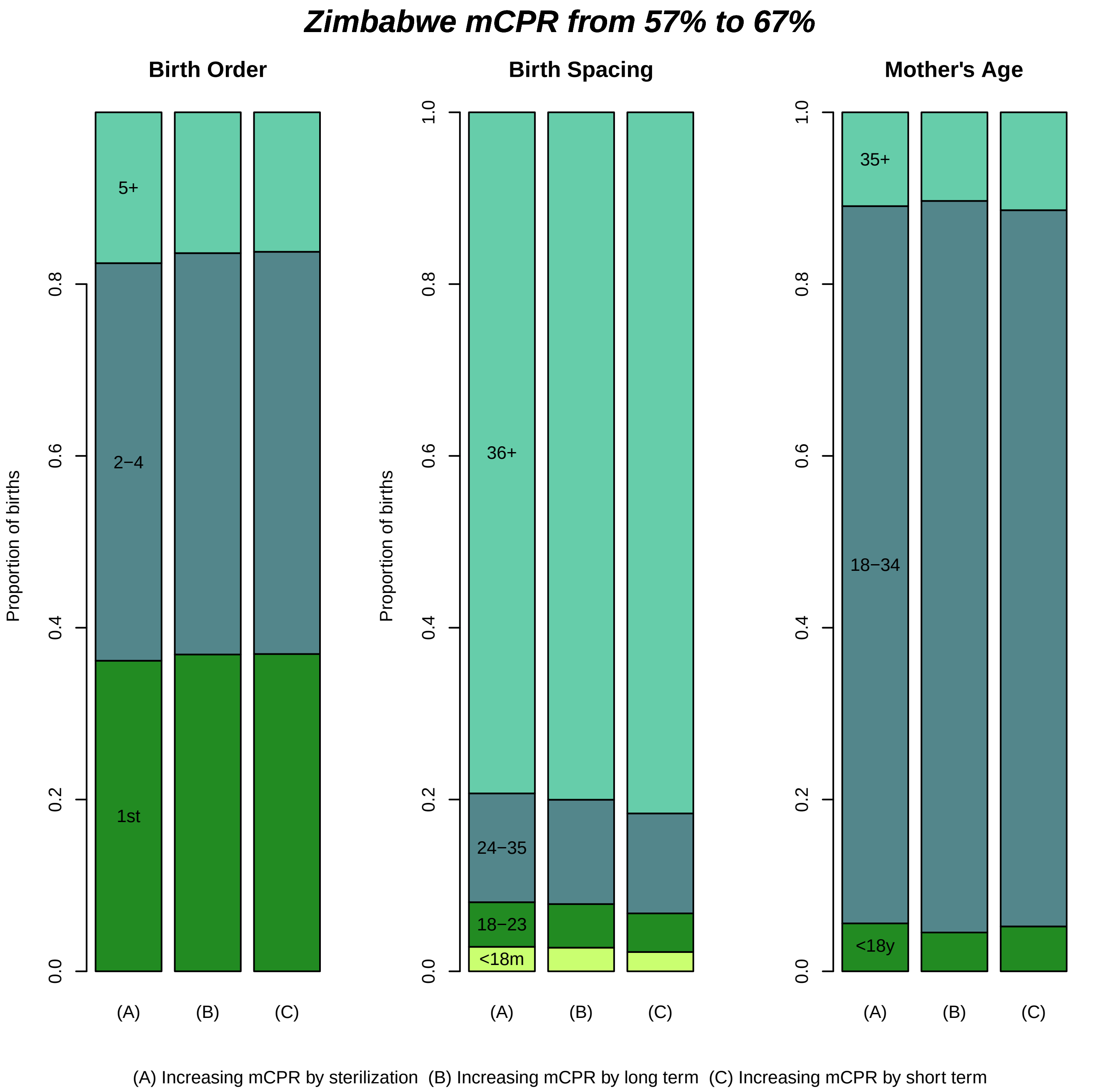

Zimbabwe mCPR from 57% to 67%
Birth Order
Birth Spacing
Mother's Age
Proportion of births
0.0
0.2
0.4
0.6
0.8
1.0
5+
2−4
1st
36+
24−35
18−23
<18m
35+
18−34
<18y
(A)
(B)
(C)
(A) Increasing mCPR by sterilization (B) Increasing mCPR by long term (C) Increasing mCPR by short term
