# Supplementary material for: Predicting high risk births with contraceptive prevalence and contraceptive method-mix in an ecologic analysis
Source: BMC Public Health. 2017 Nov 7;17(Suppl 4):786. doi: 10.1186/s12889-017-4741-6 (PMC5688497; doi:10.1186/s12889-017-4741-6)
Supplement: Supplementary file 3 — Additional predictions for how birth spacing will change with mCPR ranging between 20 and 80% for similar scenarios are shown for 71 countries. (PDF 1962 kb) [file 12889_2017_4741_MOESM3_ESM.pdf]

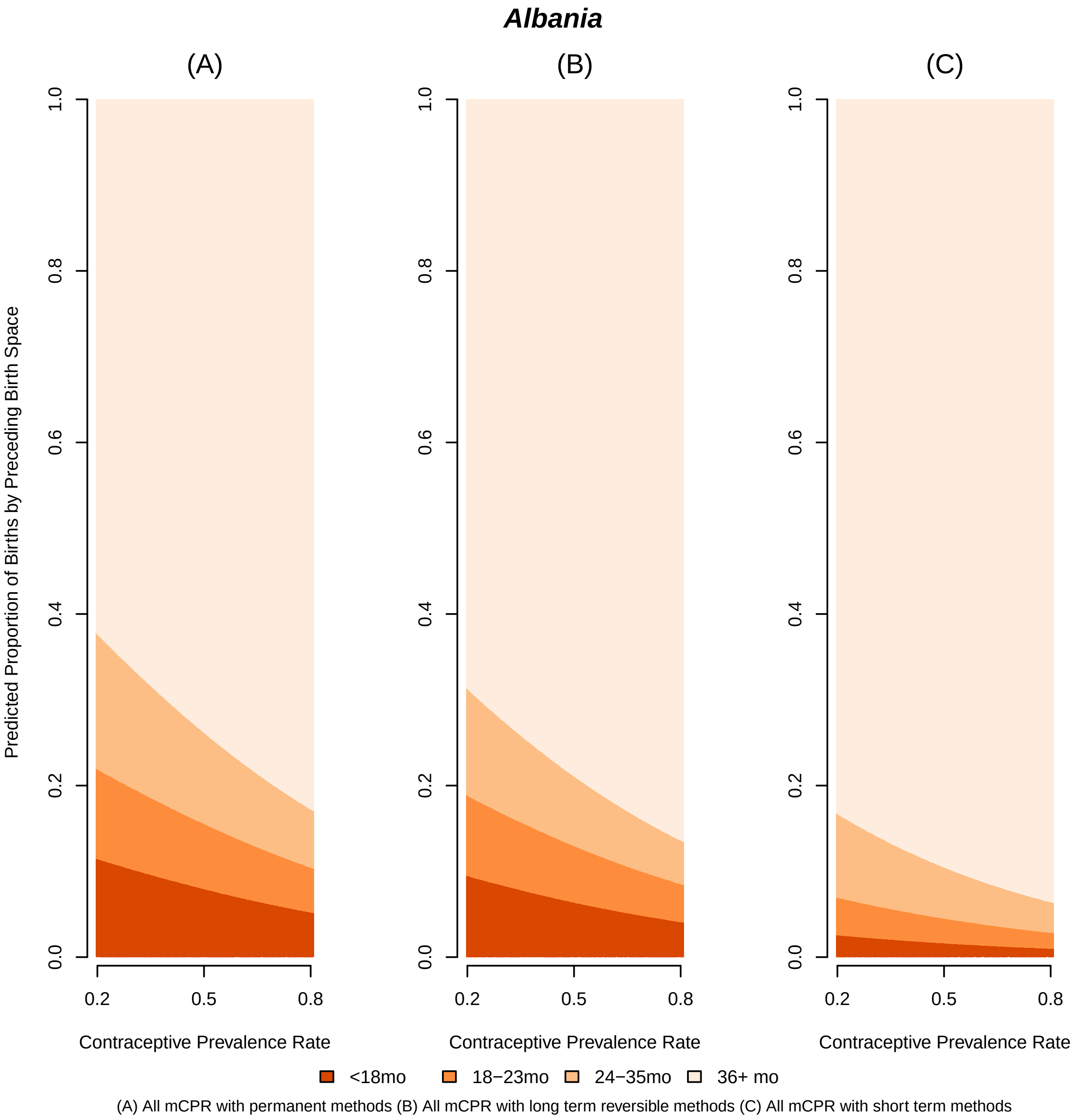
Albania
(A)
(B)
(C)
Predicted Proportion of Births by Preceding Birth Space
0.0
0.2
0.4
0.6
0.8
1.0
0.2
0.5
0.8
Contraceptive Prevalence Rate
<18mo
18−23mo
24−35mo
36+ mo
(A) All mCPR with permanent methods (B) All mCPR with long term reversible methods (C) All mCPR with short term methods

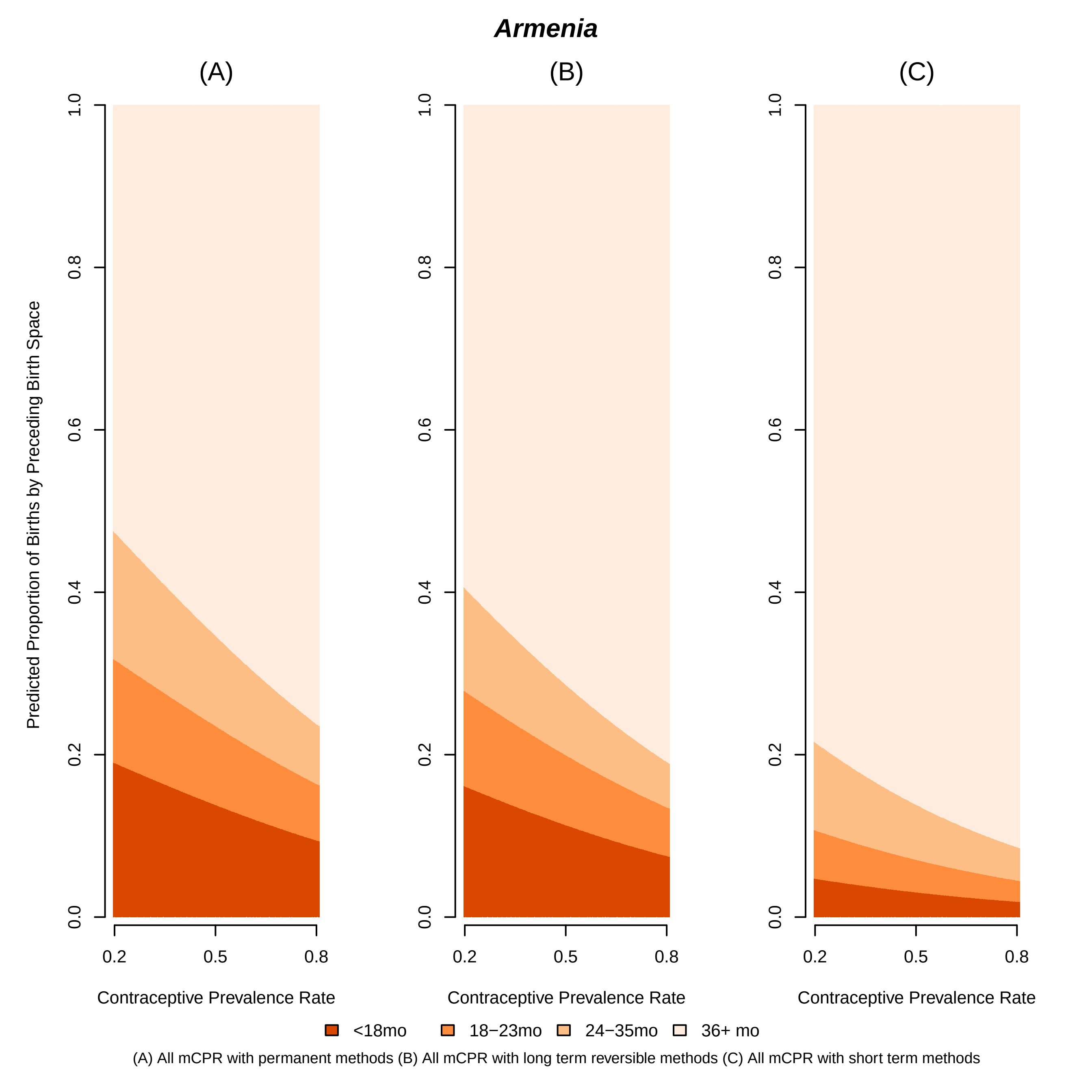
Armenia
(A)
(B)
(C)
Predicted Proportion of Births by Preceding Birth Space
0.0
0.2
0.4
0.6
0.8
1.0
0.2
0.5
0.8
Contraceptive Prevalence Rate
<18mo
18−23mo
24−35mo
36+ mo
(A) All mCPR with permanent methods (B) All mCPR with long term reversible methods (C) All mCPR with short term methods

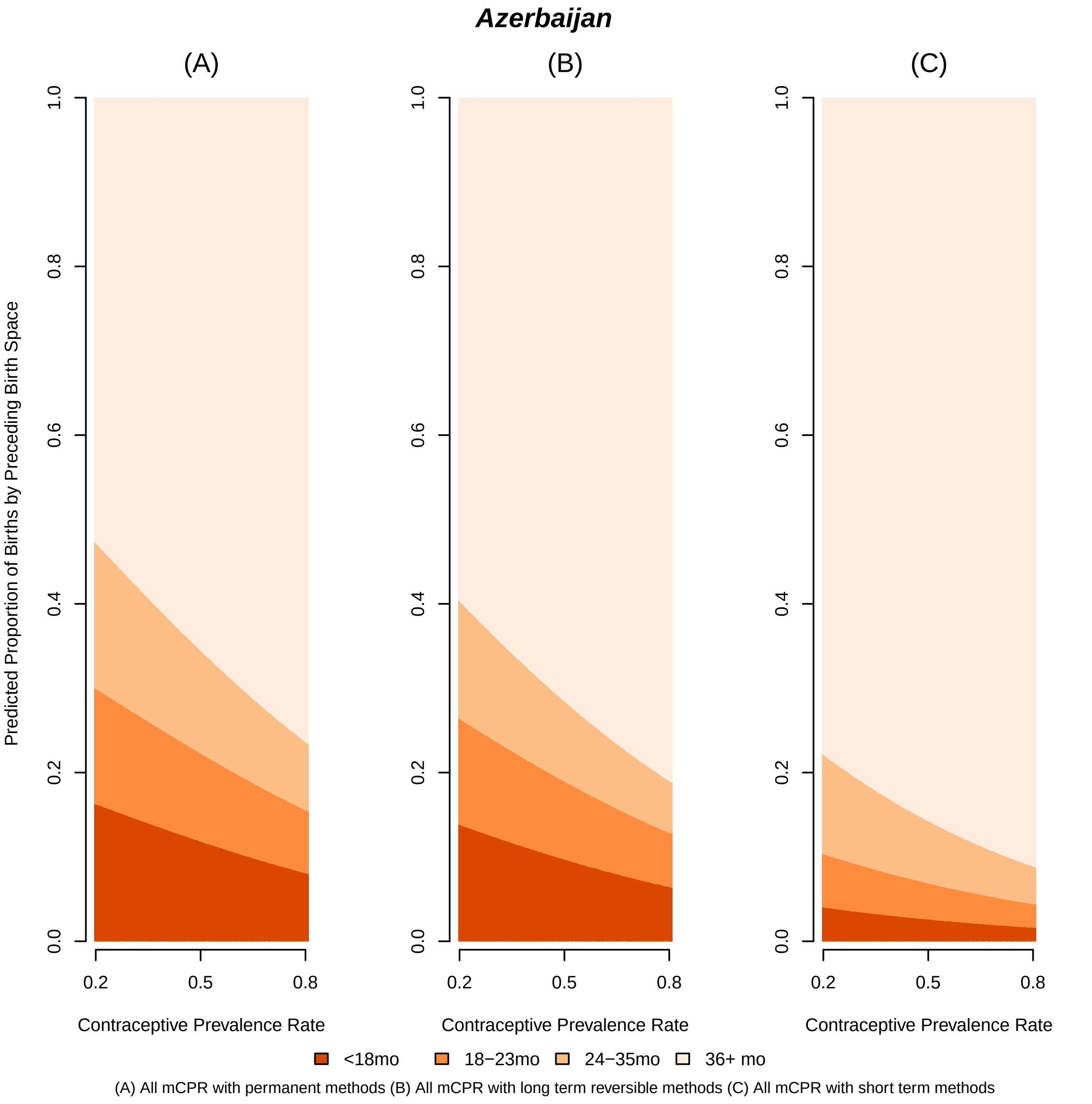
Azerbaijan
(A)
(B)
(C)
Predicted Proportion of Births by Preceding Birth Space
0.0
0.2
0.4
0.6
0.8
1.0
0.2
0.5
0.8
Contraceptive Prevalence Rate
<18mo
18−23mo
24−35mo
36+ mo
(A) All mCPR with permanent methods (B) All mCPR with long term reversible methods (C) All mCPR with short term methods

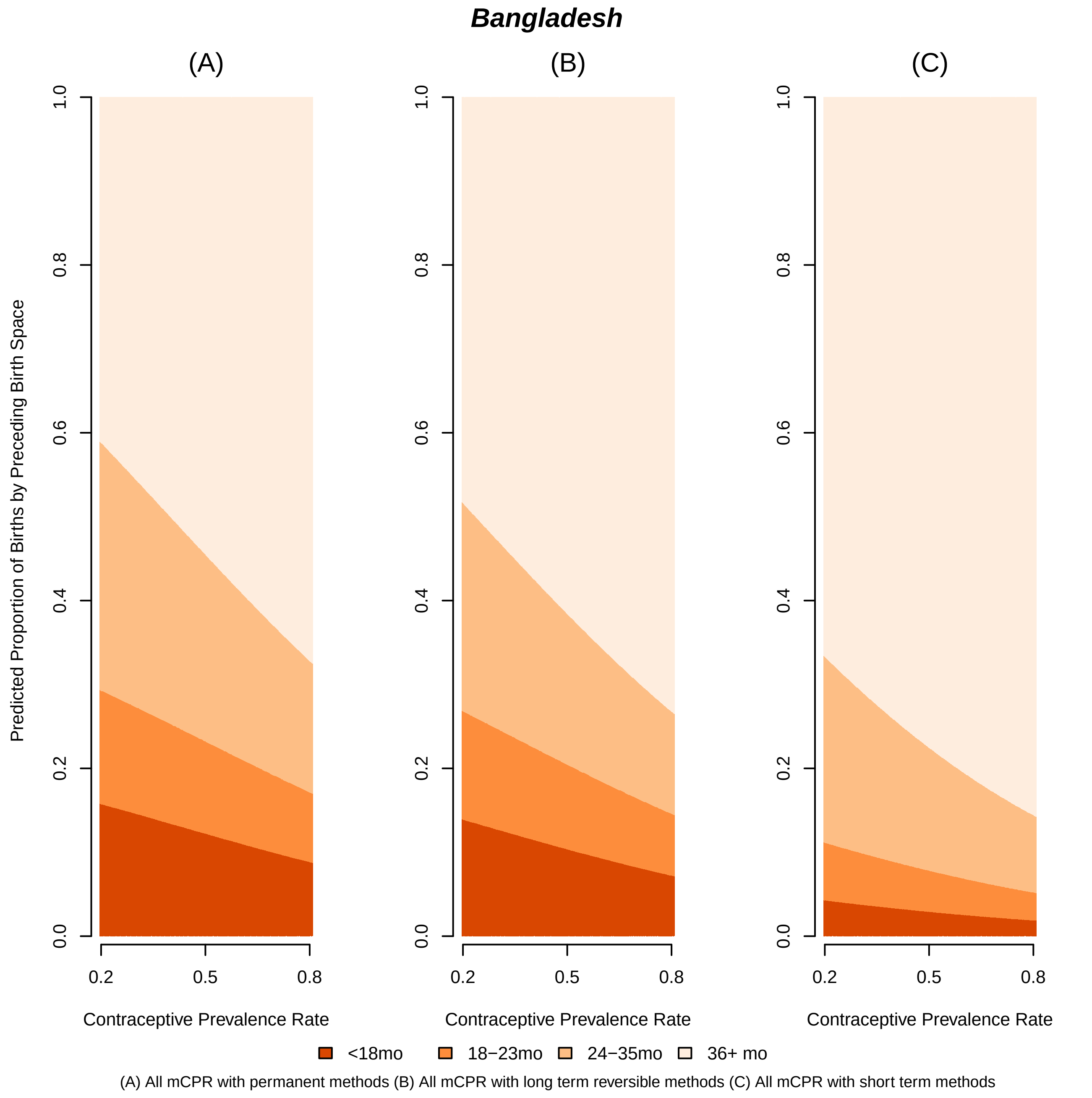

Bangladesh
(A)
(B)
(C)
Predicted Proportion of Births by Preceding Birth Space
0.0
0.2
0.4
0.6
0.8
1.0
0.2
0.5
0.8
Contraceptive Prevalence Rate
<18mo
18−23mo
24−35mo
36+ mo
(A) All mCPR with permanent methods (B) All mCPR with long term reversible methods (C) All mCPR with short term methods

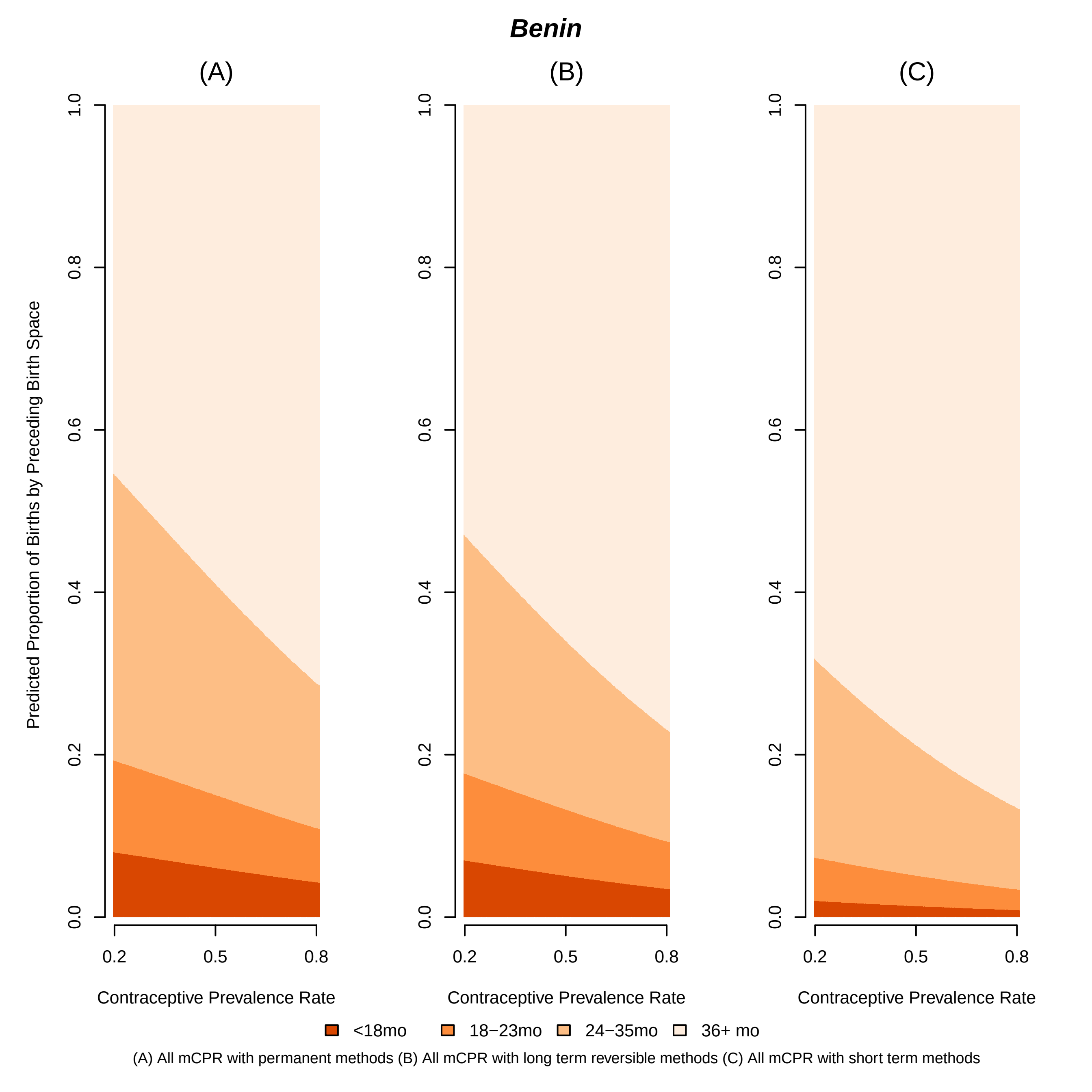
Benin
(A)
(B)
(C)
Predicted Proportion of Births by Preceding Birth Space
0.0
0.2
0.4
0.6
0.8
1.0
0.2
0.5
0.8
Contraceptive Prevalence Rate
<18mo
18−23mo
24−35mo
36+ mo
(A) All mCPR with permanent methods (B) All mCPR with long term reversible methods (C) All mCPR with short term methods

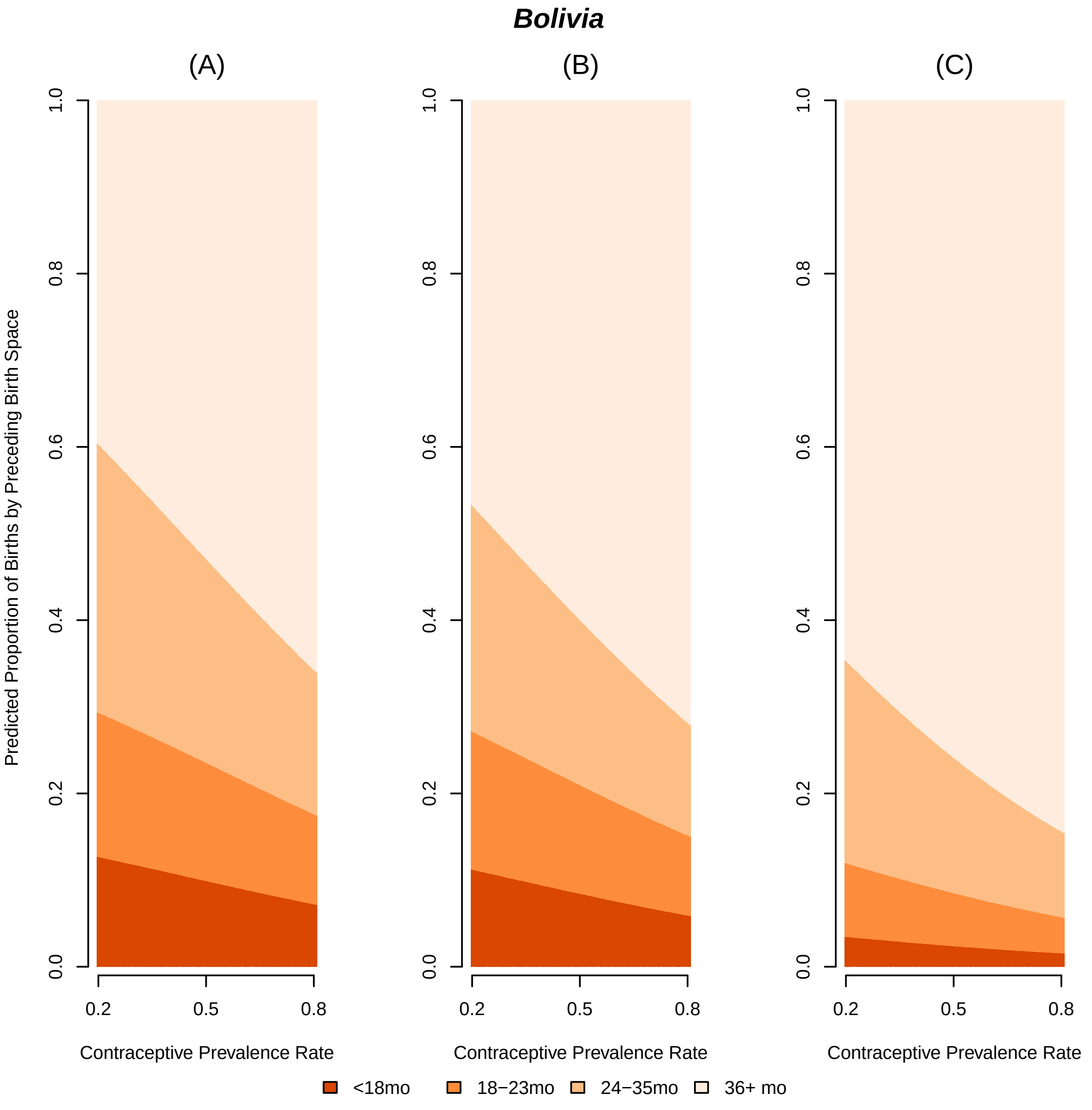

(A) All mCPR with permanent methods (B) All mCPR with long term reversible methods (C) All mCPR with short term methods

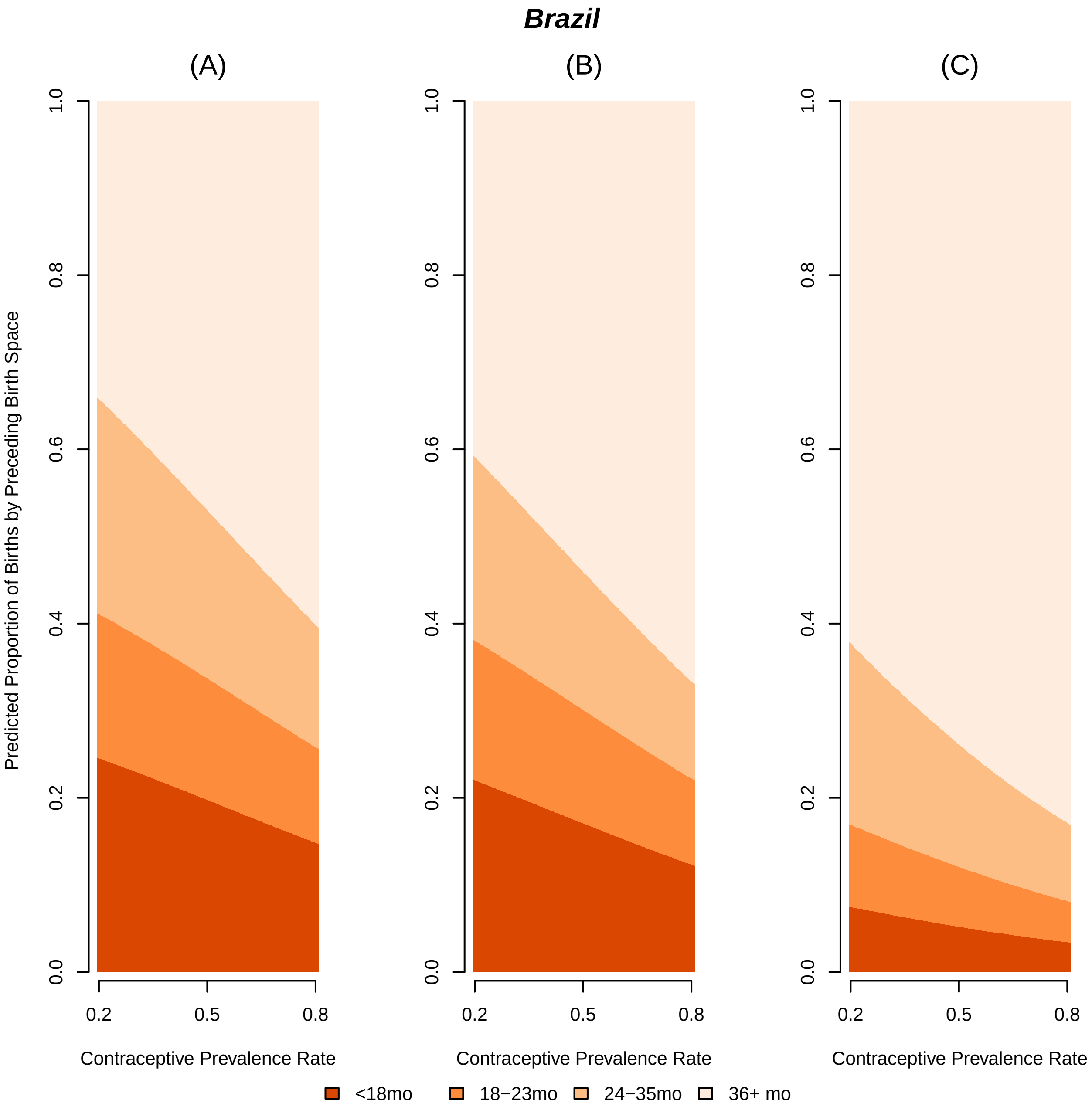

(A) All mCPR with permanent methods (B) All mCPR with long term reversible methods (C) All mCPR with short term methods

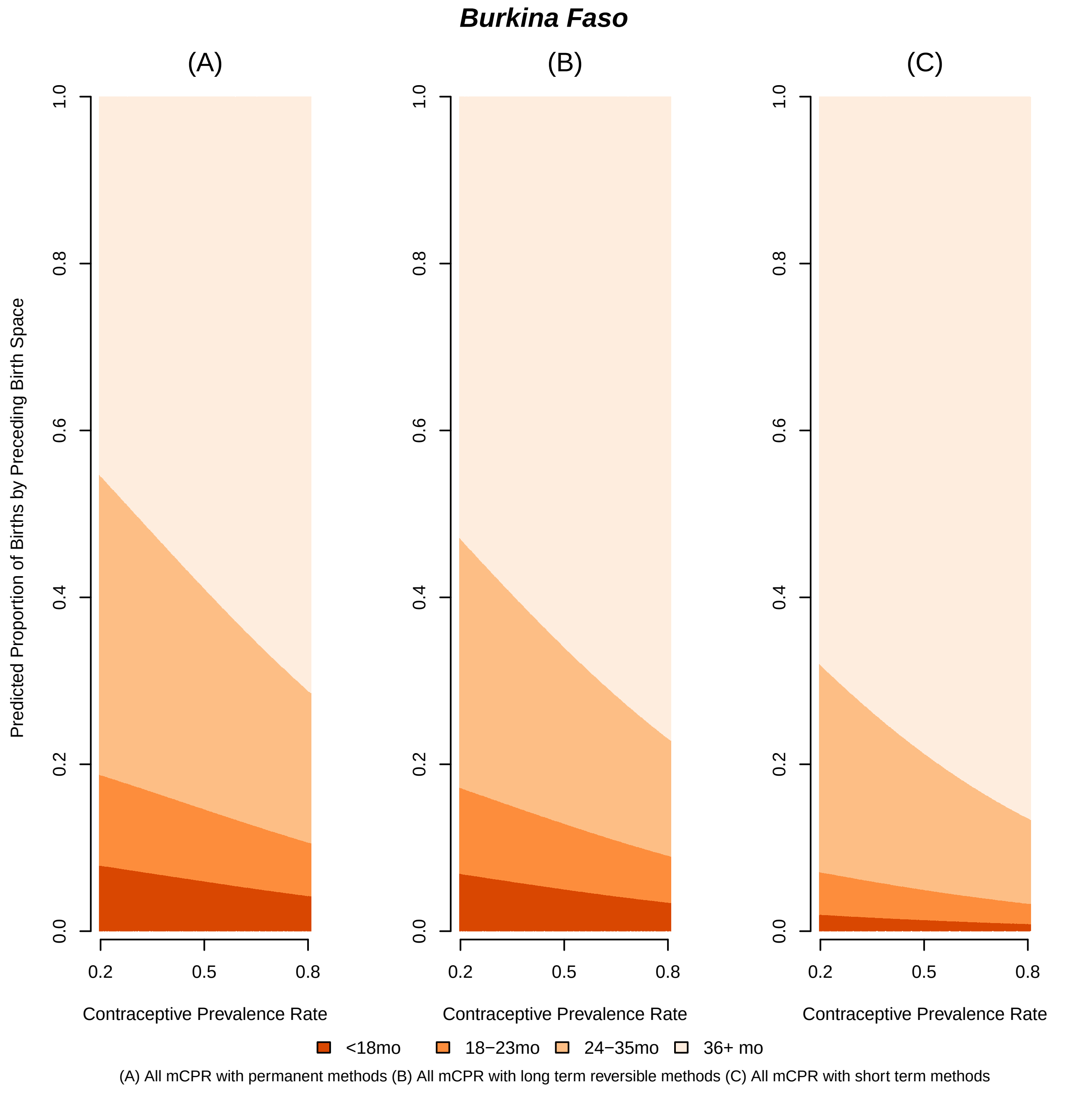
Burkina Faso
(A)
(B)
(C)
Predicted Proportion of Births by Preceding Birth Space
0.0
0.2
0.4
0.6
0.8
1.0
0.2
0.5
0.8
Contraceptive Prevalence Rate
<18mo
18−23mo
24−35mo
36+ mo
(A) All mCPR with permanent methods (B) All mCPR with long term reversible methods (C) All mCPR with short term methods

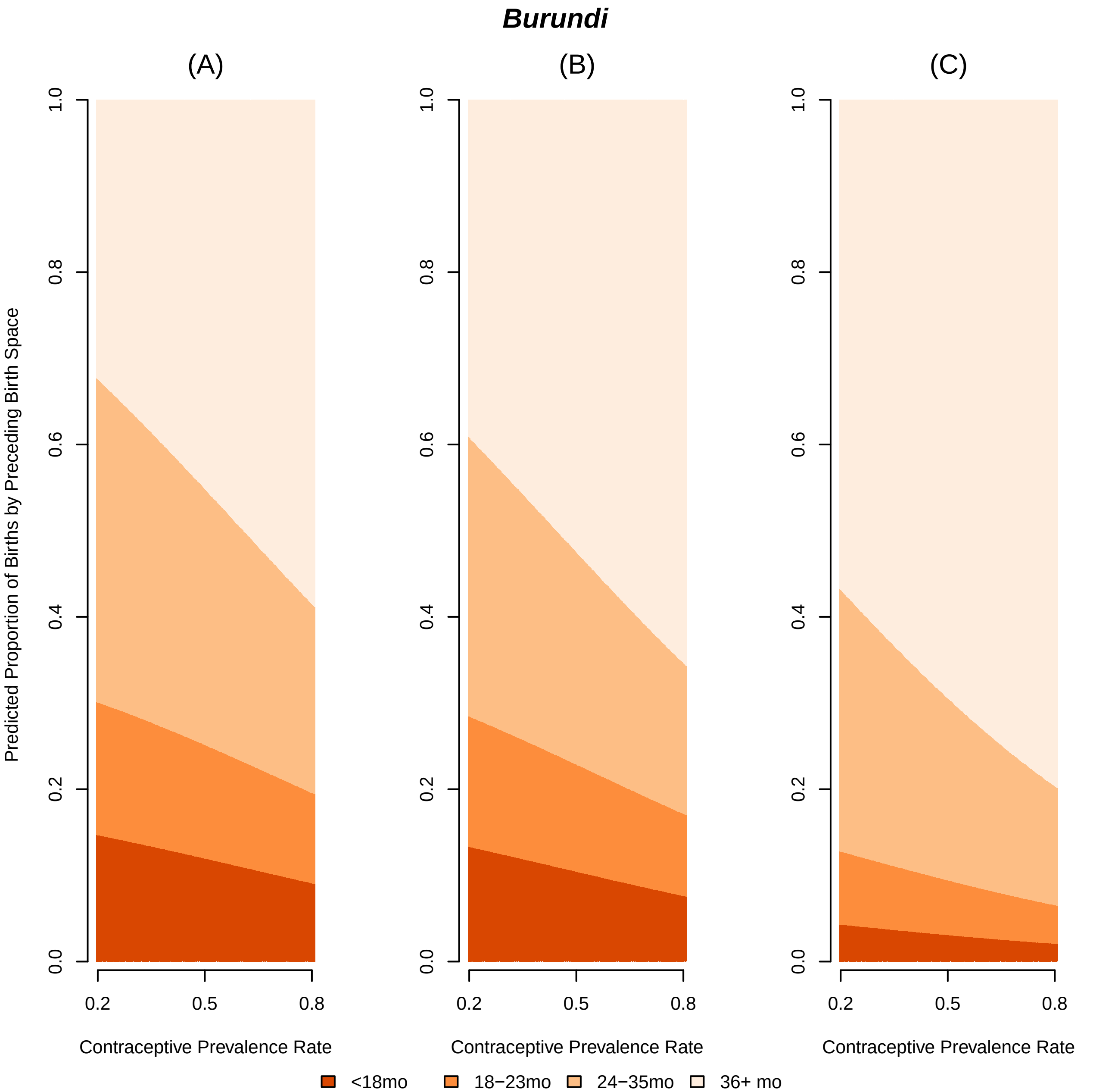

(A) All mCPR with permanent methods (B) All mCPR with long term reversible methods (C) All mCPR with short term methods

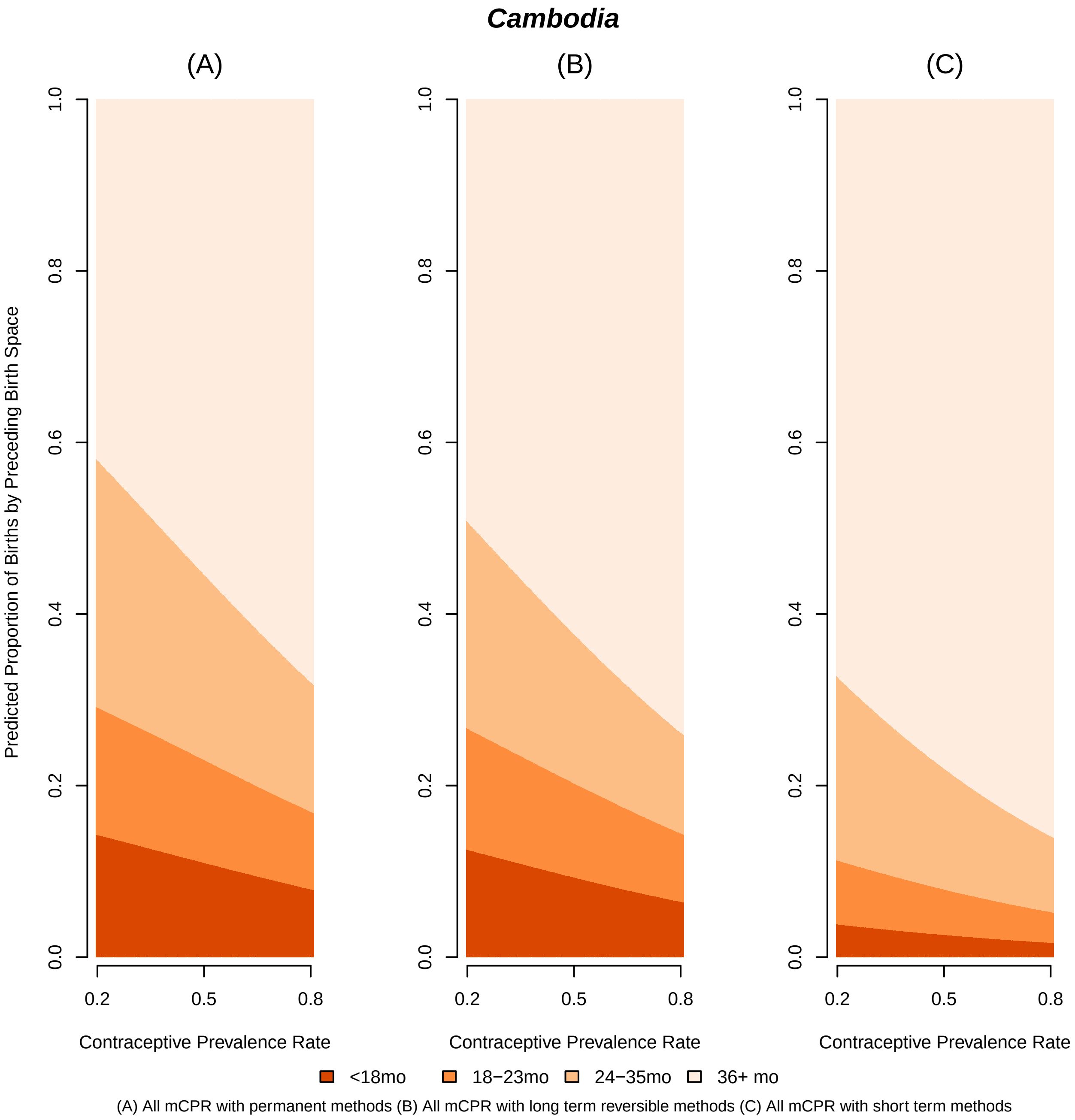
Cambodia
(A)
(B)
(C)
Predicted Proportion of Births by Preceding Birth Space
0.0
0.2
0.4
0.6
0.8
1.0
0.2
0.5
0.8
Contraceptive Prevalence Rate
<18mo
18−23mo
24−35mo
36+ mo
(A) All mCPR with permanent methods (B) All mCPR with long term reversible methods (C) All mCPR with short term methods

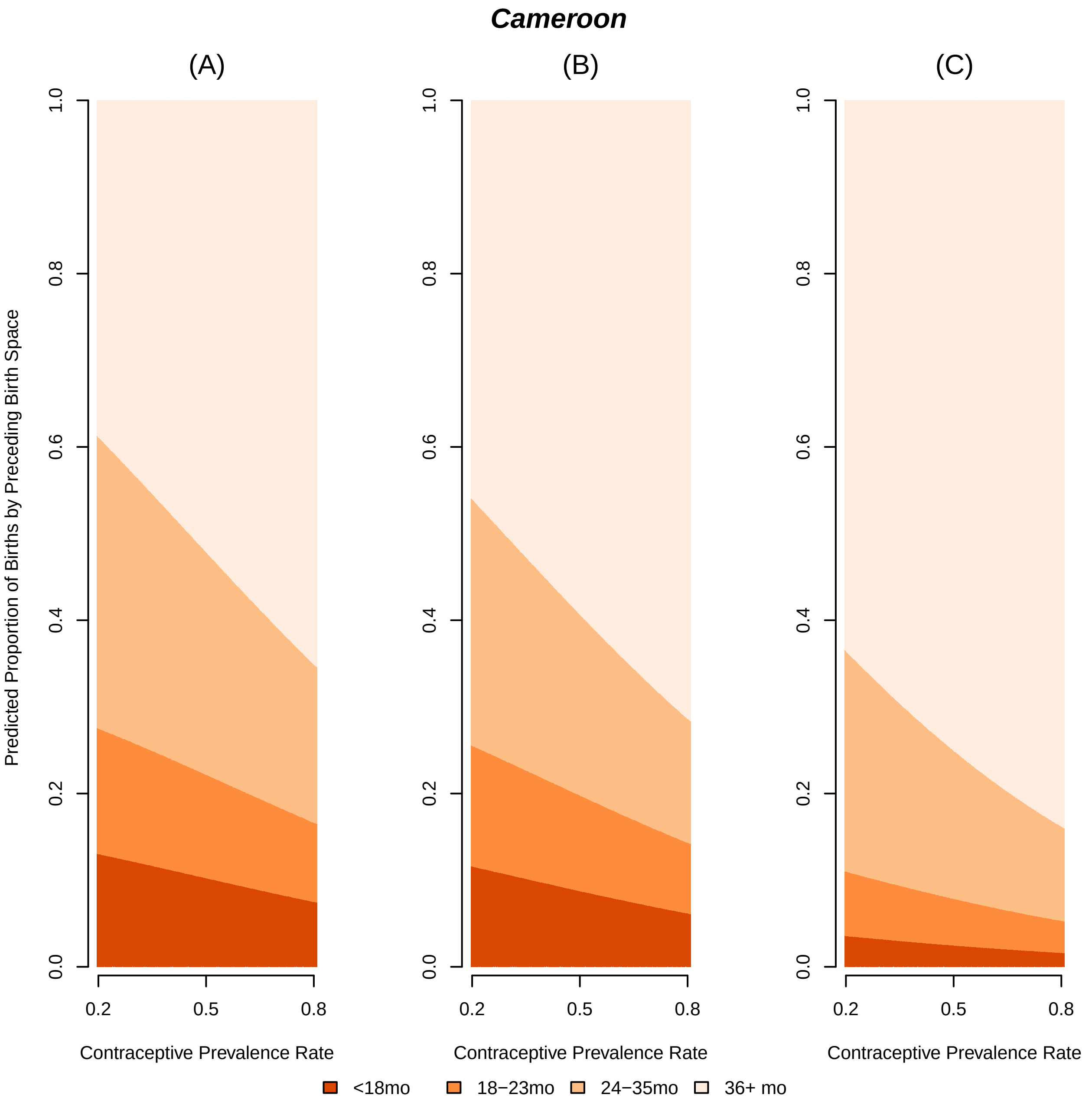

(A) All mCPR with permanent methods (B) All mCPR with long term reversible methods (C) All mCPR with short term methods

Central African Republic

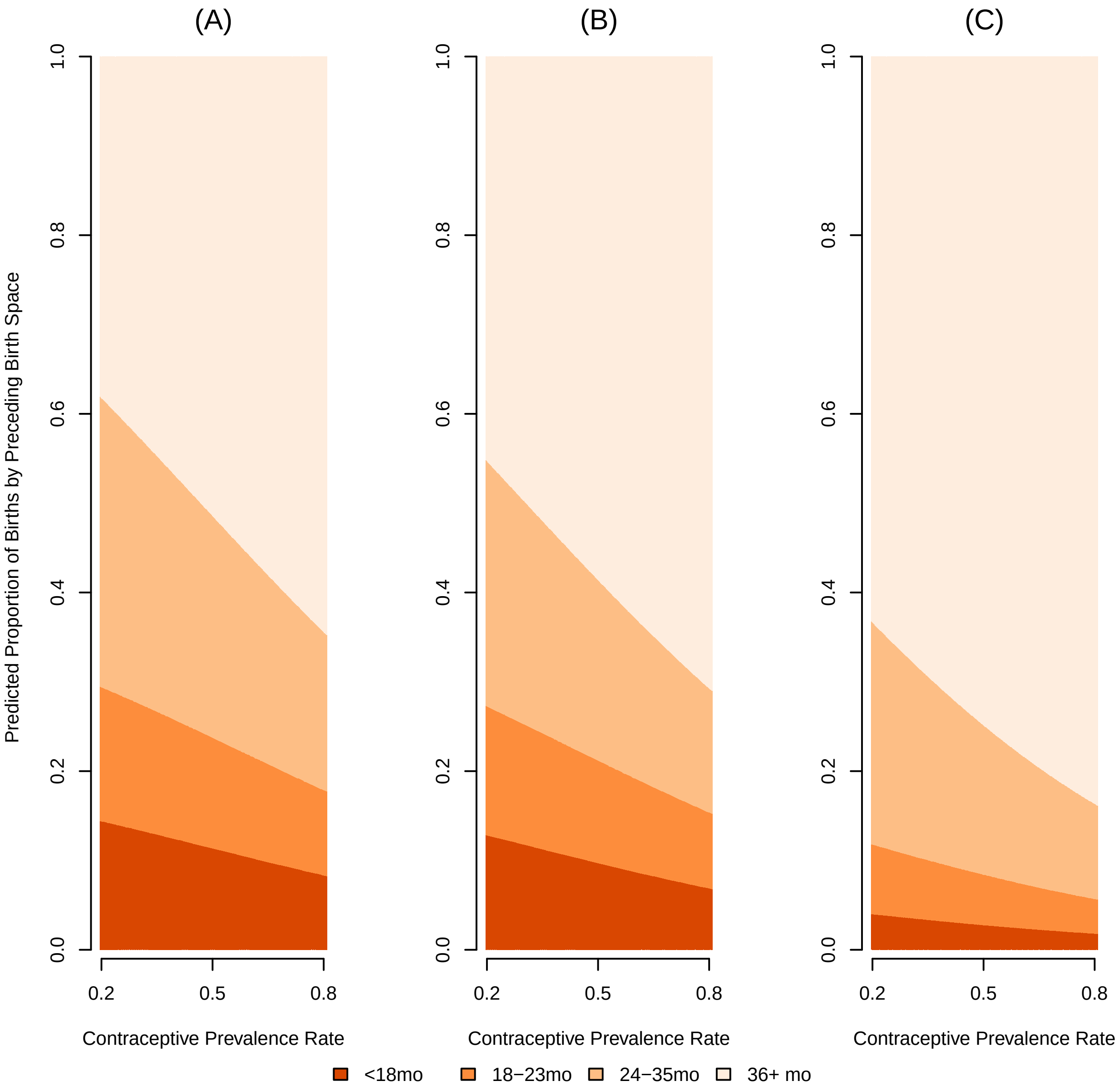

(A) All mCPR with permanent methods (B) All mCPR with long term reversible methods (C) All mCPR with short term methods

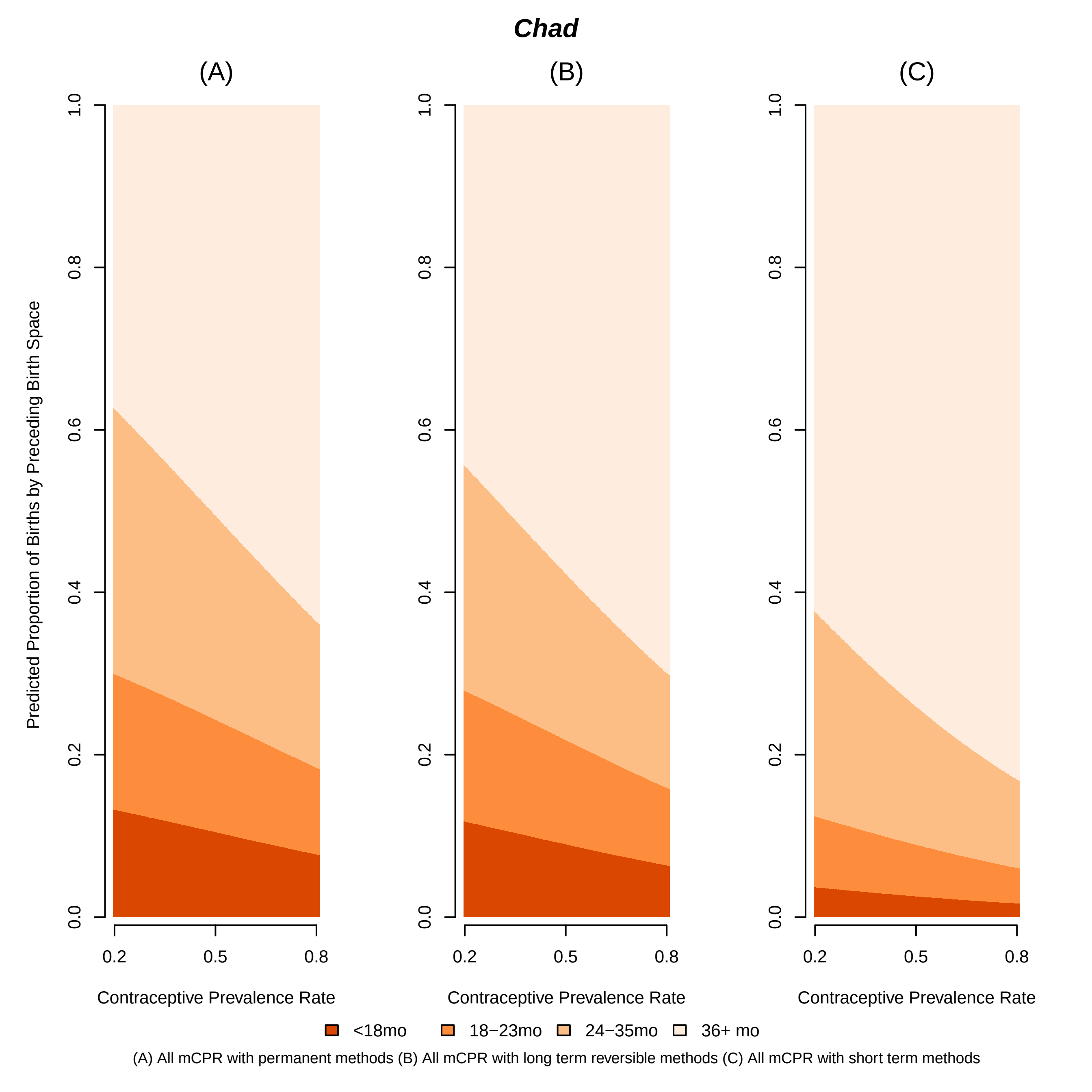
Chad
(A)
(B)
(C)
Predicted Proportion of Births by Preceding Birth Space
0.0
0.2
0.4
0.6
0.8
1.0
0.2
0.5
0.8
Contraceptive Prevalence Rate
<18mo
18−23mo
24−35mo
36+ mo
(A) All mCPR with permanent methods (B) All mCPR with long term reversible methods (C) All mCPR with short term methods

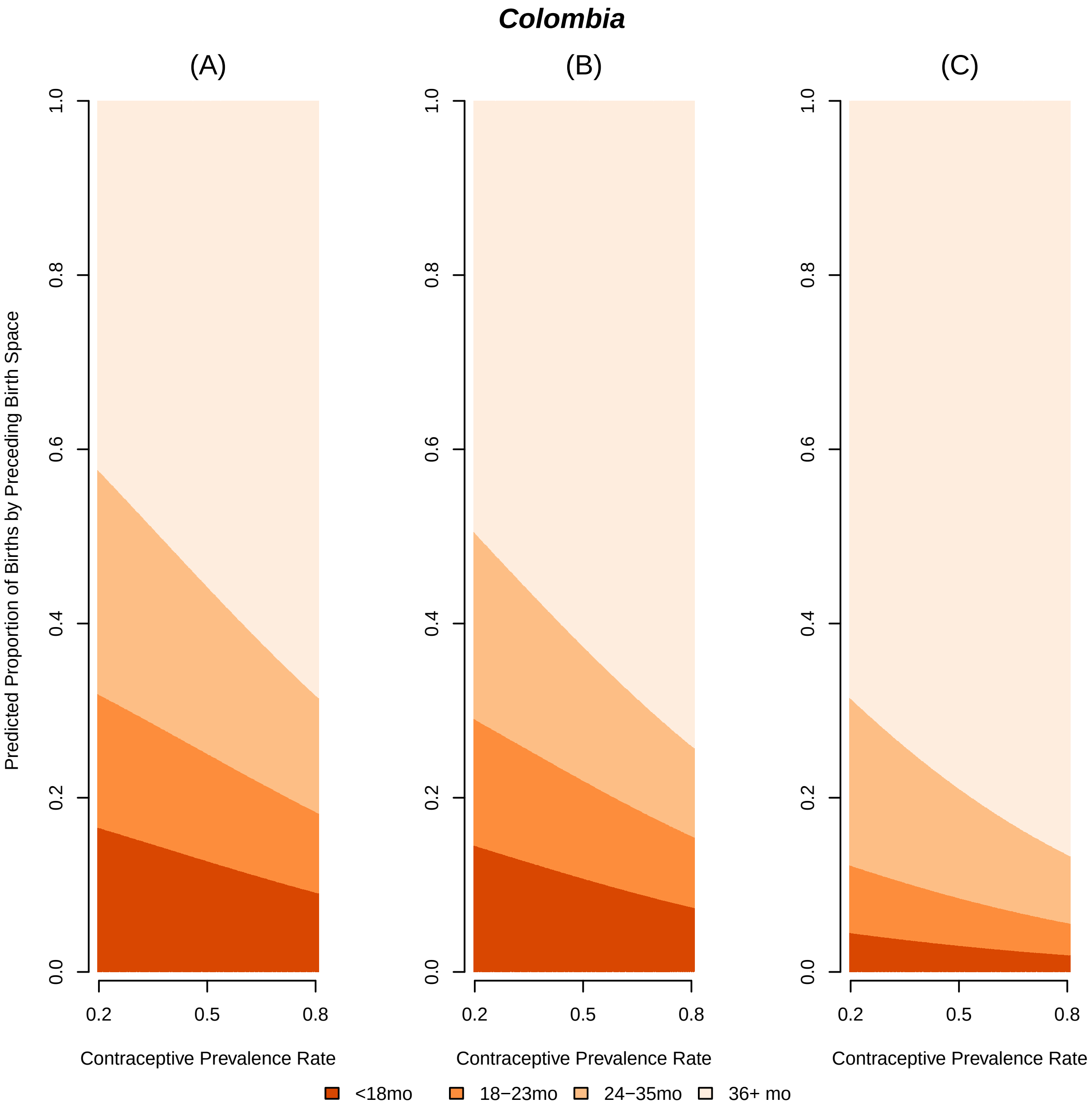

(A) All mCPR with permanent methods (B) All mCPR with long term reversible methods (C) All mCPR with short term methods

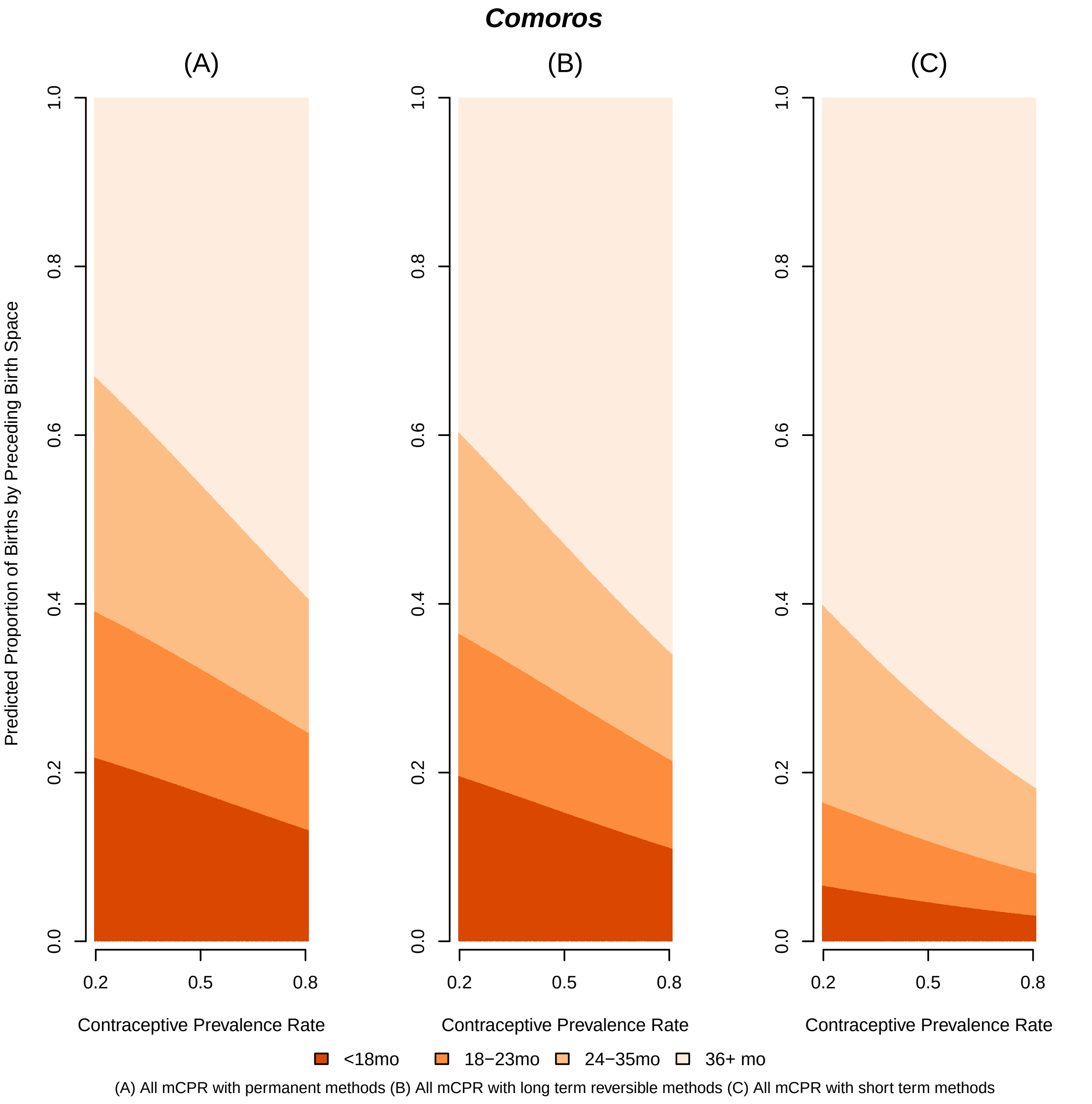
Comoros
(A)
(B)
(C)
Predicted Proportion of Births by Preceding Birth Space
0.0
0.2
0.4
0.6
0.8
1.0
0.2
0.5
0.8
Contraceptive Prevalence Rate
<18mo
18−23mo
24−35mo
36+ mo
(A) All mCPR with permanent methods (B) All mCPR with long term reversible methods (C) All mCPR with short term methods

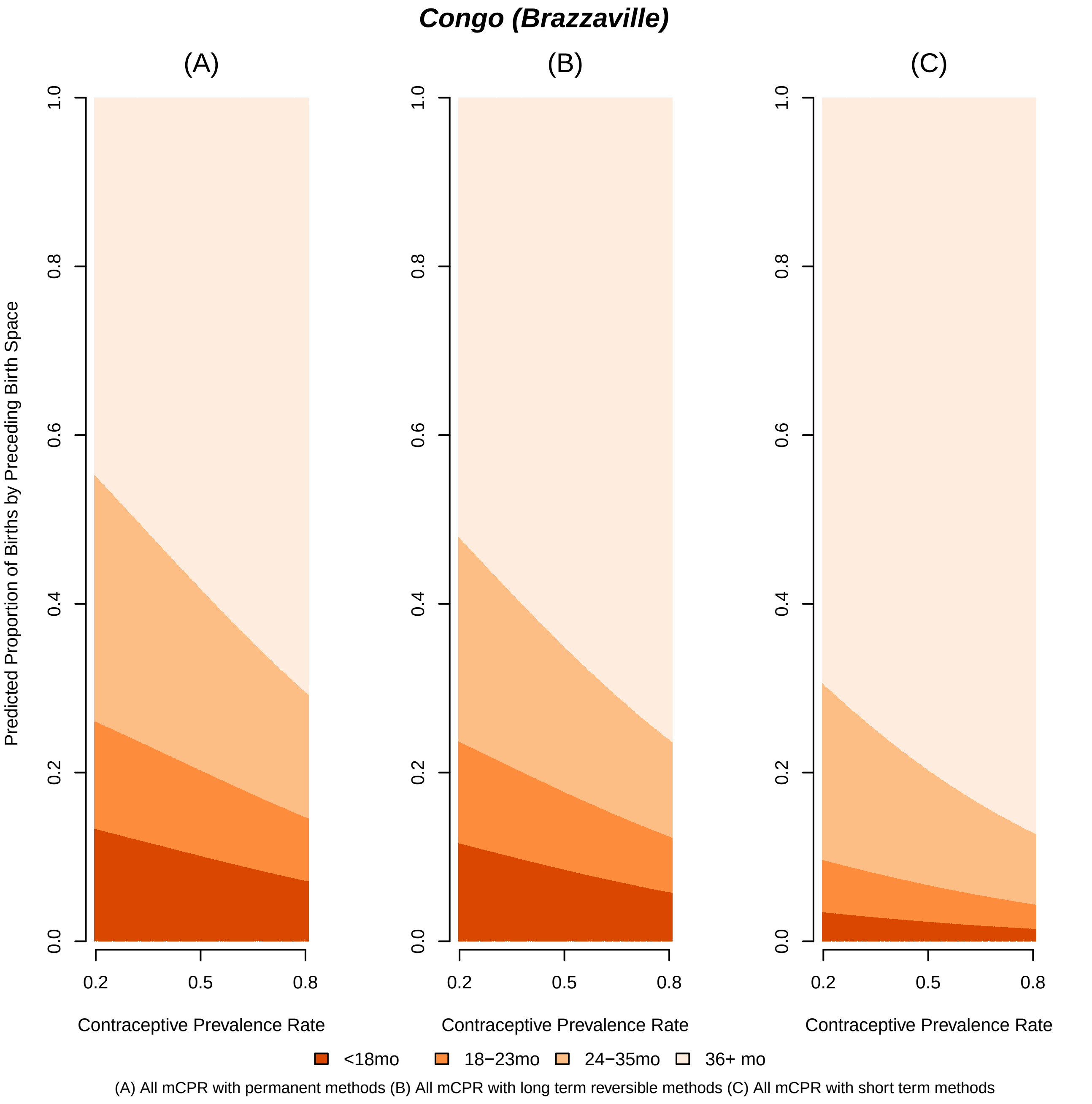

Congo (Brazzaville)
(A)
(B)
(C)
Predicted Proportion of Births by Preceding Birth Space
0.0
0.2
0.4
0.6
0.8
1.0
0.2
0.5
0.8
Contraceptive Prevalence Rate
<18mo
18−23mo
24−35mo
36+ mo
(A) All mCPR with permanent methods (B) All mCPR with long term reversible methods (C) All mCPR with short term methods

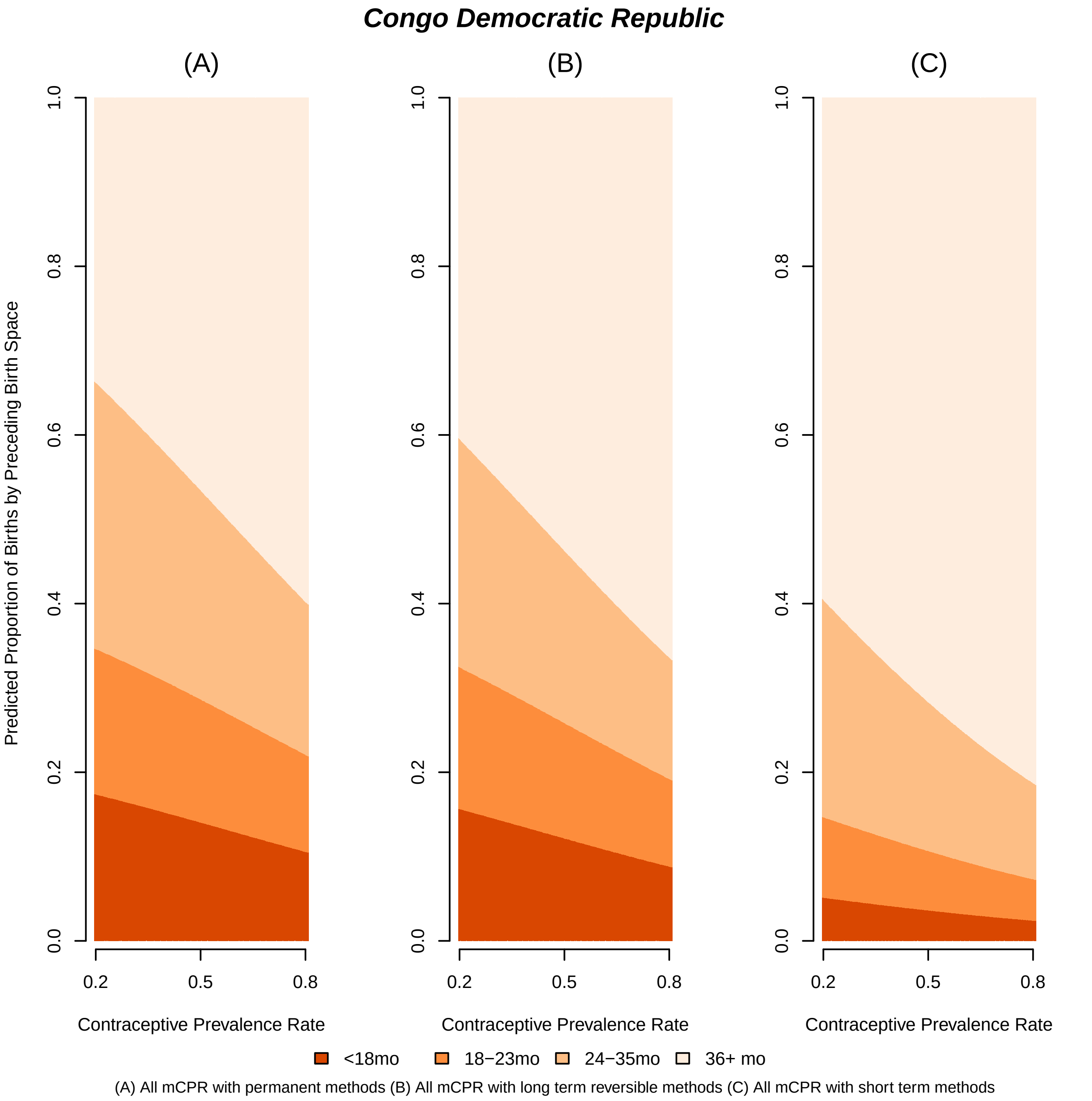

Congo Democratic Republic
(A)
(B)
(C)
Predicted Proportion of Births by Preceding Birth Space
0.0
0.2
0.4
0.6
0.8
1.0
0.2
0.5
0.8
Contraceptive Prevalence Rate
<18mo
18−23mo
24−35mo
36+ mo
(A) All mCPR with permanent methods (B) All mCPR with long term reversible methods (C) All mCPR with short term methods

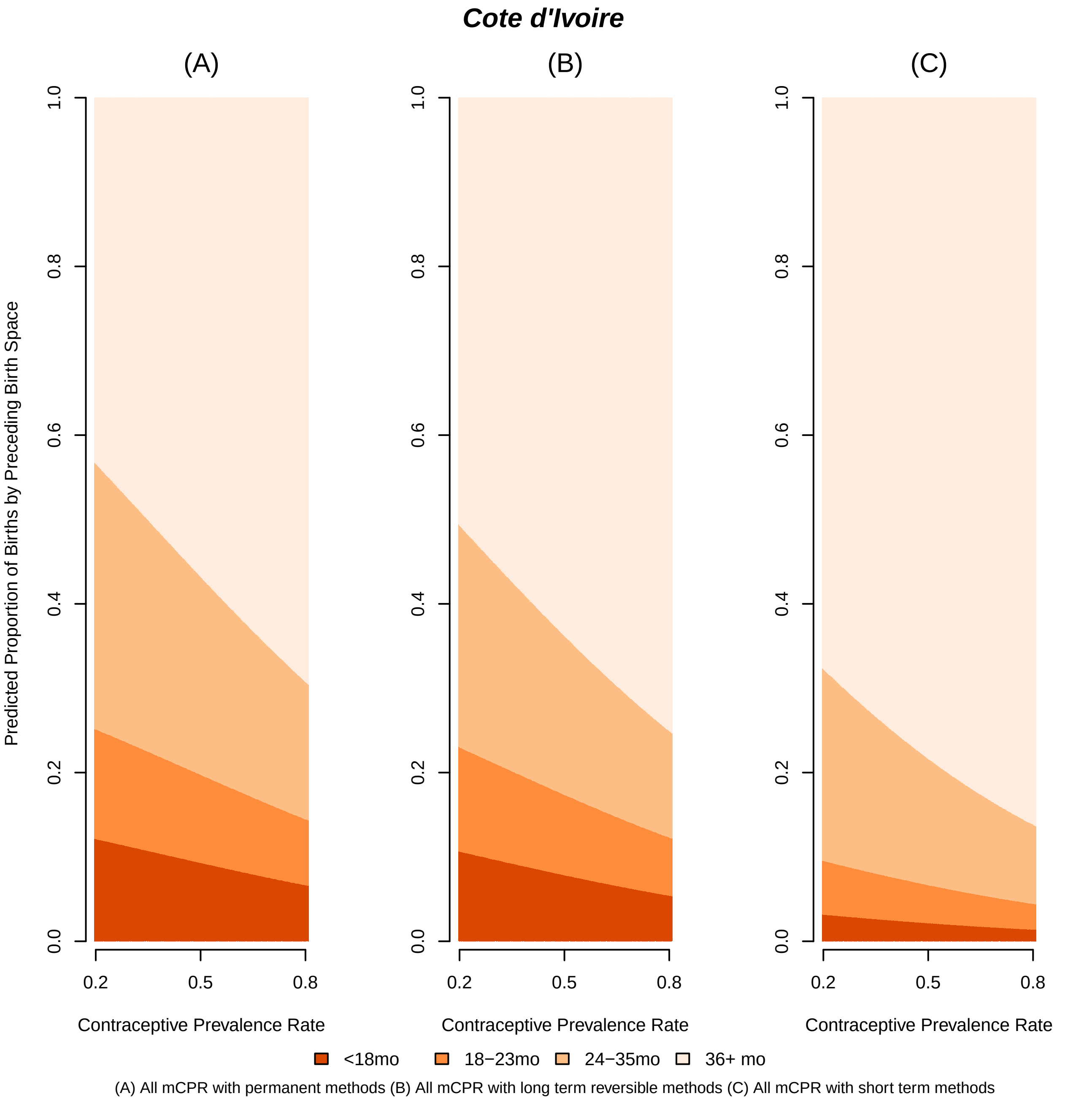

Cote d'Ivoire
(A)
(B)
(C)
Predicted Proportion of Births by Preceding Birth Space
0.0
0.2
0.4
0.6
0.8
1.0
0.2
0.5
0.8
Contraceptive Prevalence Rate
<18mo
18−23mo
24−35mo
36+ mo
(A) All mCPR with permanent methods (B) All mCPR with long term reversible methods (C) All mCPR with short term methods

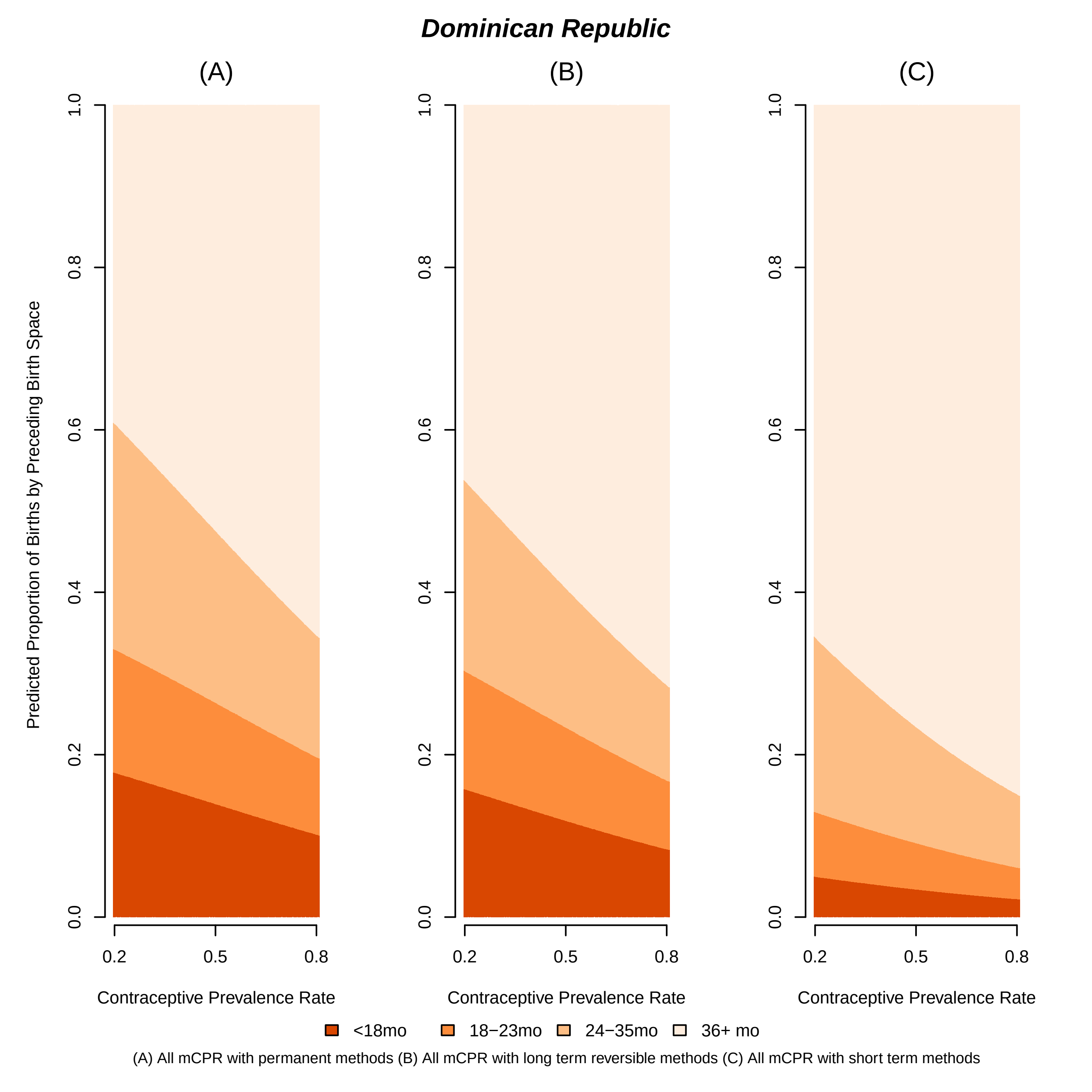
Dominican Republic
(A)
(B)
(C)
Predicted Proportion of Births by Preceding Birth Space
0.0
0.2
0.4
0.6
0.8
1.0
0.2
0.5
0.8
Contraceptive Prevalence Rate
<18mo
18−23mo
24−35mo
36+ mo
(A) All mCPR with permanent methods (B) All mCPR with long term reversible methods (C) All mCPR with short term methods

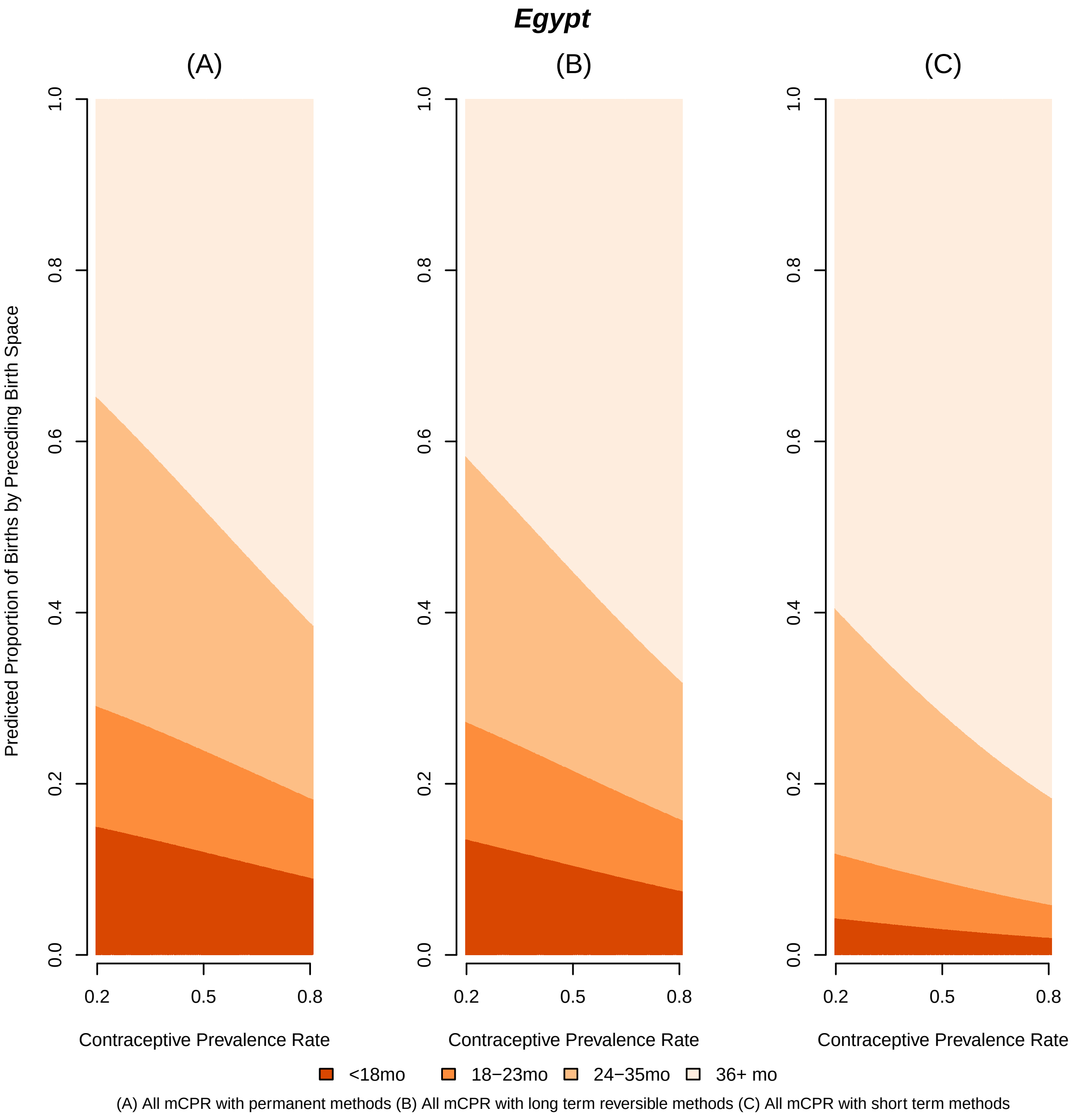

Egypt
(A)
(B)
(C)
Predicted Proportion of Births by Preceding Birth Space
0.0
0.2
0.4
0.6
0.8
1.0
0.2
0.5
0.8
Contraceptive Prevalence Rate
<18mo
18−23mo
24−35mo
36+ mo
(A) All mCPR with permanent methods (B) All mCPR with long term reversible methods (C) All mCPR with short term methods

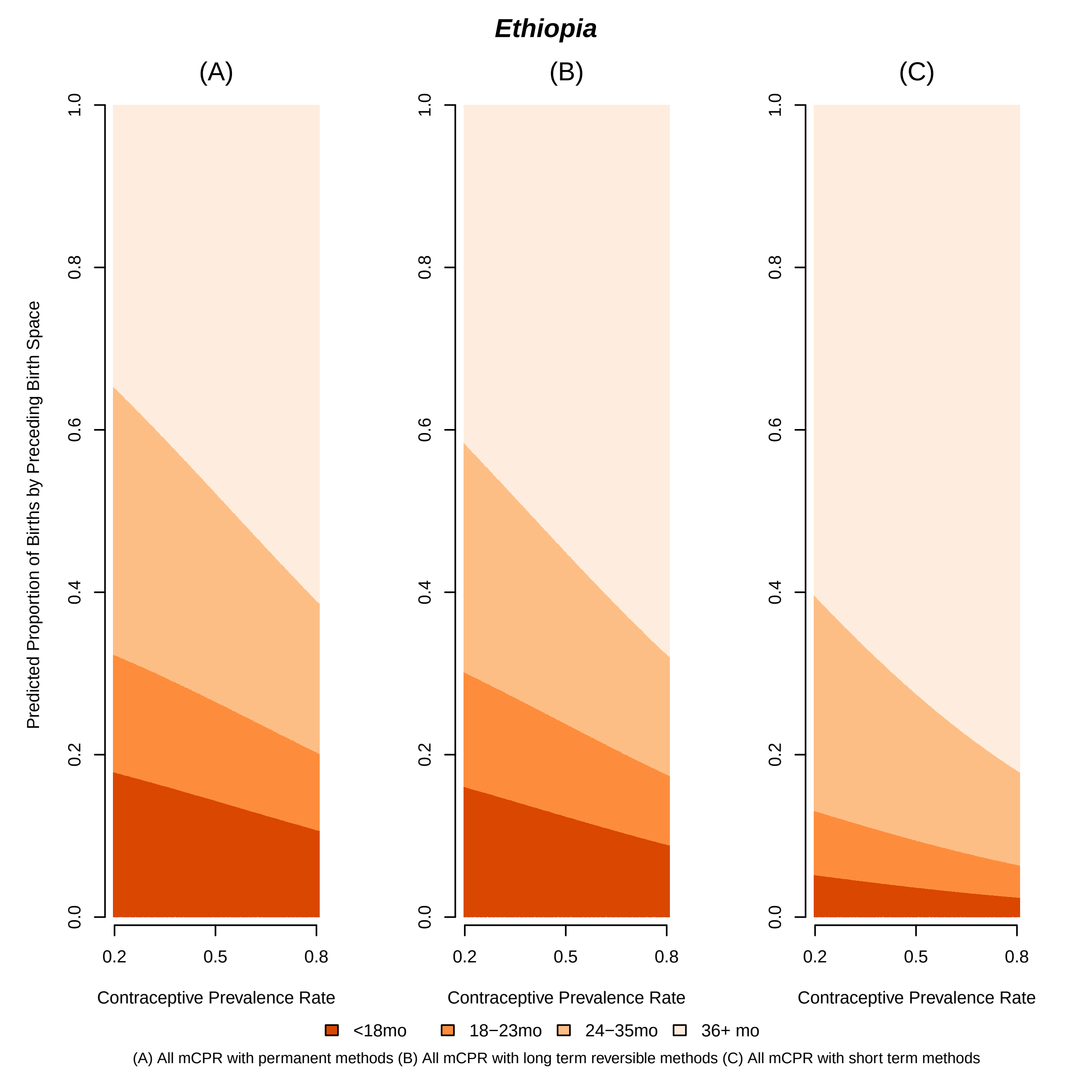

Ethiopia
(A)
(B)
(C)
Predicted Proportion of Births by Preceding Birth Space
0.0
0.2
0.4
0.6
0.8
1.0
0.2
0.5
0.8
Contraceptive Prevalence Rate
<18mo
18−23mo
24−35mo
36+ mo
(A) All mCPR with permanent methods (B) All mCPR with long term reversible methods (C) All mCPR with short term methods

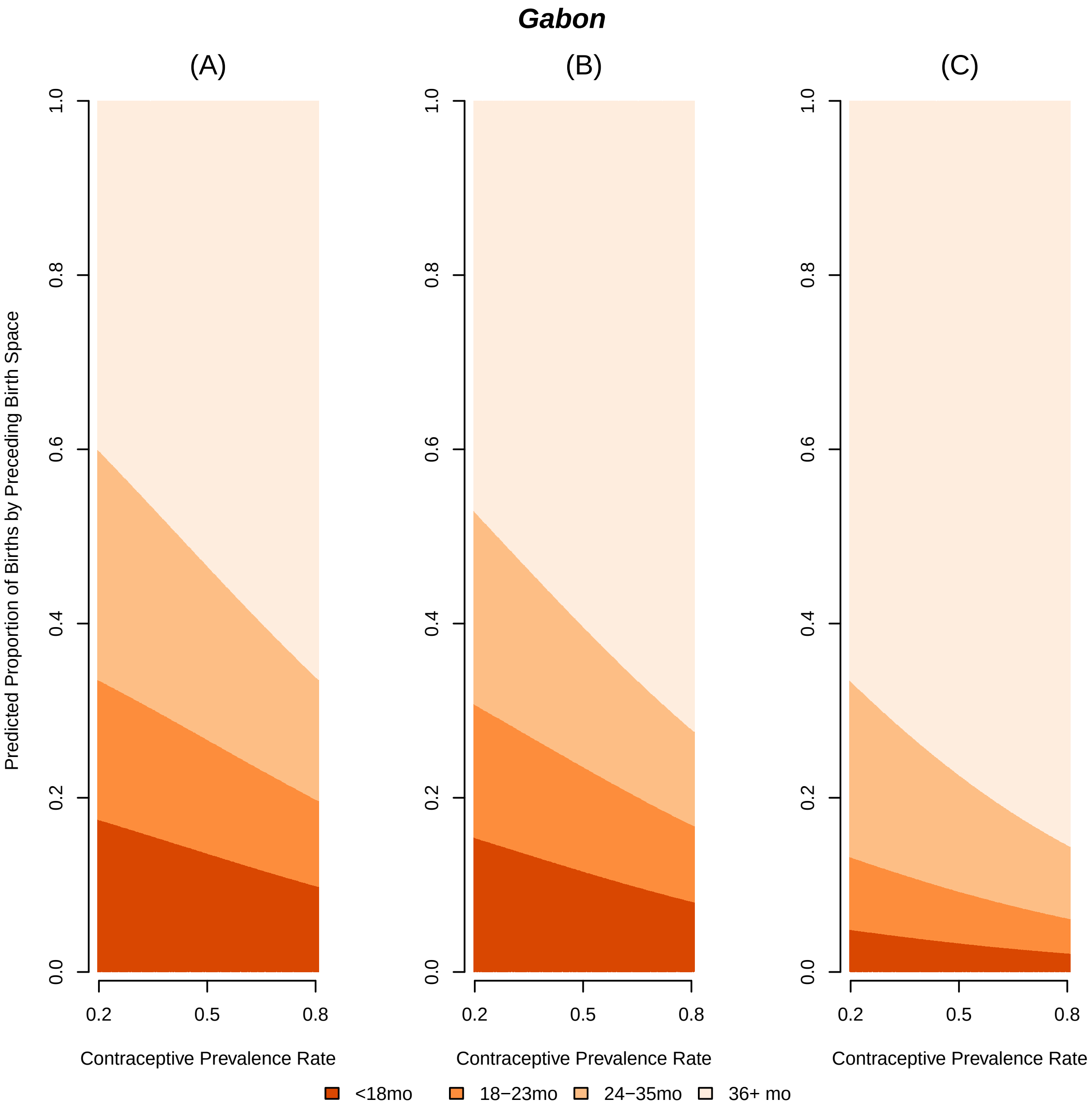

(A) All mCPR with permanent methods (B) All mCPR with long term reversible methods (C) All mCPR with short term methods

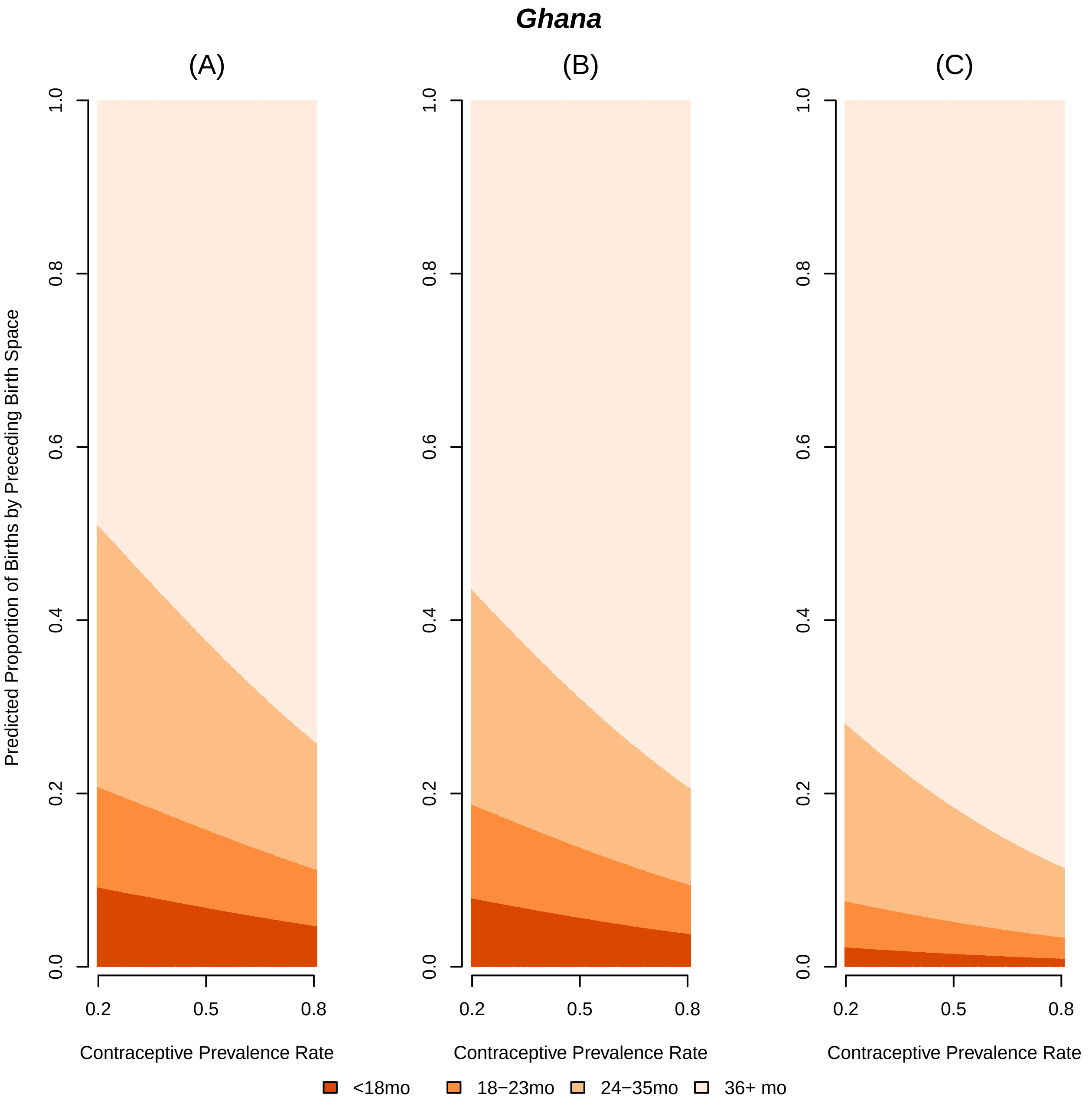

(A) All mCPR with permanent methods (B) All mCPR with long term reversible methods (C) All mCPR with short term methods

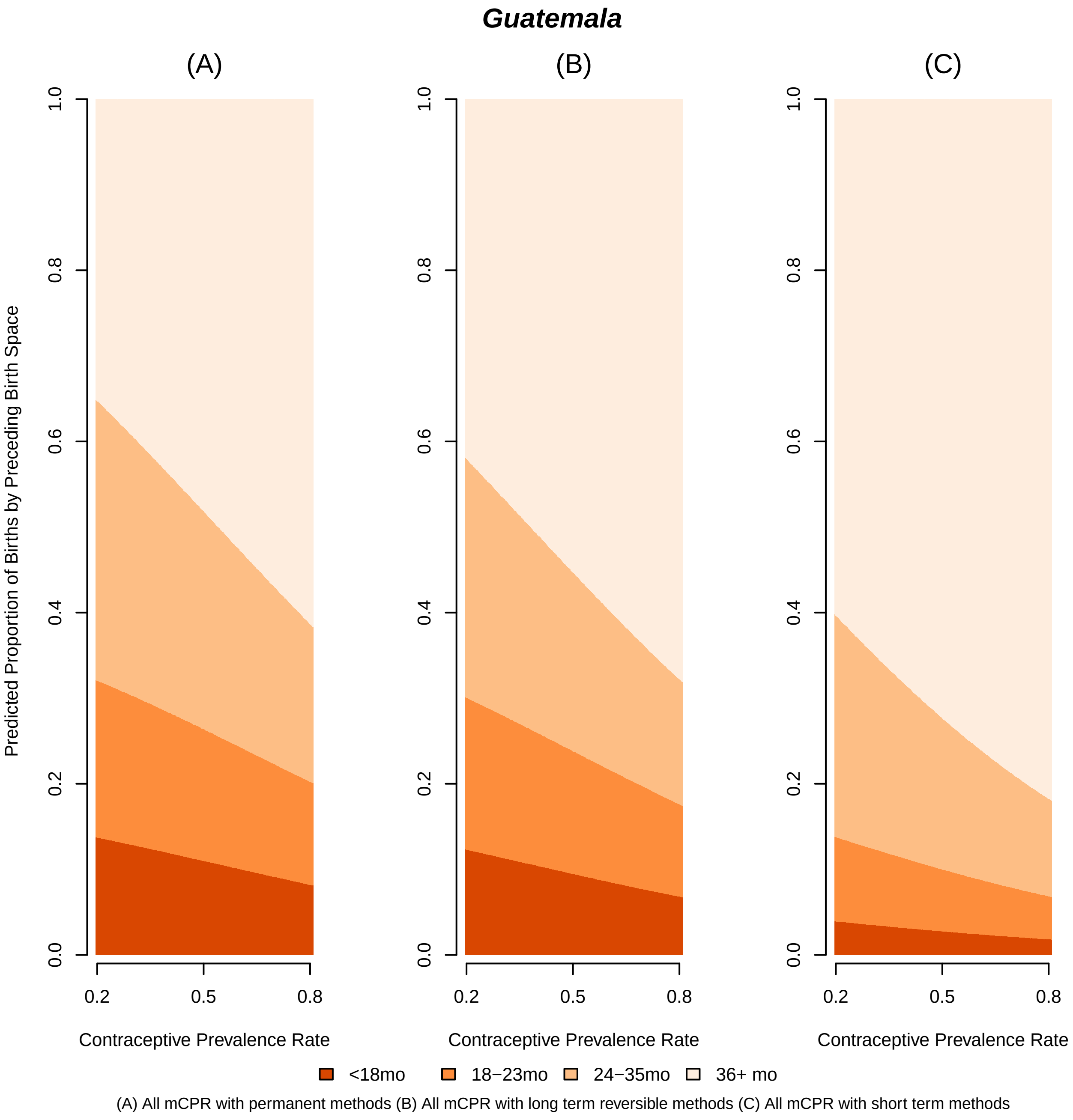
Guatemala
(A)
(B)
(C)
Predicted Proportion of Births by Preceding Birth Space
0.0
0.2
0.4
0.6
0.8
1.0
0.2
0.5
0.8
Contraceptive Prevalence Rate
<18mo
18−23mo
24−35mo
36+ mo
(A) All mCPR with permanent methods (B) All mCPR with long term reversible methods (C) All mCPR with short term methods

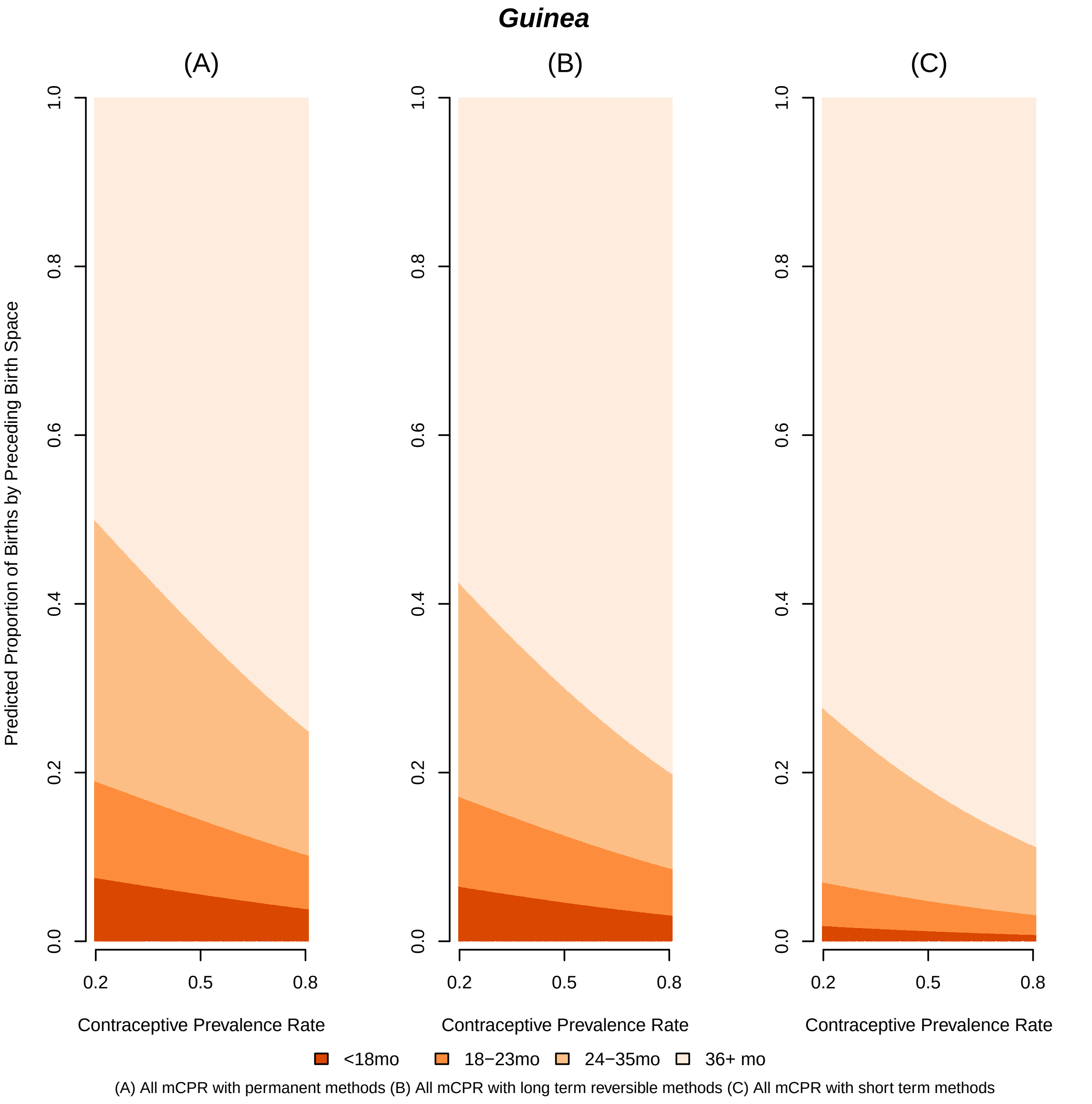
Guinea
(A)
(B)
(C)
Predicted Proportion of Births by Preceding Birth Space
1.0
0.8
0.6
0.4
0.2
0.0
0.2
0.5
0.8
Contraceptive Prevalence Rate
<18mo
18−23mo
24−35mo
36+ mo
(A) All mCPR with permanent methods (B) All mCPR with long term reversible methods (C) All mCPR with short term methods

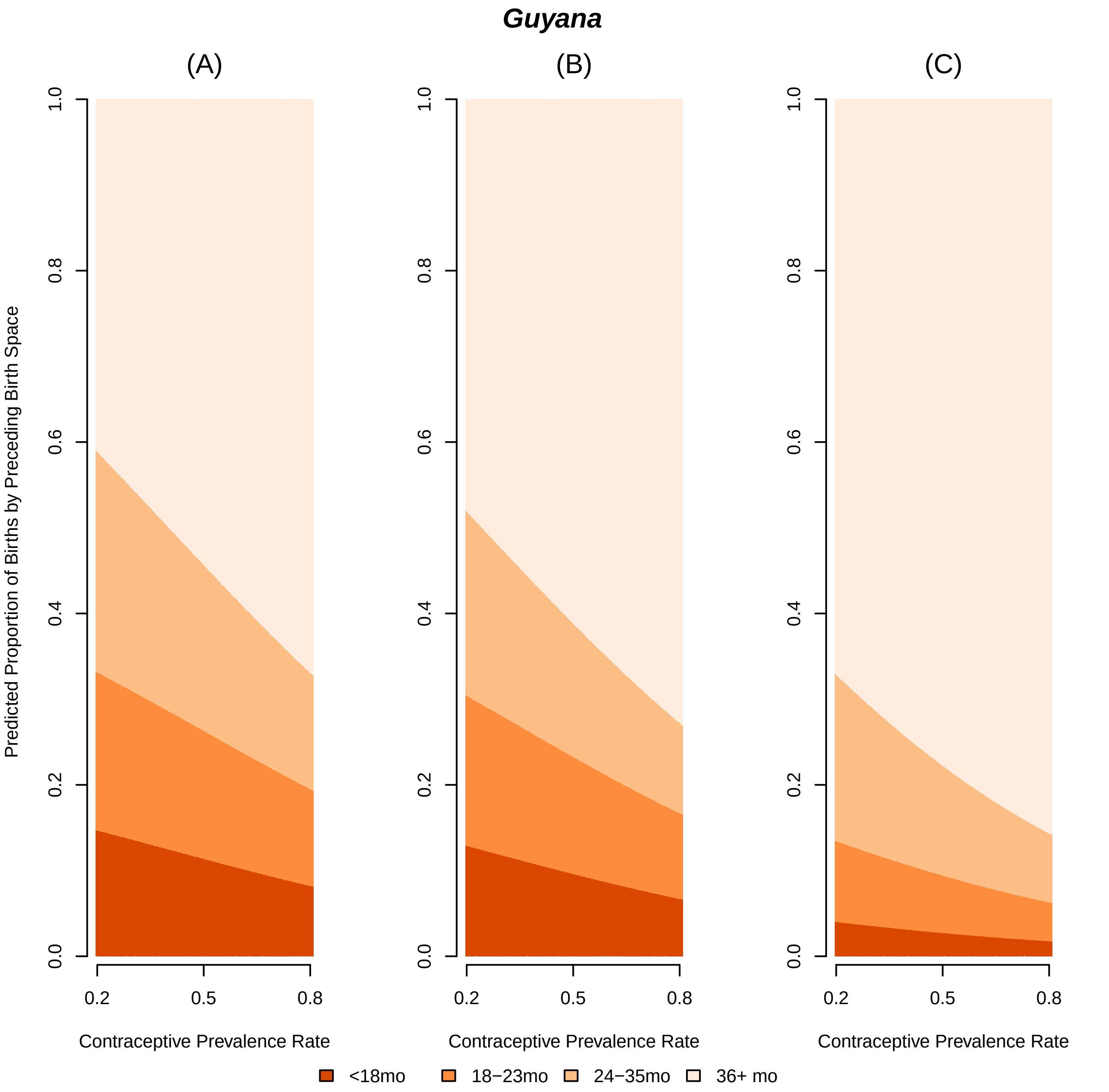

(A) All mCPR with permanent methods (B) All mCPR with long term reversible methods (C) All mCPR with short term methods

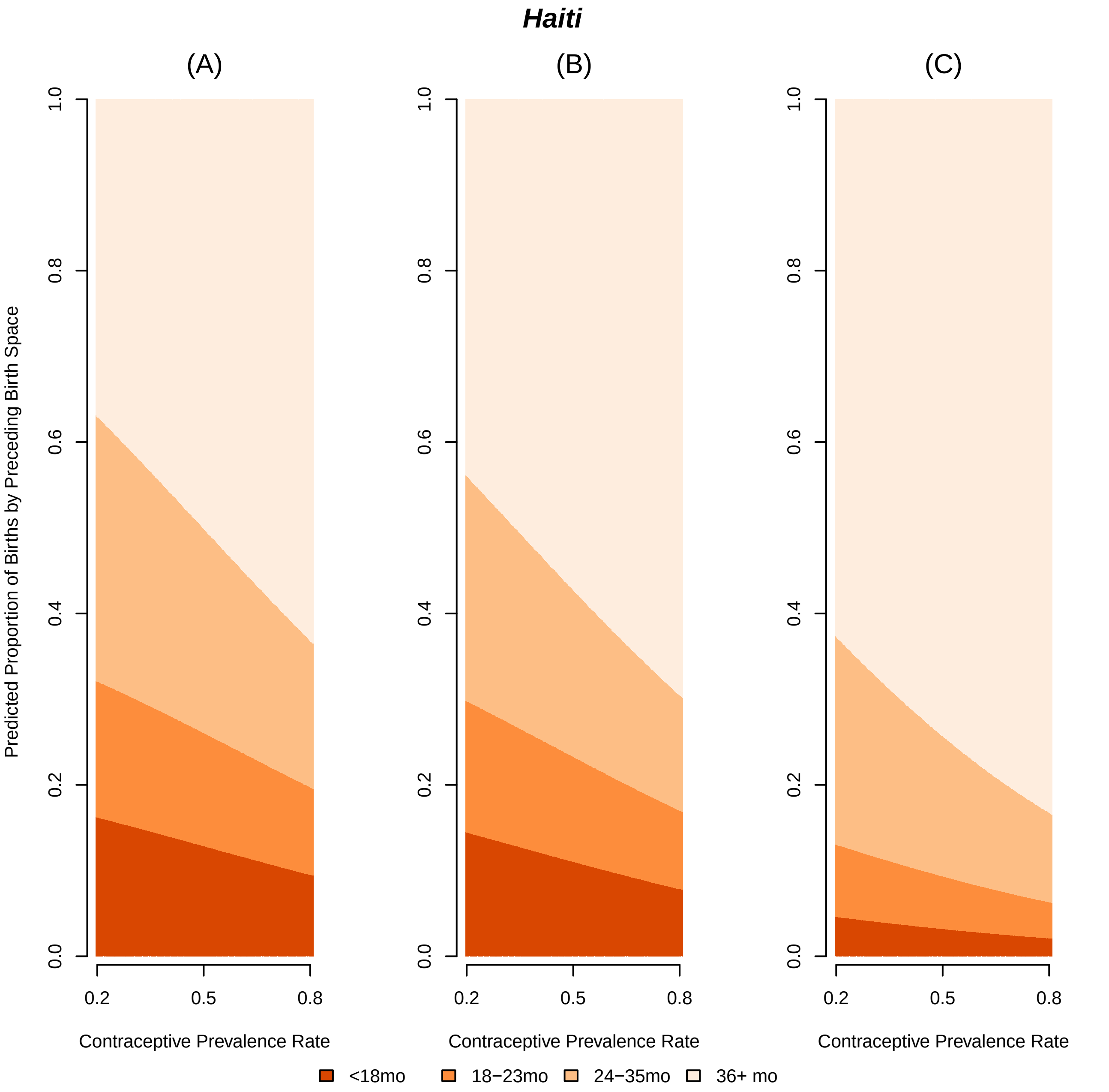

(A) All mCPR with permanent methods (B) All mCPR with long term reversible methods (C) All mCPR with short term methods

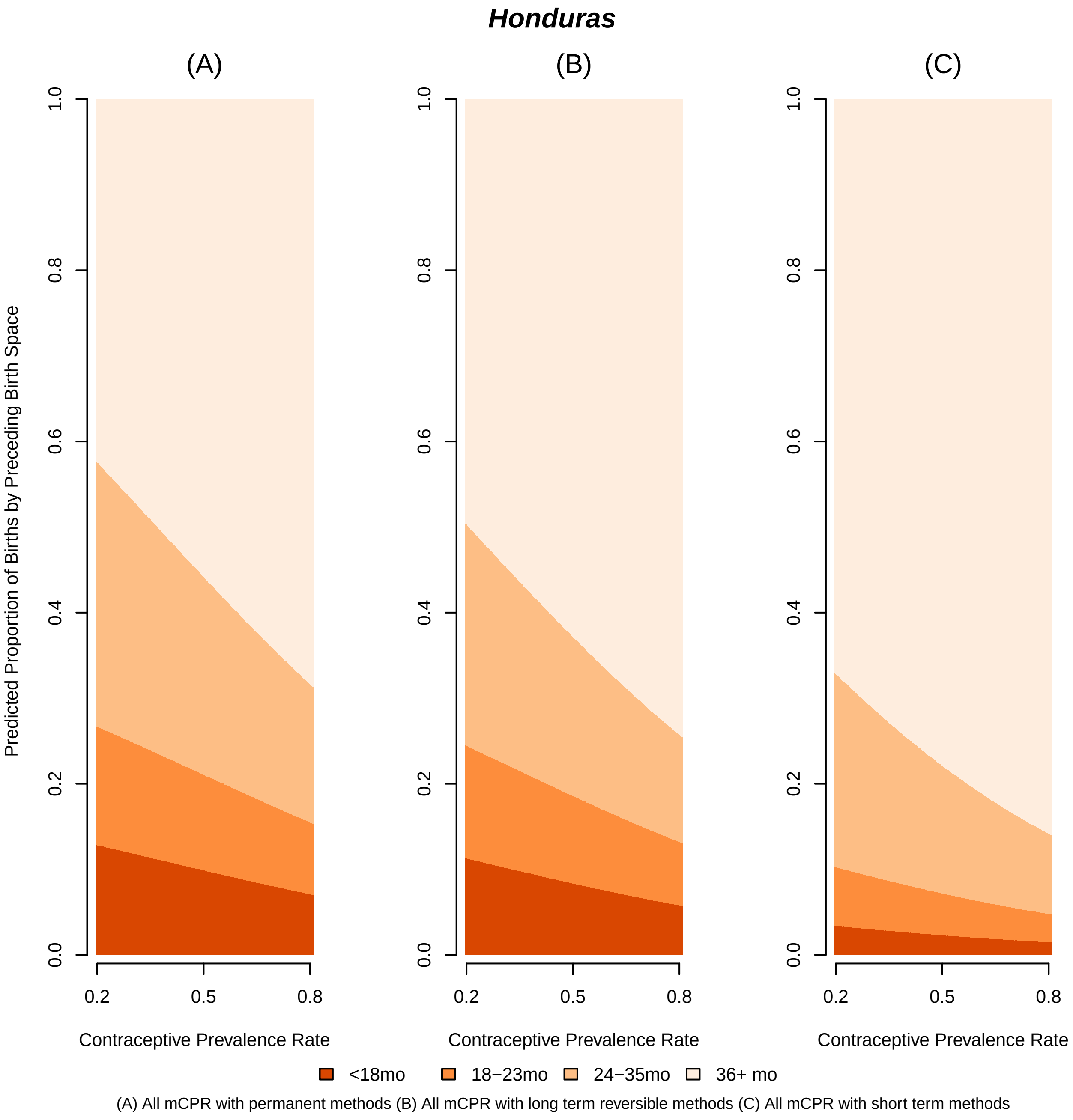
Honduras
(A)
(B)
(C)
Predicted Proportion of Births by Preceding Birth Space
0.0
0.2
0.4
0.6
0.8
1.0
0.2
0.5
0.8
Contraceptive Prevalence Rate
<18mo
18−23mo
24−35mo
36+ mo
(A) All mCPR with permanent methods (B) All mCPR with long term reversible methods (C) All mCPR with short term methods

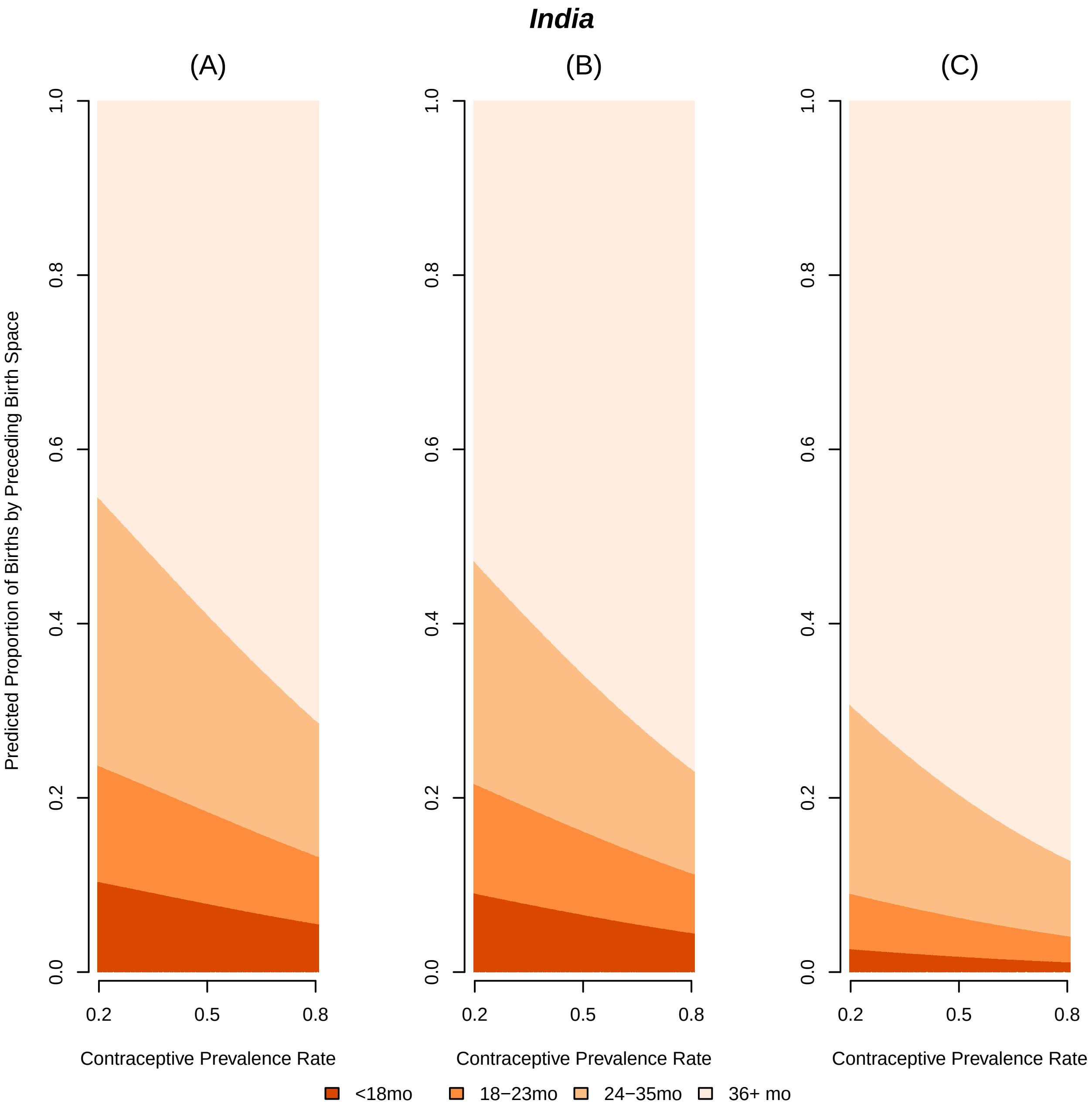

(A) All mCPR with permanent methods (B) All mCPR with long term reversible methods (C) All mCPR with short term methods

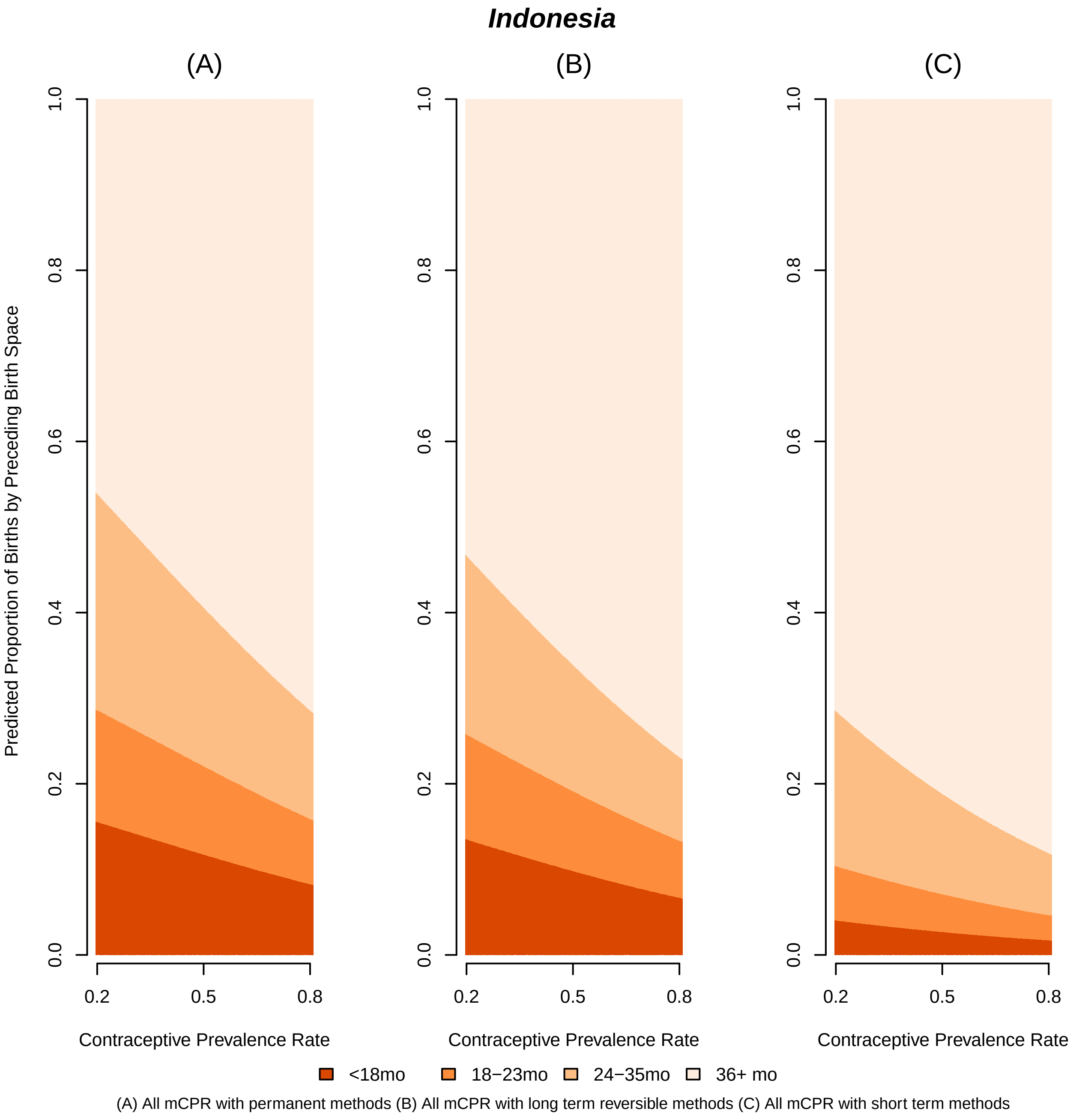
Indonesia
(A)
(B)
(C)
Predicted Proportion of Births by Preceding Birth Space
0.0
0.2
0.4
0.6
0.8
1.0
0.2
0.5
0.8
Contraceptive Prevalence Rate
<18mo
18−23mo
24−35mo
36+ mo
(A) All mCPR with permanent methods (B) All mCPR with long term reversible methods (C) All mCPR with short term methods

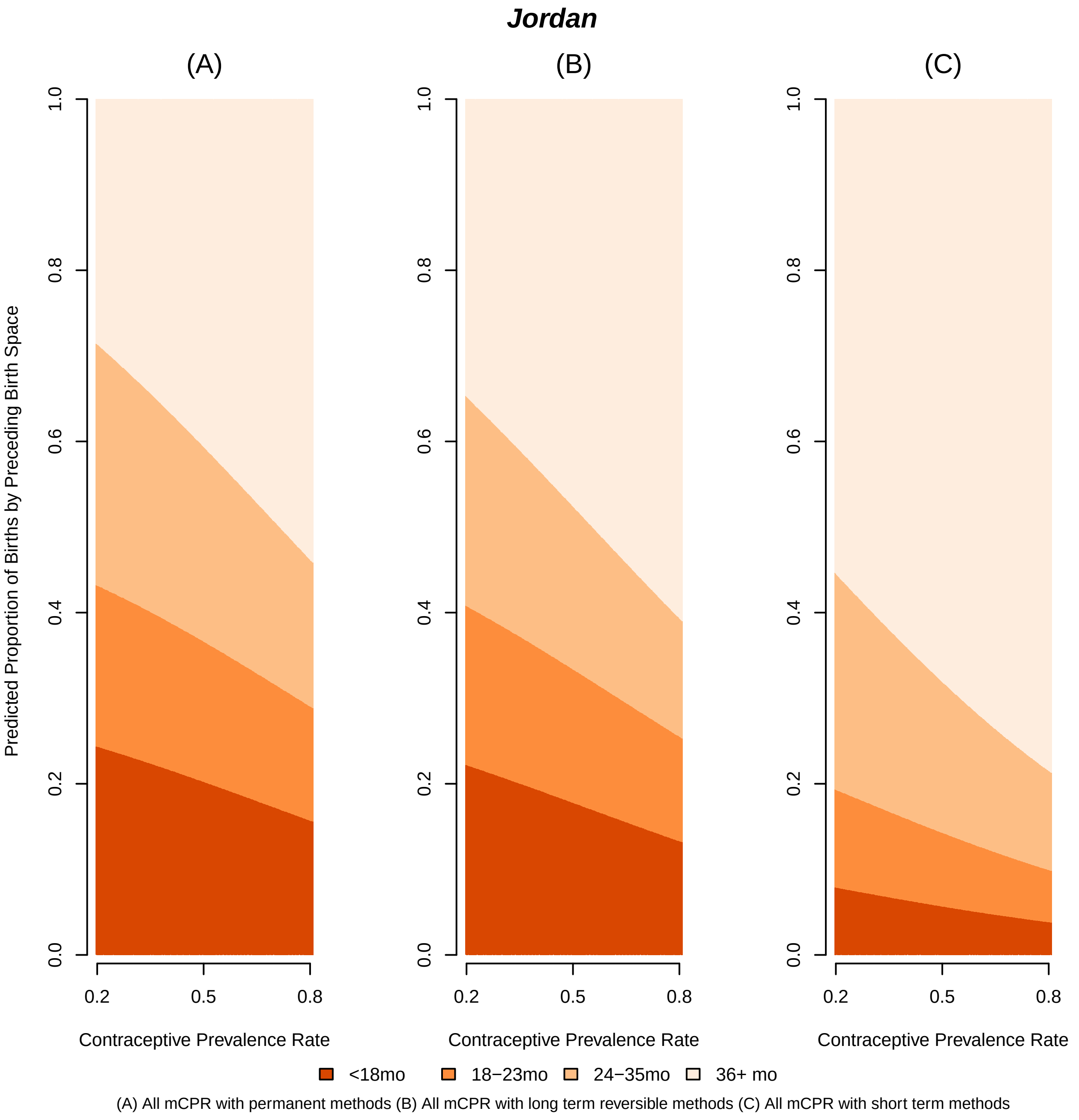
Jordan
(A)
(B)
(C)
Predicted Proportion of Births by Preceding Birth Space
0.0
0.2
0.4
0.6
0.8
1.0
0.2
0.5
0.8
Contraceptive Prevalence Rate
<18mo
18−23mo
24−35mo
36+ mo
(A) All mCPR with permanent methods (B) All mCPR with long term reversible methods (C) All mCPR with short term methods

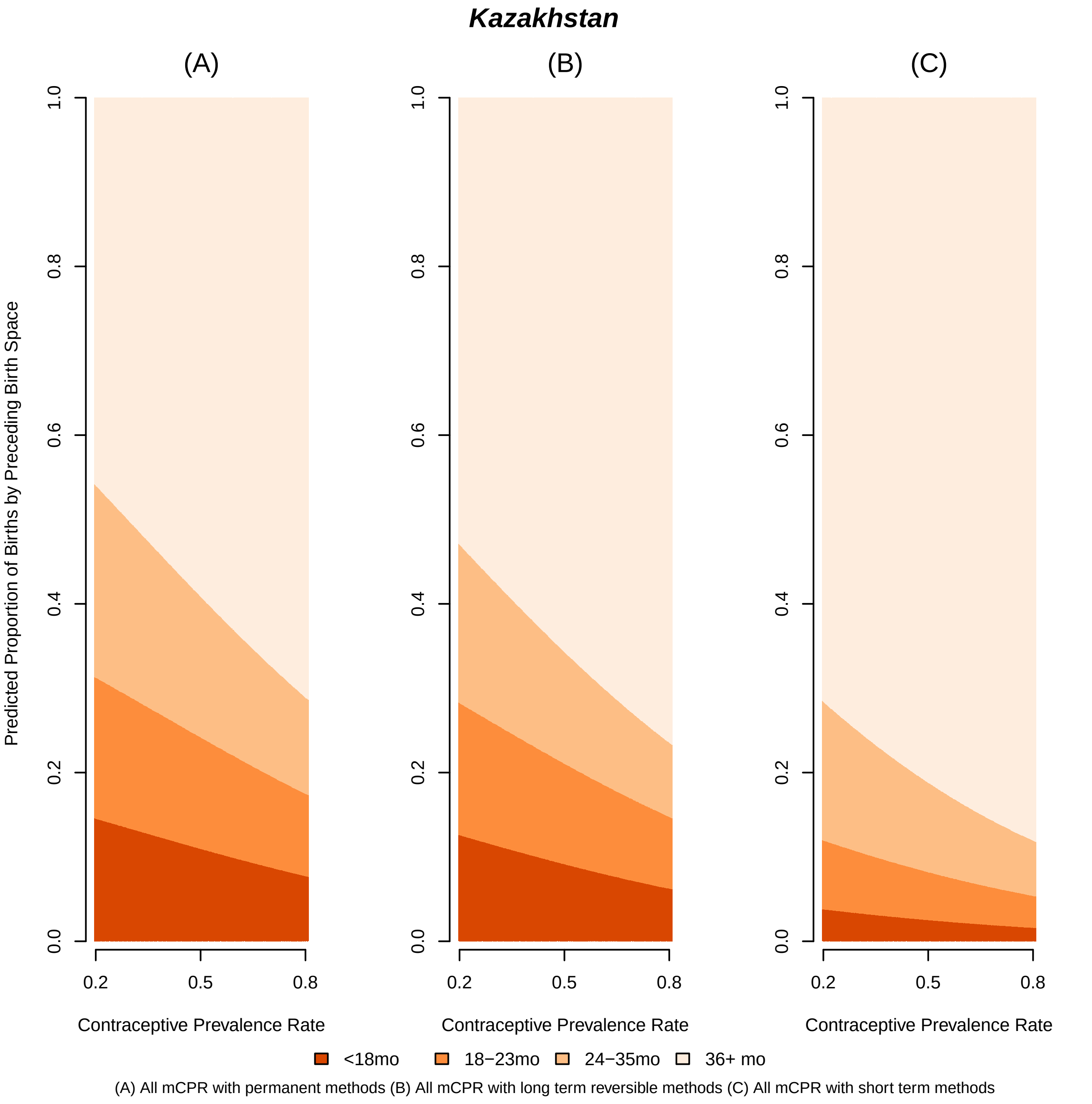

Kazakhstan
(A)
(B)
(C)
Predicted Proportion of Births by Preceding Birth Space
0.0
0.2
0.4
0.6
0.8
1.0
0.2
0.5
0.8
Contraceptive Prevalence Rate
<18mo
18−23mo
24−35mo
36+ mo
(A) All mCPR with permanent methods (B) All mCPR with long term reversible methods (C) All mCPR with short term methods

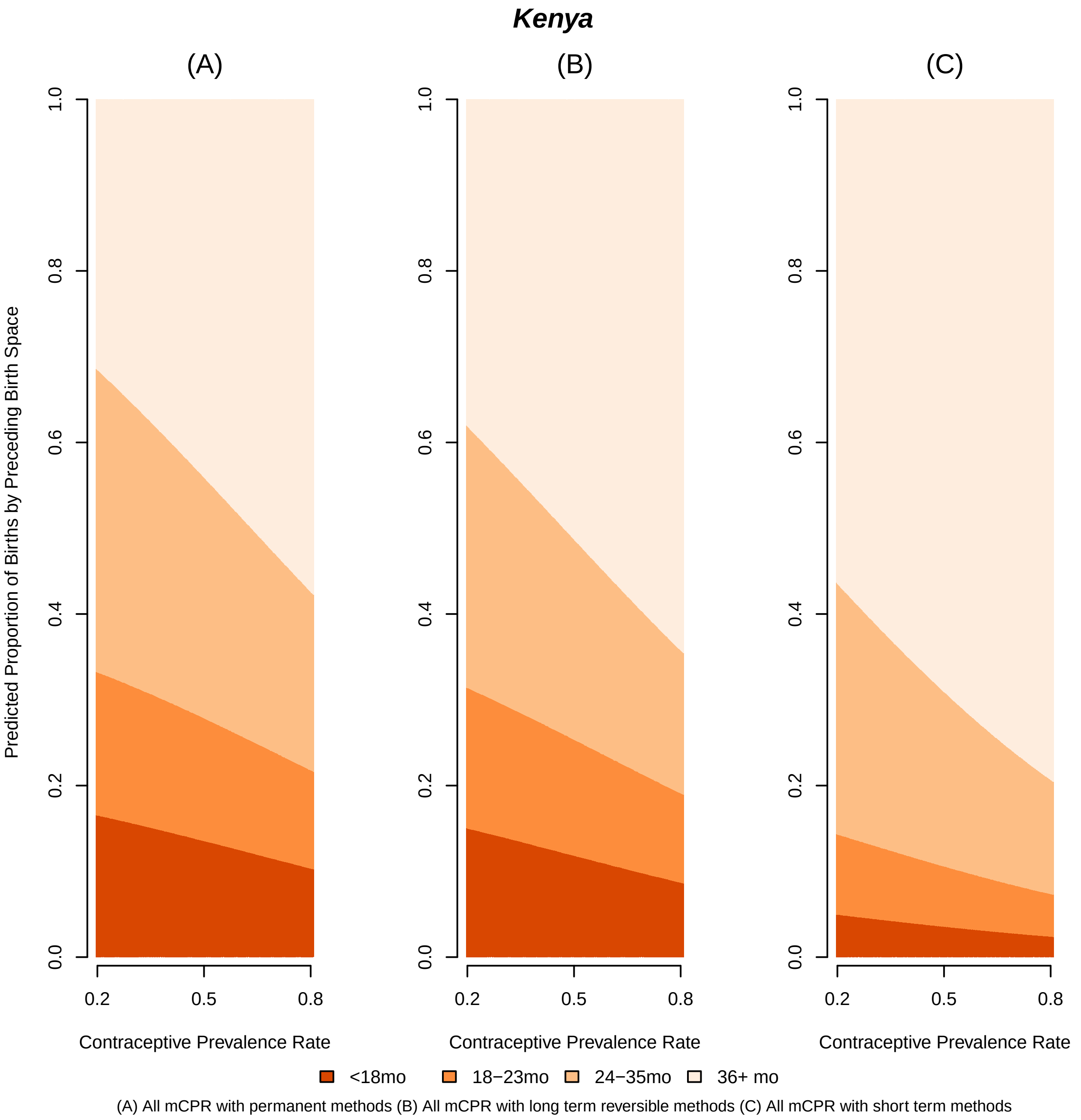
Kenya
(A)
(B)
(C)
Predicted Proportion of Births by Preceding Birth Space
0.0
0.2
0.4
0.6
0.8
1.0
0.2
0.5
0.8
Contraceptive Prevalence Rate
<18mo
18−23mo
24−35mo
36+ mo
(A) All mCPR with permanent methods (B) All mCPR with long term reversible methods (C) All mCPR with short term methods

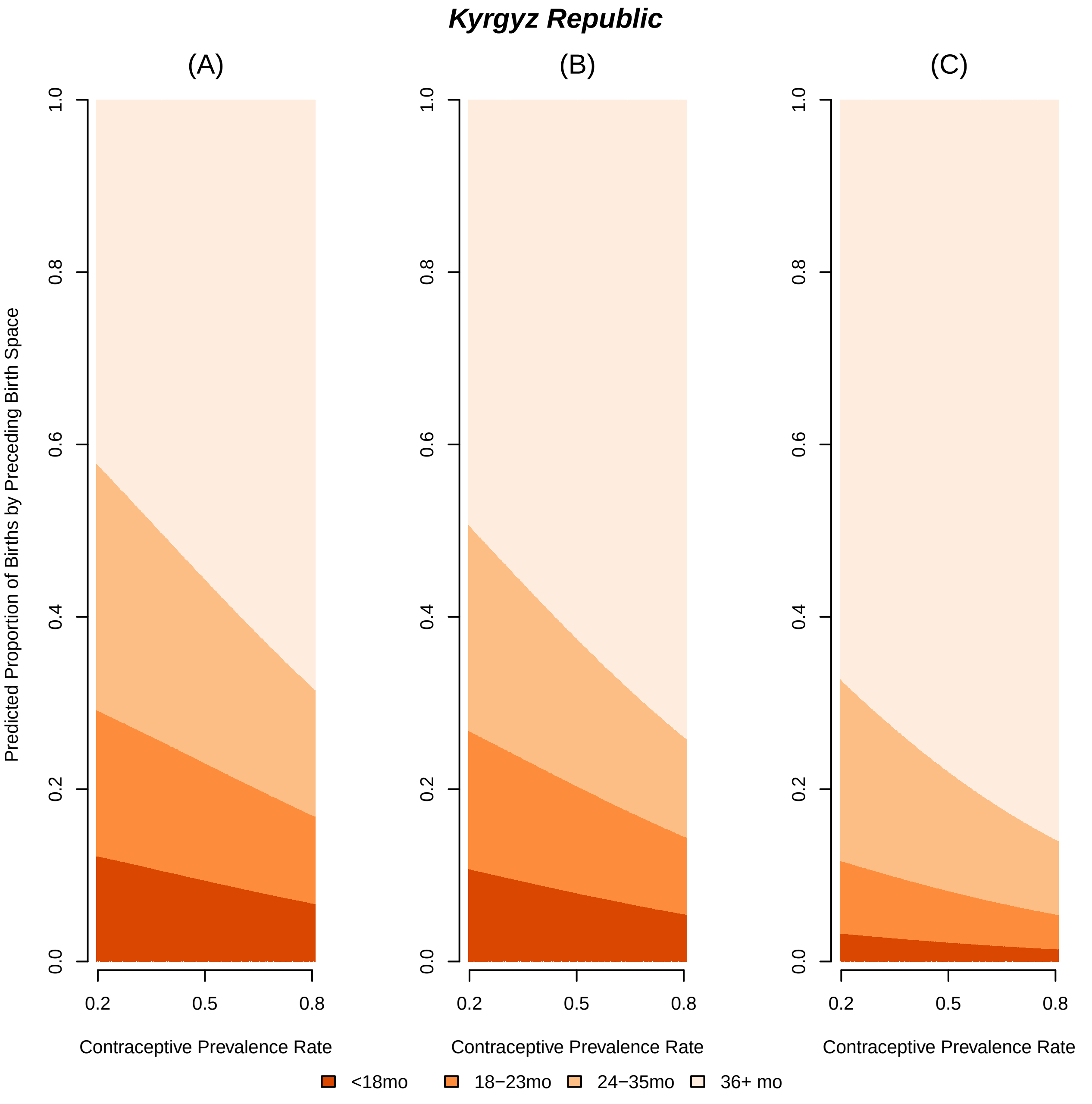

(A) All mCPR with permanent methods (B) All mCPR with long term reversible methods (C) All mCPR with short term methods

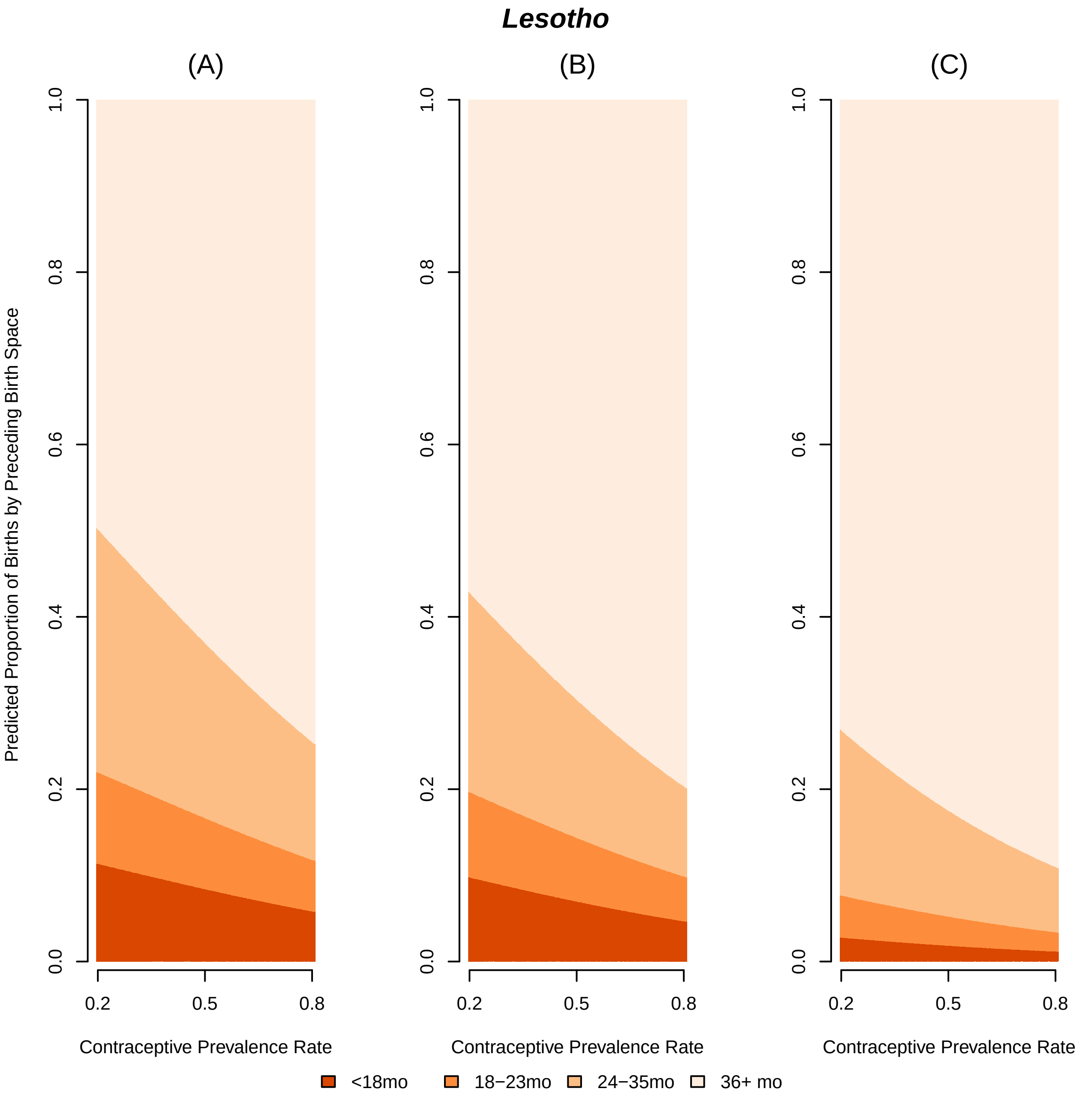

(A) All mCPR with permanent methods (B) All mCPR with long term reversible methods (C) All mCPR with short term methods

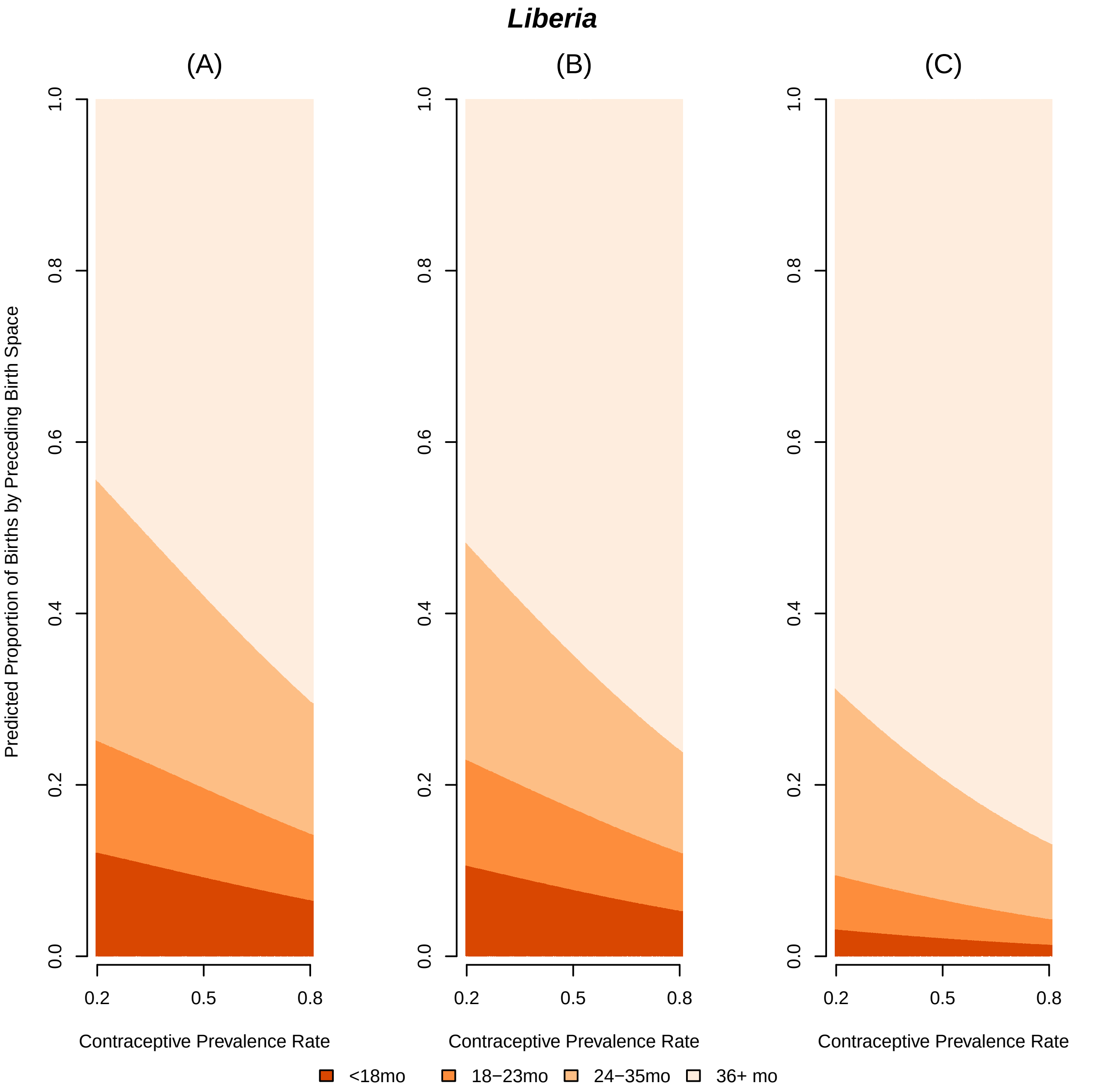

(A) All mCPR with permanent methods (B) All mCPR with long term reversible methods (C) All mCPR with short term methods

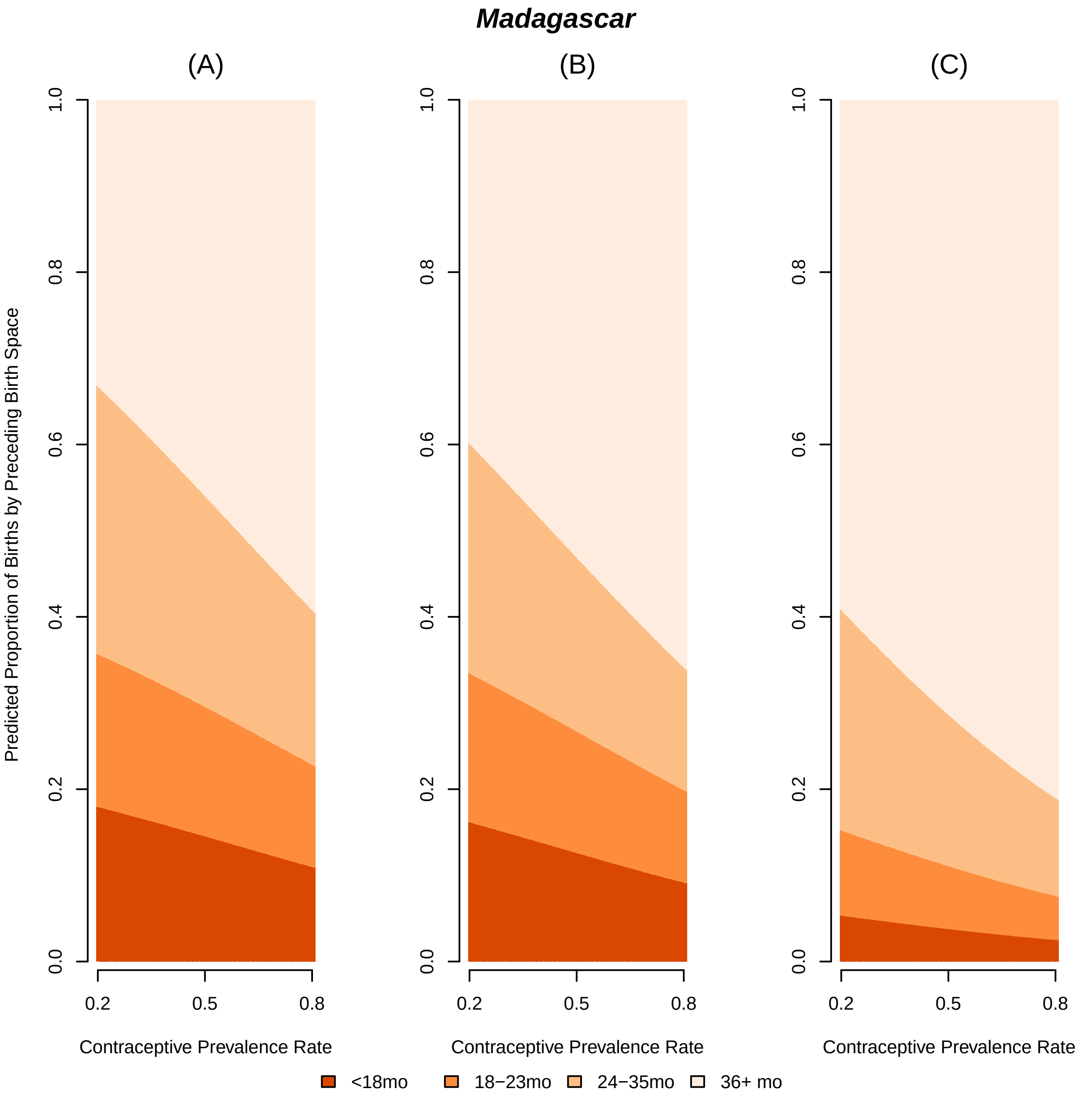

(A) All mCPR with permanent methods (B) All mCPR with long term reversible methods (C) All mCPR with short term methods

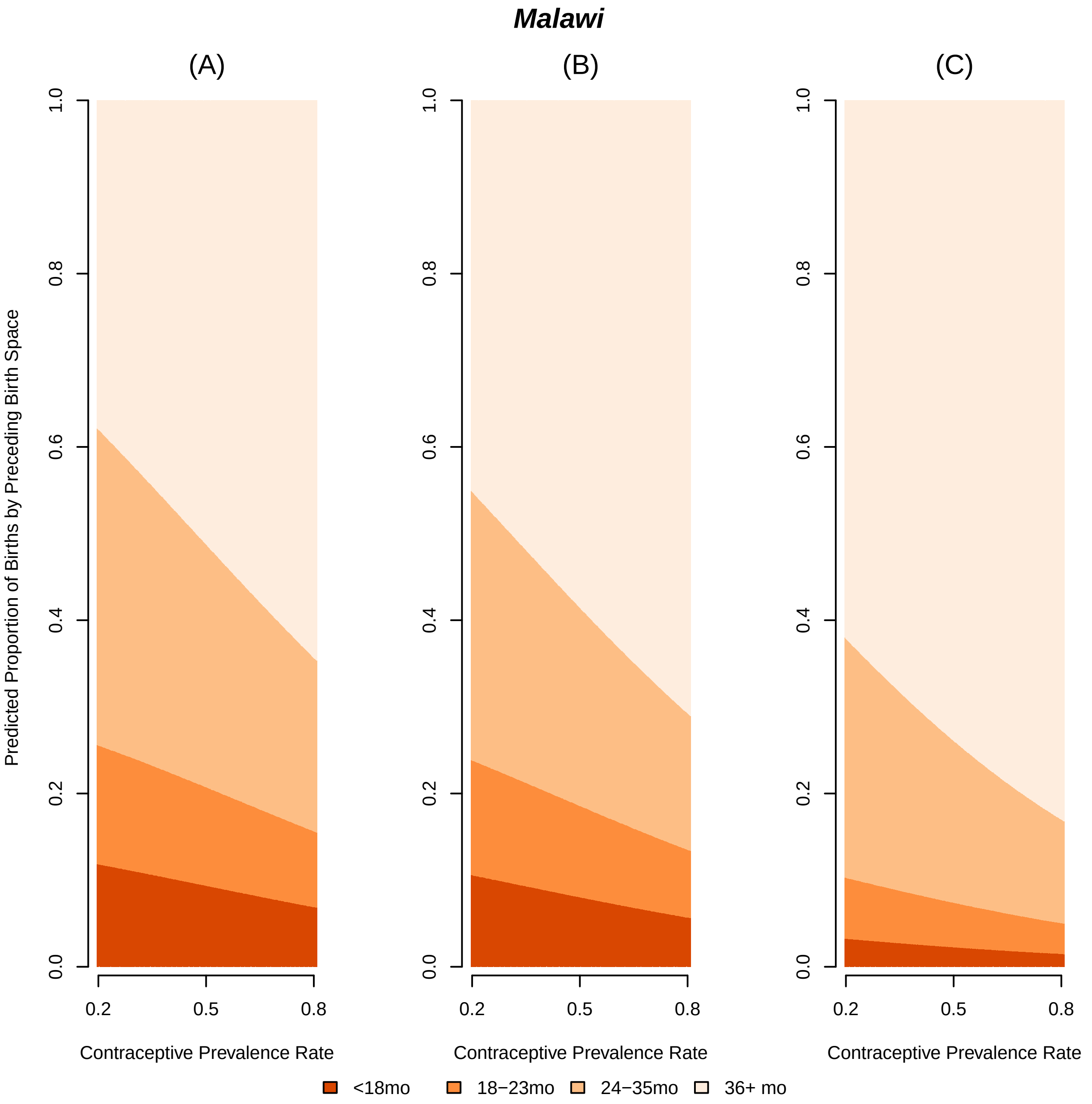

(A) All mCPR with permanent methods (B) All mCPR with long term reversible methods (C) All mCPR with short term methods

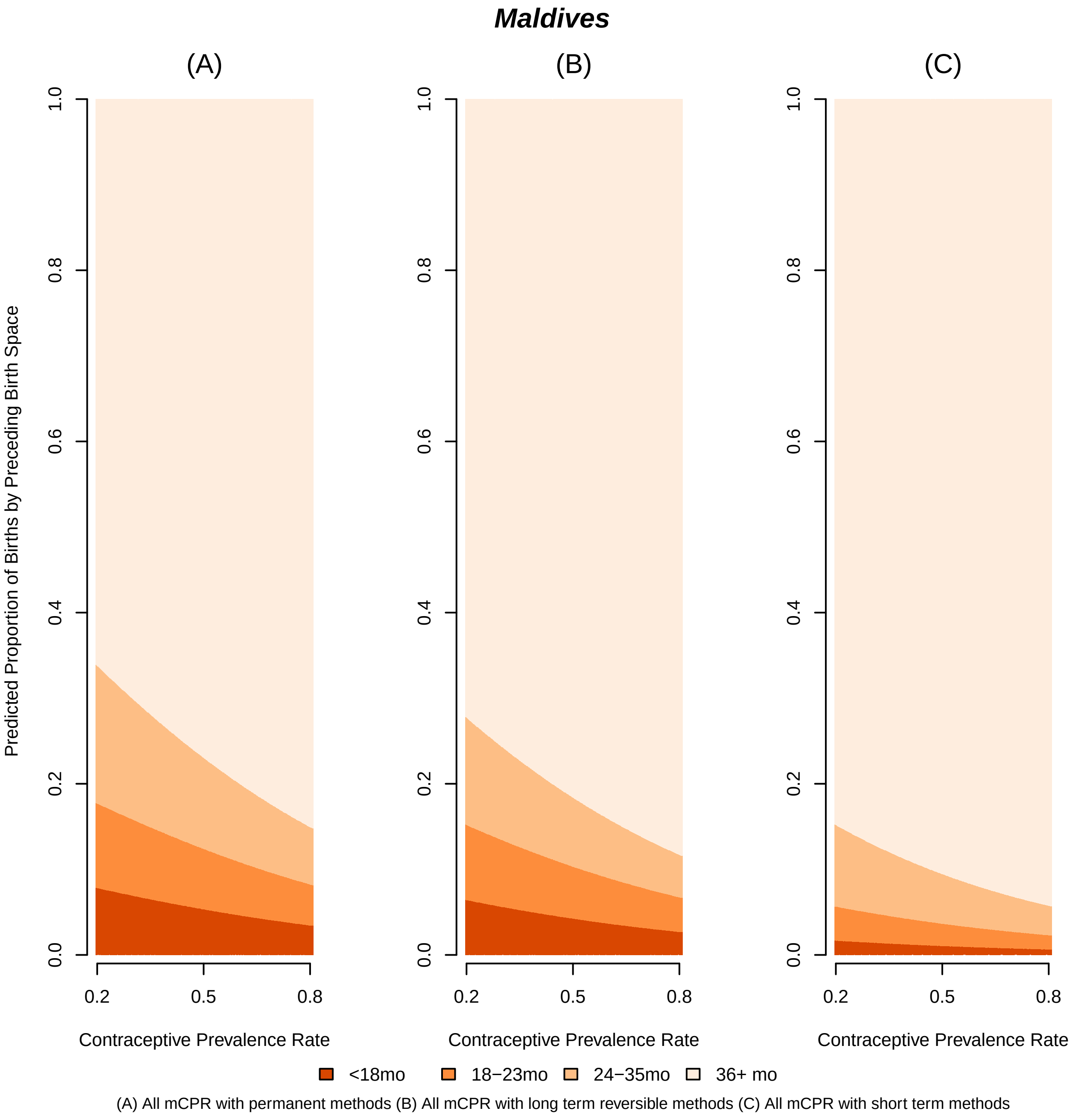
Maldives
(A)
(B)
(C)
Predicted Proportion of Births by Preceding Birth Space
0.0
0.2
0.4
0.6
0.8
1.0
0.2
0.5
0.8
Contraceptive Prevalence Rate
<18mo
18−23mo
24−35mo
36+ mo
(A) All mCPR with permanent methods (B) All mCPR with long term reversible methods (C) All mCPR with short term methods

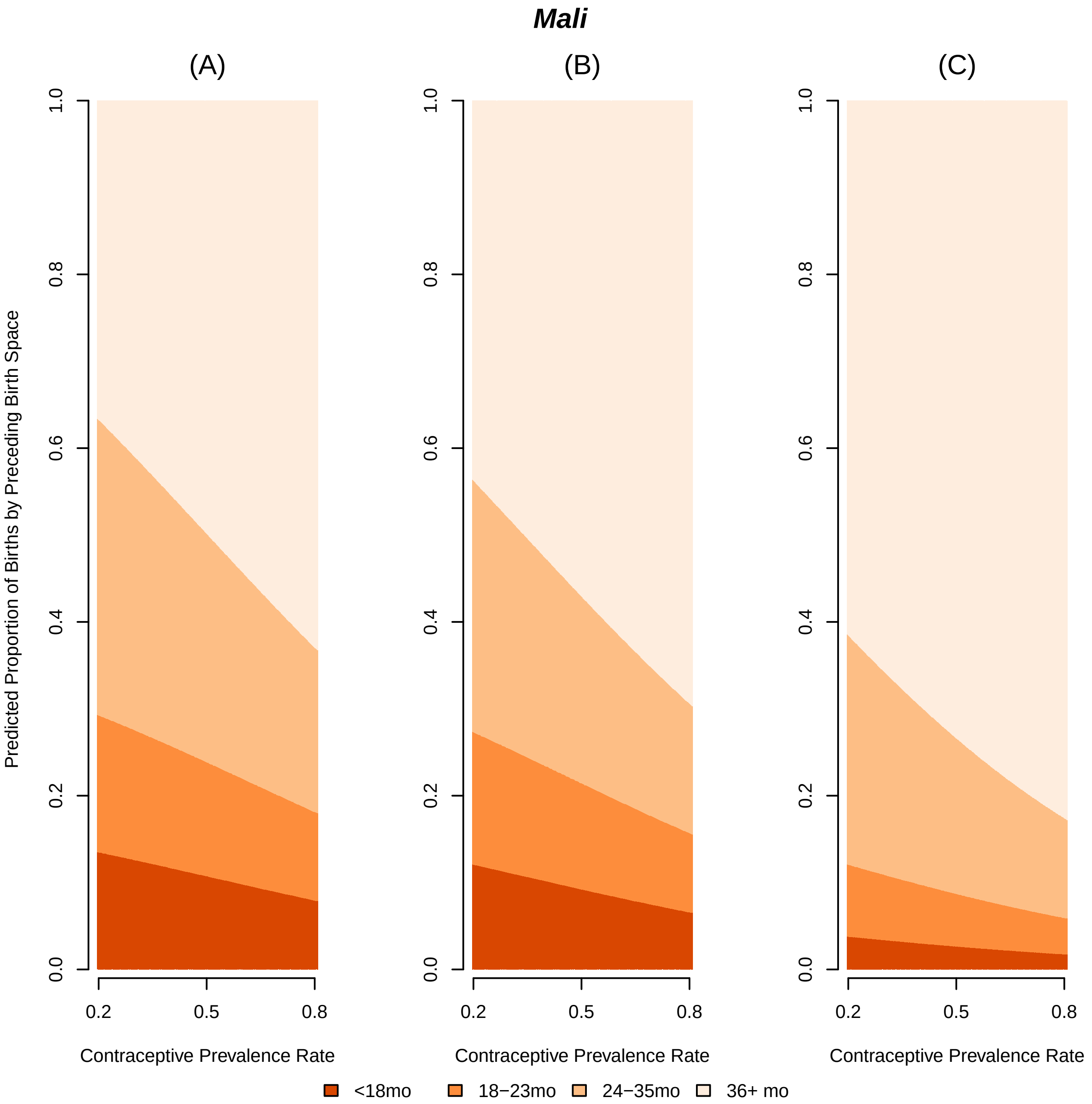

(A) All mCPR with permanent methods (B) All mCPR with long term reversible methods (C) All mCPR with short term methods

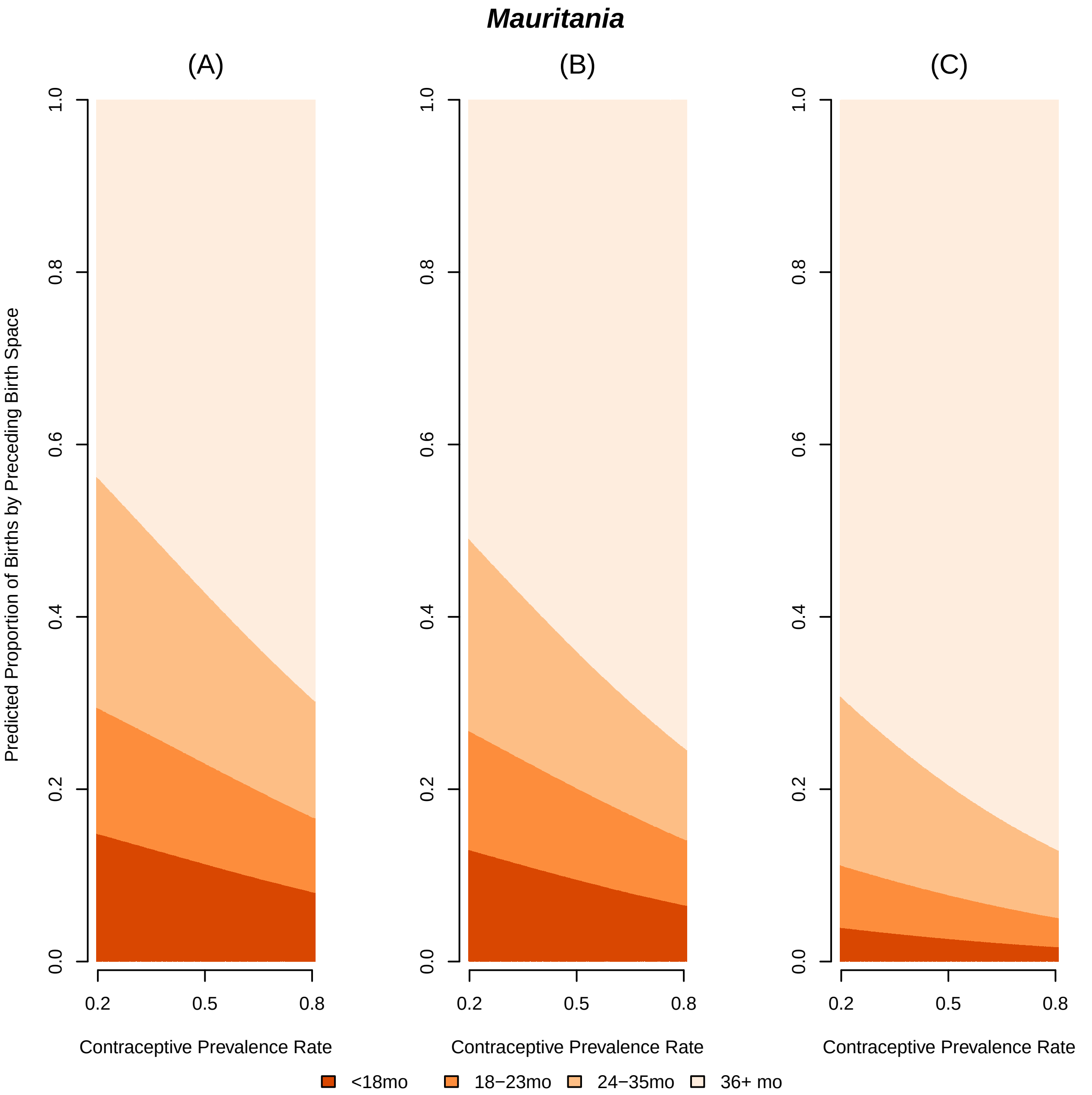

(A) All mCPR with permanent methods (B) All mCPR with long term reversible methods (C) All mCPR with short term methods

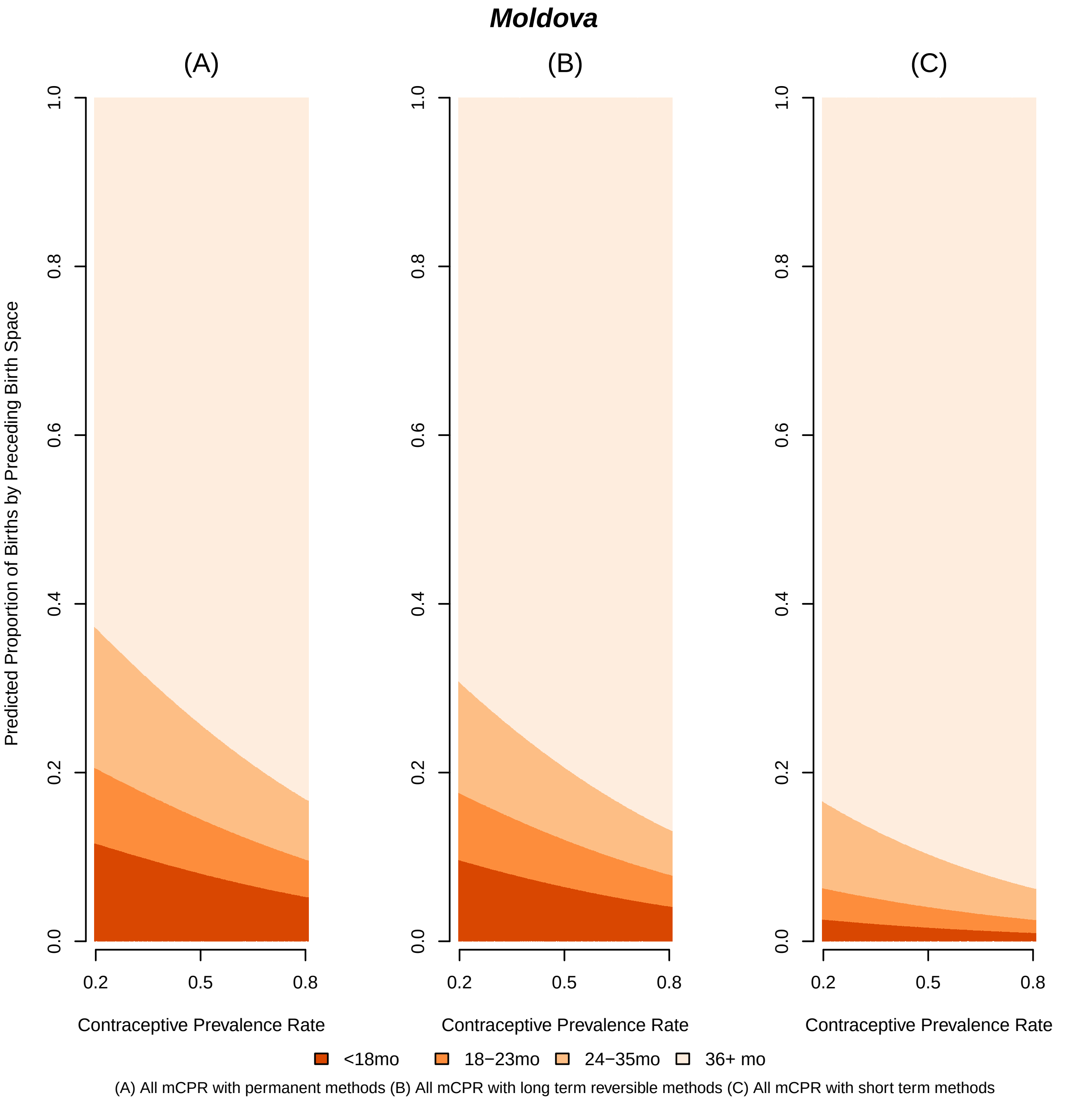
Moldova
(A)
(B)
(C)
Predicted Proportion of Births by Preceding Birth Space
0.0
0.2
0.4
0.6
0.8
1.0
0.2
0.5
0.8
Contraceptive Prevalence Rate
<18mo
18−23mo
24−35mo
36+ mo
(A) All mCPR with permanent methods (B) All mCPR with long term reversible methods (C) All mCPR with short term methods

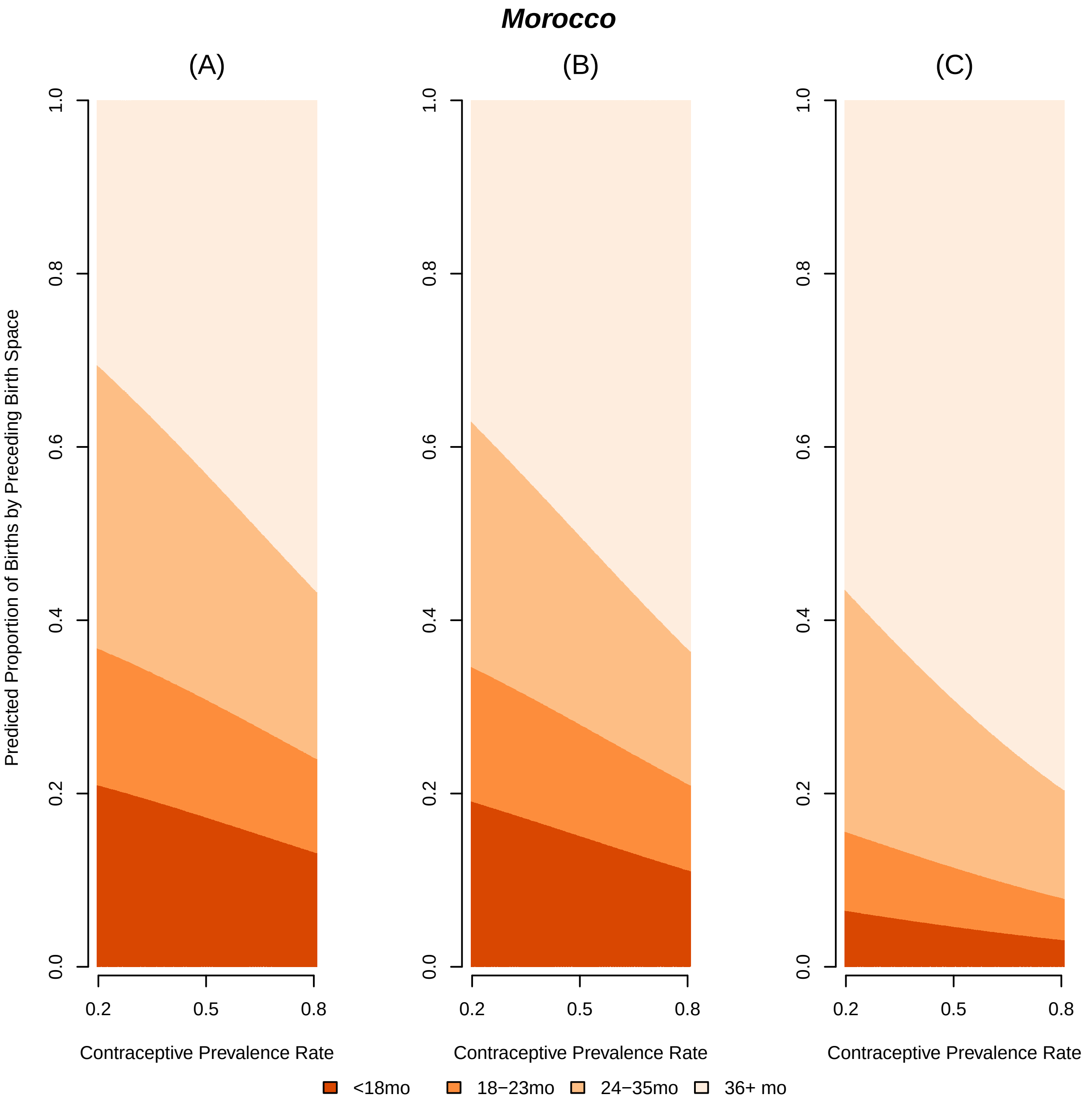

(A) All mCPR with permanent methods (B) All mCPR with long term reversible methods (C) All mCPR with short term methods

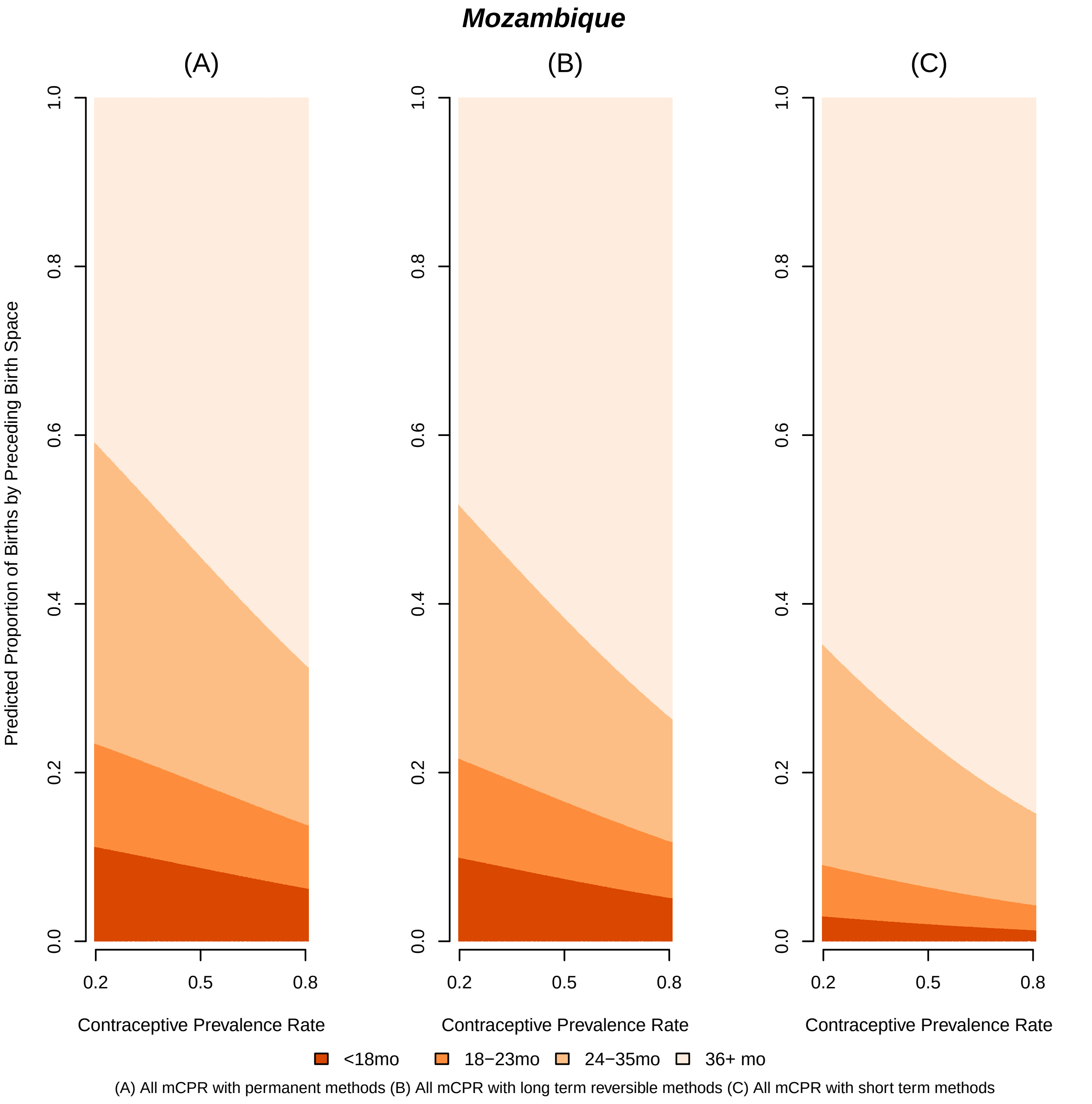
Mozambique
(A)
(B)
(C)
Predicted Proportion of Births by Preceding Birth Space
0.0
0.2
0.4
0.6
0.8
1.0
0.2
0.5
0.8
Contraceptive Prevalence Rate
<18mo
18−23mo
24−35mo
36+ mo
(A) All mCPR with permanent methods (B) All mCPR with long term reversible methods (C) All mCPR with short term methods

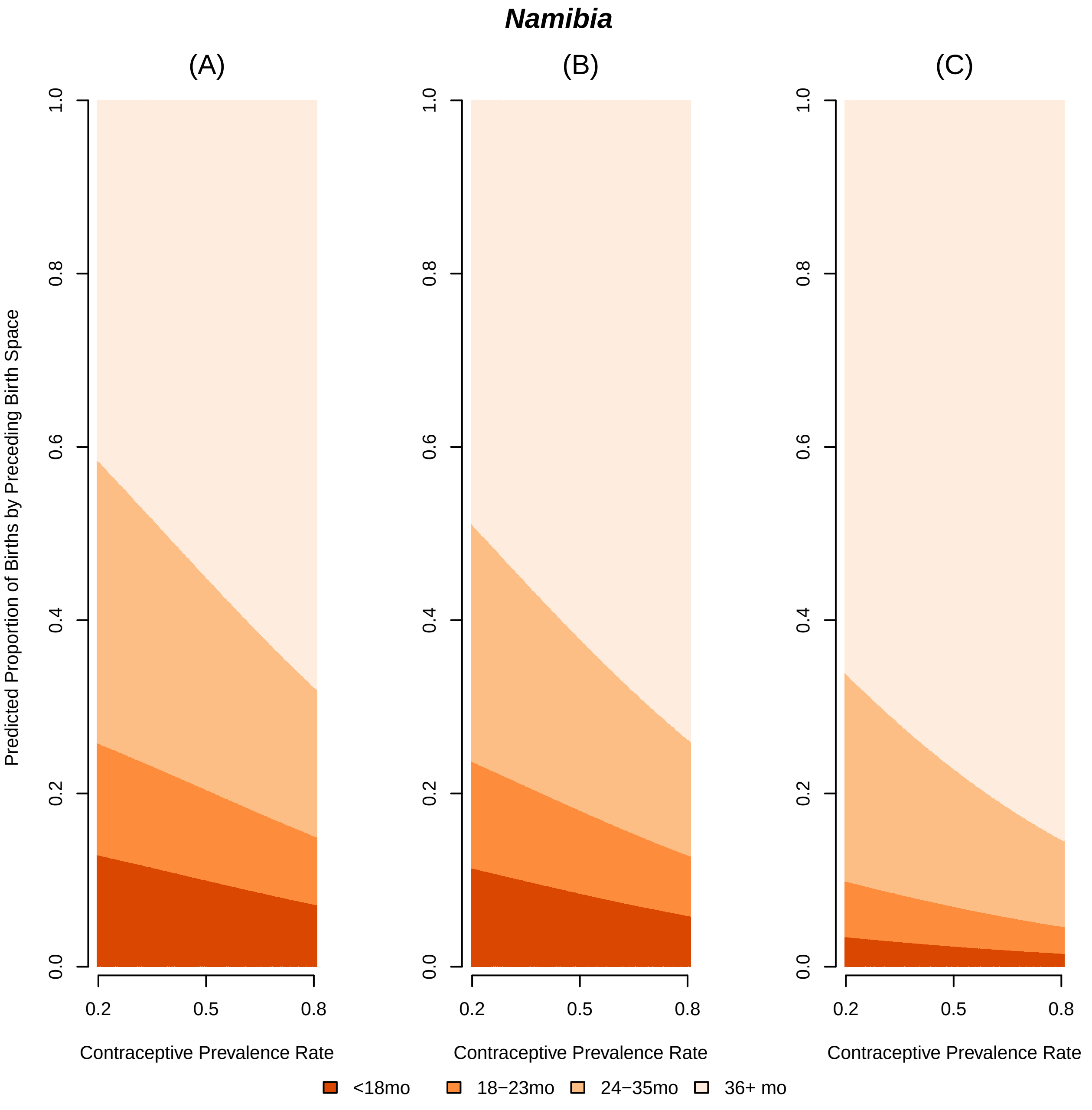

(A) All mCPR with permanent methods (B) All mCPR with long term reversible methods (C) All mCPR with short term methods

Nepal

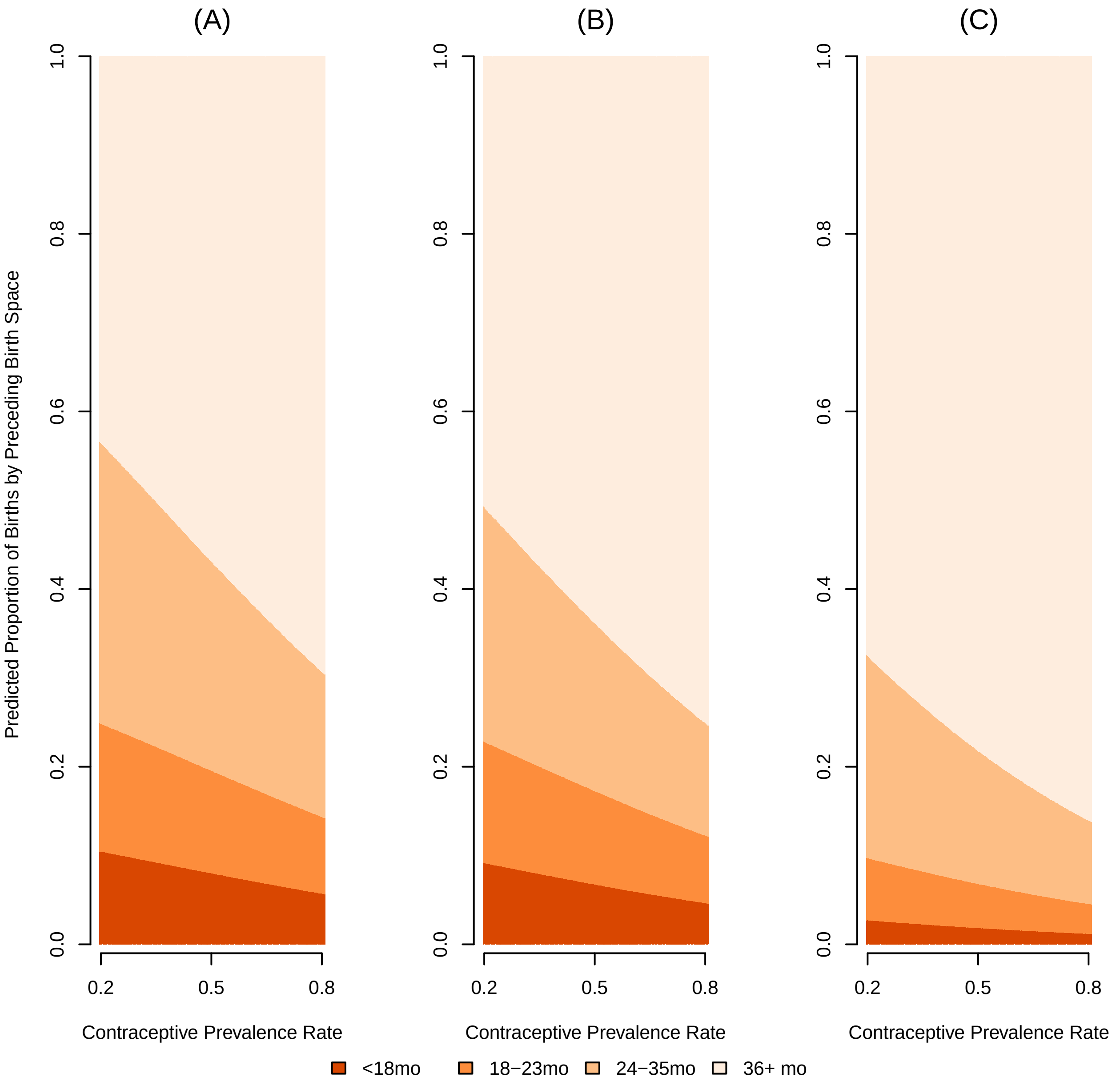

(A) All mCPR with permanent methods (B) All mCPR with long term reversible methods (C) All mCPR with short term methods

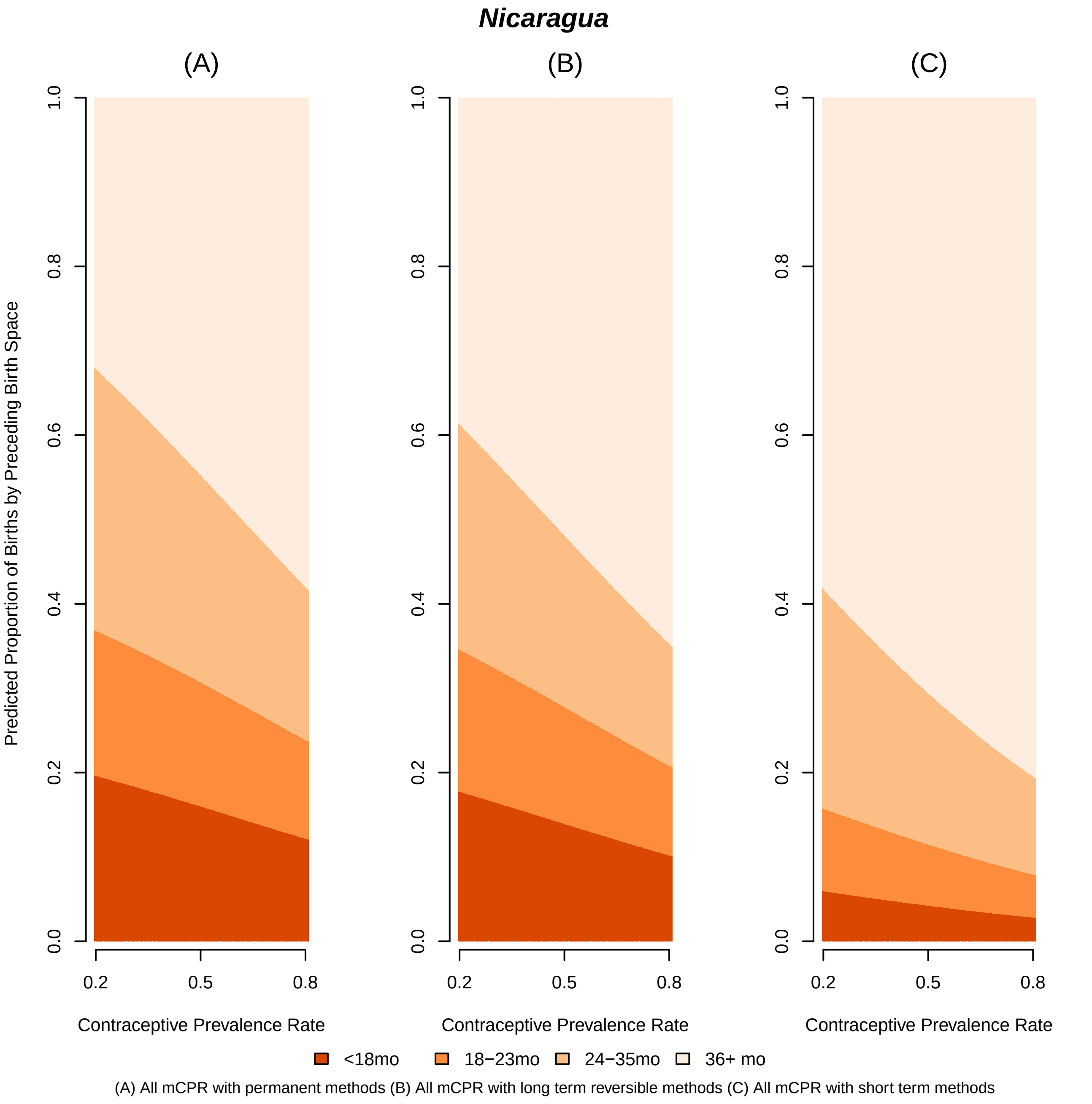
Nicaragua
(A)
(B)
(C)
Predicted Proportion of Births by Preceding Birth Space
0.0
0.2
0.4
0.6
0.8
1.0
0.2
0.5
0.8
Contraceptive Prevalence Rate
<18mo
18−23mo
24−35mo
36+ mo
(A) All mCPR with permanent methods (B) All mCPR with long term reversible methods (C) All mCPR with short term methods

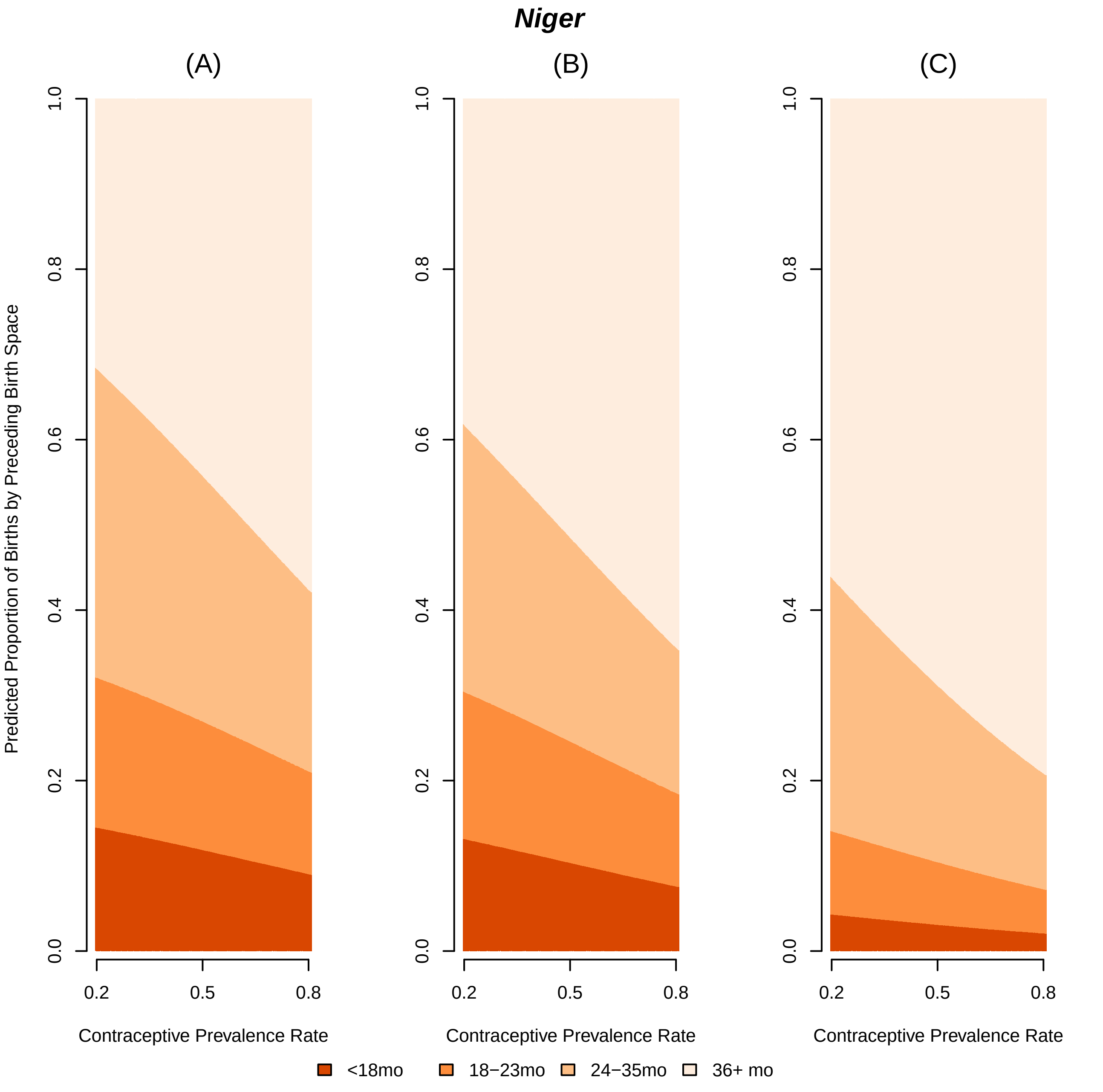

(A) All mCPR with permanent methods (B) All mCPR with long term reversible methods (C) All mCPR with short term methods

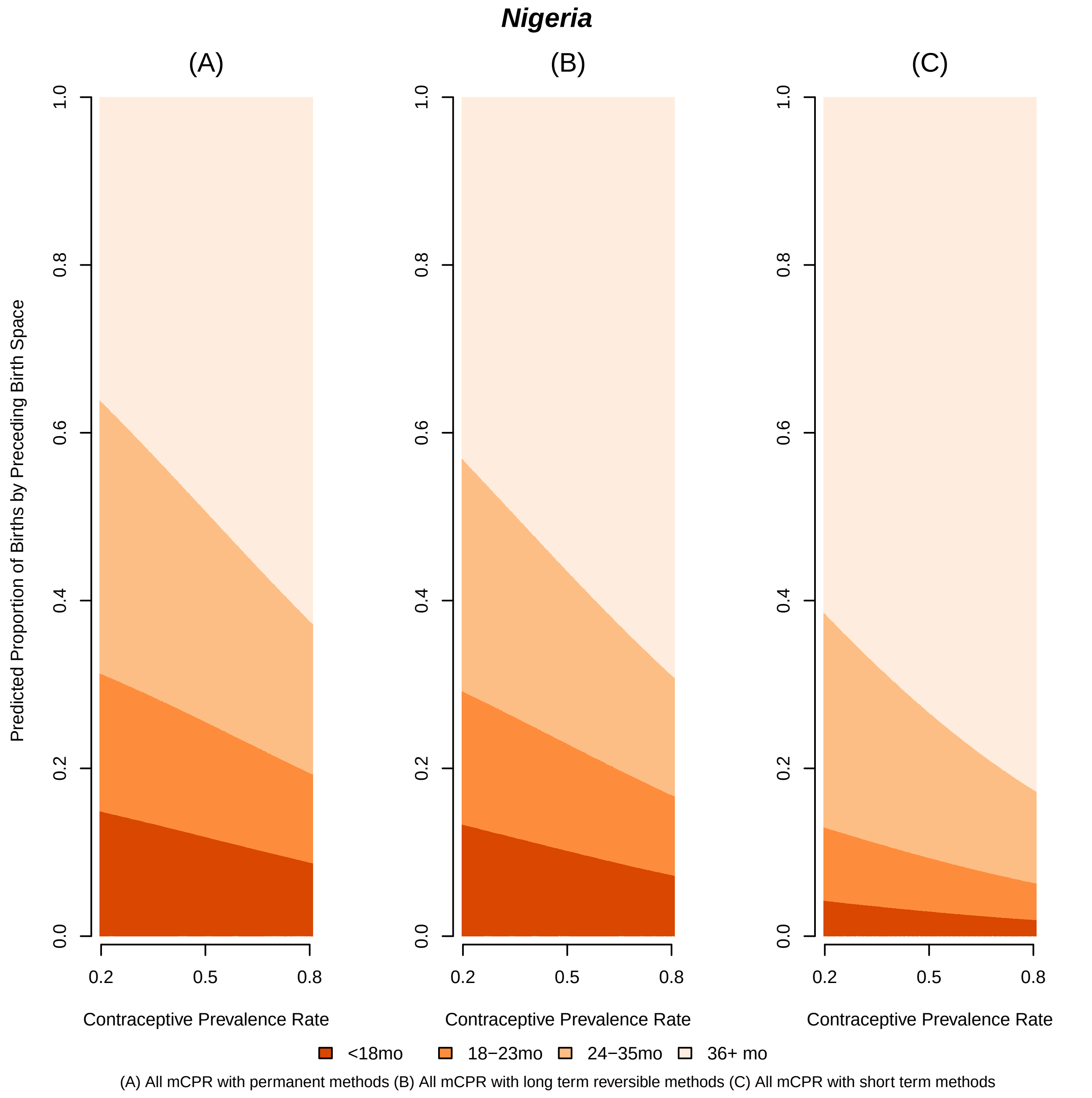
Nigeria
(A)
(B)
(C)
Predicted Proportion of Births by Preceding Birth Space
1.0
0.8
0.6
0.4
0.2
0.0
0.2
0.5
0.8
Contraceptive Prevalence Rate
<18mo
18−23mo
24−35mo
36+ mo
(A) All mCPR with permanent methods (B) All mCPR with long term reversible methods (C) All mCPR with short term methods

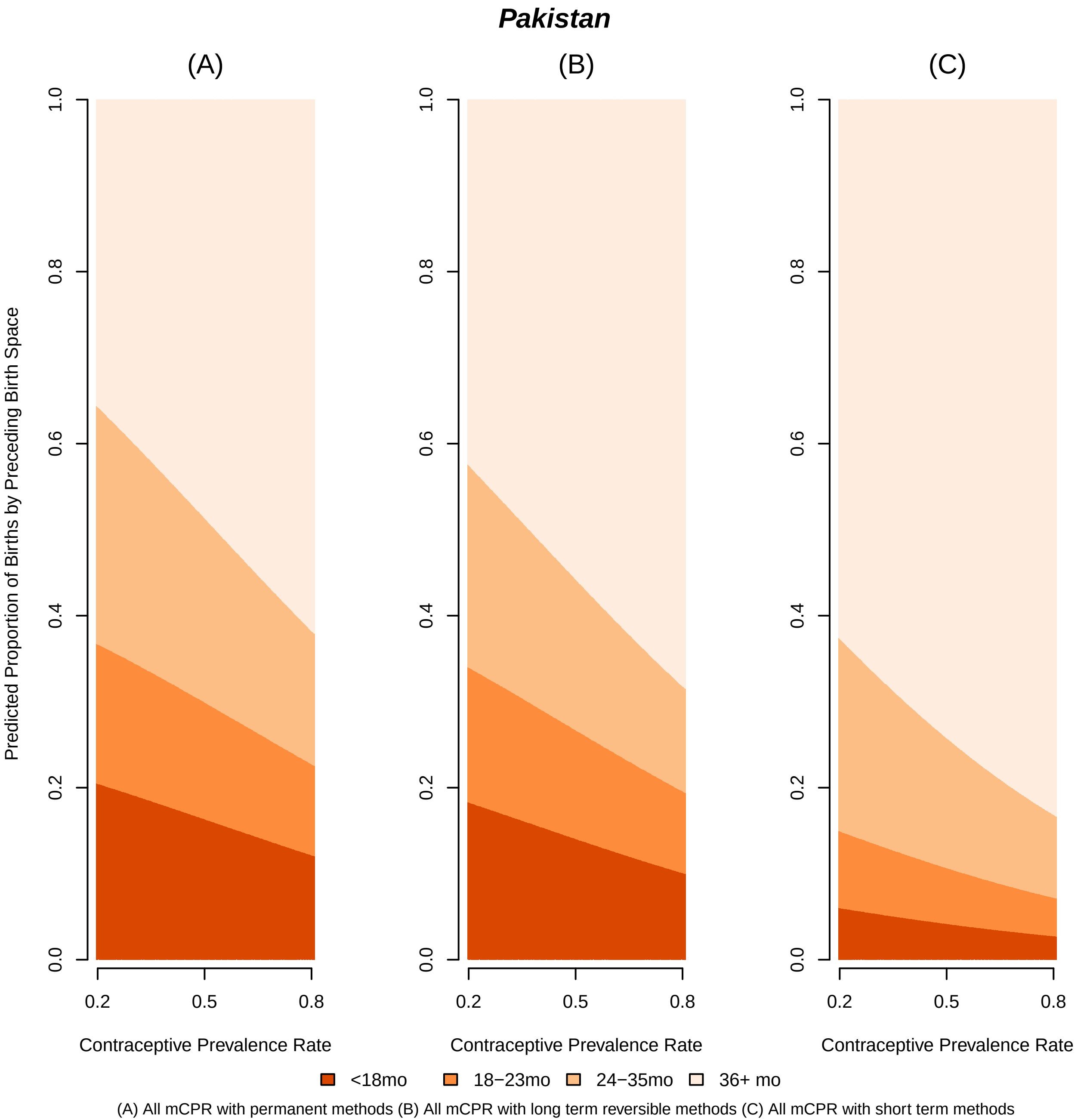

(A) All mCPR with permanent methods (B) All mCPR with long term reversible methods (C) All mCPR with short term methods

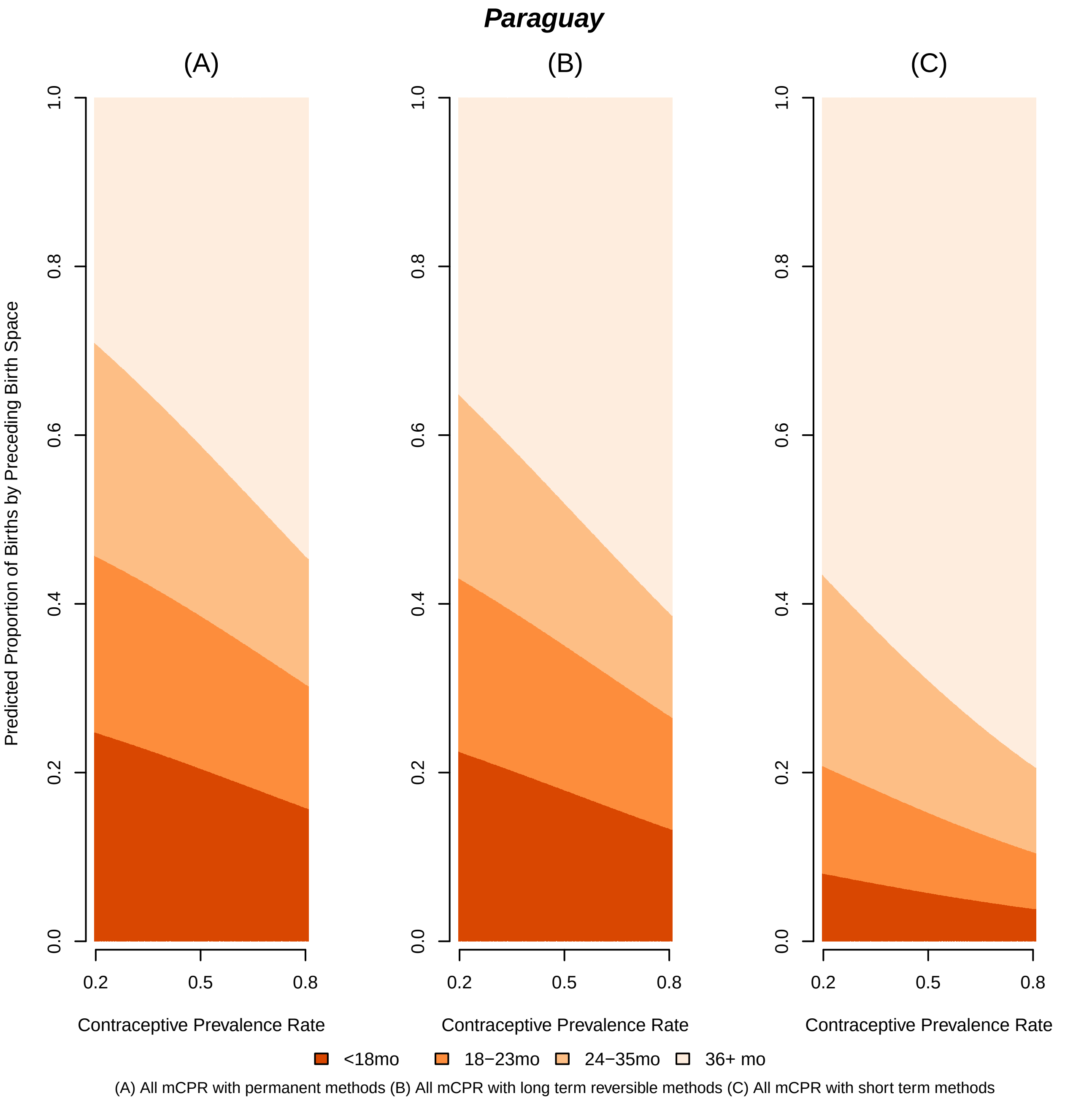
Paraguay
(A)
(B)
(C)
Predicted Proportion of Births by Preceding Birth Space
0.0
0.2
0.4
0.6
0.8
1.0
0.2
0.5
0.8
Contraceptive Prevalence Rate
<18mo
18−23mo
24−35mo
36+ mo
(A) All mCPR with permanent methods (B) All mCPR with long term reversible methods (C) All mCPR with short term methods

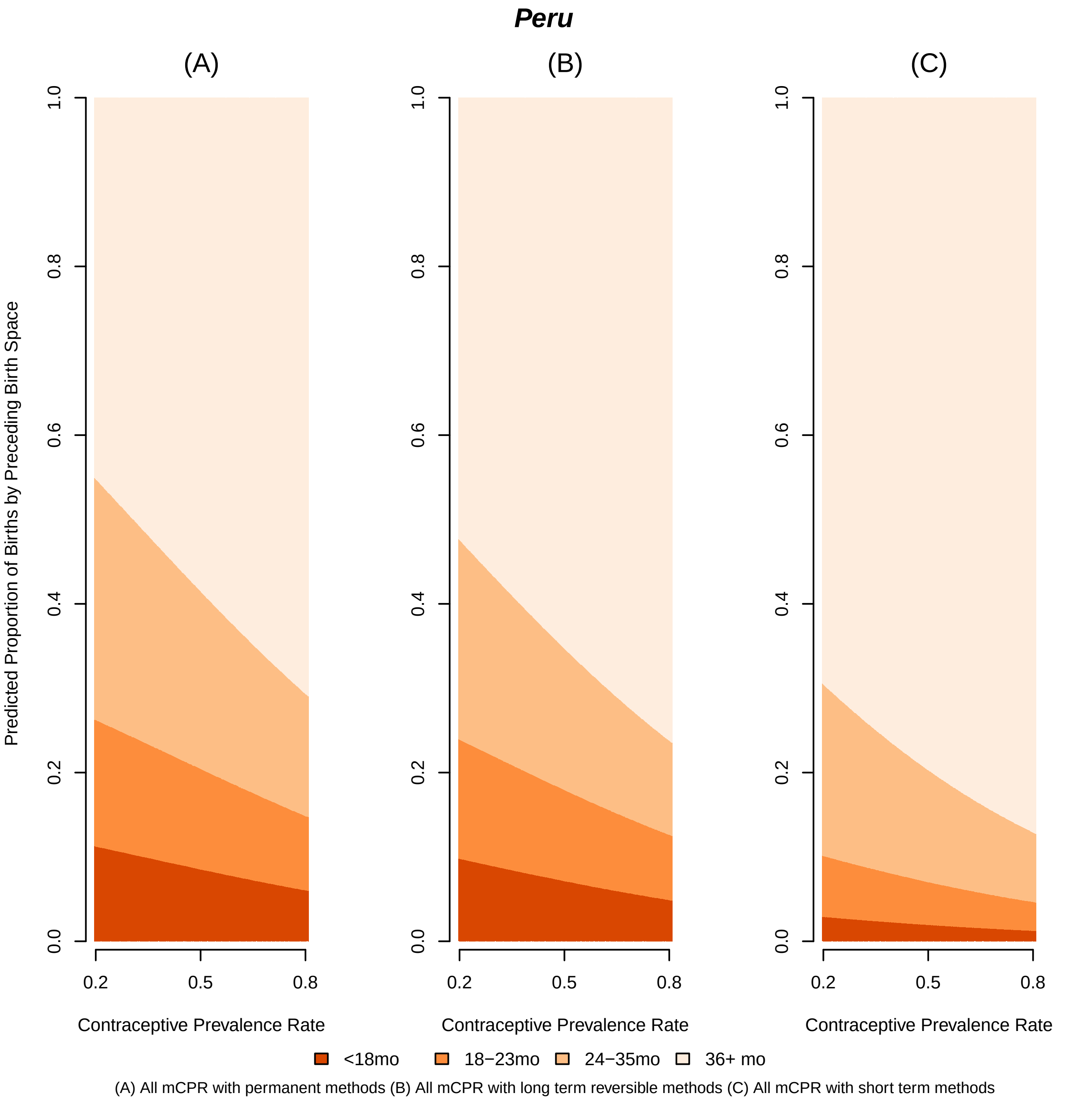
Peru
(A)
(B)
(C)
Predicted Proportion of Births by Preceding Birth Space
0.0
0.2
0.4
0.6
0.8
1.0
0.2
0.5
0.8
Contraceptive Prevalence Rate
<18mo
18−23mo
24−35mo
36+ mo
(A) All mCPR with permanent methods (B) All mCPR with long term reversible methods (C) All mCPR with short term methods

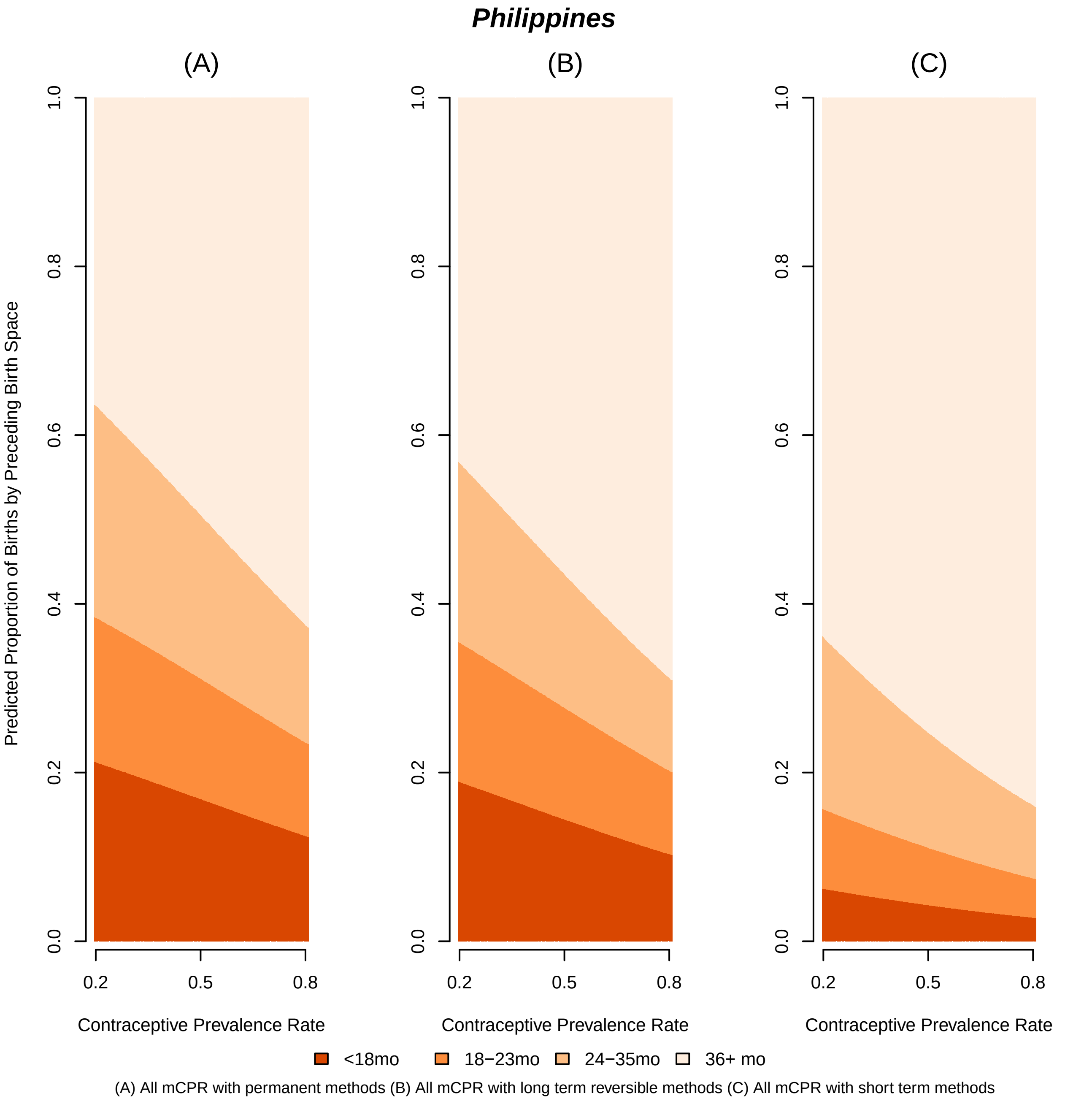
Philippines
(A)
(B)
(C)
Predicted Proportion of Births by Preceding Birth Space
0.0
0.2
0.4
0.6
0.8
1.0
0.2
0.5
0.8
Contraceptive Prevalence Rate
<18mo
18−23mo
24−35mo
36+ mo
(A) All mCPR with permanent methods (B) All mCPR with long term reversible methods (C) All mCPR with short term methods

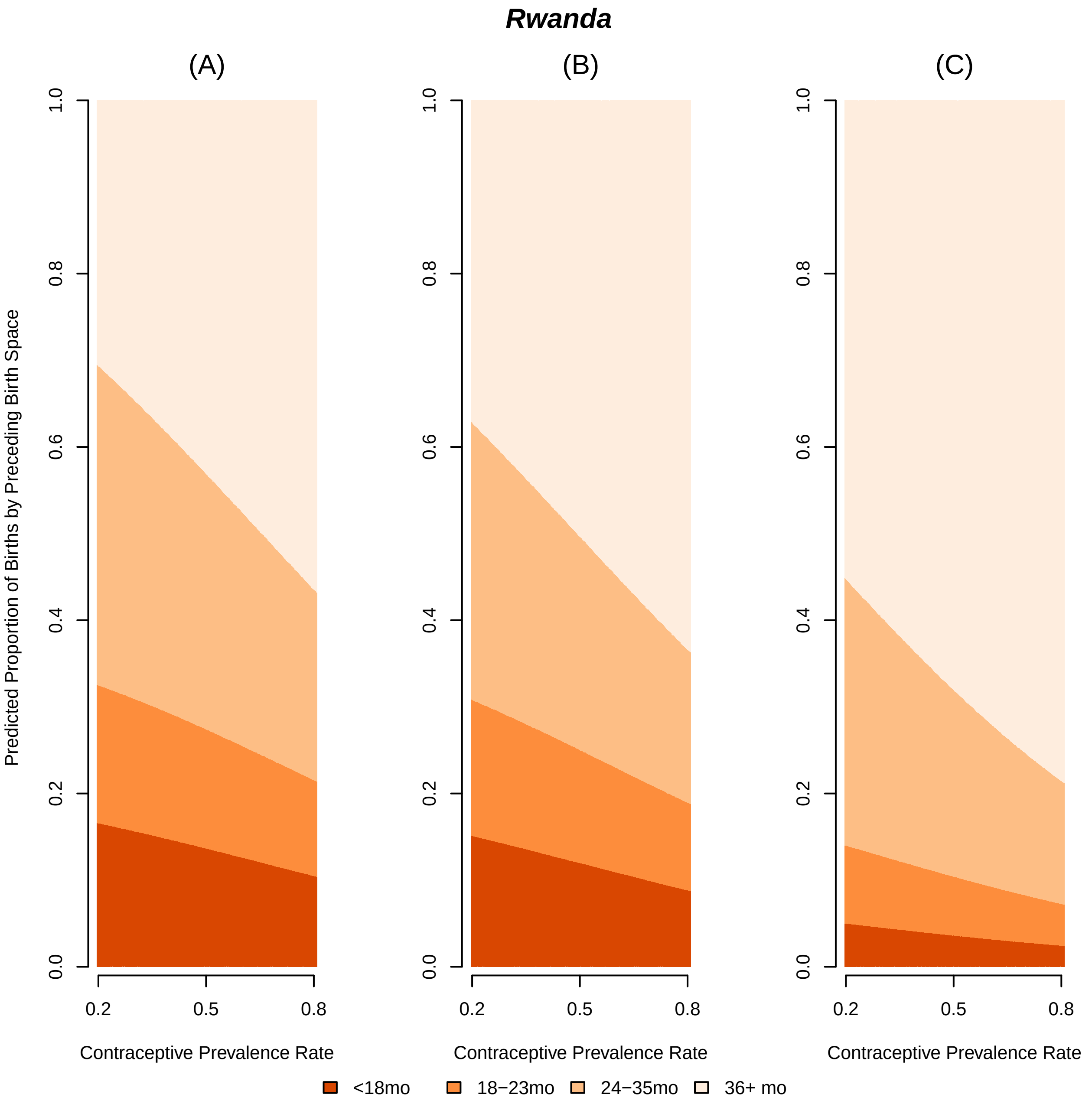

(A) All mCPR with permanent methods (B) All mCPR with long term reversible methods (C) All mCPR with short term methods

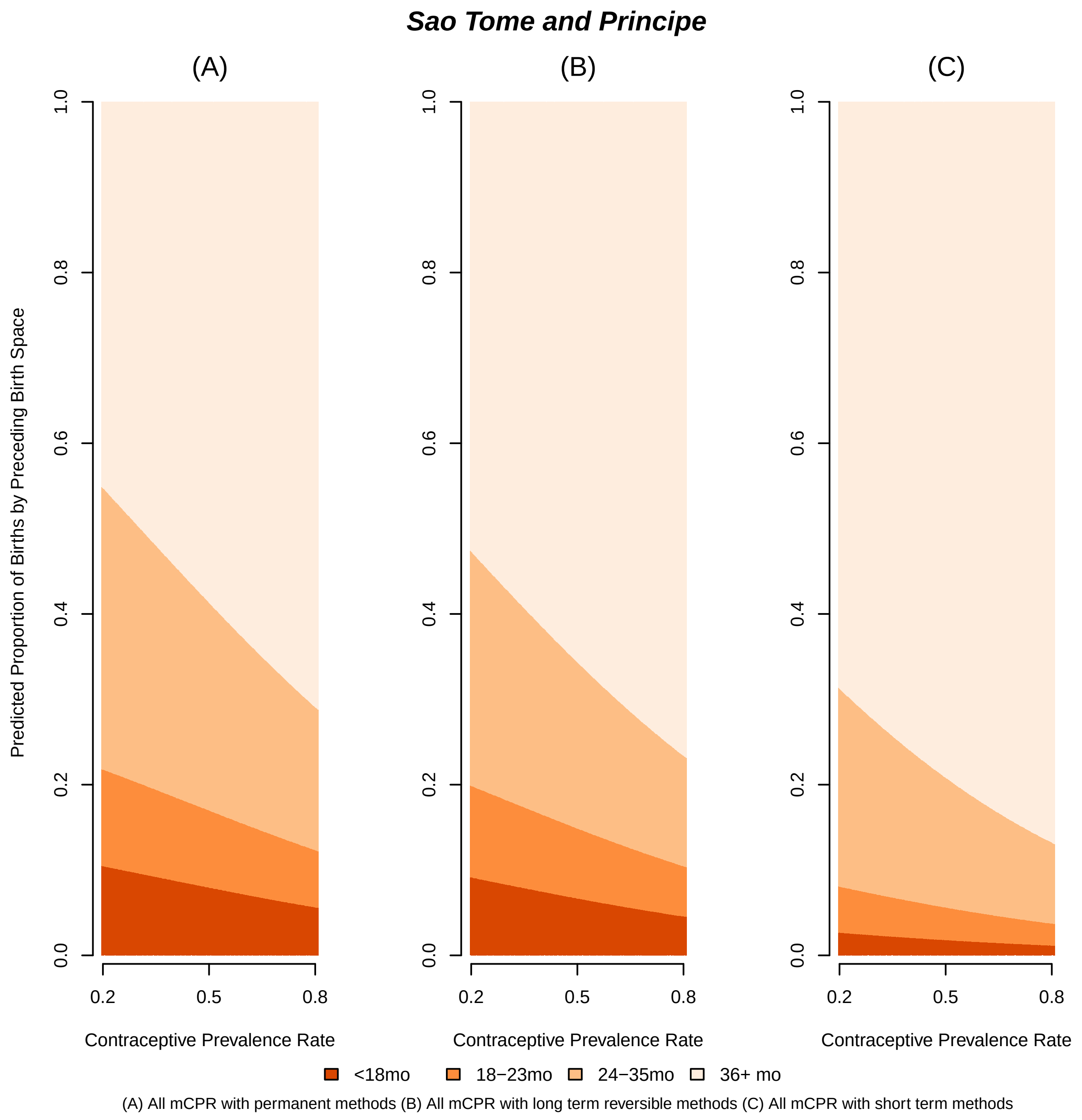

Sao Tome and Principe
(A)
(B)
(C)
Predicted Proportion of Births by Preceding Birth Space
0.0
0.2
0.4
0.6
0.8
1.0
0.2
0.5
0.8
Contraceptive Prevalence Rate
<18mo
18−23mo
24−35mo
36+ mo
(A) All mCPR with permanent methods (B) All mCPR with long term reversible methods (C) All mCPR with short term methods

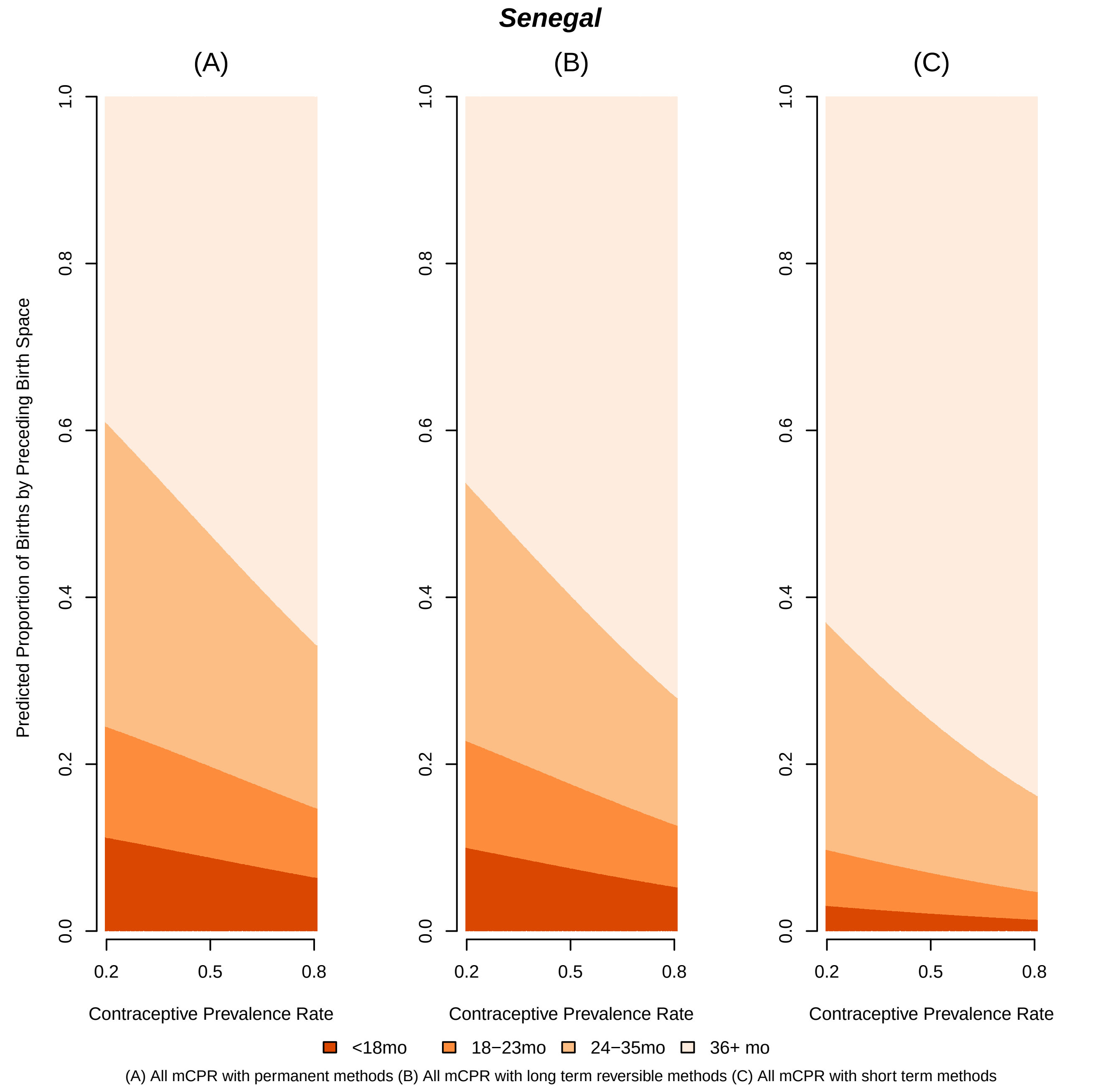
Senegal
(A)
(B)
(C)
Predicted Proportion of Births by Preceding Birth Space
0.0
0.2
0.4
0.6
0.8
1.0
0.2
0.5
0.8
Contraceptive Prevalence Rate
<18mo
18−23mo
24−35mo
36+ mo
(A) All mCPR with permanent methods (B) All mCPR with long term reversible methods (C) All mCPR with short term methods

Sierra Leone

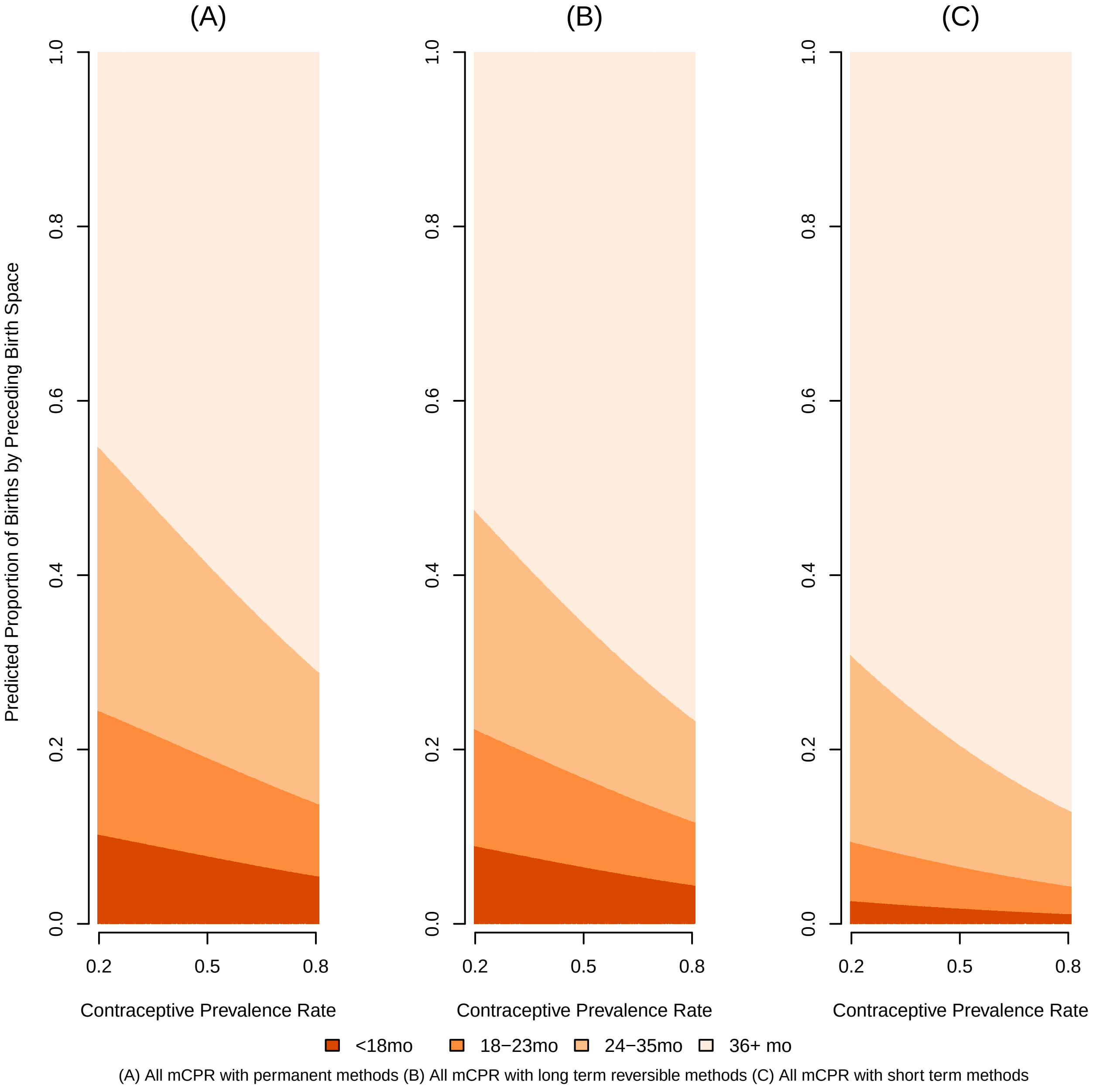

(A) All mCPR with permanent methods (B) All mCPR with long term reversible methods (C) All mCPR with short term methods

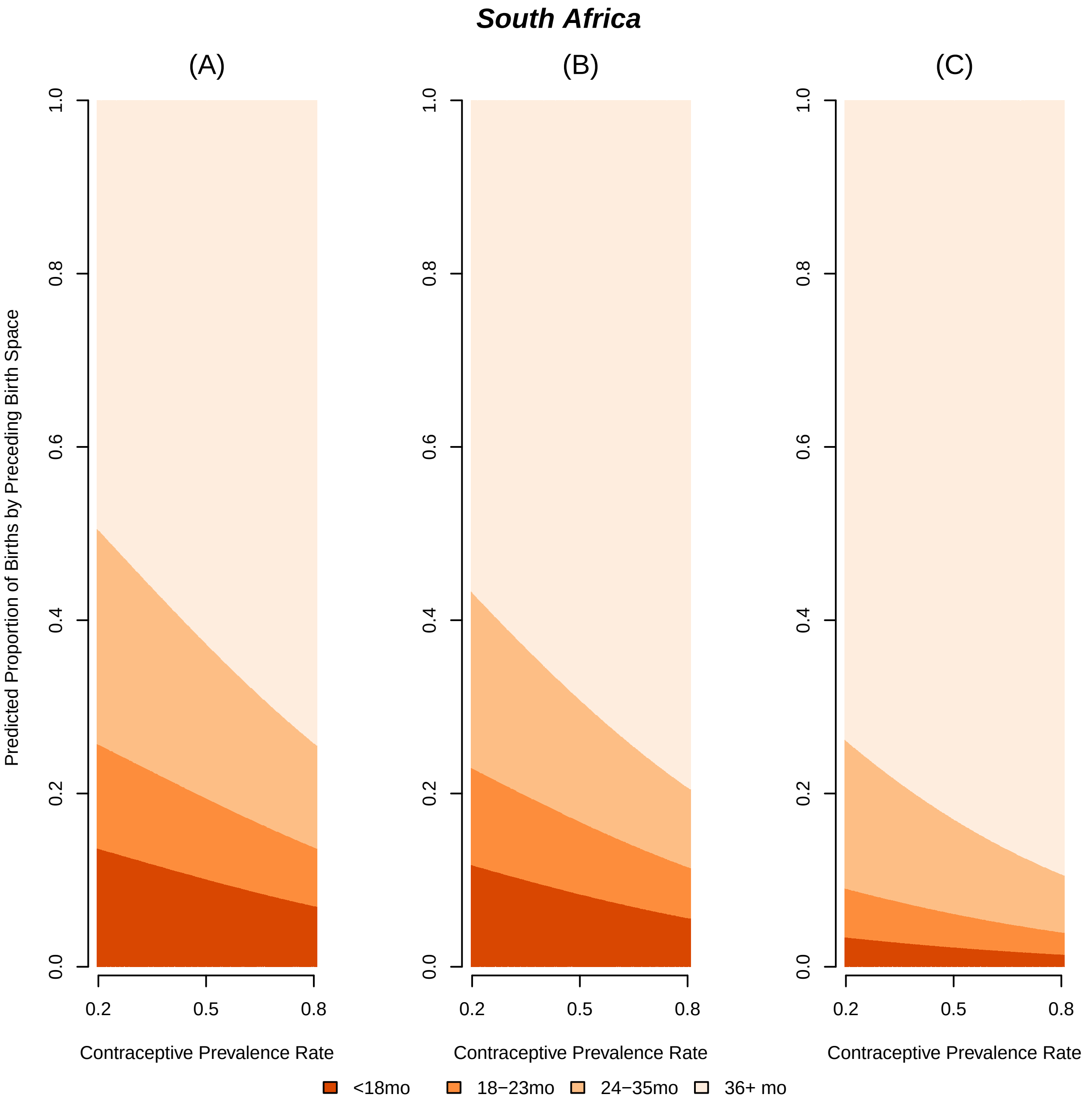

(A) All mCPR with permanent methods (B) All mCPR with long term reversible methods (C) All mCPR with short term methods

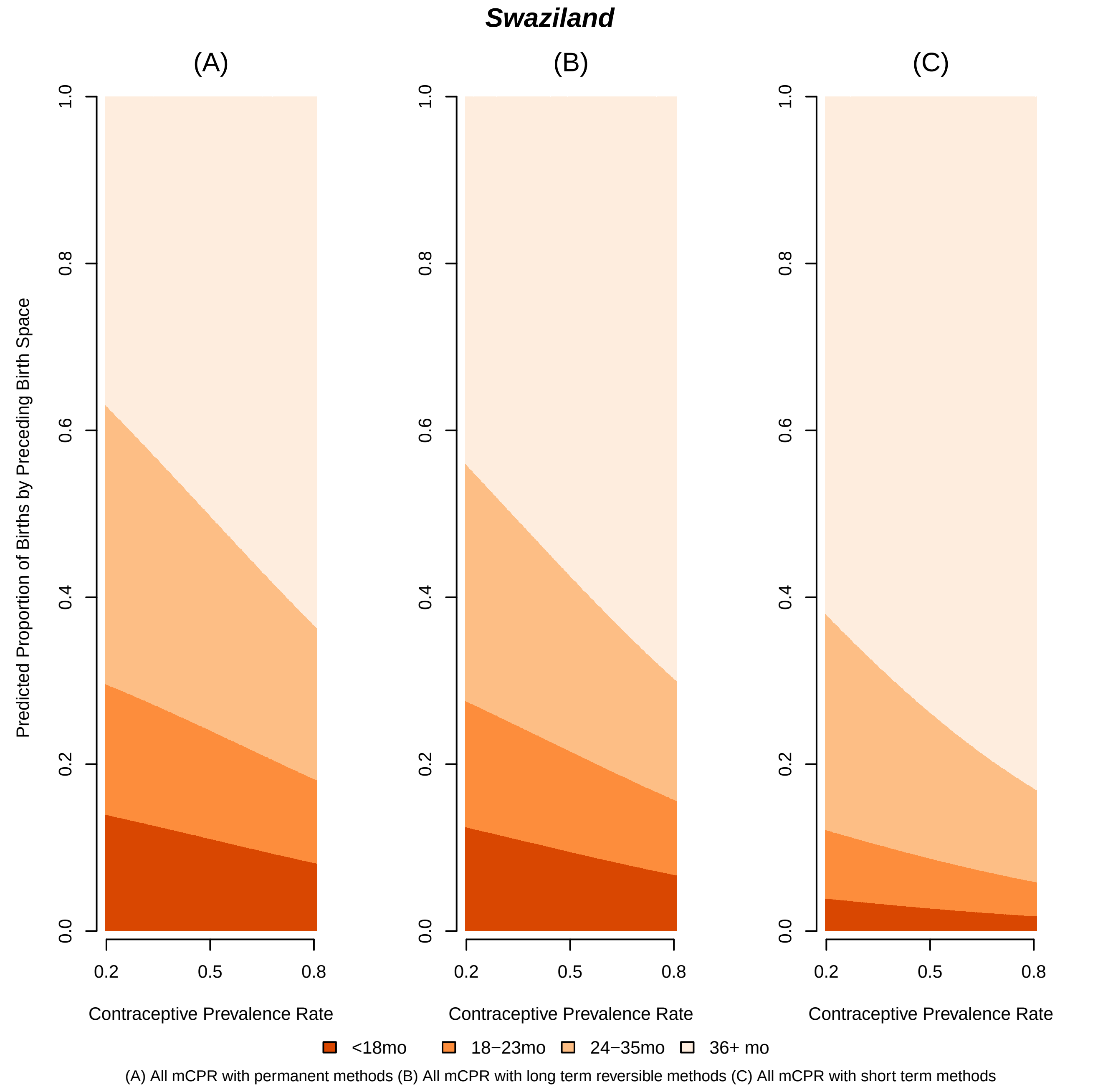
Swaziland
(A)
(B)
(C)
Predicted Proportion of Births by Preceding Birth Space
0.0
0.2
0.4
0.6
0.8
1.0
0.2
0.5
0.8
Contraceptive Prevalence Rate
<18mo
18−23mo
24−35mo
36+ mo
(A) All mCPR with permanent methods (B) All mCPR with long term reversible methods (C) All mCPR with short term methods

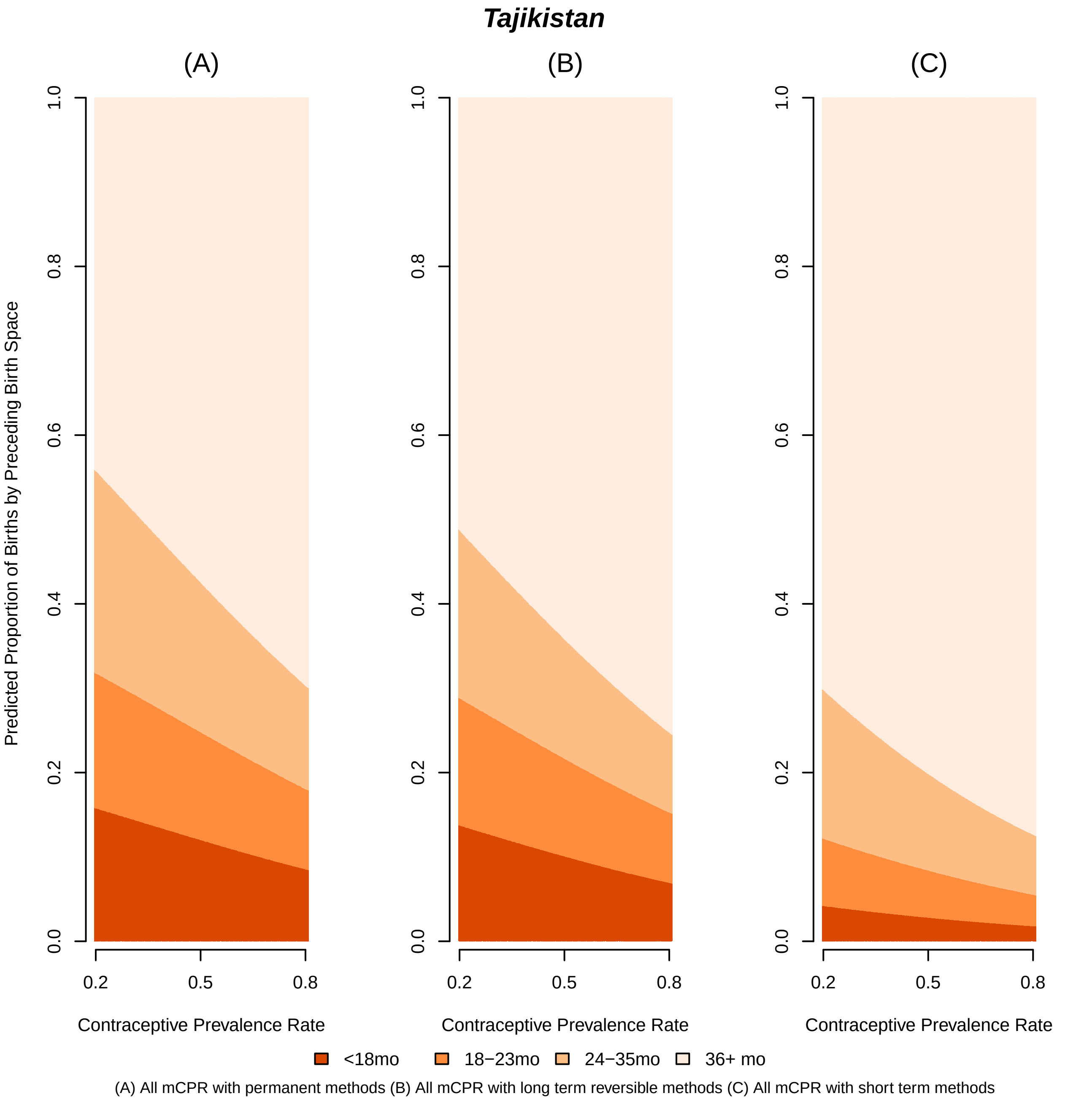
Tajikistan
(A)
(B)
(C)
Predicted Proportion of Births by Preceding Birth Space
0.0
0.2
0.4
0.6
0.8
1.0
0.2
0.5
0.8
Contraceptive Prevalence Rate
<18mo
18−23mo
24−35mo
36+ mo
(A) All mCPR with permanent methods (B) All mCPR with long term reversible methods (C) All mCPR with short term methods

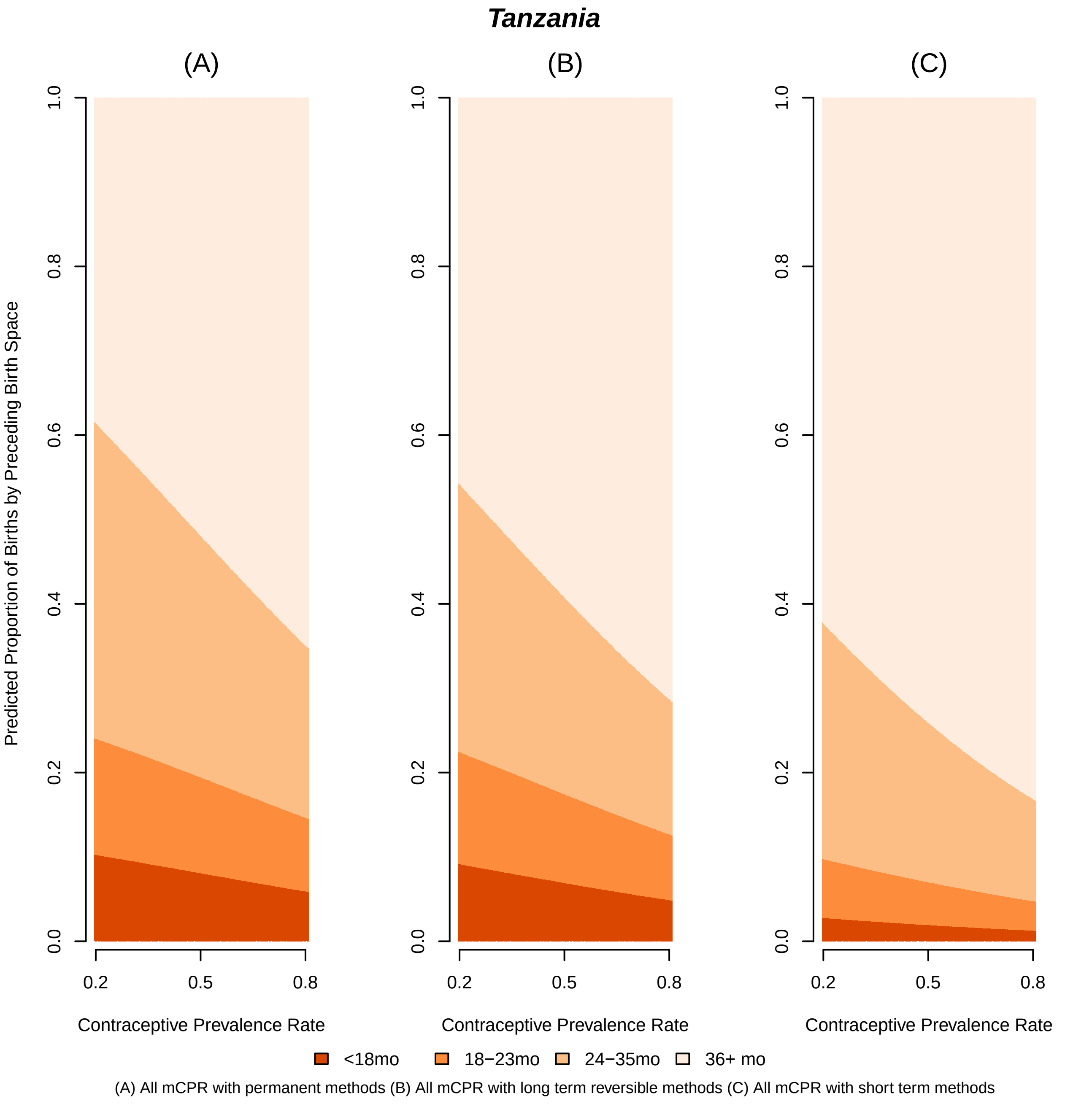
Tanzania
(A)
(B)
(C)
Predicted Proportion of Births by Preceding Birth Space
1.0
0.8
0.6
0.4
0.2
0.0
0.2
0.5
0.8
Contraceptive Prevalence Rate
<18mo
18−23mo
24−35mo
36+ mo
(A) All mCPR with permanent methods (B) All mCPR with long term reversible methods (C) All mCPR with short term methods

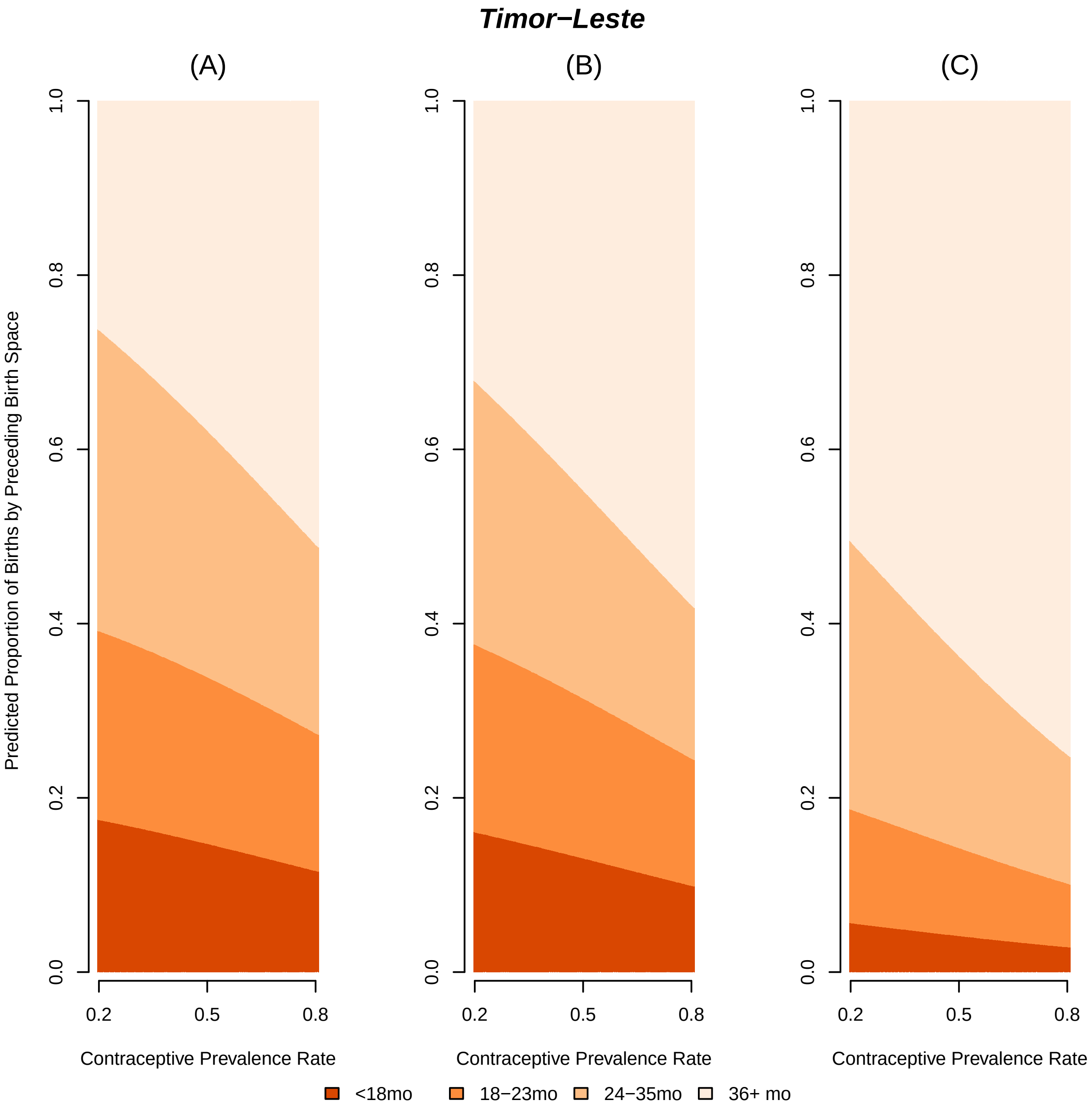

(A) All mCPR with permanent methods (B) All mCPR with long term reversible methods (C) All mCPR with short term methods

Togo

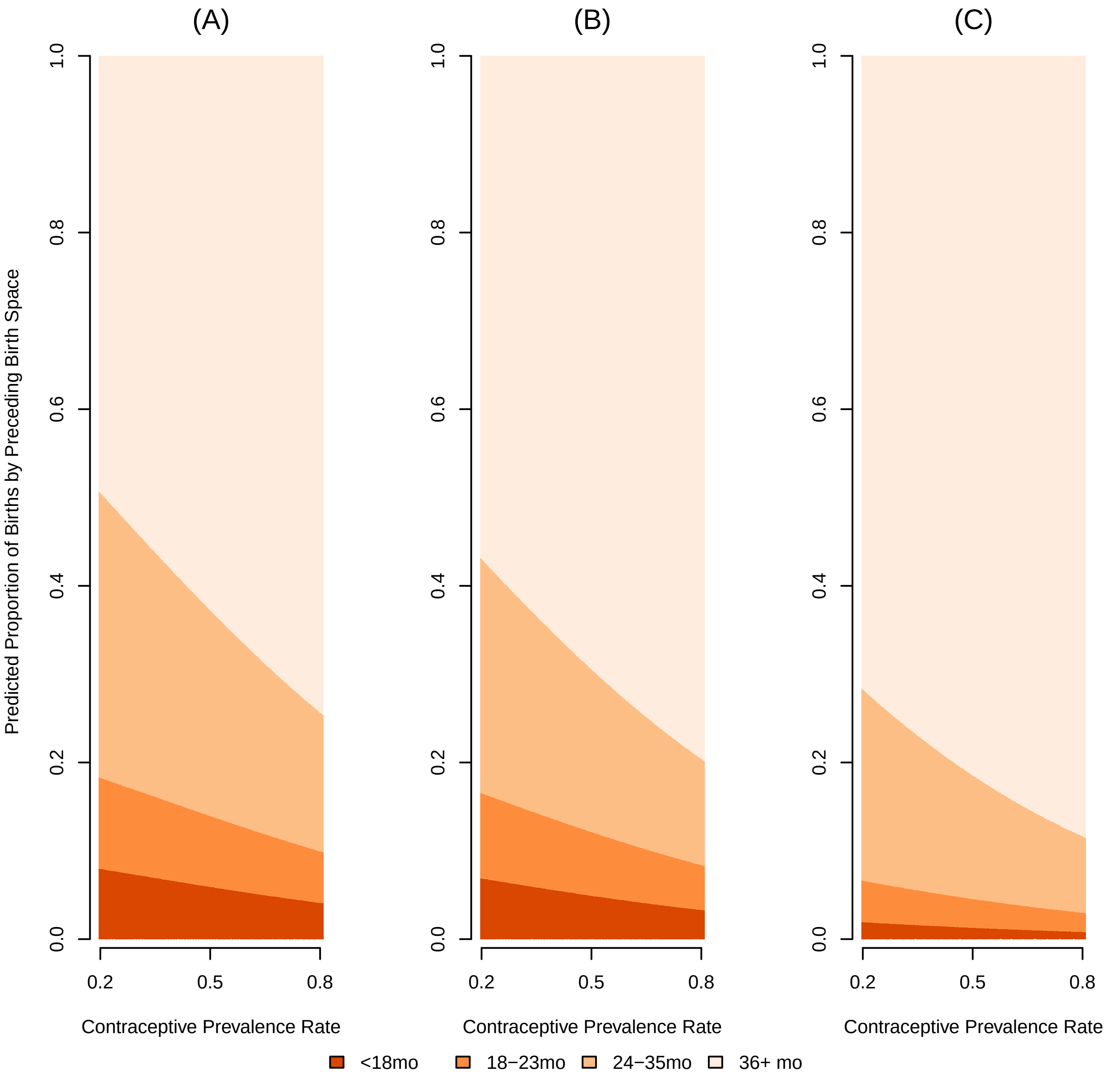

(A) All mCPR with permanent methods (B) All mCPR with long term reversible methods (C) All mCPR with short term methods

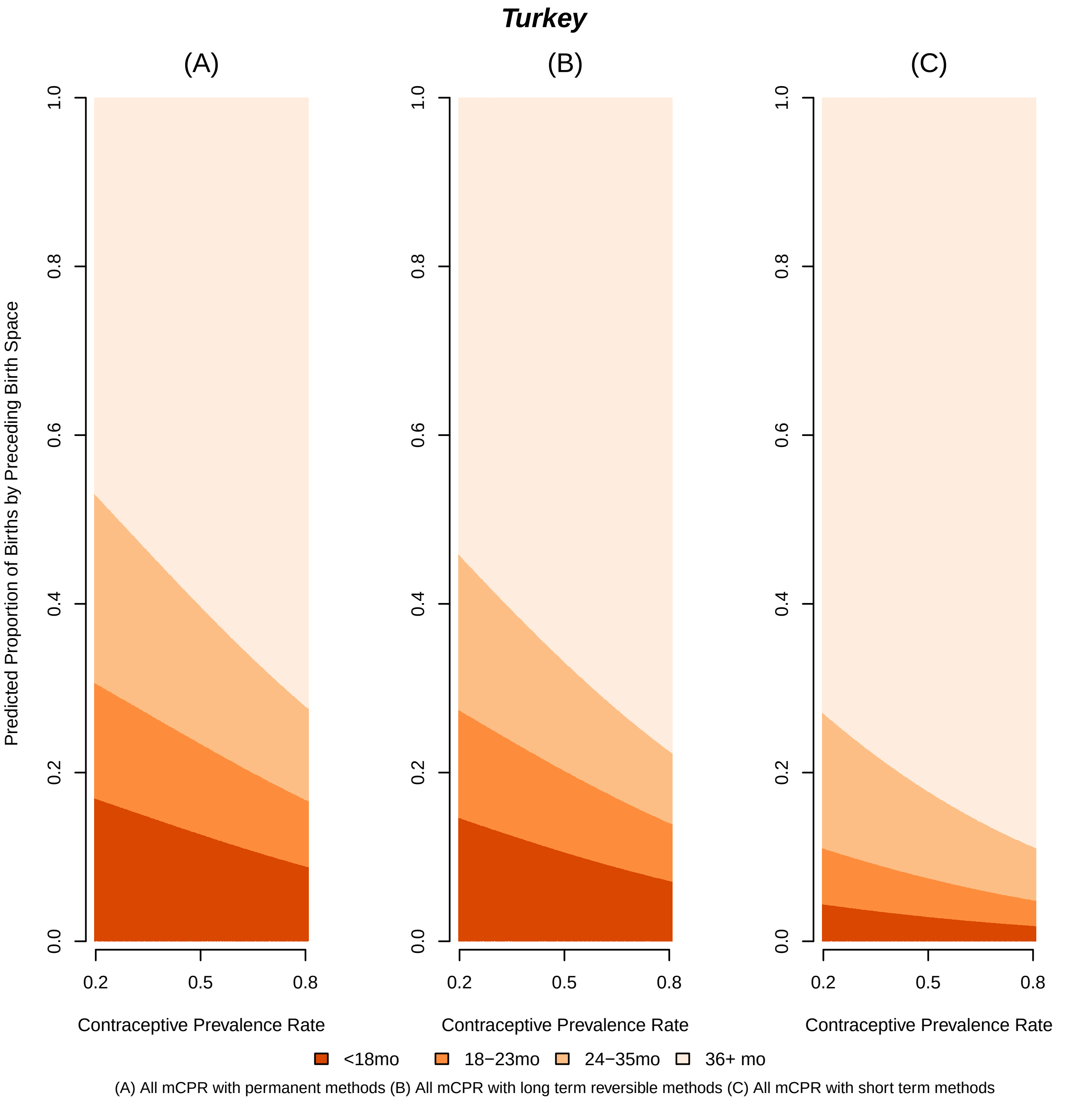
Turkey
(A)
(B)
(C)
Predicted Proportion of Births by Preceding Birth Space
0.0
0.2
0.4
0.6
0.8
1.0
0.2
0.5
0.8
Contraceptive Prevalence Rate
<18mo
18−23mo
24−35mo
36+ mo
(A) All mCPR with permanent methods (B) All mCPR with long term reversible methods (C) All mCPR with short term methods

Uganda

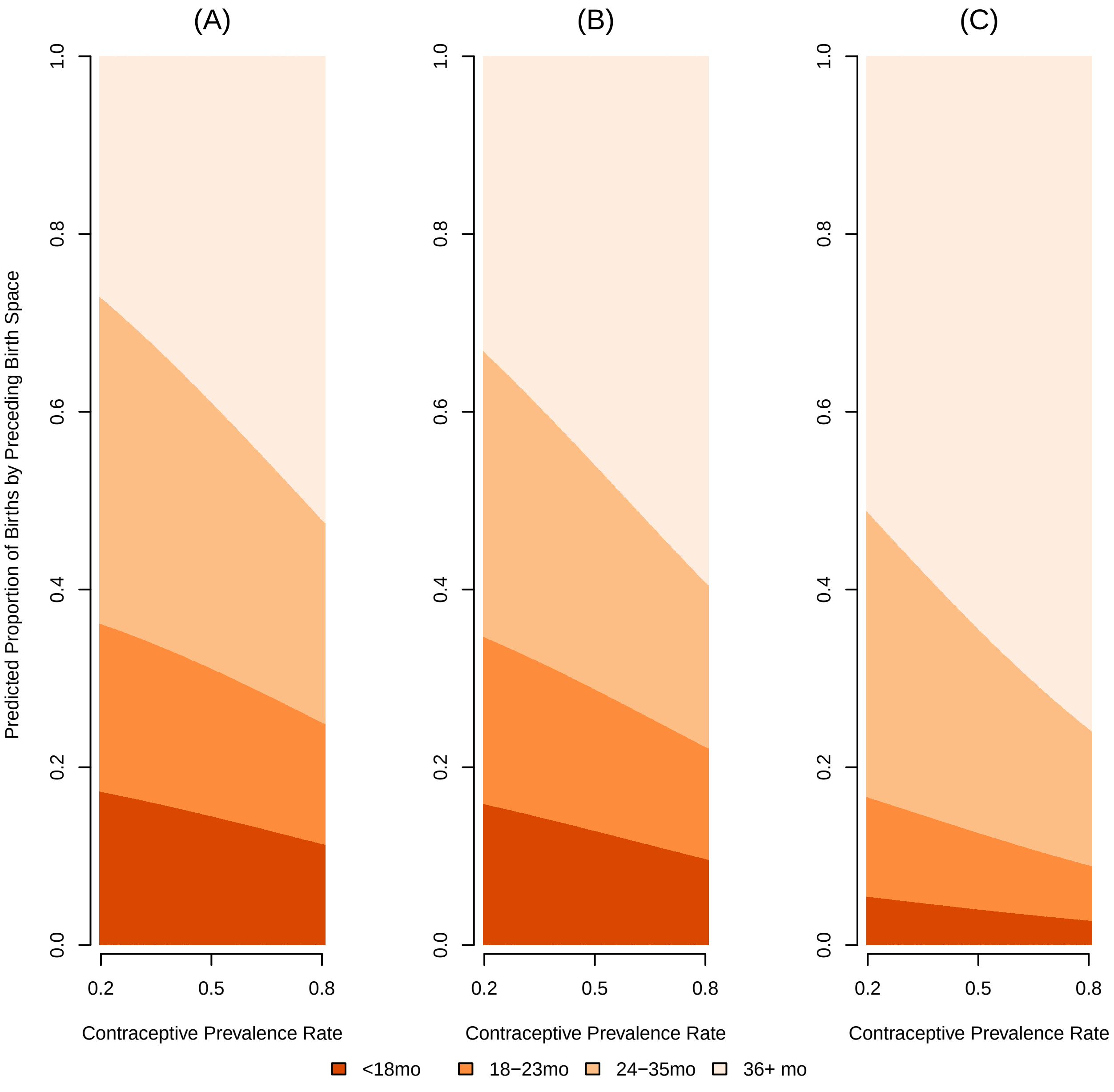

(A) All mCPR with permanent methods (B) All mCPR with long term reversible methods (C) All mCPR with short term methods

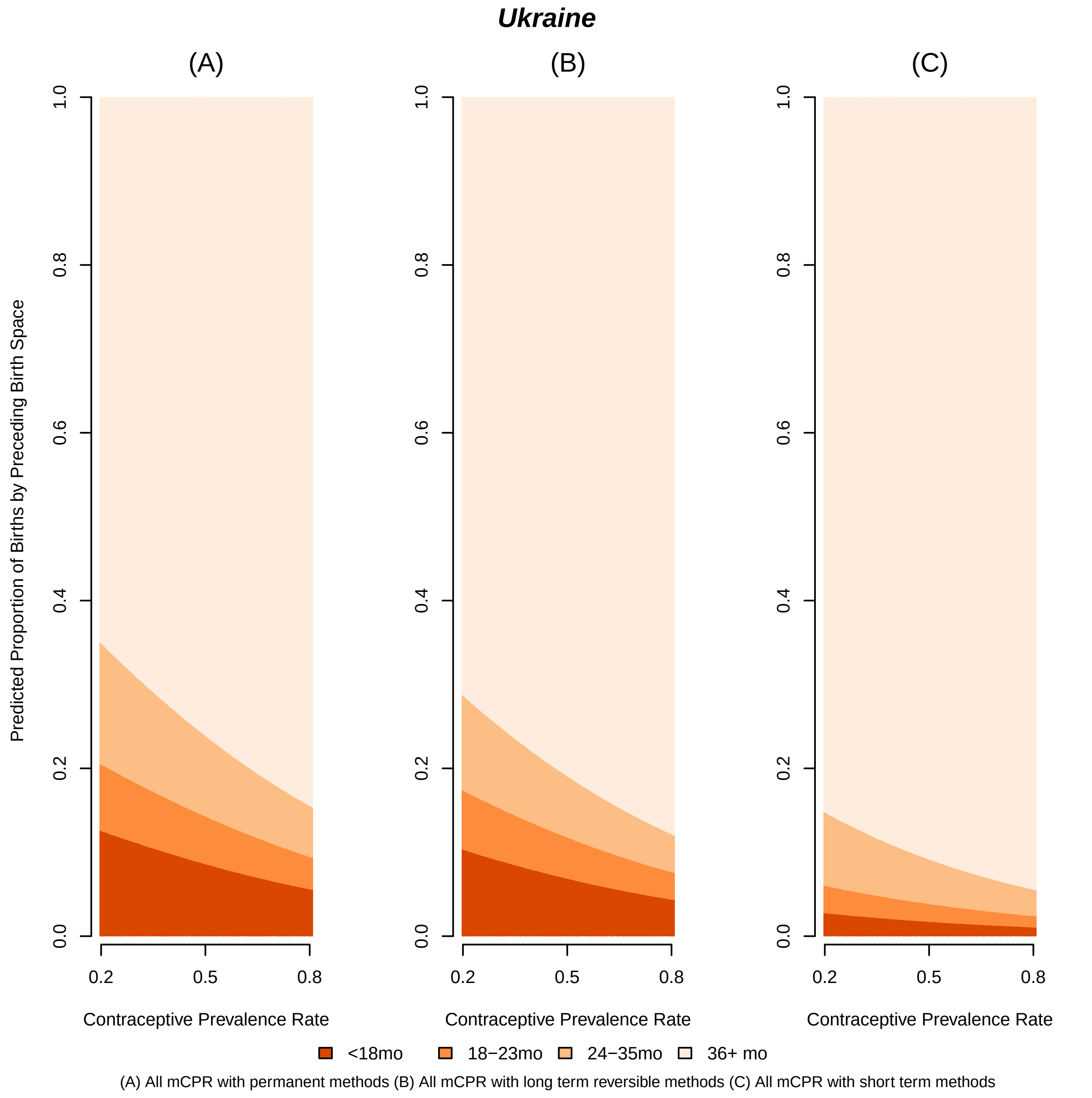

Ukraine
(A)
(B)
(C)
Predicted Proportion of Births by Preceding Birth Space
0.0
0.2
0.4
0.6
0.8
1.0
0.2
0.5
0.8
Contraceptive Prevalence Rate
<18mo
18−23mo
24−35mo
36+ mo
(A) All mCPR with permanent methods (B) All mCPR with long term reversible methods (C) All mCPR with short term methods

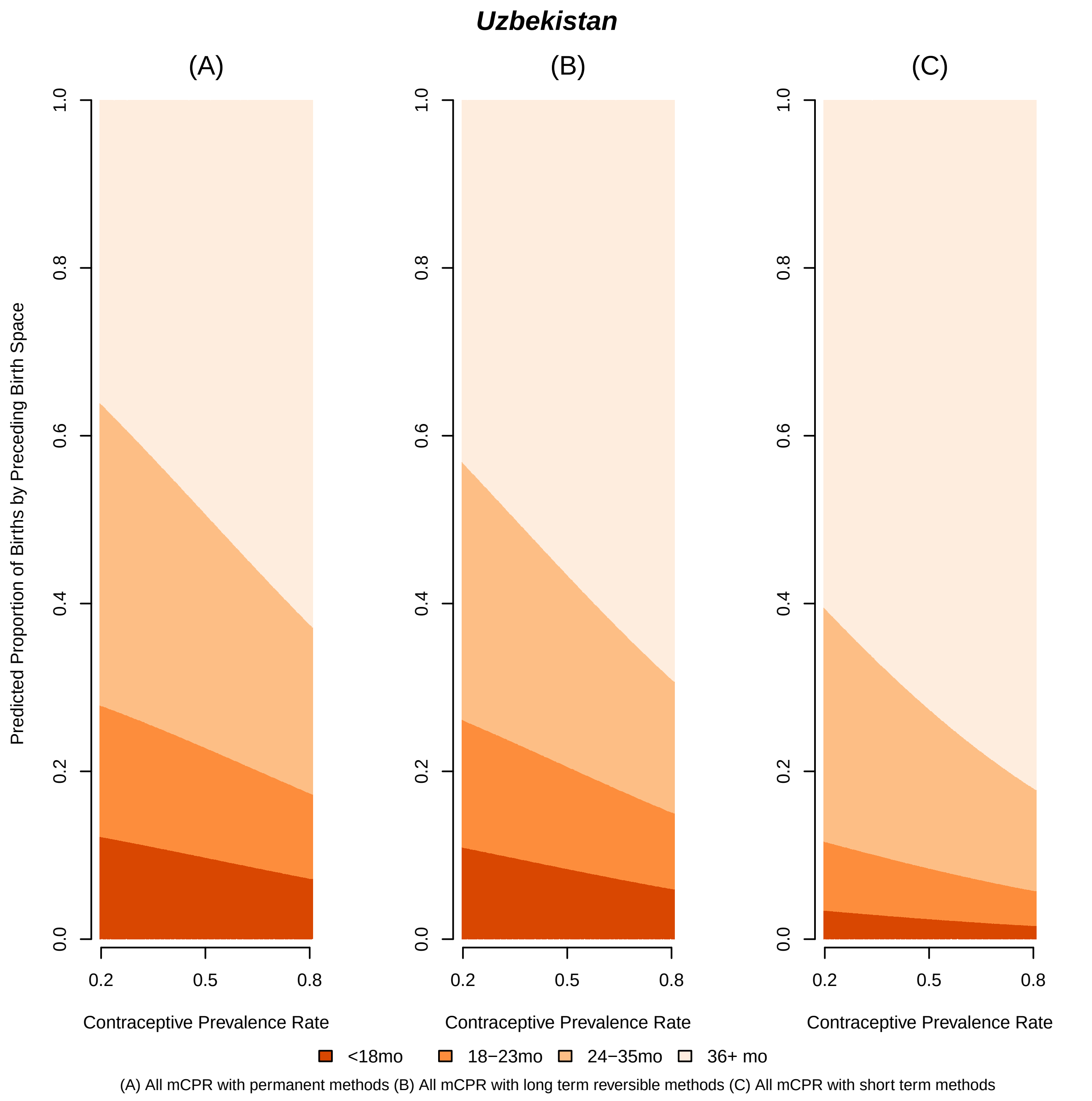
Uzbekistan
(A)
(B)
(C)
Predicted Proportion of Births by Preceding Birth Space
0.0
0.2
0.4
0.6
0.8
1.0
0.2
0.5
0.8
Contraceptive Prevalence Rate
<18mo
18−23mo
24−35mo
36+ mo
(A) All mCPR with permanent methods (B) All mCPR with long term reversible methods (C) All mCPR with short term methods

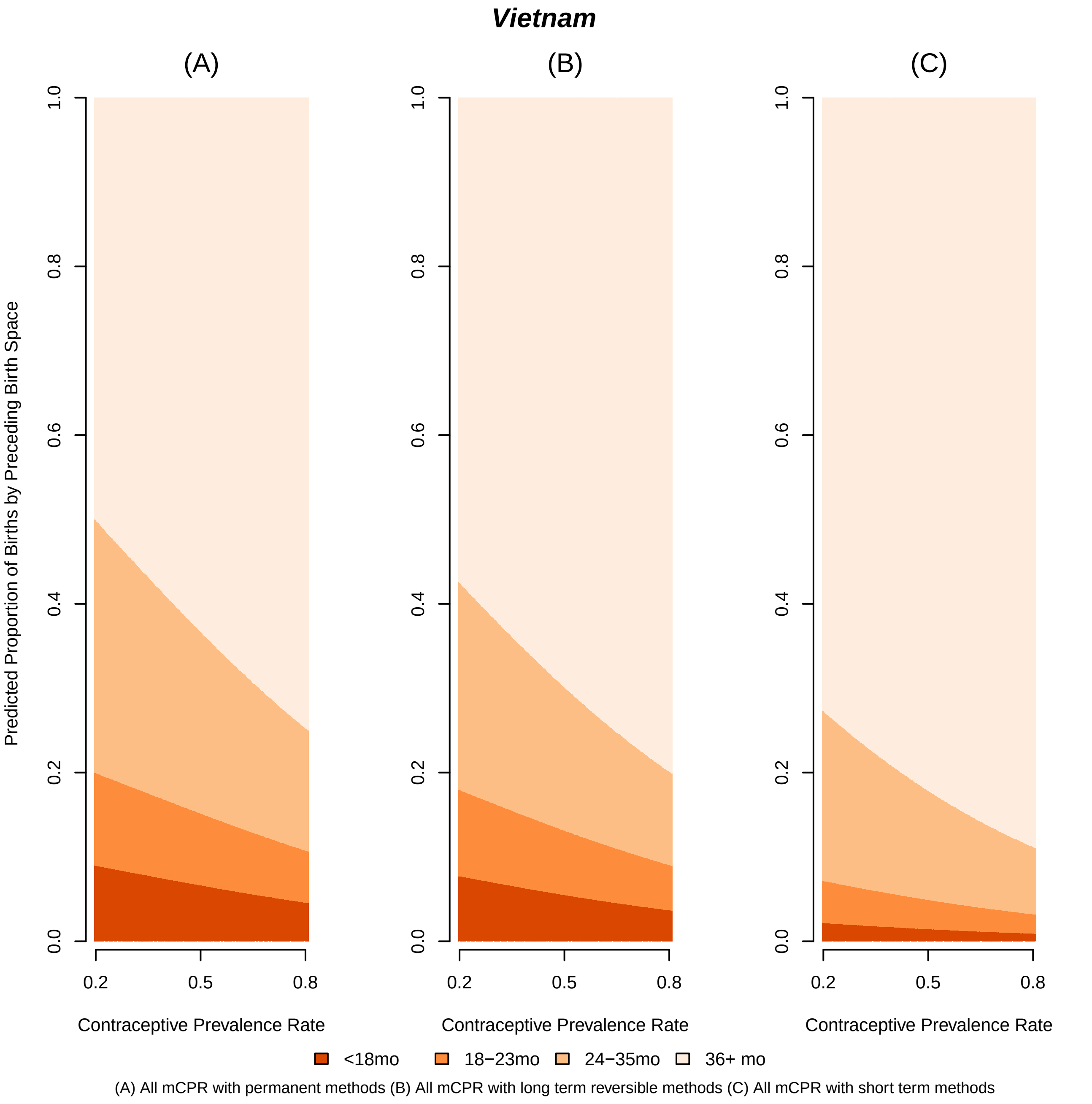
Vietnam
(A)
(B)
(C)
Predicted Proportion of Births by Preceding Birth Space
0.0
0.2
0.4
0.6
0.8
1.0
0.2
0.5
0.8
Contraceptive Prevalence Rate
<18mo
18−23mo
24−35mo
36+ mo
(A) All mCPR with permanent methods (B) All mCPR with long term reversible methods (C) All mCPR with short term methods

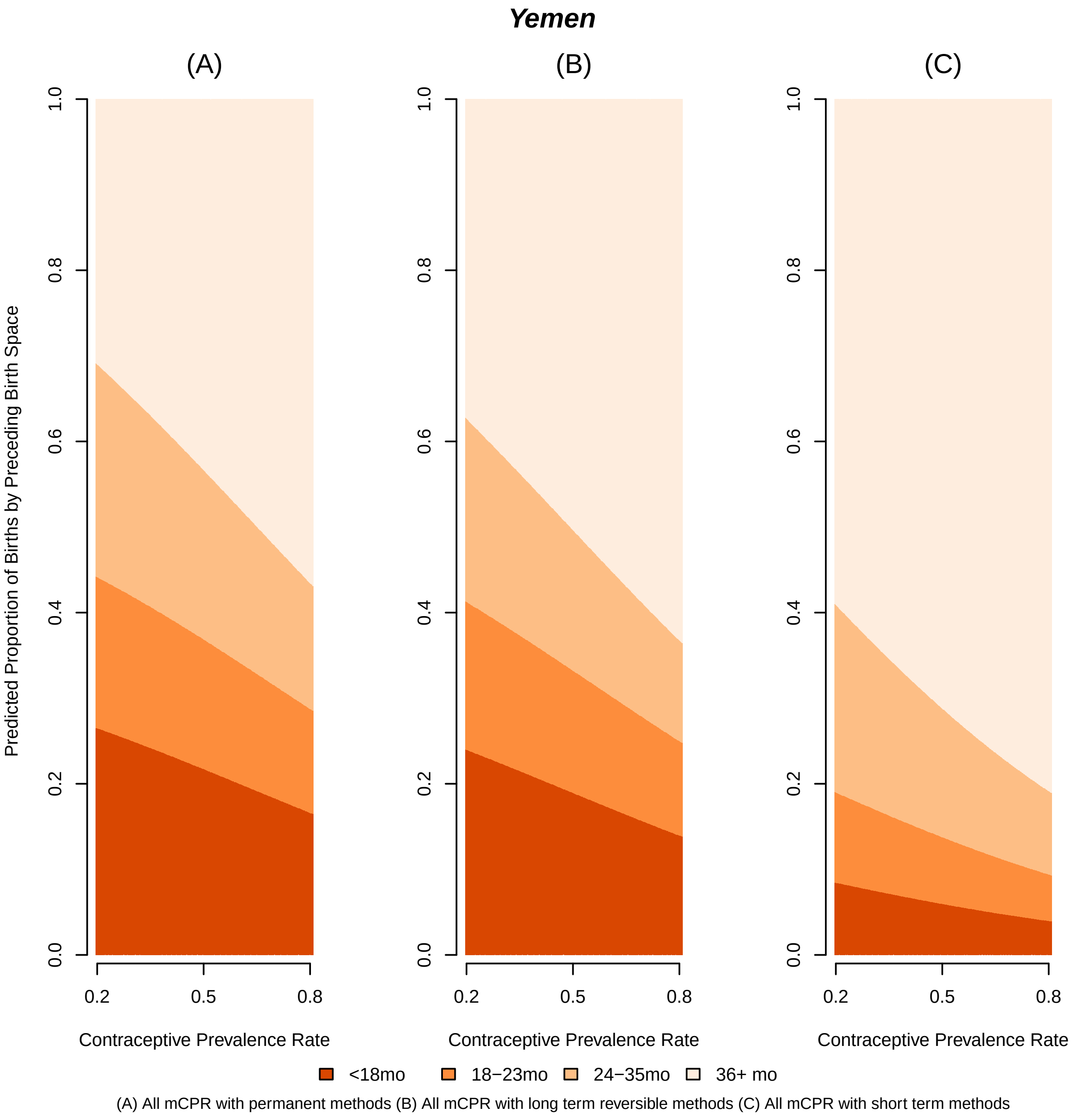
Yemen
(A)
(B)
(C)
Predicted Proportion of Births by Preceding Birth Space
0.0
0.2
0.4
0.6
0.8
1.0
0.2
0.5
0.8
Contraceptive Prevalence Rate
<18mo
18−23mo
24−35mo
36+ mo
(A) All mCPR with permanent methods (B) All mCPR with long term reversible methods (C) All mCPR with short term methods

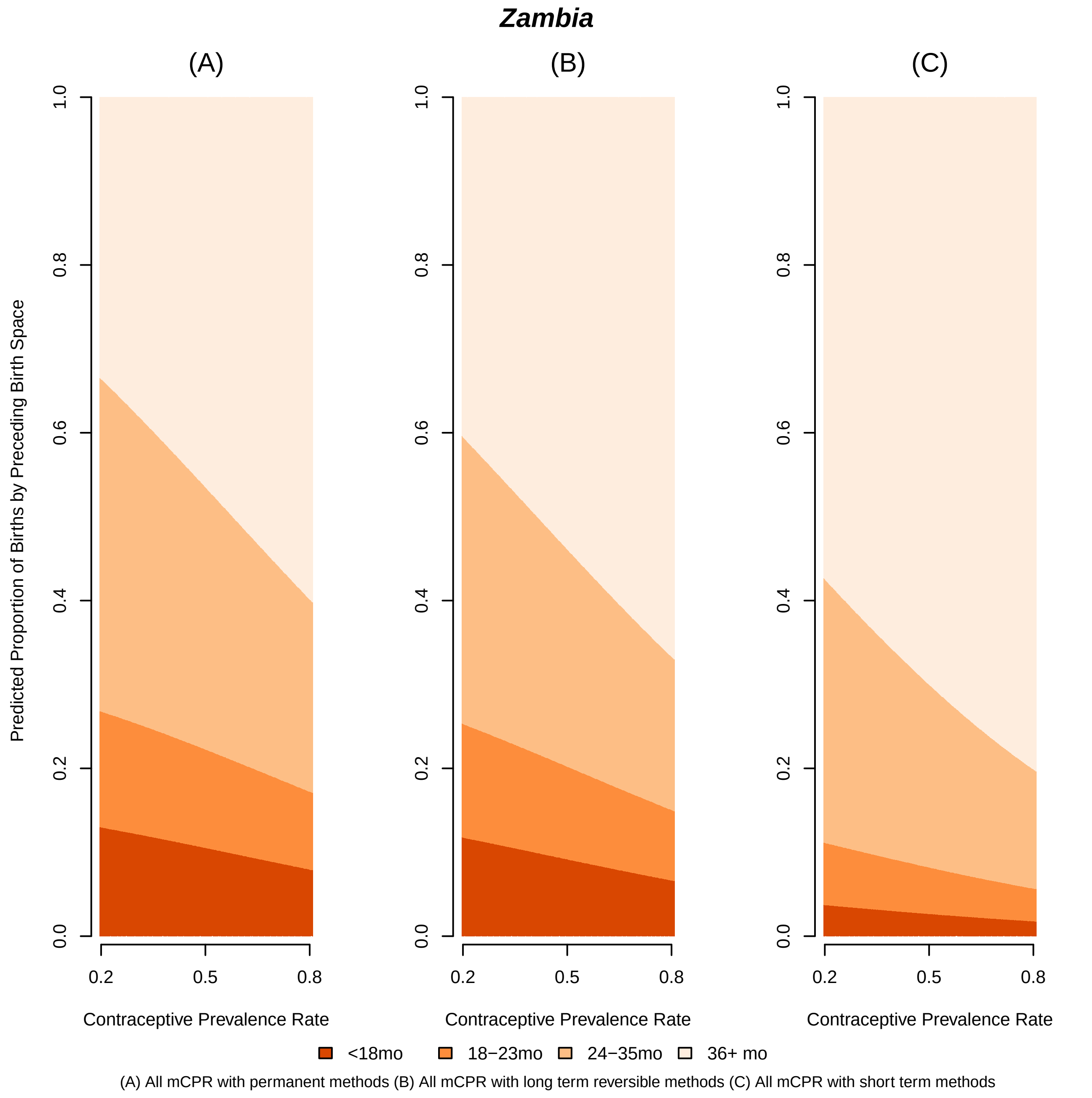
Zambia
(A)
(B)
(C)
Predicted Proportion of Births by Preceding Birth Space
0.0
0.2
0.4
0.6
0.8
1.0
0.2
0.5
0.8
Contraceptive Prevalence Rate
<18mo
18−23mo
24−35mo
36+ mo
(A) All mCPR with permanent methods (B) All mCPR with long term reversible methods (C) All mCPR with short term methods

Zimbabwe

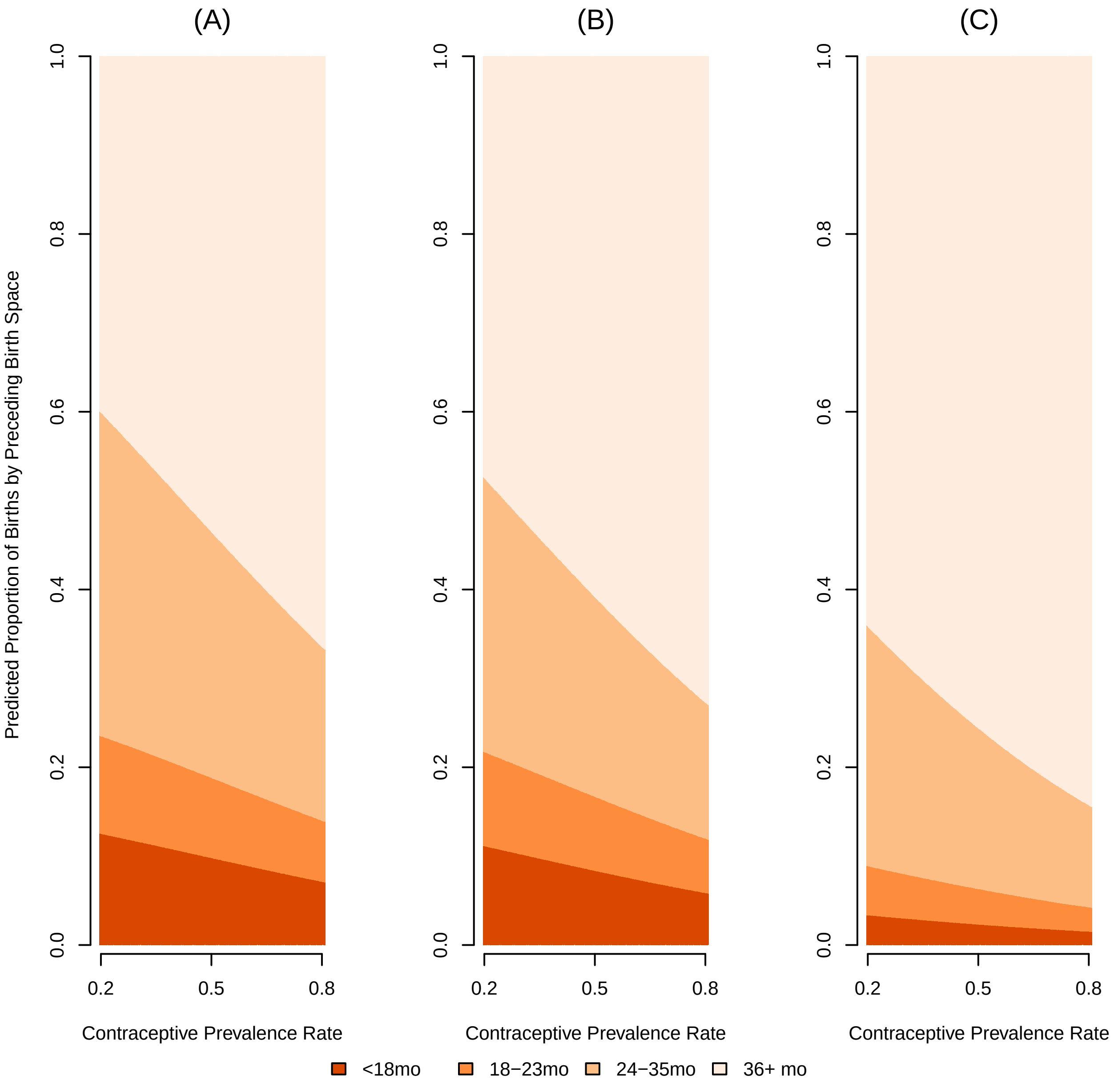

(A) All mCPR with permanent methods (B) All mCPR with long term reversible methods (C) All mCPR with short term methods
